# Pushing the Limits of Neutral Organic Electron Donors: A Tetra(iminophosphorano)-Substituted Bispyridinylidene

**DOI:** 10.1002/anie.201505378

**Published:** 2015-07-24

**Authors:** Samuel S Hanson, Eswararao Doni, Kyle T Traboulsee, Graeme Coulthard, John A Murphy, C Adam Dyker

**Affiliations:** Department of Chemistry, University of New Brunswick Fredericton, New Brunswick, E3B 5A3 (Canada); WestCHEM, Department of Pure and Applied Chemistry, University of Strathclyde 295 Cathedral Street, Glasgow G1 1XL (UK)

**Keywords:** electron transfer, iminophosphoranes, organic reductants, reduction, substituent effects

## Abstract

A new ground-state organic electron donor has been prepared that features four strongly π-donating iminophosphorano substituents on a bispyridinylidene skeleton. Cyclic voltammetry reveals a record redox potential of −1.70 V vs. saturated calomel electrode (SCE) for the couple involving the neutral organic donor and its dication. This highly reducing organic compound can be isolated (44 %) or more conveniently generated in situ by a deprotonation reaction involving its readily prepared pyridinium ion precursor. This donor is able to reduce a variety of aryl halides, and, owing to its redox potential, was found to be the first organic donor to be effective in the thermally induced reductive S–N bond cleavage of *N*,*N*-dialkylsulfonamides, and reductive hydrodecyanation of malonitriles.

Recently, organic electron donors^1^–^7^ such as **A** (*E*_1/2_=−1.20 V vs. SCE) and **Ba** (*E*_1/2_=−1.24 V vs. SCE), have emerged as exciting new reagents in organic synthesis (see Scheme 1). Such ground-state, neutral organic molecules are associated with exceptionally negative redox potentials, yet are soluble and tunable, and should therefore complement traditional heterogeneous metal-based reductants in that they can offer alternate reaction conditions (including the absence of metallic by-products) or unique selectivity.^8^, ^9^ To date, these reagents have been effectively used in the reduction of organic substrates such as aryl halides,^5^, ^7^, ^10^, ^11^ sulfones and arenesulfonamides,^7^, ^12^ Weinreb amides,^13^ acyloin derivatives,^14^ triflates, and triflamide.^15^ Until recently, such reductions would only have been expected from strong inorganic reducing agents such as alkali metals or samarium(II) species.^16^–^18^

**Scheme 1:**
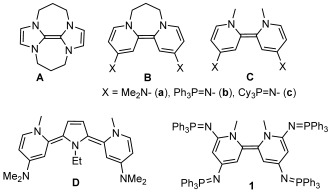
Structure of organic electron donors A, B, C, D, and 1.

The utility and power of these organic donors has been further increased by their photoexcitation. Indeed, photoactivation of **Ba** allows for the reduction of challenging substrates such as activated benzenes, *N*,*N*-dialkyl arenesulfonamides, benzylic esters and ethers, benzyl malonates and cyanoacetates,^19^–^22^ which could not be reduced by **Ba** in the ground state (see Scheme 1). To complement these achievements with photoactivation, it is desirable to expand the library of known organic reducing agents, particularly into the realm of increasingly negative redox potentials so that increasingly difficult reductions can be effected from the ground state.

Our groups have recently described novel bis(iminophosphorano)-substituted bispyridinylidenes **Bb**, **Bc**, **Cb**, and **Cc**^23^ as well as tricyclic **D**,^24^ all of which act as two electron donors. These compounds represent the most powerful organic reducing agents yet reported, with redox potentials reaching −1.50 V (**D**) and −1.51 V (**Cc**) vs. SCE (see Scheme 1).^23^, ^24^ Whereas the extrinsic effect of solvation is highly important in governing the redox potential of the alkali metals,^25^ the strongly reducing nature of these compounds is attributed to the formation of aromatic rings upon their oxidation to the respective dications, as well as the intrinsic effect of the exceptional π-donating substituents, with iminophosphorano groups being more powerful in this regard than typical amino substituents.^23^ Here we report on the effect of incorporating four iminophosphorano groups onto the bispyridinylidene skeleton, as in donor **1**, which provides potentials reaching −1.70 V vs. SCE (see Scheme 1). The utility of **1** as a ground-state electron donor is demonstrated in the reduction of challenging sulfonamides, aryl halides, and malononitriles, including substrates which have proven inert to previous organic donors, except with photoactivation.

Before attempting the preparation of **1**, we targeted bispyridinylidene **4** (Scheme 2) derived from 2-iminophosphoranopyridine **2**, in order to assess the effect of an iminophosphorano substituent in this position. Pyridine **2** is known,^26^ and can be easily prepared in 80 % yield on a 20 g scale. Gratifyingly, the addition of 1,3-diiodopropane to two equivalents of this pyridine cleanly afforded the bispyridinium diiodide **3**, which was isolated in 83 % yield. Exclusive alkylation at the pyridyl nitrogen is in line with previous observations involving **2**,^27^ but contrasts the analogous reaction with 2-(dimethylamino)pyridine, where both the pyridyl and exocyclic nitrogen centers were alkylated.^28^ Subsequently, the reaction of **3** with two equivalents of KN(SiMe_3_)_2_ (KHMDS) cleanly produced the desired iminophosphorano-substituted donor **4**, though it could only be isolated in low yield (12 %) owing to its poor solubility. Nevertheless, the isolated quantities were sufficient to allow for its chemical oxidation with hexachloroethane to **4^2+^-2 Cl^−^** and subsequent electrochemical analysis by cyclic voltammetry. In this way, redox potentials of −1.25 and −1.08 V vs SCE were determined for the **4^+^**/**4** and **4^2+^**/**4^+^** couples, respectively. Though **4** should still be considered a relatively strong donor, these potentials are less reducing than for **Bb** (*E*^1^_1/2_=−1.36 V, *E*^2^_1/2_=−1.23 V vs. SCE),^23^ showing that the bispyridinylidene framework is less sensitive to substitution at the 2-, rather than the 4-position, of the pyridyl ring.

**Scheme 2:**
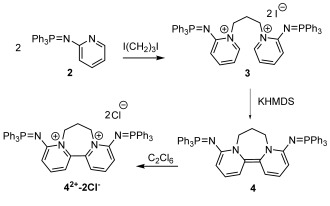
Synthesis of iminophosphorano-substituted bispyridinylidene 4, and its oxidation to the corresponding dichloride 4^2+^-2 Cl^−^.

Encouraged by the successful preparation of **4**, we then prepared tetrasubstituted donor **1** (Scheme 3, see the Supporting Information for the propylene-bridged analog of **1**). Initially, the 4-iminophosphorano functionality was introduced by the addition of **5** to a dichloromethane solution containing triethylamine and in situ generated dibromotriphenylphosphorane. The resulting chloropyridine **6** was isolated on a 60 g scale in 89 % yield, and was subsequently methylated at the pyridyl nitrogen to give chloropyridinium salt **7** (26 g, 96 %). A combination of 1,8-diazabicyclo[5.4.0]undec-7-ene (DBU) and aminotriphenyl-phosphonium bromide was then used to generate nucleophilic Ph_3_P–NH, which in the presence of excess DBU, was able to substitute the chloride of **7** to give pyridinium salt **8** (20 g, 66 % after recrystallization). The preparation of **1** was completed by the deprotonation of **8** with KHMDS in toluene. After three hours, donor **1**, which is virtually insoluble in toluene, was isolated in a 44 % yield after being collected by filtration and extracted into benzene. ^31^P{^1^H} and ^1^H NMR spectra of the isolated solid show that **1** occurs as a 2:1 mixture of *Z* (^31^P: 0.4 and −7.8 ppm) and *E* (^31^P: −1.3 and −5.4 ppm) isomers. The preference for the *Z* isomer is supported by ROESY NMR experiments, and is in line with previous experimental^23^ and theoretical^29^ investigations on bispyridinylidenes. The low isolated yield for **1** should not be regarded as a major disadvantage, as the donor can be effectively used as a reductant when generated in situ. As for **4**, donor **1** was oxidized to its more stable dichloride salt by its reaction with hexachloroethane, and analyzed by cyclic voltammetry. This electrochemical analysis revealed a half-wave potential of −1.70 V for the **1^2+^**/**1** couple, making **1** the strongest neutral organic electron donor by a substantial margin (190 mV more powerful than **Cc**, and over 450 mV more powerful than **Ba**).

**Scheme 3:**
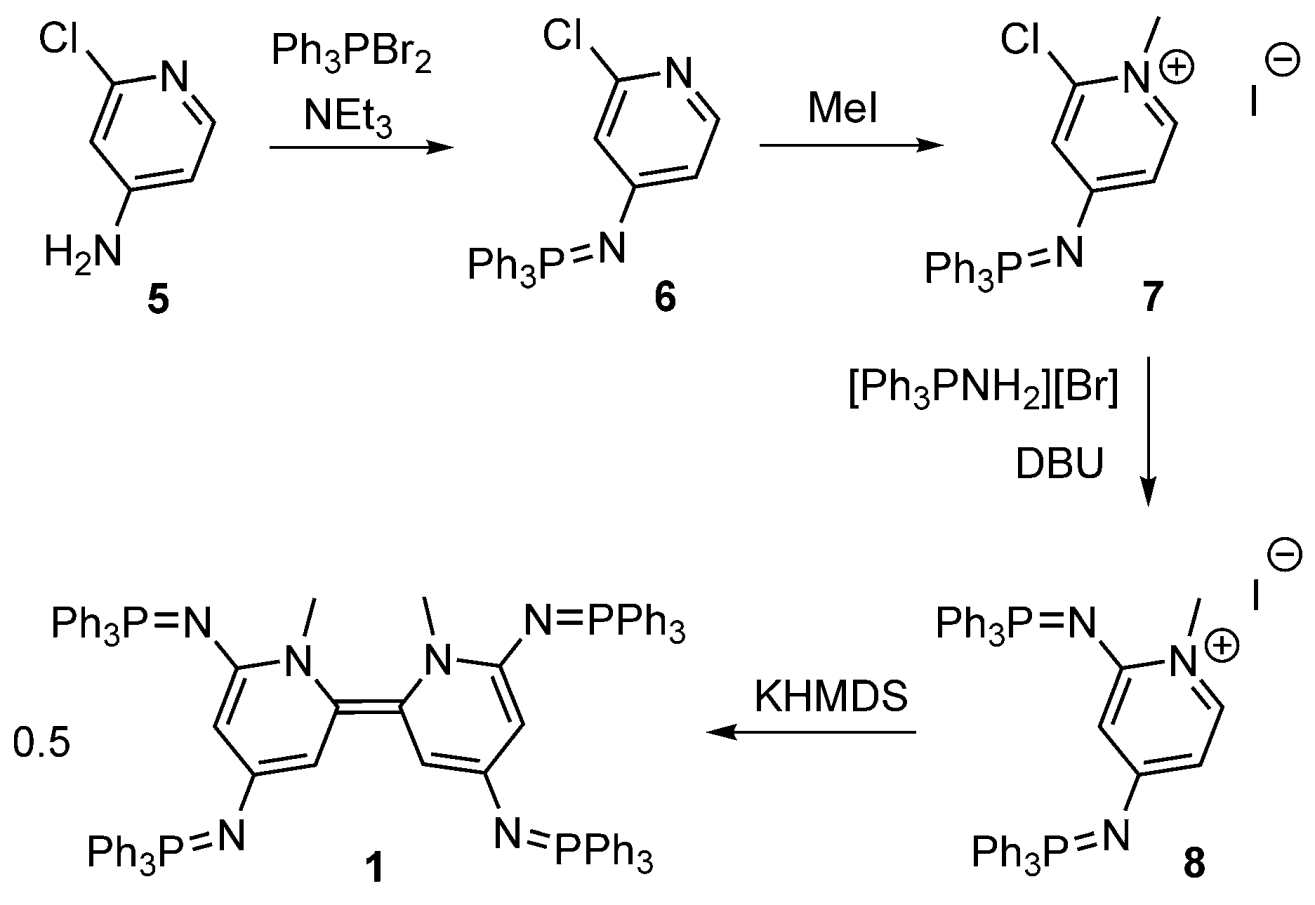
Preparation of tetrasubstituted organic electron donor 1 (only the major *Z* isomer is shown).

Owing to the superior reducing power of **1** over **A** and **Ba**, we were particularly keen to investigate the use of **1**, generated in situ from an equimolar mixture of **8** and KHMDS, in the reductive S–N bond cleavage of sulfonamides (Scheme 4). In arenesulfonamide deprotections, the ease of reductive cleavage increases with the stability of the nitrogen leaving group, and so *N*,*N*-dialkyl arenesulfonamides had proven to be amongst the toughest of substrates to deprotect by previous donors under thermal activation. For example, dialkyl arenesulfonamide **9**, which lacks any π-system to stabilize N-containing leaving group, proved to be unreactive to **A** in the ground state (110 °C, 18 h), but was reduced to **10** in 65 % yield by **Ba** (6 equiv) after 72 h of photoexcitation.^22^ Gratifyingly, even with 8 equivalents of **8** (equating to at most 4 equiv of **1**), amine **10** was produced in good yield (75 %) within 24 h at 110 °C. Compound **11 a** proved more challenging, but with eight equivalents of donor-precursor **8** (4 equiv of **1**), yields of **12** (56 %) comparable to those achieved using 6 equiv of **Ba** under photolysis (59 %) were achieved.^22^ As expected, yields of **12** from the reduction of mesyl-substituted **11 b** (6 %) were much lower than were achieved from tosyl-derived **11 a**, owing to the absence of the relatively low-energy LUMO of the arene fragment in **11 b**. Nevertheless, the outcomes are a testament to the strength of ground-state donor **1**, which is the first ground-state organic electron donor able to effect the reduction of dialkylsulfonamides.

**Scheme 4:**
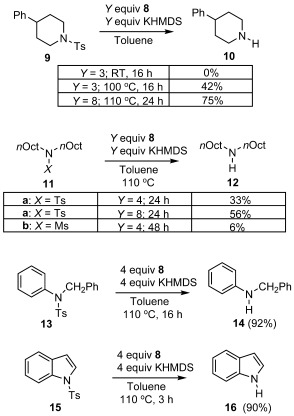
The reduction of sulfonamides by in situ generated 1 (Ts=tosyl group and Ms=mesyl group).

Moving to more activated sulfonamides, two equivalents of the in situ generated donor gave high yields of deprotected products **14** (92 %) and **16** (90 %) from compounds **13** and **15**, respectively. The reduction of these substrates, which is facilitated by the formation of a resonance-stabilized nitrogen-containing leaving group, has been previously accomplished by donor **A** (albeit with six equivalents of donor).^12^

In the case of aryl halides (Scheme 5), donor **1** (2 equiv) reduces iodides **17 a** and **19 a** at room temperature, to products **18** and **20**/**21**, respectively, where the formation of **21** suggests the involvement of aryl anion intermediates. Recent computational studies^30^ suggest that the reduction potential for aryl radicals to form aryl anions is about −1 V vs. SCE, which is considerably more negative than the original experimental estimate,^31^ but this potential would still be easily reached by donor **1**. Importantly, under otherwise identical conditions, **17 a** was quantitatively recovered in the absence of **8**, demonstrating the necessity of donor **1** in effecting the reductions.

**Scheme 5:**
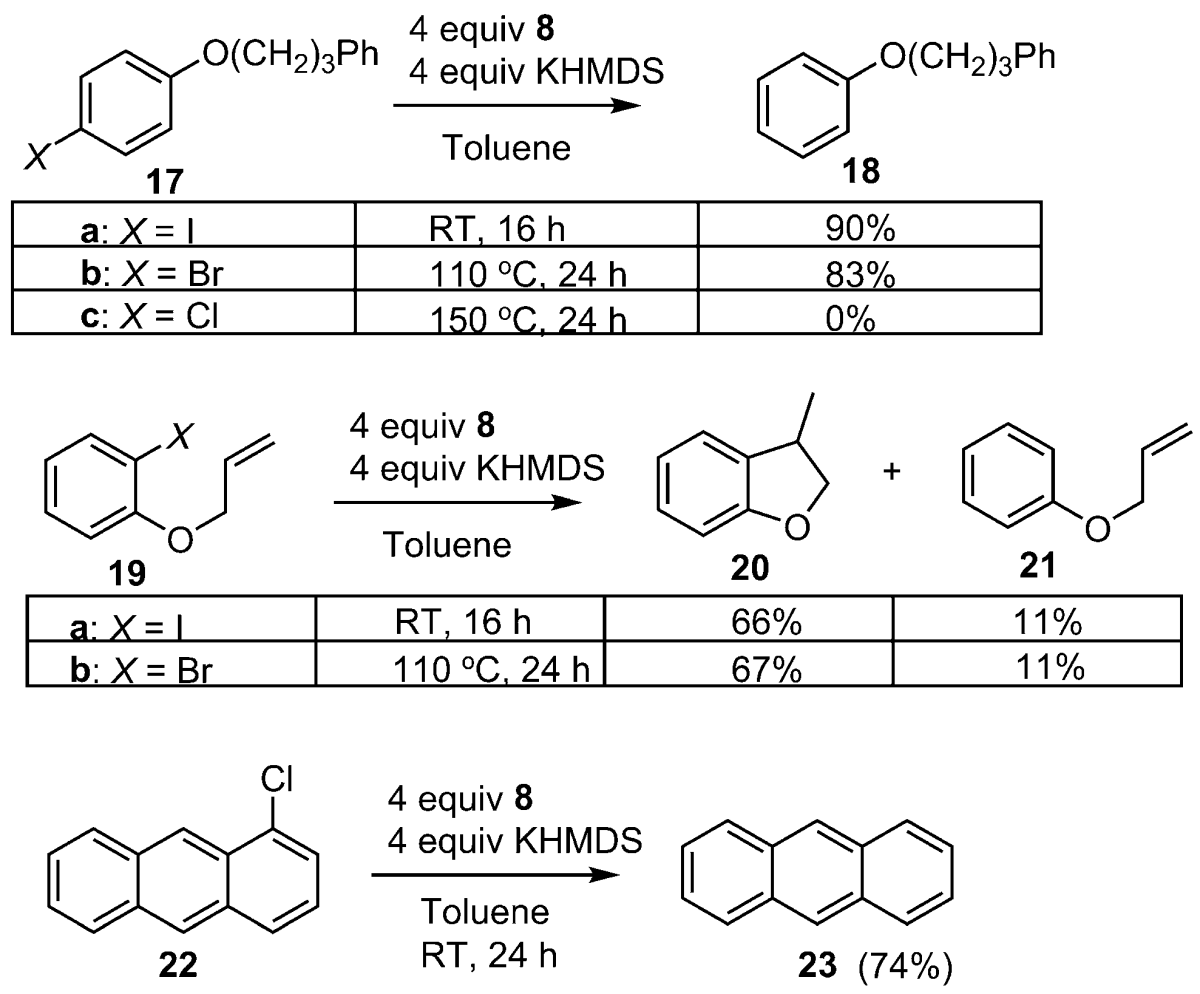
The reduction of aryl halides by in situ generated 1.

The reduction of iodides **17 a** and **19 a** have been similarly effected by a number of organic donors,^7^, ^10^, ^32^ including **A**, **Ba**, and **Ca**, so more challenging aryl halide substrates were also investigated. The related bromides **17 b** and **19 b** were reduced by donor **1** at higher temperatures and with longer reaction times (110 °C, 24 h), but the chloride **17 c** remained inert to **1** under thermal conditions. In contrast, 1-chloroanthracene **22**, with a lower energy LUMO owing to the extended π system, was easily reduced to anthracene **23** (RT, 24 h).

These encouraging results prompted the investigation of the effectiveness of **1** in the hydrodecyanation of malononitriles (Scheme 6). Such a process is typically conducted through the use of tributyltin hydride/α,α′-azobisisobutyronitrile (AIBN),^33^, ^34^ or SmI_2_ in hexamethylphosphoramide,^35^ but it has more recently been effected by N-heterocyclic carbene boranes/radical initiator,^36^ or by **Ba** under photoactivated conditions.^37^ No organic electron donor has achieved this reduction from its ground state. With comparable yields to those achieved by **Ba**, compound **1** is able to effectively hydrodecyanate malononitriles **24**, **26**, and **28** to give the respective mononitriles **25** (92 %), **27** (91 %), and **29** (89 %). The lack of cyclized product in the case of **26** is in line with expectations; an initially formed radical intermediate **33**, formed from generalized malononitrile substrate **32** should be easily reduced to the corresponding anion **34** under the heavily reducing reaction conditions.^36^ These anionic mononitrile products would be inert to further reduction, allowing isolation of the mononitrile products **25**, **27** and **29**, in excellent yields. Neutral mononitrile **30** is also inert to reduction under these conditions, as was demonstrated in a separate reaction.

**Scheme 6:**
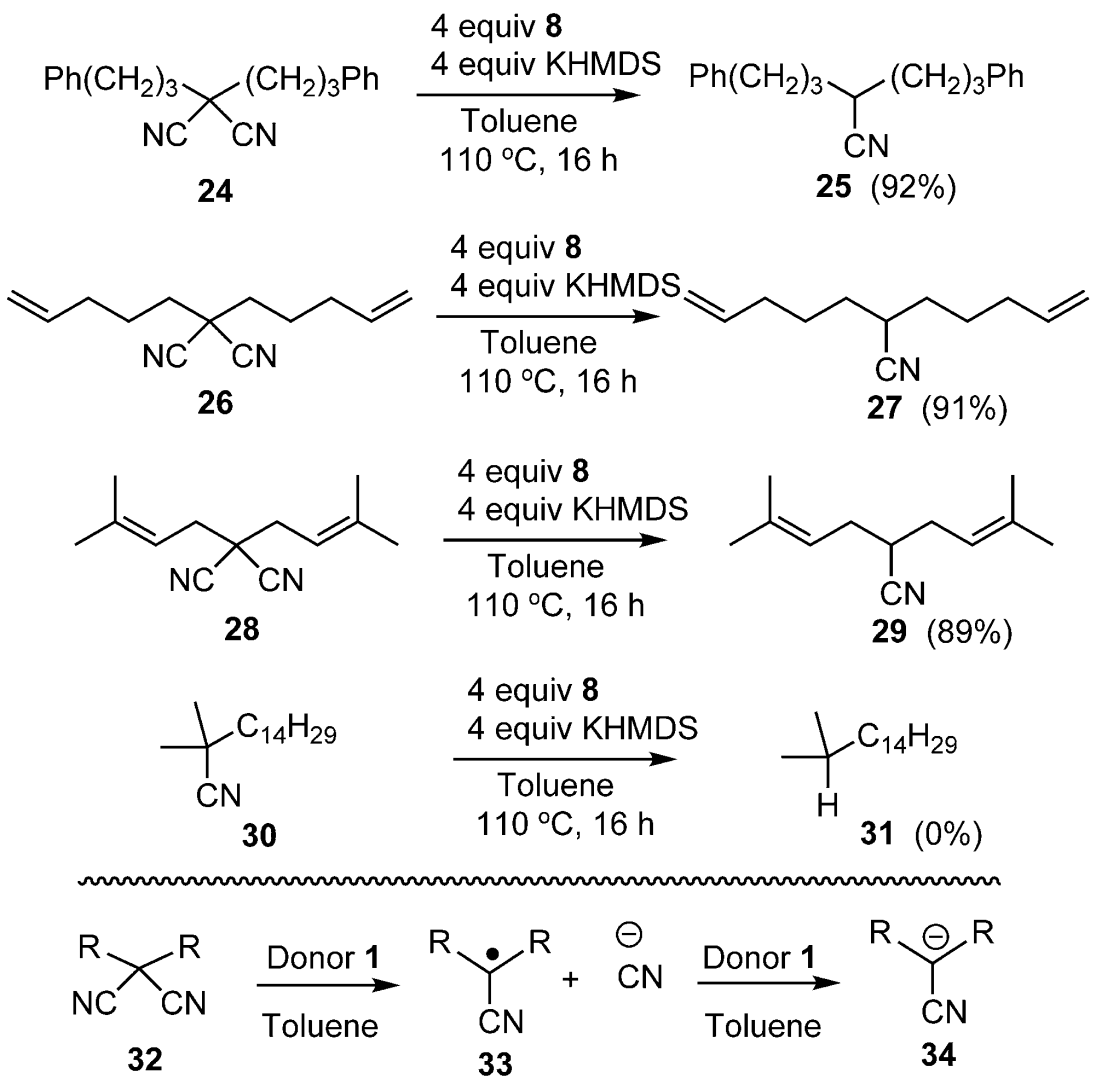
The reduction of malononitriles by in situ generated 1.

In conclusion, tetra(iminophosphorano)-substituted bispyridinylidene **1** represents the most reducing organic neutral compound known, with its redox potential surpassing the previous record holder by 190 mV. It is the only organic electron donor with the ability to reduce dialkylarenesulfonamides as well as malononitriles without photoexcitation. Further reductions involving donor **1** are currently under investigation.

## Supporting Information

As a service to our authors and readers, this journal provides supporting information supplied by the authors. Such materials are peer reviewed and may be re-organized for online delivery, but are not copy-edited or typeset. Technical support issues arising from supporting information (other than missing files) should be addressed to the authors.

miscellaneous_information
