# Supplementary material for: Pushing the Limits of Neutral Organic Electron Donors: A Tetra(iminophosphorano)-Substituted Bispyridinylidene
Source: Angew Chem Int Ed Engl. 2015 Jul 24;54(38):11236–9. doi: 10.1002/anie.201505378 (PMC4581462; doi:10.1002/anie.201505378)
Supplement: Supplementary file 1 — miscellaneous_information [file anie0054-11236-sd1.pdf]

Angewandte Chemie
Eine Zeitschrift der Gesellschaft Deutscher Chemiker

Supporting Information

Pushing the Limits of Neutral Organic Electron Donors: A Tetra(iminophosphorano)-Substituted Bispyridinylidene

Samuel S. Hanson, Eswararao Doni, Kyle T. Traboulsee, Graeme Coulthard, John A. Murphy, and C. Adam Dyker**

anie_201505378_sm_miscellaneous_information.pdf

Table of Contents

1. General Information…………………………………………………………………S-2

2. Preparation of Electron Donors and Related Compounds………………..………….S-4

3. Experimental Procedures for Reduction Reactions ……………………….……..…….S-16

4. Electrochemical Studies of 1, S1a, and 4………………………………………………...S-32

5. References……………………………………………………………………………….S-34

6. Spectral Data…………………………………………………………………………….S-36

1. General Information

General Experimental Procedures

All reactions were performed in oven-dried or flame-dried reaction flasks/pressure tubes under an argon atmosphere using standard Schlenk line techniques or in an argon-filled Innovative Technology glove-box. All the solvents used were dried and degassed using the freeze-pump-thaw procedures and stored over 4 Å molecular sieves. Diethyl ether, tetrahydrofuran, dichloromethane and hexane were dried with a Pure-Solv 400 or Seca solvent purification systems. Dimethylformamide and acetonitrile were dried and distilled over calcium hydride prior to use. Deuterated solvents used for air and moisture sensitive compounds were dried and distilled over CaH_2 and degassed before use. A glove box (Innovative Technology Inc., U.S.A.) was used to generate the active donor in situ. An Ocean Optics USB2000+UV-Vis spectrometer was used to measure the UV-Vis spectrum of compound **1**.

A Büchi rotary evaporator was used to concentrate the reaction mixtures. Thin layer chromatography (TLC) was performed using aluminium-backed sheets of silica gel and visualized under a UV lamp (254 nm). The plates were developed using vanillin or $KMnO_4$ solution. Column chromatography was performed to purify compounds by using silica gel 60 (200-400 mesh).

Ferrocene that was used as internal standard for cyclic voltammetry was purchased from Sigma Aldrich and recrystallized from hexane, dried in *vacuo* and stored in the glove-box prior to use. Triphenylphosphine, 2-aminopyridine, 4-amino-2-chloropyridine and 1,8-diazabicyclo[5.4.0]undec-7-ene (DBU) were purchased from Sigma Aldrich and used without further purification. All other reagents were used as purchased from commercial suppliers and used without further purifications unless stated otherwise. 2-Triphenyliminophosphorane[1-3] and amino(triphenyl)phosphonium bromide[4-6] were prepared according to literature procedures with slight modifications.

Standard Analyses. Proton (^{1}H) NMR spectra were recorded at 25 ^{o}C on a 300 MHz Varian UNITY INOVA spectrometer or 400 MHz Varian UNITY spectrometer or 400 MHz Bruker DPX spectrometer or 500 MHz Bruker DRX spectrometer and referenced to residual protons of deuterated solvents (C_6D_6: ^{1}H, δ = 7.16, ^{13}C, δ = 128.06; $CDCl_3$: ^{1}H, δ = 7.26, ^{13}C, δ = 77.0; DMF-d_7: ^{1}H, δ = 8.03; ^{13}C, δ = 163.15, DMSO-d_6: ^{1}H, δ = 2.50; ^{13}C, δ = 39.5). Carbon NMR

(^{13}C) spectra were recorded at 101 MHz or 125 MHz. Phosphorus NMR (^{31}P) spectra were recorded at on a Varian UNITY INOVA 300 MHz or Varian UNITY 400 spectrometer at 121.4 MHz or 161.8 MHz respectively, with phosphoric acid H_3PO_4 (85%) as external standard (at δ 0 ppm). The chemical shifts are quoted in parts per million (ppm). Signal multiplicities are abbreviated as: s, singlet; d, doublet; t, triplet; q, quartet; m, multiplet; bs, broad singlet; coupling constants are given in Hertz (Hz).

Infra-Red spectra were recorded on a Perkin Elmer Spectrum One FT IR Spectrometer either pressed as discs in KBr or as films applied on NaCl crystal plates or using an ATR-IR spectrometer. An Ocean Optics USB2000+UV-Vis spectrometer was used to measure the UV-Vis spectrum of compound **1**. Melting points were determined on a Gallenkamp or DigiMelt Melting point apparatus. High resolution mass spectra were recorded at the EPSRC National Mass Spectrometry Service Centre, Swansea or at the Mass Spectrometry Laboratory at Dalhousie University, Halifax, NS, Canada. The spectra were recorded using electron ionization (EI), chemical ionization (CI), fast atom bombardment (FAB) or electrospray ionization (ESI) techniques, as stated for each compound.

Electrochemistry. Electrochemical studies were performed using a standard three-electrode system, using a Biologic SP-150. The working electrode was a platinum disc (diameter 0.07 cm^3) and the counter electrode was a platinum wire. The reference electrode was composed of a silver wire in a solution containing 0.01 M $AgNO_3$ and 0.1 M tetrabutylammonium hexafluorophosphate, (TBAH, $n\mathrm{Bu_4NPF_6}$) in acetonitrile. The CVs were conducted in DMF, with 0.1 M solution of TBAH as the supporting electrolyte, at scan rates of 50 mV/s with ferrocene as internal standard. The experiments were performed under an argon atmosphere at room temperature in the glove box.

2. Detailed Experimental Procedures

Synthesis of amino(triphenyl)phosphonium bromide, $[Ph_3PNH_2][Br]$

Amino(triphenyl)phosphonium bromide was prepared according to literature procedures,[4-6] but with slight modifications.

Acetonitrile (220 mL) was added to triphenylphosphine (35.000 g, 133.44 mmol) and the mixture heated to dissolution. Bromine (*ca* 7.0 mL, 135.89 mmol) was added dropwise to the colorless solution until a persistent yellow solution was obtained. After stirring for 30 min, ammonia gas was bubbled in for 30 min yielding an instantaneous white precipitate. The mixture was stirred further for 30 min. Diethyl ether (40 mL) was added and the white solid was collected by filtration. The solid was washed with diethyl ether (50 mL) and dried under vacuum to give an off white solid of $[Ph_3PNH_2][Br]$ and an equimolar amount of NH_4Br (78.5% $[Ph_3PNH_2][Br]$ by mass), in virtually quantitative yield (58.998 g; corresponding to = 46.311 g (129.3 mmol) of $[Ph_3PNH_2][Br]$). This mixture was used as isolated for the preparation of **8**. The spectral data for $[Ph_3PNH_2][Br]$ are as reported.

Modified synthesis of triphenyliminophosphorane, $Ph_3P{=}NH$[1-3] (for the preparation of **S8a**)

Sodium hydroxide (1M: 40 mL) was added to a slurry of the $[Ph_3PNH_2][Br]/NH_4Br$ mixture (1.847g of the mixture, corresponding to 1.450 g, 4.05 mmol of $[Ph_3PNH_2][Br]$) in dichloromethane (50 mL) and stirred for 2 min. The organic layer was collected after solvent extraction and was further extracted twice with distilled water (2 x 25 mL). The solution was concentrated to ca 5 ml, diethyl ether (10 ml) and hexane (20 mL) were added and the solid precipitate was collected by filtration and dried *in vacuo* to give a white solid. (1.015 g) comprising of a mixture of $Ph_3P{=}NH$ and $Ph_3P{=}O$ in a ratio of 5:1. This was used without further purification.

Synthesis of 2-(triphenyliminophosphorano)pyridine, 2

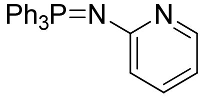

2

2-(triphenyliminophosphorano)pyridine, **2** has been synthesized previously using a different route[2] but was prepared as shown below.

Bromine (4.0 mL, 78.3 mmol) was added dropwise to a solution of triphenylphosphine (20.000 g, 76.3 mmol) in CH_2Cl_2 (170 mL) at 0 °C. The resulting yellow solution was stirred at 0 °C for 30 mins. Anhydrous triethylamine (23.00 mL, 163.8 mmol, 2.1 eq.) was added followed by 4-aminopyridine (7.185 g, 76.3 mmol). The resulting mixture was warmed to room temperature and stirred for 4 h. The reaction was quenched with distilled water (180 mL) and the aqueous phase was separated and discarded. The organic layer was further extracted using distilled water (3 x 180 mL), dried with $MgSO_4$ and filtered, before being concentrated to ~ 50 mL. Diethyl ether (150 mL) was added to a concentrated solution to precipitate the product which was subsequently collected by filtration and washed further with diethyl ether (50 mL). The filtrate was also concentrated (15 mL) and filtered. The two fractions of the product were combined and dried in *vacuo* to give **2** (21.484 g, 78%) as a light yellow solid. The ^{1}H, ^{13}C and ^{31}P NMR spectra were as reported.

Synthesis of Bispyridinium diiodide, 3

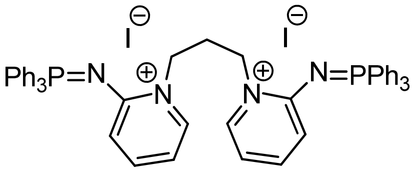

3

A mixture of **2** (12.342 g, 34.3 mmol) and 1,3-diiodopropane (1.80 mL, 15.7 mmol) in acetonitrile (65 mL) was heated to dissolution. The resulting solution was stirred at 90 °C for 18 h, cooled to room temperature and concentrated to 40 mL. Diethyl ether (140 mL) was added to precipitate the product which was subsequently filtered and the solid collected. After all volatiles had been removed, the solid was recrystallized from hot 2-propanol/acetonitrile (8:1) and dried *in vacuo* at 110 °C to give **3** (13.030 g, 83 %) as an off-white solid. mp >260 °C. ^{1}H NMR (400 MHz, $CDCl_3$): δ 2.69 (quint, 2H, CH_2, J = 7.2 Hz), 5.07 (t, 4H, 2 NCH_2, J = 7.2 Hz), 6.37 (d, 2H, PyrH, J = 8.8 Hz), 6.81 (td, 2H, PyrH, J = 6.8 and 1.2 Hz), 7.48 – 7.65 (m, 32H, PhH, PyrH), 8.77 (dt, 2H, J = 6.8 and 1.5 Hz). ^{13}C NMR (101 MHz, $CDCl_3$): δ 29.0 (CH_2), 51.1 (NCH_2), 114.0 (CH), 118.6 (d, CH, J = 8.9 Hz), 125.1 (d, C, J = 103.4 Hz), 130.0 (d, CH, J = 12.7 Hz),

132.3 (d, CH, J = 10.4 Hz), 134.1 (CH, J = 2.9 Hz), 142.8 (CH), 142.9 (CH), 156.6 (d, C, J = 10.3 Hz). ^{31}P NMR (121 MHz, $CDCl_3$): δ 18.6 ppm. **HRMS** (ESI+): m/z calcd for $C_{49}H_{44}N_4P_2$: 375.1515 $[M]^{2+}$; found: 375.1515

Synthesis of Bispyridinylidene 4

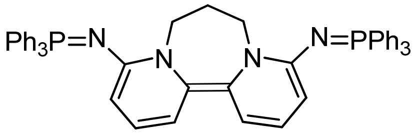

4

Toluene (20 mL) was added to a mixture of diiodide **3** (1.500 g, 1.49 mmol) and KHMDS (0.596 g, 2.99 mmol) and stirred for 1h at room temperature. After taking out an aliquot for ^{1}H and ^{31}P NMR (see Figure **S1a-b** below), the purple-colored mixture was subsequently filtered and the solid washed with benzene (30 ml) and hexane (20 mL). The filtrate was collected and dried *in vacuo* to give **4** (0.136 g, 12%) as a purple solid. The ^{1}H NMR of the solid in deuterated benzene showed broad and almost indiscernible peaks in the phenyl region but no peak in the pyridyl region and in the ^{31}P NMR spectra (see Figure **S1c-d** below). ^{1}H NMR (300 MHz, C_6D_6): δ 2.51 (quint, 2H, CH_2, J = 5.7 Hz), 4.29 (d, 2H, PyrH, J = 6.0 Hz), 4.66 (t, 4H, 2 NCH_2, J = 5.7 Hz), 5.72 (dd, 2H, PyrH, J = 9.3 Hz, 6.0 Hz), 6.01 (d, 2H, PyrH, J = 9.3 Hz), 6.95 -7.03 (m, 18H, PhH), 7.71 – 7.78 (m, 12H, ArH). ^{31}P NMR (121 MHz, C_6D_6): δ 4.7 ppm.

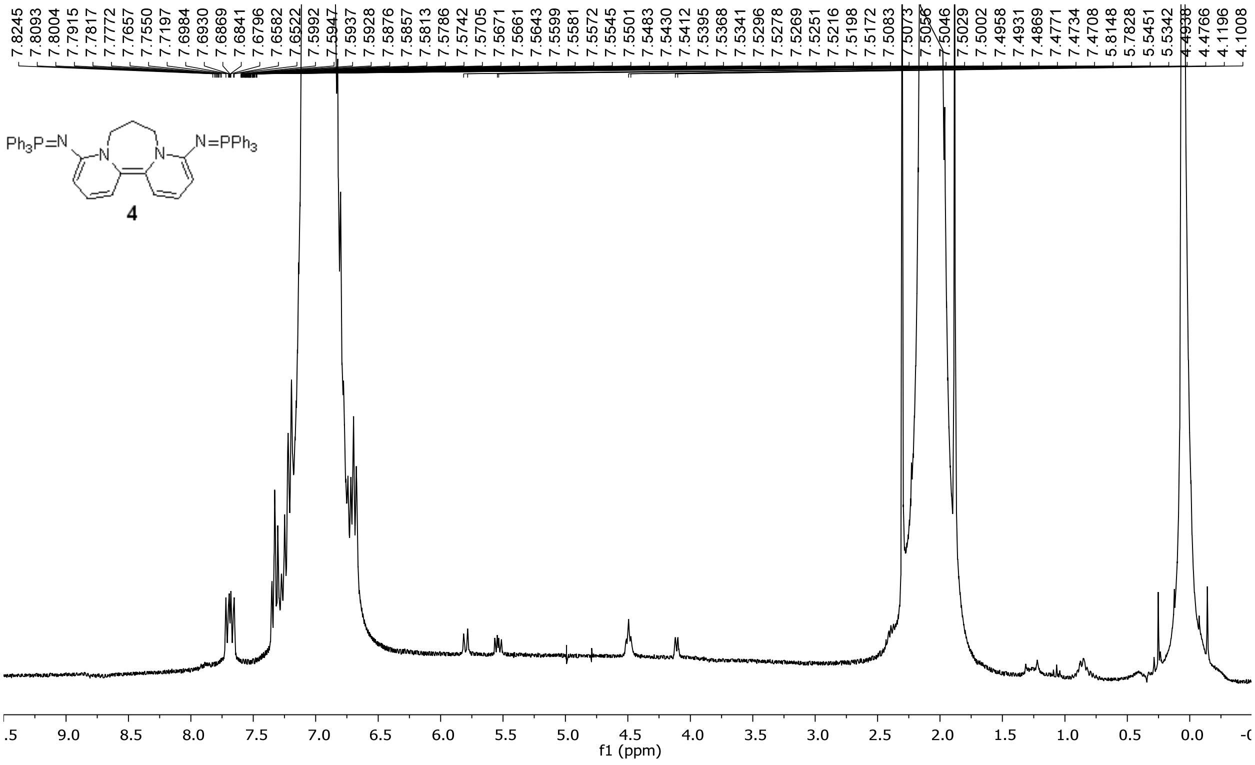

Figure S1a. ^{1}H NMR of reaction mixture of compound **4** in toluene

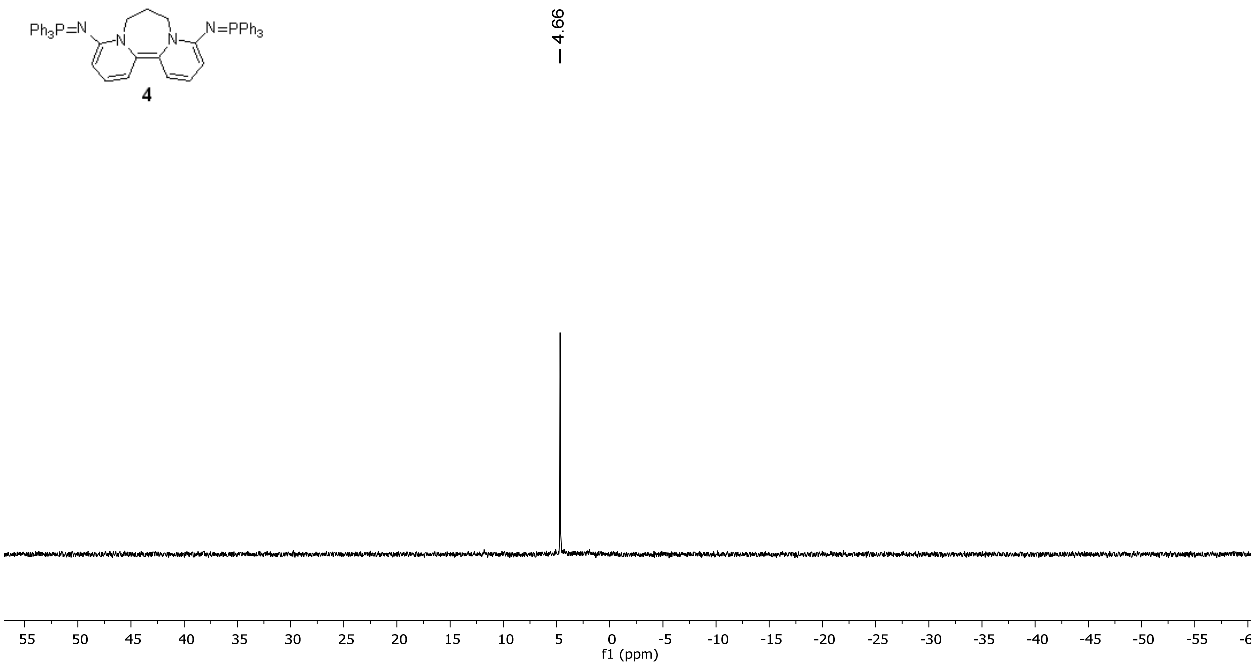

Figure S1b. ^{31}P NMR of reaction mixture of compound **4** in toluene

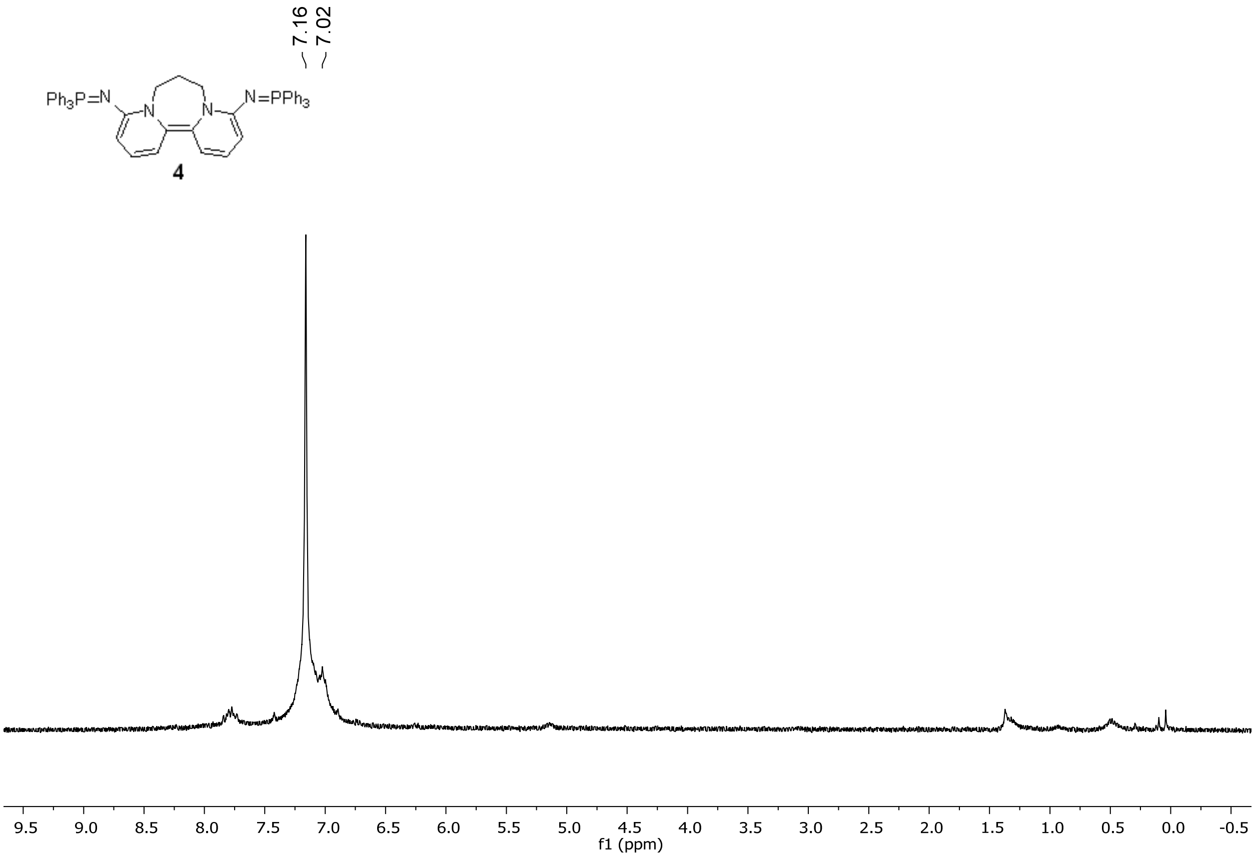

Figure S1c. ^{1}H NMR of purple solid of **4** in C_6D_6

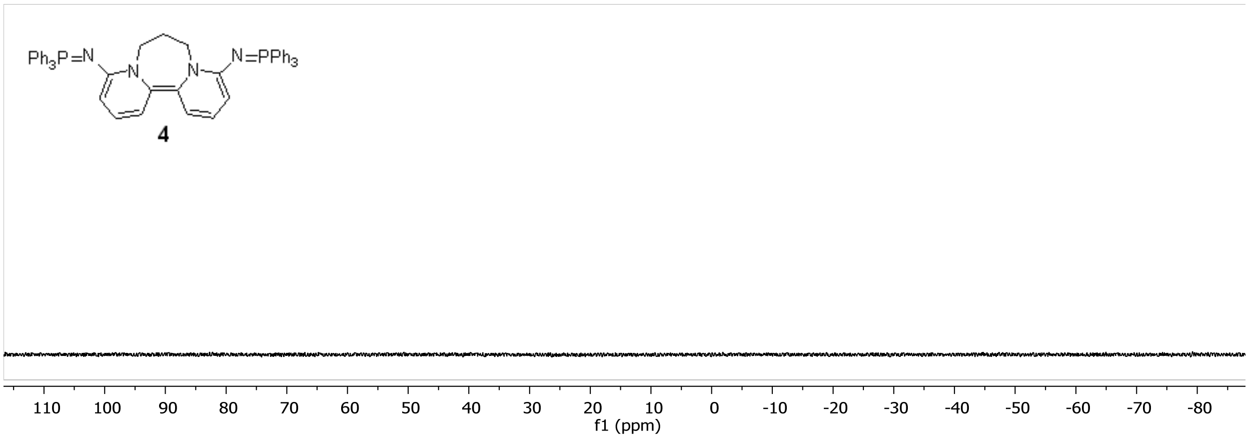

Figure S1d. ^{31}P NMR of purple solid of **4** in C_6D_6

Synthesis of bispyridinium dichloride, 4^{2+}-2Cl$^-$

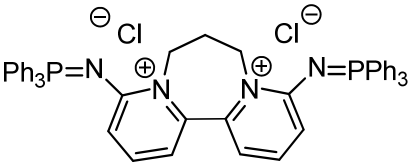

4^{2+}-2Cl$^-$

Hexachloroethane (0.046 g, 0.198 mmol) was added to a solution of **4** (0.096 g, 0.13 mmol) in benzene (2 ml) and stirred overnight. Diethyl ether (5 mL) was added and the precipitate filtered and washed further with diethyl ether (5 mL). The solid was collected and dried in *vacuo* (50 °C) to give **4^{2+}-2Cl$^-$** as brown solid. mp 191-194 °C (dec). ^{1}H NMR (300 MHz, $CDCl_3$): δ 2.64-2.78 (m, 2H, CH_2), 4.20-4.56 (m, 2H, 2 NCH_2), 5.76-5.82 (m, 2H, 2 NCH_2), 6.53 (d, 2H, PyrH, J = 9.0 Hz), 7.15 (d, 2H, PyrH, J = 7.2 Hz), 7.57 (dd, 2H, J = 9.0 and 1.5 Hz), 7.57 – 7.81 (m, 30H, ArH). ^{13}C NMR (101 MHz, $CDCl_3$): δ 26.8 (CH_2), 45.8 (NCH_2), 116.7 (CH), 120.8 (d, CH, J = 8.4 Hz), 125.2 (d, C, J = 103.8 Hz), 130.0 (d, CH, J = 12.8 Hz), 132.7 (d, CH, J = 10.9 Hz), 134.0 (d, CH, J = 2.9 Hz), 142.1 (CH), 144.0 (C), 156.8 (d, C, J = 11.3 Hz). ^{31}P NMR (121 MHz, $CDCl_3$): δ 19.6 ppm. **HRMS** (ESI+): m/z calcd for $C_{49}H_{42}N_4P_2$: 374.1437 $[M]^{2+}$; found: 374.1441. **Reduction Potential**: −1.25 V and −1.08 V versus SCE.

Synthesis of 2-Chloro-4-(triphenyliminophosphorano)pyridine, 6

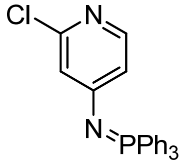

6

Bromine (9.50 mL, 187.30 mmol) was added dropwise to a solution of triphenylphosphine (47.700 g, 181.20 mmol) in dichoromethane (300 mL) at 0°C under the flow of argon. The resulting yellow solution was stirred at 0°C for 30 min. Anhydrous triethylamine (63.40 mL, 451.20 mmol, 2.5 eq.) was added, followed by 4-amino-2-chloropyridine (22.140 g, 172.20 mmol) at 0°C. After warming to room temperature, the mixture was stirred for 16h, quenched with distilled water (250 mL) and extracted. The aqueous phase was separated and discarded. The organic layer was further extracted using distilled water (250 mL x 4 times). The organic layer was dried with $MgSO_4$, filtered and concentrated. Diethyl ether (180 mL) was added to the

concentrated solution to precipitate the product which was subsequently collected by filtration, and washed with diethyl ether (80 mL). The filtrate was also collected, concentrated (60 mL) and the product precipitated with diethyl ether (100 mL), and washed with diethyl ether (20 mL). Both fractions were dried in *vacuo* to give **6** (59.836 g, 89%) as an off-white solid. mp 173-175 °C. ^{1}H NMR (300 MHz, $CDCl_3$): δ 6.45 (ddd, 1H, PyrH, J = 5.7, 2.1, 0.8 Hz), 6.58 (ddd, 1H, PyrH, J = 2.1, 0.8, 0.4 Hz), 7.44-7.54 (m, 6H, ArH), 7.54-7.63 (m, 3H, ArH), 7.66-7.76 (m, 6H, ArH), 7.79 (ddd, 1H, PyrH, J = 5.7, 1.2, 0.4 Hz). ^{13}C NMR (101 MHz, $CDCl_3$): δ117.7 (d, CH, J = 20.3 Hz), δ117.8 (d, CH, J = 18.6 Hz) 129.2 (d, C, J = 100.3 Hz), 129.1 (d, CH, J = 12.0 Hz), 132.4 (CH), 132.7 (d, CH, J = 10.0 Hz), 148.8 (CH), 151.6 (C), 161.5 (C). ^{31}P NMR (121 MHz, $CDCl_3$): δ 9.1 ppm. **HRMS** (ESI+): m/z calcd for $C_{23}H_{19}ClN_2P$: 389.0974 $[M+H]^+$; found: 389.0966 $[M+H]^+$

Synthesis of 2-Chloro-N-methyl-4-(triphenyliminophosphorano)pyridinium iodide, 7

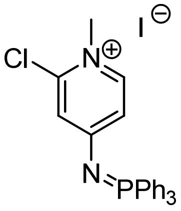

7

A mixture of **6** (20.112 g, 51.72 mmol, 2.2 eq.) and iodomethane (3.65 mL, 56.84 mmol) in acetonitrile (140 mL) was heated to dissolution. The solution was stirred at 85 °C for 18 h. After cooling, the volume was reduced to *ca.* 30 mL and diethyl ether (160 mL) was added to precipitate the product which was collected by filtration, washed with diethyl ether (30 mL) and dried under vacuum to afford **7** (26.264 g, 96 %) as off-white to pale yellow solid. mp 184-186 °C. ^{1}H NMR (300 MHz, $CDCl_3$): δ 4.14 (s, 3H, CH_3), 6.44 (dd, 1H, PyrH, J = 7.2, 2.7 Hz), 6.86 (d, 1H, PyrH, J = 2.7 Hz), 7.56-7.69 (m, 15H, ArH), 8.81 (d, 1H, PyrH, J = 7.2 Hz). ^{13}C NMR (101 MHz, $CDCl_3$): δ 44.2 (CH_3), 116.9 (d, CH, J = 16.9 Hz), 120.7 (d, CH, J = 24.0 Hz), 125.6 (d, C, J = 101.8 Hz), 129.9 (d, CH, J = 12.0 Hz), 132.6 (d, CH, J = 10.5 Hz), 133.9 (CH), 143.5 (C), 145.5 (CH), 166.4 (C). ^{31}P NMR (121 MHz, $CDCl_3$): δ 16.3 ppm. **HRMS** (ESI+): m/z calcd for $C_{24}H_{21}ClN_2P$: 403.1126 $[M]^+$; found: 403.1121

Synthesis of N-methyl-2, 4-bis(triphenyliminophosphorano)pyridinium iodide, 8

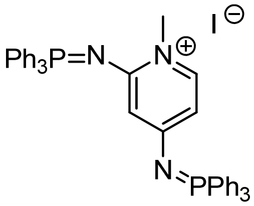

8

1,8-Diazabicyclo[5.4.0]undec-7-ene, DBU (26.00 mL, 173.51 mmol, 4.26 equiv) was added to a slurry of the amino(triphenyl)phosphonium bromide/ammonium bromide mixture (19.077 g of the mixture, corresponding to 14.975 g, 41.80 mmol, 1.03 equiv. of [Ph_3PNH_2][Br]) in acetonitrile (150 mL) to instantaneously give an pale yellow solution. Compound **7** (21.591 g, 40.68 mmol, 1 equiv.) was added and the mixture stirred for 18h at 85 °C. On cooling, the volume of the mixture was reduced and the precipitate collected by filtration, washed with cold acetonitrile (30 ml) and dried in *vacuo*. Recrystallization from acetonitrile and drying at 50 °C gave **8** (20.613 g, 66 %) as an off-white solid. mp 254-255 °C. ^{1}H NMR (300 MHz, $CDCl_3$): δ 3.88 (s, 3H, CH_3), 5.35 (d, 1H, PyrH, J = 2.4 Hz), 6.32 (dd, 1H, PyrH, J = 7.2 and 2.4 Hz), 7.29 – 7.42 (m, 24H, ArH), 7.48 – 7.56 (m, 6H, ArH), 7.70 (dt, 1H, PyrH, J = 7.2 and 2.1 Hz), ^{13}C NMR (101 MHz, $CDCl_3$): δ 40.9 (CH_3), 105.8 (dd, CH, J = 15.2, 7.4 Hz), 113.9 (d, CH, J = 24.3 Hz), 127.0 (d, C, J = 103.4 Hz), 127.7(d, C, J = 100.8 Hz), 129.2 (d, CH, J = 18.2 Hz), 129.4 (d, CH, J = 18.4 Hz), 132.1 (d, CH, J = 10.4 Hz), 132.2 (d, CH, J = 10.2 Hz), 132.8 (CH, J = 2.8 Hz), 133.2 (CH, J = 2.9 Hz), 140.4 (CH), 155.7(d, C, J = 10.6 Hz), 164.2 (C). ^{31}P NMR (121 MHz, $CDCl_3$): δ 9.8 and 12.1 ppm. **HRMS** (ESI+): m/z calcd for $C_{42}H_{36}N_3P_2$: 644.2379 $[M]^+$; found: 644.2374

Synthesis of tetrasubstituted bispyridinylidene, **1**

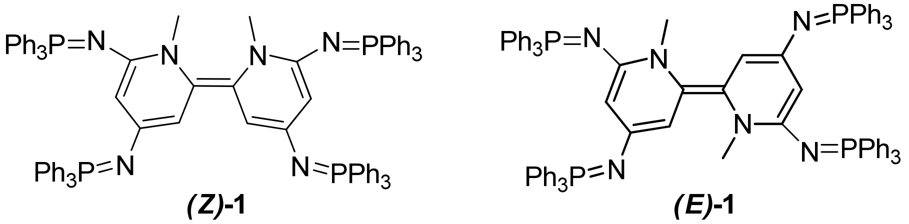

N-methyl-2,4-bis(triphenyliminophosphorano)pyridinium iodide, **8** (0.655 g, 0.85 mmol) was added to a solution of KHMDS (0.199 g, 1.00 mmol) in toluene (15 ml). The purple-colored

reaction mixture was stirred for 3h and filtered. The filtrate was discarded. The crude solid residue on filter was extracted with benzene (40 mL) and filtered into a clean Schlenk flask. Removal of solvents from the filtrate gave **1** (0.238 g, 43.5 %) as an air and moisture sensitive purple solid present as a mixture of two isomers ***(Z)*-1** and ***(E)*-1** in a ratio of 2:1. ^{1}**H NMR** (300 MHz, C_6D_6): ***(Z)*-1**: δ 3.65 (s, 6H, CH_3), 4.61 (s, 2H, PyrH), 5.80 (s, 2H, PyrH). ***(E)*-1**: δ 3.33 (s, 6H, CH_3), 4.78 (s, 2H, PyrH), 5.78 (s, 2H, PyrH). ***(Z)*-1** and ***(E)*-1**: δ 6.85 – 7.00 (m, 36H, ArH), 7.66 – 7.82 (m, 54H, ArH). ^{31}**P NMR** (121 MHz, C_6D_6): δ 0.4 and –7.8 (major, ***Z***); –1.3 and –5.4 (minor, ***E***). λ_{max} (DMF, 1.0 x 10^{-3} M) = 308 (ε = 2.9 x 10^{3} $M^{-1}cm^{-1}$), 444 (ε = 1.2 x 10^{3} $M^{-1}cm^{-1}$), 604 (ε = 5.1 x 10^{2} $M^{-1}cm^{-1}$)

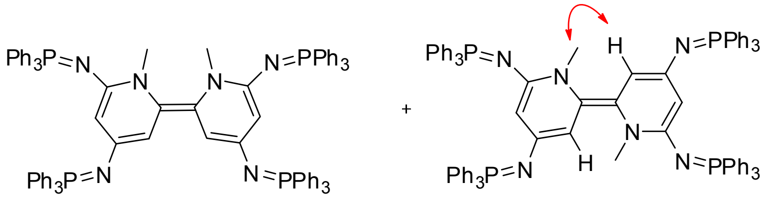

(*Z*)-**1** (major) (*E*)-**1** (minor)

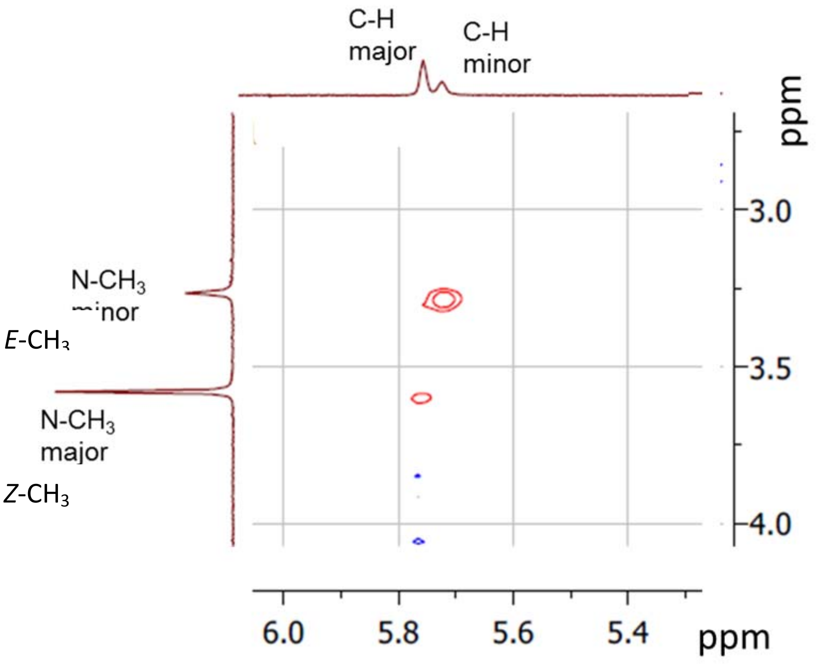

Figure S1e. ROESY spectrum showing the strong correlation between the C-H and the N-CH_3 for only the minor (*E*)-isomer of compound **1**

Synthesis of bipyridinylidinium dichloride, 1^{2+}-2Cl^{-}

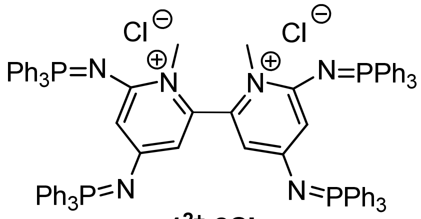

1^{2+}-2Cl^{-}

Hexachloroethane (0.036 g, 0.153 mmol) was added to a solution of **1** (0.130 g, 0.102 mmol) in benzene. The mixture was stirred for 18h. Diethyl ether (5 mL) was added and the precipitate was filtered. The solid was washed with diethyl ether (5 mL) and dried in *vacuo* at 50 °C to afford **1^{2+}-2Cl^{-}** as beige solid (0.130 g, 93.5%). mp 186-188 °C (dec). ^{1}H NMR (300 MHz, $CDCl_3$): δ 3.78 (s, 6H, CH_3), 5.36 (d, 2H, PyrH, J = 2.4 Hz), 6.60 (d, 2H, PyrH, J = 2.4 Hz), 7.24 – 7.71 (m, 60H, ArH). ^{13}C NMR (101 MHz, $CDCl_3$): δ 37.2 (CH_3), 104.9 (d, CH, J = 12.2 Hz), 117.5 (dd, CH, J = 24.1, 7.4 Hz), 126.4 (d, C, J = 103.6 Hz), 127.2 (d, C, J = 101.0 Hz), 129.5 (d, CH, J = 12.2 Hz), 129.7 (d, CH, J = 12.7 Hz), 132.2 (d, CH, J = 10.4 Hz), 132.3 (d, CH, J = 10.2 Hz), 132.8 (d, CH, J = 2.8 Hz), 133.1 (d, CH, J = 2.9 Hz), 143.1 (C), 156.6 (d, C, J = 10.6 Hz), 164.2 (C). ^{31}P NMR (121 MHz, $CDCl_3$): δ 10.7 and 13.5 ppm. **HRMS** (ESI+): m/z calcd for $C_{84}H_{70}N_6P_4$: 643.2301 $[M]^{2+}$; found: 643.2298. **Reduction Potential**: −1.70 V versus SCE.

Synthesis of bispyridinium diiodide, S7a

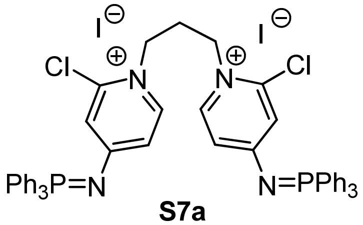

S7a

A mixture of **6** (5.025 g, 12.92 mmol) and 1,3-diiodopropane (0.70 mL, 6.10 mmol) in acetonitrile (45 mL) was heated to dissolution with a heat gun. The resulting solution was stirred at 90 °C for 96 h, cooled to room temperature and concentrated to ca 15 mL. Diethyl ether (20 mL) was added to precipitate the product which was collected by filtration and further washed with diethyl ether (20 mL). Drying under vacuum gave **S7a** was obtained as a yellow solid (5.900 g, 90%). mp 231-232 °C (dec). ^{1}H NMR (300 MHz, $CDCl_3$): δ 2.49 (quint, 2H, CH_2, J = 7.9 Hz), 4.75 (t, 4H, 2 NCH_2, J = 7.9 Hz), 6.37 (dd, 2H, PyrH, J = 7.2 and 2.7 Hz), 6.79 (d, 2H, PyrH, J = 2.7 Hz), 7.55 – 7.71 (m, 30H, ArH), 8.91 (d, 2H, PyrH, J = 7.2 Hz), ^{13}C NMR (101 MHz, $CDCl_3$): δ 30.9 (CH_2), 52.1 (NCH_2), 117.7 (d, CH, J = 17.7 Hz), 121.1 (d, CH, J = 24.0 Hz), 125.7 (d, C, J = 101.0 Hz), 129.9 (d, CH, J = 12.0 Hz), 132.6 (d, CH, J = 10.5 Hz), 133.9 (CH), 143.5 (C), 144.3 (CH), 166.5 (C). ^{31}P NMR (121 MHz, $CDCl_3$): δ 16.6 ppm. **HRMS** (ESI+): m/z calcd for $C_{49}H_{42}Cl_2N_4P_2$: 409.1126 $[M]^{2+}$; found: 409.1107

Synthesis of bispyridinium diiodide, S8a

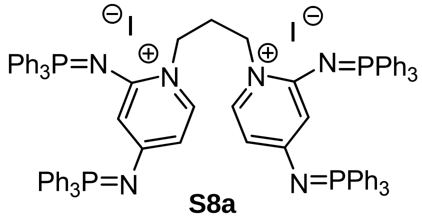

S8a

Acetonitrile (35 mL) was added to a mixture of **S7a** (1.010 g, 0.94 mmol) and Ph_3P=NH (2.317 g, 8.36 mmol). The mixture was heated to dissolution and stirred for 72h at 95 °C. The volume was reduced to 20 mL and diethyl ether (60 mL) was added to precipitate the product as a gel. Volatiles were removed to obtain a solid. Diethyl ether (50 mL) was added and the mixture was concentrated to about 5 mL. The product was collected by filtration, dried in *vacuo*, then recrystallized from isopropanol (35 mL), washed with diethyl ether (20 mL) and dried under vacuum at 120 °C to give **S8a** as pale yellow solid (0.514 g, 35.1 %). ^{1}H NMR (400 MHz, $CDCl_3$): δ 2.41 (quint, 2H, CH_2, J = 6.8 Hz), 4.72 (t, 4H, 2 NCH_2, J = 6.8 Hz), 5.36 (d, 2H, PyrH, J = 1.2 Hz), 6.23 (dd, 2H, PyrH, J = 7.2 and 2.0 Hz), 7.26-7.52 (m, 60H, ArH), 7.88 (dt, 2H, PyrH, J = 7.2, 2.0 Hz). ^{13}C NMR (101 MHz, $CDCl_3$): δ 30.2 (CH_2), 49.6 (NCH_2), 106.4 (d, CH, J = 16.3 Hz), 113.8 (d, CH, J = 22.6 Hz), 126.7 (d, C, J = 100.4 Hz), 127.7 (d, C, J = 98.4 Hz), 129.2 (d, CH, J = 12.4 Hz), 129.5 (d, CH, J = 12.7 Hz), 132.0 (d, CH, J = 10.2 Hz), 132.0 (d, CH,

J = 10.0 Hz), 132.9 (CH), 133.3 (CH), 140.1 (CH), 155.4 (d, C, J = 10.4 Hz), 164.0 (C). ^{31}P NMR (121 MHz, $CDCl_3$): δ 9.6 and 13.0 ppm

Synthesis of bipyridinylidinium dichloride, S1a^{2+}-2Cl$^-$

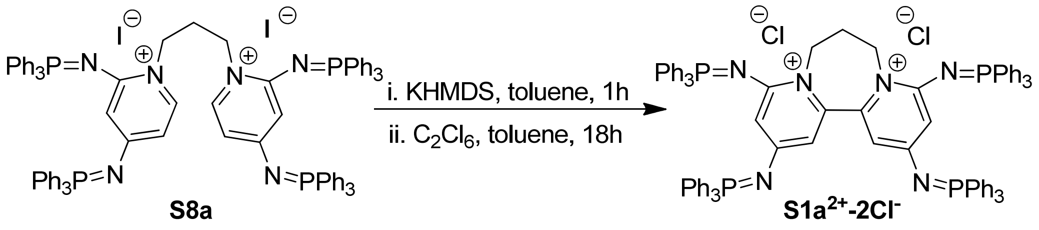

Toluene (0.5 mL) was added to a mixture of KHMDS (0.012g, 0.058 mmol) and **S8a** (0.045 g, 0.029 mmol) and stirred for 1h. Hexachloroethane (C_2Cl_6) was added and the mixture was stirred for 16h. Diethyl ether was added (10 mL). The mixture was filtered and the solid product washed with diethyl ether (5 mL) and dried under vacuum to give a purple solid, composed of the desired product **S1a^{2+}-2Cl$^-$** (0.033 g, 61 %) and some unreacted **S8a** (0.0041 g, 7%). ^{1}H NMR (300 MHz, $CDCl_3$): **S1a^{2+}-2Cl$^-$** δ 2.45-2.50 (m, 2H, CH_2), 3.73-3.83 (m, 2H, NCH_2), 5.38 (d, 2H, PyrH, J = 2.3 Hz), 5.61 (m, 2H, NCH_2), 6.63 (d, 2H, PyrH, J = 2.3 Hz), 7.11-7.58 (m, 60H, ArH). ^{31}P NMR (121 MHz, $CDCl_3$): δ 10.0 and 13.5 ppm. **Reduction Potential**: −1.72 V and −1.58 V versus SCE.

3. Experimental Procedures for Reduction Reactions

Generation of "*in situ* donor" from the salt 8 and general reduction reaction procedure

Salt **8** (2 equiv.) was added to a pressure tube. KHMDS (2 equiv.) and toluene (9 mL) were added into the tube in glove box. The resulting reaction mixture was stirred (a deep purple colour appeared within 1 minute) for 2 h at room temperature, inside the glove box, and generated "*in situ* donor" (1 equiv.).

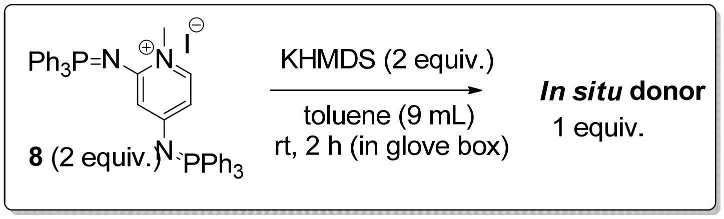

Later, substrate (in 1 mL toluene) was added into the tube and the tube was sealed properly before removing from the glove box. Reaction was then carried out for a specified time in a fume cupboard. After cooling the pressure tube to room temperature, the reaction mixture was quenched with 1N HCl (10 mL) and then extracted with diethyl ether (3 x 10 mL). The combined ether layers were washed with water (10 mL), brine solution (10 mL), dried over anhydrous sodium sulfate, filtered and concentrated using rotary evaporator. The crude products obtained were purified by column chromatography.

Reduction of sulfonamides

Acid-base work-up procedure for the reduction of sulfonamides

The reaction mixture was quenched with 1N HCl (10 mL) and extracted with diethyl ether (3 x 10 mL) and the collected ether layers were set aside. The residual aqueous phase was then basified with 2N NaOH (10 mL) and extracted with diethyl ether (3 x 10 mL). The combined ether layers were washed with water (10 mL), brine solution (10 mL) and dried over anhydrous sodium sulfate. The filtered solution was concentrated and the crude product was purified by column chromatography.

Synthesis of 4-phenyl-1-tosylpiperidine 9[7]

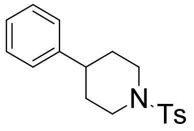

To a mixture of 4-phenylpiperidine (0.483 g, 3 mmol) and pyridine (1 mL), *p*-toluenesulfonyl chloride (0.686 g, 3.6 mmol) in dry dichloromethane (10 mL) was added slowly and stirred at room temperature for 1 h. The reaction mixture was then washed with aqueous 1N HCl (10 mL) and extracted with diethyl ether (2 x 10 mL). The combined organic phases were washed with water (10 mL), brine solution (10 mL) and dried over anhydrous sodium sulfate. The filtered solution was concentrated and was purified by column chromatography [5% ethyl acetate in petroleum ether] to yield 4-phenyl-1-tosylpiperidine **9** (0.714 g, 75%) as a white powder m.p. 150-152 ^{o}C (lit.:[7] 151-152 ^{o}C); [Found: (CI corona$^+$) $(M+H)^+$ 316.1368. $C_{18}H_{22}NO_2S$ (M+H) requires 316.1366]; ν_{max}(ATR)/cm^{-1} 3025, 2943, 2922, 2840, 1595, 1493, 1450, 1341, 1164; ^{1}H-NMR (400 MHz, $CDCl_3$) δ 1.84-1.92 (4H, m, ArCH(C*H*$_2$)$_2$), 2.34-2.45 (3H, m, ArC*H*, NC*H*$_2$), 2.48 (3H, s, ArC*H*$_3$), 3.94-3.98 (2H, m, NC*H*$_2$), 7.16 (2H, d, J = 8.8 Hz, ArH), 7.21-7.25 (1H, m, ArH), 7.28-7.34 (2H, m, ArH), 7.37 (2H, d, J = 8.4 Hz, ArH), 7.71 (2H, d, J = 8.4 Hz, ArH); ^{13}C-NMR (100 MHz, $CDCl_3$) δ 21.0 (CH_3), 32.1 (CH_2), 41.3 (CH), 46.4 (CH_2), 126.1 (CH), 126.2 (CH), 127.3 (CH), 128.1 (CH), 129.1 (CH), 132.7 (C), 143.0 (C), 144.4 (C); *m/z* (ESI$^+$) 316 [$(M+H)^+$, 100%], 247 (6).

Synthesis of 4-methyl-*N,N*-dioctylbenzenesulfonamide 11[8]

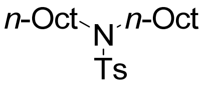

di-*N*-Octylamine (0.482 g, 2 mmol) and pyridine (1 mL) were treated with *p*-toluenesulfonyl chloride (0.46 g, 2.4 mmol) in dry DCM (5 mL) and afforded 4-methyl-*N,N*-dioctylbenzenesulfonamide **11** (0.556 g, 70%) as a colourless oil, after following the synthesis method of **9**. [Found: (ESI$^+$) $(M+H)^+$ 396.2931. $C_{23}H_{42}NO_2S$ (M+H) requires 396.2931]; ν_{max}(film)/cm^{-1} 3029, 2927, 2856, 1599, 1466, 1376, 1341, 1159; ^{1}H-NMR (400 MHz, $CDCl_3$) δ 0.88 (6H, t, J = 7.2 Hz, CH_2CH_2C*H*$_3$), 1.25-1.30 (20H, m, NCH_2CH_2C*H*$_2$C*H*$_2$C*H*$_2$C*H*$_2$C*H*$_2$$CH_3$), 1.48-1.52 (4H, m, NCH_2C*H*$_2$$CH_2$), 2.42 (3H, s, ArC*H*$_3$), 3.09 (4H, t, J = 7.6 Hz, NC*H*$_2$$CH_2CH_2$),

7.28 (2H, d, J = 8.0 Hz, ArH), 7.68 (2H, d, J = 8.0 Hz, ArH); ^{13}C-NMR (100 MHz, $CDCl_3$) δ 14.1 (CH_3), 21.4 (CH_3), 22.6 (CH_2), 26.7 (CH_2), 28.7 (CH_2), 29.2 (2 x CH_2), 31.8 (CH_2), 48.2 (CH_2), 127.1 (CH), 129.5 (CH), 137.2 (C), 142.8 (C); *m/z* (ESI^+) 808 [$(2M+NH_4)^+$, 100%], 396 [$(M+H)^+$, 85%].

Synthesis of *N*-benzyl-4-methyl-*N*-phenylbenzenesulfonamide 13[9]

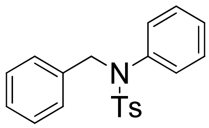

N-Benzylaniline (0.916 g, 5 mmol) and pyridine (1 mL) were treated with *p*-toluenesulfonyl chloride (1.1439 g, 6 mmol) in dry DCM (10 mL) and afforded *N*-benzyl-4-methyl-*N*-phenylbenzenesulfonamide **13** (1.616 g, 96%) as a white solid, after following the synthesis method of **9**. m.p. 138-140 °C (lit.:[9] 139-140 °C); [Found: (ESI^+) $(M+H)^+$ 338.1204. $C_{20}H_{20}NO_2S$ (M+H) requires 338.1209]; ν_{max}(film)/cm^{-1} 3027, 2919, 1597, 1492, 1347, 1153, 1095; ^{1}H-NMR (400 MHz, $CDCl_3$) δ 2.46 (3H, s, ArC*H₃*), 4.73 (2H, s, ArC*H₂*NAr), 6.97-7.00 (2H, m, ArH), 7.20-7.23 (8H, m, ArH), 7.27-7.30 (2H, m, ArH), 7.54-7.56 (2H, m, ArH); ^{13}C-NMR (100 MHz, $CDCl_3$) δ 21.6 (CH_3), 54.7 (CH_2), 127.6 (CH), 127.8 (CH), 128.4 (CH), 128.5 (CH), 128.8 (CH), 129.0 (CH), 129.1 (CH), 129.5 (CH), 135.7 (C), 136.0 (C), 139.0 (C), 143.5 (C); *m/z* (ESI^+) 355 [$(M+NH_4)^+$, 55%], 338 [$(M+H)^+$, 100%], 183 (3).

Synthesis of 1-tosyl-1H-indole 15[10]

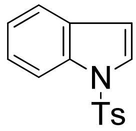

Indole (0.234 g, 2 mmol) and pyridine (1 mL) were treated with *p*-toluenesulfonyl chloride (0.46 g, 2.4 mmol) in dry DCM (5 mL) and afforded 1-tosyl-1H-indole **15** (0.522 g, 91%) as a white solid, after following the synthesis method of **9**. m.p. 82-84 °C (lit.:[10] 83-84 °C); **[**Found: (ESI^+) $(M+H)^+$ 272.0739. $C_{15}H_{14}NO_2S$ (M+H) requires 272.0740]; ν_{max}(KBr)/cm^{-1} 3116, 2919, 1596, 1446, 1369, 1260, 1168, 1128; ^{1}H-NMR (400 MHz, $CDCl_3$) δ 2.34 (3H, s, C*H₃*), 6.67 (1H, dd, J = 3.6, 0.8 Hz, ArH), 7.21-7.26 (3H, m, ArH), 7.30-7.35 (1H, m, ArH), 7.53-7.59 (2H, m, ArH), 7.76-7.79 (2H, m, ArH), 8.00-8.02 (1H, m, ArH); ^{13}C-NMR (100 MHz, $CDCl_3$) δ 21.6 (CH_3), 109.0 (CH), 113.6 (CH), 121.4 (CH), 123.3 (CH), 124.6 (CH), 126.3 (CH), 126.8 (CH), 129.9

(CH), 130.7 (C), 134.8 (C), 135.3 (C), 144.9 (C); *m/z* (ESI^+) 289 [$(M+NH_4)^+$, 29%], 272 [$(M+H)^+$, 100%].

Synthesis of *N,N*-dioctylmethanesulfonamide 11b[11]

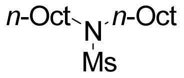

di-*N*-Octylamine (0.482 g, 2 mmol) and pyridine (1 mL) were treated with methanesulfonyl chloride (0.275 g, 2.4 mmol) in dry DCM (5mL) and afforded *N*,*N*-dioctylmethanesulfonamide **11b** (0.536 g, 84%) as a pale yellow oil, after following the synthesis method of **9**. ν_{max} (ATR)/cm^{-1} 2924, 2854, 1465, 1332, 1143, 958, 783, 736; ^{1}H-NMR (400 MHz, $CDCl_3$) δ 0.89 (6H, t, J = 7.2 Hz, CH_2CH_3), 1.28-1.30 (20H, m, $NCH_2CH_2(CH_2)_5CH_3$), 1.57-1.62 (4H, m, $NCH_2CH_2CH_2$), 2.82 (3H, s, SO_2CH_3), 3.15 (4H, t, J = 8.0 Hz, NCH_2CH_2); ^{13}C-NMR (100 MHz, $CDCl_3$) δ 14.2 (CH_3), 22.8 (CH_2), 26.9 (CH_2), 28.9 (CH_2), 29.35 (CH_2), 29.37 (CH_2), 31.9 (CH_2), 38.4 (CH_3), 47.9 (CH_2).

The spectral data were consistent with the literature data of the same compound.[11]

Reduction of 4-phenyl-1-tosylpiperidine 9 using "*in situ* donor"

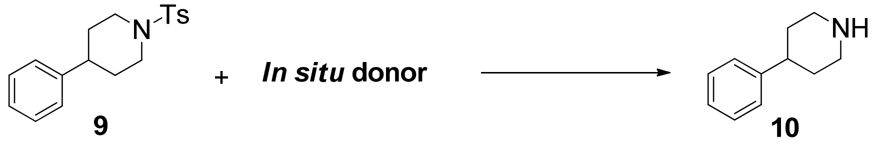

1.5 equiv.	(i) rt, 16 h	0%
1.5 equiv.	(ii) 100 °C, 16 h	42%
4 equiv.	(iii) 110 °C, 24 h	75%

(i) The general reduction reaction procedure was applied to 4-phenyl-1-tosylpiperidine **9** (0.032 g, 0.1 mmol) using the salt **8** (0.231 g, 0.3 mmol) for 16 h at room temperature. After following the general acid-base work-up procedure for the reduction of sulfonamides, the reaction did not provide any product **10**.

(ii) The general reduction reaction procedure was applied to 4-phenyl-1-tosylpiperidine **9** (0.032 g, 0.1 mmol) using the salt **8** (0.231 g, 0.3 mmol) for 16 h at 100 °C. After following

the general acid-base work-up procedure for the reduction of sulfonamides, the reaction provided reduced product **10** (0.0067 g, 42%).

(iii) The general reduction reaction procedure was applied to 4-phenyl-1-tosylpiperidine **9** (0.032 g, 0.1 mmol) using the salt **8** (0.617 g, 0.8 mmol) for 24 h at 110 ^{o}C. After following the general acid-base work-up procedure for the reduction of sulfonamides, the reaction provided reduced product 4-phenylpiperidine **10**[12] (0.012 g, 75%) as a white solid. m.p. 62-64 ^{o}C (lit.:[12] 62-64 ^{o}C); [Found: (CI corona^{+}) $(M+H)^{+}$ 162.1277. $C_{11}H_{16}N$ (M+H) requires 162.1277]; ν_{max}(ATR)/cm^{-1} 3293, 2931, 2917, 2848, 1644, 1543, 1451, 1413, 1370, 1247; ^{1}H-NMR (400 MHz, $CDCl_3$) δ 1.69-1.73 (2H, m, ArCHC*H*$_2$), 1.80 (1H, bs, N*H*), 1.84-1.88 (2H, m, ArCHC*H*$_2$), 2.61-2.67 (1H, m, ArC*H*), 2.74-2.81 (2H, m, NHC*H*$_2$), 3.20-3.23 (2H, m, NHC*H*$_2$), 7.20-7.35 (5H, m, ArH); ^{13}C-NMR (100 MHz, $CDCl_3$) δ 34.1 (CH_2), 42.6 (CH), 46.7 (CH_2), 125.6 (CH), 126.3 (CH), 127.9 (CH), 146.3 (C). The spectral data were consistent with the literature data of the same compound.[12]

Reduction of 4-methyl-*N*,*N*-dioctylbenzenesulfonamide 11 using "*in situ* donor"

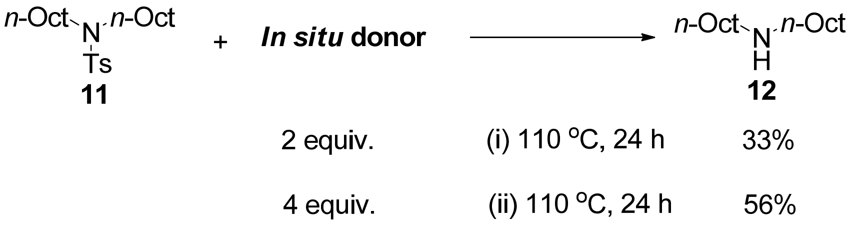

(i) The general reduction reaction procedure was applied to 4-methyl-*N*,*N*-dioctylbenzenesulfonamide **11** (0.0395 g, 0.1 mmol) using the salt **8** (0.309 g, 0.4 mmol) for 24 h at 110 ^{o}C. After following the general acid-base work-up procedure for the reduction of sulfonamides, the reaction provided reduced product **12** (0.008 g, 33%).

(ii) The general reduction reaction procedure was applied to 4-methyl-*N*,*N*-dioctylbenzenesulfonamide **11** (0.0395 g, 0.1 mmol) using the salt **8** (0.617 g, 0.8 mmol) for 24 h at 110 ^{o}C. After following the general acid-base work-up procedure for the reduction of sulfonamides, the reaction provided reduced product **12**[11] (0.0136 g, 56%) as a colourless oil. [Found: (ESI^{+}) $(M+H)^{+}$ 242.2841. $C_{16}H_{36}N$ (M+H) requires 242.2842]; ν_{max}(film)/cm^{-1} 3274, 3116, 2955, 2924, 2853, 1468, 720; ^{1}H-NMR (400 MHz, $CDCl_3$) δ 0.87 (6H, t, J = 7.6 Hz,

$N(CH_2)_7CH_3$), 1.05 (1H, bs, NH), 1.26-133 (20H, m, $N(CH_2)_2(CH_2)_5CH_3$), 1.44-1.49 (4H, m, $NCH_2CH_2(CH_2)_5CH_3$), 2.58 (4H, t, J = 7.2 Hz, $NCH_2(CH_2)_7CH_3$); ^{13}C-NMR (100 MHz, $CDCl_3$) δ 14.1 (CH_3), 22.7 (CH_2), 27.4 (CH_2), 29.3 (CH_2), 29.6 (CH_2), 30.2 (CH_2), 31.8 (CH_2), 50.2 (CH_2); *m/z* (ESI$^+$) 242 [(M+H)$^+$, 100%].

Reduction of *N*-benzyl-*N*-phenyl-4-methylbenzenesulfonamide 13 using "*in situ* donor"

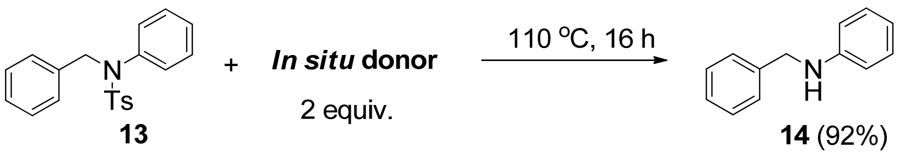

The general reduction reaction procedure was applied to *N*-benzyl-*N*-phenyl-4-methylbenzenesulfonamide **13** (0.0337 g, 0.1 mmol) using the salt **8** (0.308 g, 0.4 mmol) for 16 h at 110 °C. After following the general acid-base work-up procedure for the reduction of sulfonamides, the reaction provided reduced product *N*-benzylaniline **14**[13] (0.0168 g, 92%) as a white solid m.p. 34-35 °C (lit.:[13] 33-34 °C); [Found: (ESI$^+$) (M+H)$^+$ 184.1122. $C_{13}H_{14}N$ (M+H) requires 184.1121]; ν_{max}(KBr) /cm^{-1} 3417, 3022, 2924, 2846, 1603, 1512, 1329, 736; ^{1}H-NMR (400 MHz, $CDCl_3$) δ 4.25 (1H, bs, NH), 4.35 (2H, s, $ArCH_2N$), 6.66-6.68 (2H, m, ArH), 6.72-6.76 (1H, m, ArH), 7.17-7.21 (2H, m, ArH), 7.27-7.31 (1H, m, ArH), 7.34-7.41 (4H, m, ArH); ^{13}C-NMR (100 MHz, $CDCl_3$) δ 48.4 (CH_2), 112.9 (CH), 117.6 (CH), 127.3 (CH), 127.6 (CH), 128.7 (CH), 129.3 (CH), 139.5 (C), 148.2 (C); *m/z* (ESI$^+$) 184 [(M+H)$^+$, 100%].

Reduction of 1-tosyl-1H-indole 15 using "*in situ* donor"

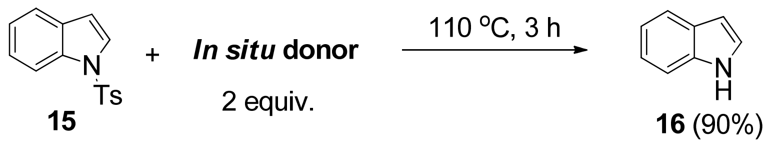

The general reduction reaction procedure was applied to 1-tosyl-1H-indole **15** (0.0271 g, 0.1 mmol) using the salt **8** (0.308 g, 0.4 mmol) for 3 h at 110 °C. After following the general acid-base work-up procedure for the reduction of sulfonamides, the reaction provided reduced product indole **16**[14] (0.0105 g, 90%) as a white solid m.p. 51-53 °C; (lit.:[14] 51-53 °C); ν_{max}(KBr) /cm^{-1} 3400, 3098, 3049, 1576, 1506, 1456, 1247, 745; ^{1}H-NMR (400 MHz, $CDCl_3$) δ 6.59-6.60 (1H,

m, ArH), 7.14-7.18 (1H, m, ArH), 7.21-7.27 (2H, m, ArH), 7.42 (1H, dd, J = 8.0, 0.4 Hz ArH), 7.92 (1H, d, J = 8.0 Hz, ArH), 8.11 (1H, bs, NH); ^{13}C-NMR (100 MHz, $CDCl_3$) δ 102.6 (CH), 111.0 (CH), 119.8 (CH), 120.8 (CH), 122.0 (CH), 124.1 (CH), 127.9 (C), 135.8 (C). The spectral data were consistent with the literature data.[14]

Reduction of *N,N*-dioctylmethanesulfonamide 11b using "*in situ* donor"

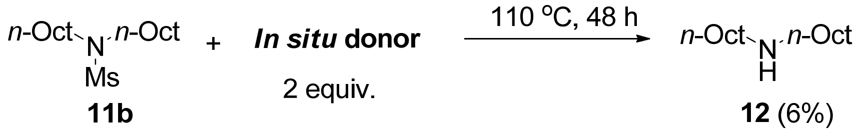

The general reduction reaction procedure was applied to *N,N*-dioctylmethanesulfonamide **11b** (0.0319 g, 0.1 mmol) using the salt **8** (0.617 g, 0.8 mmol) for 48 h at 110 °C. After following the general acid-base work-up procedure for the reduction of sulfonamides, the reaction provided reduced product **12** (0.0015 g, 6%) as a colourless oil. The spectral data of the product were consistent with the previously reported data of the same compound.

Reduction of aryl halides

Synthesis of 1-iodo-4-(3-phenylpropoxy)benzene 17a[15]

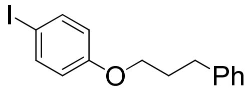

Potassium carbonate (0.414 g, 3 mmol) was added to a solution of 4-iodophenol (0.495 g, 2.25 mmol) and 1-iodo-3-phenylpropane (0.369 g, 1.5 mmol) in DMF (10 ml). The resulting suspension was stirred at room temperature for 16 h. At this point, the reaction was quenched with water (10 mL) and extracted with diethyl ether (3 x 15 mL). The combined organic phases were washed again with water (10 mL), brine solution (10 mL) and dried over anhydrous sodium sulfate, filtered and concentrated using rotary evaporator. The crude product was then purified by column chromatography (5% diethyl ether in hexane) to afford 1-iodo-4-(3-phenylpropoxy)benzene **17a** (0.426 g, 84%).

^{1}H-NMR (400 MHz, $CDCl_3$) δ 2.07-2.14 (2H, m, OCH_2CH_2), 2.81 (2H, t, J = 8.0 Hz, $ArCH_2$), 3.93 (2H, t, J = 6.4 Hz, OCH_2), 6.66-6.69 (2H, m, ArH), 7.19-7.23 (3H, m, ArH), 7.27-7.32 (2H,

m, ArH), 7.53-7.57 (2H, m, ArH); ^{13}C-NMR (100 MHz, $CDCl_3$) δ 30.8 (CH_2), 32.2 (CH_2), 67.1 (CH_2), 82.7 (C), 117.1 (CH), 126.1 (CH), 128.6 (CH), 128.64 (CH), 138.3 (CH), 141.5 (C), 159.0 (C). The spectral data were consistent with the literature data.[15]

Synthesis of 1-bromo-4-(3-phenylpropoxy)benzene 17b[16]

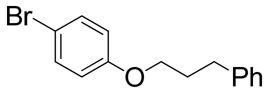

4-Bromophenol (0.390 g, 2.25 mmol) and 1-iodo-3-phenylpropane (0.369 g, 1.5 mmol) were treated with potassium carbonate (0.414 g, 3 mmol) and afforded 1-bromo-4-(3-phenylpropoxy)benzene **17b** (0.351 g, 81%), after following the synthesis method of **17a**.

^{1}H-NMR (400 MHz, $CDCl_3$) δ 2.07-2.14 (2H, m, OCH_2CH_2), 2.81 (2H, t, J = 7.6 Hz, $ArCH_2$), 3.94 (2H, t, J = 6.4 Hz, OCH_2), 6.76-6.80 (2H, m, ArH), 7.19-7.23 (3H, m, ArH), 7.28-7.32 (2H, m, ArH), 7.35-7.39 (2H, m, ArH); ^{13}C-NMR (100 MHz, $CDCl_3$) δ 30.8 (CH_2), 32.2 (CH_2), 67.2 (CH_2), 112.8 (C), 116.5 (CH), 126.1 (C), 128.6 (CH), 128.64 (CH), 132.4 (CH), 141.5 (C), 158.3 (C). The spectral data were consistent with the literature data.[16]

Synthesis of 1-chloro-4-(3-phenylpropoxy)benzene 17c[17]

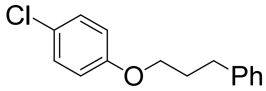

4-Chlorophenol (0.289 g, 2.25 mmol) and 1-iodo-3-phenylpropane (0.369 g, 1.5 mmol) were treated with potassium carbonate (0.414 g, 3 mmol) and afforded 1-chloro-4-(3-phenylpropoxy)benzene **17c** (0.302 g, 82%), after following the synthesis method of **17a**.

^{1}H-NMR (400 MHz, $CDCl_3$) δ 2.07-2.14 (2H, m, OCH_2CH_2), 2.81 (2H, t, J = 8.0 Hz, $ArCH_2$), 3.94 (2H, t, J = 6.4 Hz, OCH_2), 6.80-6.84 (2H, m, ArH), 7.19-7.25 (5H, m, ArH), 7.28-7.33 (2H, m, ArH); ^{13}C-NMR (100 MHz, $CDCl_3$) δ 30.8 (CH_2), 32.2 (CH_2), 67.3 (CH_2), 115.9 (CH), 125.6 (C), 126.1 (CH), 128.6 (CH), 128.7 (CH), 129.4 (CH), 141.5 (C), 157.8 (C). The spectral data were consistent with the literature data.[17]

Synthesis of 1-(allyloxy)-2-iodobenzene 19a[18]

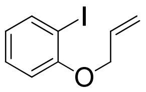

2-Iodophenol (1.21 g, 5.5 mmol) and allyl bromide (0.605 g, 5 mmol) were treated with potassium carbonate (1.036 g, 7.5 mmol) and afforded 1-(allyloxy)-2-iodobenzene **19a** (1.031 g, 79%), after following the synthesis method of **17a**.

^{1}H-NMR (400 MHz, $CDCl_3$) δ 4.61 (2H, dt, J = 4.8, 1.6 Hz, ArOCH_2), 5.33 (1H, dq, J = 10.8, 1.6 Hz, CH=CH_2), 5.54 (1H, dq, J = 17.2, 1.6 Hz, CH=CH_2), 6.03-6.12 (1H, m, CH=CH_2), 6.71-6.75 (1H, m, ArH), 6.82 (1H, dd, J = 8.4, 1.2 Hz, ArH), 7.27-7.31 (1H, m, ArH), 7.80 (1H, dd, J = 8.0, 1.2 Hz, ArH); ^{13}C-NMR (100 MHz, $CDCl_3$) δ 69.8 (CH_2), 86.8 (C), 112.6 (CH), 117.7 (CH_2), 122.8 (CH), 129.5 (CH), 132.7 (CH), 139.6 (CH), 157.2 (C). The spectral data were consistent with the literature data.[18]

Synthesis of 1-(allyloxy)-2-bromobenzene 19b[19]

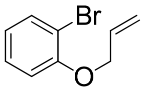

2-bromophenol (0.951 g, 5.5 mmol) and allyl bromide (0.605 g, 5 mmol) were treated with potassium carbonate (1.036 g, 7.5 mmol) and afforded 1-(allyloxy)-2-bromobenzene **19b** (0.855 g, 80%), after following the synthesis method of **17a**.

^{1}H-NMR (400 MHz, $CDCl_3$) δ 4.63 (2H, dt, J = 5.2, 1.6 Hz, ArOCH_2), 5.32 (1H, dq, J = 10.8, 1.6 Hz, CH=CH_2), 5.49 (1H, dq, J = 17.2, 1.6 Hz, CH=CH_2), 6.03-6.13 (1H, m, CH=CH_2), 6.83-6.87 (1H, m, ArH), 6.91 (1H, dd, J = 8.4, 1.2 Hz, ArH), 7.23-7.27 (1H, m, ArH), 7.55 (1H, dd, J = 8.0, 1.2 Hz, ArH); ^{13}C-NMR (100 MHz, $CDCl_3$) δ 69.8 (CH_2), 112.5 (C), 113.8 (CH), 117.9 (CH_2), 122.1 (CH), 128.5 (CH), 132.8 (CH), 133.6 (CH), 155.1 (C). The spectral data were consistent with the literature data.[19]

Reduction of 1-iodo-4-(3-phenylpropoxy)benzene 17a using "*in situ* donor"

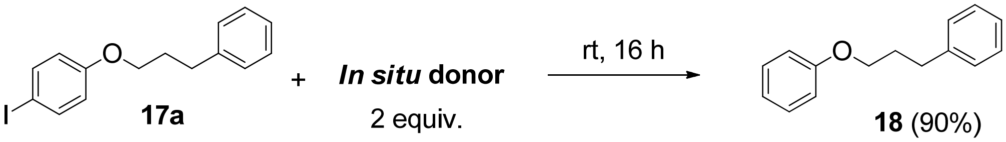

The general reduction reaction procedure was applied to 1-iodo-4-(3-phenylpropoxy)benzene **17a** (0.0338 g, 0.1 mmol) using the salt **8** (0.308 g, 0.4 mmol) for 16 h at room temperature. After following the general work-up procedure, the reaction provided reduced product (3-phenoxypropyl)benzene **18**[15] (0.019 g, 90%) as a colourless oil.

^{1}H-NMR (400 MHz, $CDCl_3$) δ 2.09-2.16 (2H, m, OCH_2CH_2), 2.83 (2H, t, J = 7.6 Hz, $ArCH_2$), 3.99 (2H, t, J = 6.4 Hz, OCH_2), 6.90-6.97 (3H, m, ArH), 7.19-7.24 (3H, m, ArH), 7.27-7.33 (4H, m, ArH); ^{13}C-NMR (100 MHz, $CDCl_3$) δ 31.0 (CH_2), 32.3 (CH_2), 66.9 (CH_2), 114.7 (CH), 120.7 (CH), 126.1 (CH), 128.6 (CH), 128.7 (CH), 129.6 (CH), 141.7 (C), 159.2 (C). The spectral data were consistent with the literature data.[15]

Reaction of 1-iodo-4-(3-phenylpropoxy)benzene 17a using KHMDS only

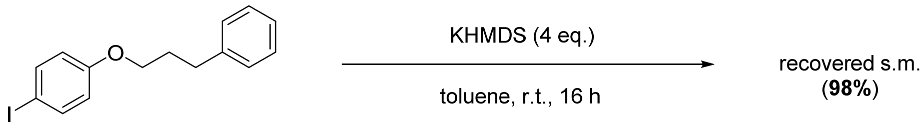

1-iodo-4-(3-phenylpropoxy)benzene 17a (0.0338 g, 0.100 mmol, 1 eq.) was added, as a solid, to an oven-dried pressure tube inside a glovebox. KHMDS (0.0798 g, 0.400 mmol, 4 eq.) was added to the tube followed by toluene (10 mL). The pressure tube was sealed, removed from the glovebox and stirred at room temperature for 16 h. Water (10 mL) was added and the mixture extracted with Et_2O (3 x 15 mL). The combined organic phases were dried over anhydrous sodium sulfate, filtered, and concentrated to give recovered 17a (0.0330 g, 98%). The spectral data of the isolated material were consistent with the previously reported data for **17a**.

Reduction of 1-bromo-4-(3-phenylpropoxy)benzene 17b using "*in situ* donor"

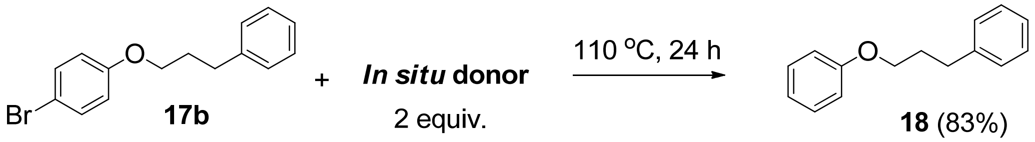

The general reduction reaction procedure was applied to 1-bromo-4-(3-phenylpropoxy)benzene **17b** (0.0291 g, 0.1 mmol) using the salt **8** (0.308 g, 0.4 mmol) for 24 h at 110 °C. After following the general work-up procedure, the reaction provided reduced product (3-phenoxypropyl)benzene **18** (0.0176 g, 83%) as a colourless oil. The spectral data of the product were consistent with the previously reported data of the same compound.

Reduction of 1-chloro-4-(3-phenylpropoxy)benzene 17c using "*in situ* donor"

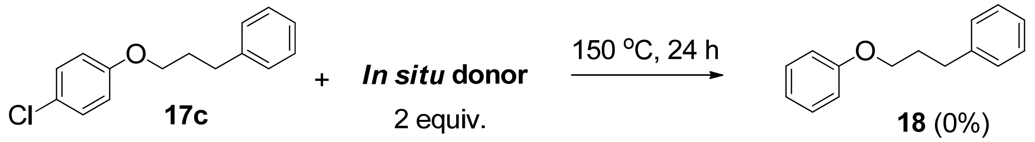

The general reduction reaction procedure was applied to 1-chloro-4-(3-phenylpropoxy)benzene **17c** (0.025 g, 0.1 mmol) using the salt **8** (0.308 g, 0.4 mmol) for 24 h at 150 °C. After following the general work-up procedure, the reaction provided recovery of starting material only.

Reduction of 1-(allyloxy)-2-iodobenzene 19a using "*in situ* donor"

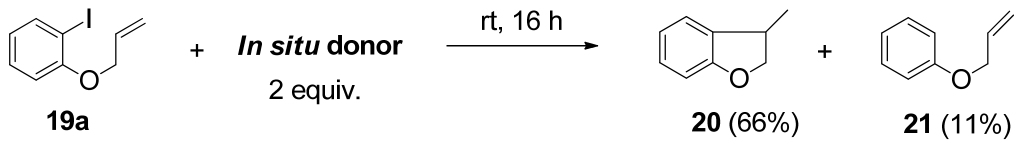

The general reduction reaction procedure was applied to 1-(allyloxy)-2-iodobenzene **19a** (0.052 g, 0.2 mmol) using the salt **8** (0.617 g, 0.8 mmol) for 16 h at room temperature. After following the general work-up procedure, the reaction provided both cyclised product 3-methyl-2,3-dihydrobenzofuran **20**[20] (0.0176 g, 66%) and (allyloxy)benzene **21**[21] (0.003 g, 11%) as colourless oils.

For **20**: ^{1}H-NMR (400 MHz, $CDCl_3$) δ 1.34 (3H, d, J = 6.8 Hz, ArCHCH_3), 3.51-3.60 (1H, m, ArCHCH$_3$), 4.08 (1H, dd, J = 8.8, 7.6 Hz, ArOCH_2), 4.69 (1H, t, J = 8.8 Hz, ArOCH_2), 6.80 (1H, d, J = 8.0 Hz, ArH), 6.88 (1H, td, J = 7.2, 0.8 Hz, ArH), 7.10-7.17 (2H, m, ArH); ^{13}C-NMR (100 MHz, $CDCl_3$) δ 19.4 (CH_3), 36.6 (CH), 78.6 (CH_2), 109.6 (CH), 120.6 (CH), 123.9 (CH), 128.1 (CH), 132.4 (C), 159.9 (C). The spectral data were consistent with the literature data.[20]

For **21**: ^{1}H-NMR (400 MHz, $CDCl_3$) δ 4.56 (2H, dt, J = 5.2, 1.6 Hz, ArOCH_2), 5.30 (1H, dq, J = 10.4, 1.2 Hz, CH=CH_2), 5.43 (1H, dq, J = 17.2, 1.6 Hz, CH=CH_2), 6.03-6.13 (1H, m, CH=CH$_2$), 6.92-6.98 (3H, m, ArH), 7.27-7.32 (2H, m, ArH); ^{13}C-NMR (100 MHz, $CDCl_3$) δ 68.9 (CH_2), 114.9 (2 x CH), 117.7 (CH_2), 121.0 (CH), 129.6 (2 x CH), 133.5 (CH), 158.8 (C). The spectral data were consistent with the literature data.[21]

Reduction of 1-(allyloxy)-2-bromobenzene 19b using "*in situ* donor"

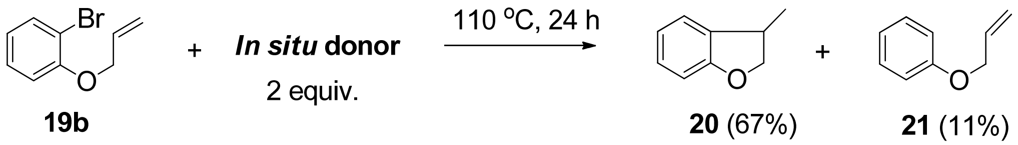

The general reduction reaction procedure was applied to 1-(allyloxy)-2-bromobenzene **19b** (0.0426 g, 0.2 mmol) using the salt **8** (0.617 g, 0.8 mmol) for 24 h at 110 °C. After following the general work-up procedure, the reaction provided both cyclised product 3-methyl-2,3-dihydrobenzofuran **20** (0.018 g, 67%) and (allyloxy)benzene **21** (0.003 g, 11%) as colourless oils. The spectral data of the products were consistent with the previously reported data of the same compounds.

Reduction of 1-chloroanthracene 22 using "*in situ* donor"

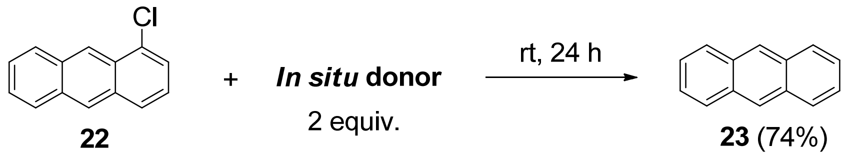

The general reduction reaction procedure was applied to 1-chloroanthracene **22** (0.0212 g, 0.1 mmol) using the salt **8** (0.308 g, 0.4 mmol) for 24 h at room temperature. After following the general work-up procedure, the reaction provided anthracene **23** (0.0132 g, 74%) as a light brown solid.^{1}H-NMR (400 MHz, $CDCl_3$) δ 7.47-7.51 (4H, m, ArH), 8.01-8.05 (4H, m, ArH),

8.45 (2H, s, ArH); ^{13}C-NMR (100 MHz, $CDCl_3$) δ 125.5 (CH), 126.4 (CH), 128.3 (CH), 131.8 (C). The spectral data were consistent with the literature data.[22]

Reduction of malononitriles

Synthesis of 2,2-bis(3-phenylpropyl)malononitrile 24[23]

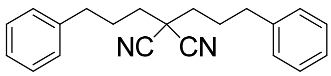

A solution of malononitrile (0.330 g, 5 mmol) in dry tetrahydrofuran (2 mL) was added slowly to suspension of sodium hydride (~60%, 0.480 g, 12 mmol) in dry tetrahydrofuran (10 mL) at 0 ^{o}C under argon gas. The resulting solution was stirred for 15 min at room temperature and (3-bromopropyl)benzene (2.388 g, 12 mmol) in dry tetrahydrofuran (2 mL) was added slowly into the reaction flask. The reaction mixture was further stirred at reflux conditions for 24 h under argon gas. At this point, the reaction was quenched with water (15 mL) and extracted with diethyl ether (3 x 15 mL). The combined organic phases were washed once again with water (10 mL), brine solution (10 mL) and dried over anhydrous sodium sulfate. The concentrated solution was purified by column chromatography (10% diethyl ether in hexane) to yield 2,2-bis(3-phenylpropyl)malononitrile **24** (1.224 g, 81%) as a white crystalline solid m.p. 78-80 ^{o}C. [Found: (CI corona$^+$) $(M+H)^+$ 303.1853. $C_{21}H_{23}N_2$ (M+H) requires 303.1856]; ν_{max}(film)/cm^{-1} 3026, 2926, 2860, 2245, 1600, 1496, 1452, 702; ^{1}H-NMR (500 MHz, $CDCl_3$) δ 1.88-1.91 (4H, m, $CCH_2CH_2CH_2Ar$), 1.97-2.03 (4H, m, $CCH_2CH_2CH_2Ar$), 2.72 (4H, t, J = 7.0 Hz, $ArCH_2$), 7.17 (4H, d, J = 7.0 Hz, ArH), 7.23 (2H, t, J = 7.5 Hz, ArH), 7.31 (4H, t, J = 7.5 Hz, ArH); ^{13}C-NMR (100 MHz, $CDCl_3$) δ 27.1 (CH_2), 34.9 (CH_2), 37.2 (CH_2), 37.7 (C), 115.7 (C), 126.6 (CH), 128.5 (CH), 128.8 (CH), 140.2 (C); *m/z* (CI corona$^+$) 303 [$(M+H)^+$, 100%], 191 (4).

Synthesis of 2,2-di(pent-4-en-1-yl)malononitrile 26[24]

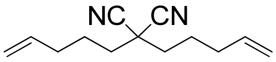

A solution of malononitrile (0.330 g, 5 mmol) in dry tetrahydrofuran (2 mL) was added slowly to suspension of sodium hydride (~60%, 0.440 g, 11 mmol) in dry tetrahydrofuran (10 mL) at 0 ^{o}C under argon gas. The resulting solution was stirred for 15 min at room temperature and 5-

bromopent-1-ene (1.6392 g, 11 mmol) in dry tetrahydrofuran (2 mL) was added slowly into the reaction flask. The reaction mixture was further stirred at reflux conditions for 24 h under argon gas. At this point, the reaction was quenched with water (15 mL) and extracted with diethyl ether (3 x 15 mL). The combined organic phases were washed once again with water (10 mL), brine solution (10 mL) and dried over anhydrous sodium sulfate. The concentrated solution was purified by column chromatography (5% diethyl ether in hexane) to yield 2,2-di(pent-4-en-1-yl)malononitrile **26** (0.726 g, 72%) as a colourless oil. [Found: (CI corona$^+$) $(M+H)^+$ 203.1542. $C_{13}H_{19}N_2$ (M+H) requires 203.1543]; ν_{max}(film)/cm^{-1} 3080, 2933, 2866, 2241, 1641, 1460, 914; ^{1}H-NMR (400 MHz, $CDCl_3$) δ 1.75-1.83 (4H, m, =$CHCH_2C\mathit{H_2}CH_2$), 1.91-1.96 (4H, m, $C\mathit{CH_2}CH_2CH_2$), 2.16-2.21 (4H, m, =$CHC\mathit{H_2}CH_2CH_2$), 5.04-5.11 (4H, m, $C\mathit{H_2}$=$CHCH_2$), 5.73-5.83 (2H, m, CH_2=$C\mathit{H}CH_2$); ^{13}C-NMR (100 MHz, $CDCl_3$) δ 24.7 (CH_2), 32.7 (CH_2), 37.2 (CH_2), 37.7 (C), 115.7 (C), 116.4 (CH_2), 136.7 (CH); *m/z* (CI corona$^+$) 203 [$(M+H)^+$, 100%], 178 (16).

Synthesis of 2,2-*bis*(3-methylbut-2-en-1-yl)malononitrile 28[23]

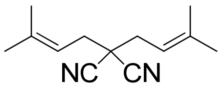

A solution of malononitrile (0.397 g, 6 mmol) in dry tetrahydrofuran (2 mL) was added slowly to suspension of sodium hydride (~60%, 0.528 g, 13.2 mmol) in dry tetrahydrofuran (10 mL) at 0 ^{o}C under argon gas. The resulting solution was stirred for 15 min at room temperature and 1-bromo-3-methylbut-2-ene (1.967 g, 13.2 mmol) in dry tetrahydrofuran (2 mL) was added slowly into the reaction flask. The reaction mixture was further stirred at reflux conditions for 16 h under argon gas. At this point, the reaction was quenched with water (15 mL) and extracted with diethyl ether (3 x 15 mL). The combined organic phases were washed once again with water (10 mL), brine solution (10 mL) and dried over anhydrous sodium sulfate. The concentrated solution was purified by column chromatography (5% ethyl acetate in petroleum ether) to yield 2,2-*bis*(3-methylbut-2-en-1-yl)malononitrile **28** (1.047 g, 86%) as a white solid m.p. 62-64 ^{o}C. ^{1}H-NMR (400 MHz, $CDCl_3$) δ 1.73 [6H, s, =$(CH_3)(C\mathit{H_3})$], 1.83 [6H, s, =$(C\mathit{H_3})(CH_3)$], 2.66 (4H, d, J = 7.6 Hz, =$CHC\mathit{H_2}$), 5.27-5.32 (2H, m, =$C\mathit{H}CH_2$); ^{13}C-NMR (100 MHz, $CDCl_3$) δ 18.4 (CH_3), 26.1 (CH_3), 35.7 (CH_2), 38.2 (C), 114.9 (CH), 115.9 (C), 140.5 (C).

Synthesis of 2,2-dimethylhexadecanenitrile 30[23]

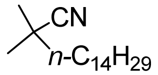

Lithium diisopropylamide (1.5 M in THF, 8 mL, 12 mmol) was added dropwise into a flask containing isobutyronitrile (0.691 g, 10 mmol) in dry THF (20 mL) at -78 °C under argon gas. The reaction contents were stirred for 1 h before adding 1-bromotetradecane dropwise into the flask at -78 °C. Later, the reaction contents were allowed to warm to room temperature and stirred for 16 h under argon gas. At this point, the reaction was quenched with sat. NH_4Cl (15 mL) and extracted with diethyl ether (3 x 15 mL). The combined ether phases were washed once again with water (10 mL), brine solution (10 mL) and dried over anhydrous sodium sulfate. The concentrated solution was purified by column chromatography (5% diethyl ether in petroleum ether) to yield 2,2-dimethylhexadecanenitrile **30** (2.005 g, 76%) as a white solid m.p. 30-32 °C. ^{1}H-NMR (400 MHz, $CDCl_3$) δ 0.89 (3H, t, *J* = 6.8 Hz, $C_{13}H_{26}CH_3$), 1.27-1.31 (22H, m, $CH_2CH_2(CH_2)_{11}CH_3$), 1.34 (6H, s, $C(CH_3)_2$), 1.44-1.55 (4H, m, $CH_2CH_2(CH_2)_{11}CH_3$); ^{13}C-NMR (100 MHz, $CDCl_3$) δ 14.2 (CH_3), 22.8 (CH_2), 25.4 (CH_2), 26.8 (2 x CH_2), 29.5 (CH_2), 29.6 (CH_2), 29.7 (CH_2), 29.77 (5 x CH_2), 29.81 (2 x CH_2), 32.1 (CH_2), 32.5 (C), 115.7 (C); *m/z* (CI corona$^+$) 203 [$(M+H)^+$, 100%], 178 (16).

Reduction of 2,2-bis(3-phenylpropyl)malononitrile 24 using "*in situ* donor"

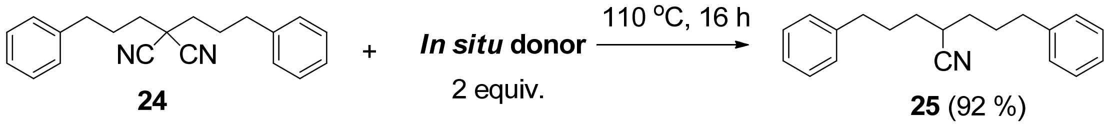

The general reduction reaction procedure was applied to 2,2-bis(3-phenylpropyl)malononitrile **24** (0.0302 g, 0.1 mmol) using the salt **8** (0.308 g, 0.4 mmol) for 16 h at 110 °C. After following the general work-up procedure, the reaction provided 5-phenyl-2-(3-phenylpropyl)pentanenitrile **25**[23] (0.0256 g, 92%) as a colourless oil. [Found: (CI corona$^+$) $(M+H)^+$ 278.1905. $C_{20}H_{24}N$ (M+H) requires 278.1903]; ν_{max}(film)/cm^{-1} 3026, 2941, 2860, 2235, 1602, 1494, 1452, 696; ^{1}H-NMR (400 MHz, $CDCl_3$) δ 1.52-1.80 (6H, m, $CCH_2CH_2CH_2Ar$, $CCH_2CH_2CH_2Ar$), 1.83-1.94 (2H, m, $CCH_2CH_2CH_2Ar$), 2.49-2.56 (1H, m, CHCN), 2.65 (4H, t, *J* = 7.6 Hz, $ArCH_2$), 7.16-7.23 (6H, m, ArH), 7.30 (4H, t, *J* = 7.6 Hz, ArH); ^{13}C-NMR (100 MHz, $CDCl_3$) δ 28.9 (CH_2),

31.6 (CH), 31.8 (CH_2), 35.4 (CH_2), 122.2 (C), 126.2 (CH), 128.5 (CH), 128.6 (CH), 141.4 (C); *m/z* (CI corona$^+$) 278 [$(M+H)^+$, 100%], 249 (15), 203 (13), 95 (16).

Reduction of 2,2-di(pent-4-en-1-yl)malononitrile 26 using "*in situ* donor"

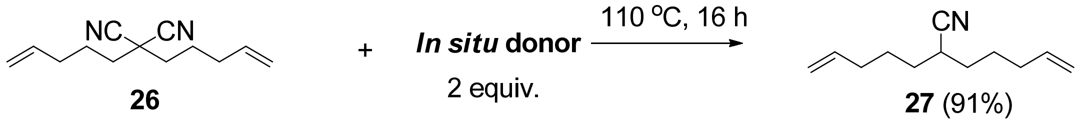

The general reduction reaction procedure was applied to 2,2-di(pent-4-en-1-yl)malononitrile **26** (0.0404 g, 0.2 mmol) using the salt **8** (0.617 g, 0.8 mmol) for 16 h at 110 °C. After following the general work-up procedure, the reaction provided 2-(pent-4-en-1-yl)hept-6-enenitrile **27**[23] (0.0321 g, 91%) as a colourless oil. [Found: (CI corona$^+$) $(M+H)^+$ 178.1587. $C_{12}H_{20}N$ (M+H) requires 178.1590]; ν_{max}(film)/cm^{-1} 3078, 2931, 2862, 2237, 1641, 1460, 910; ^{1}H-NMR (400 MHz, $CDCl_3$) δ 1.53-1.69 (8H, m, $=CHCH_2C\mathit{H_2}CH_2$, $=CHCH_2CH_2C\mathit{H_2}$), 2.08-2.13 (4H, m, $=CHC\mathit{H_2}CH_2CH_2$), 2.50-2.57 (1H, m, C*H*CN), 4.98-5.07 (4H, m, $C\mathit{H_2}=CHCH_2$), 5.74-5.84 (2H, m, $CH_2=C\mathit{H}CH_2$); ^{13}C-NMR (125 MHz, $CDCl_3$) δ 26.4 (CH_2), 31.5 (CH), 31.7 (CH_2), 33.2 (CH_2), 115.4 (CH_2), 122.3 (C), 137.8 (CH); *m/z* (CI corona$^+$) 195 [$(M+NH_4)^+$, 11%], 178 [$(M+H)^+$, 100%], 117 (14).

Reduction of 2,2-*bis*(3-methylbut-2-en-1-yl)malononitrile 28 using "*in situ* donor"

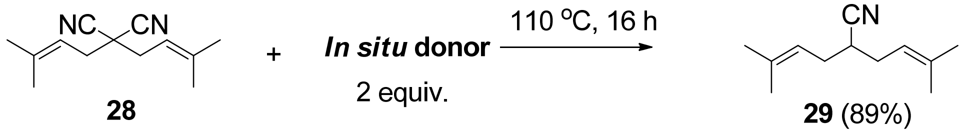

The general reduction reaction procedure was applied to 2,2-*bis*(3-methylbut-2-en-1-yl)malononitrile **28** (0.0404 g, 0.2 mmol) using the salt **8** (0.617 g, 0.8 mmol) for 16 h at 110 °C. After following the general work-up procedure, the reaction provided 5-methyl-2-(3-methylbut-2-en-1-yl)hex-4-enenitrile **29**[23] (0.0314 g, 89%) as a colourless oil. ^{1}H-NMR (400 MHz, $CDCl_3$) δ 1.65 (6H, s, $=(CH_3)(C\mathit{H_3})$), 1.75 (6H, s, $=(C\mathit{H_3})(CH_3)$), 2.24-2.36 (4H, m, $=CHC\mathit{H_2}$), 2.48-2.55 (1H, m, C*H*CN), 5.16-5.20 (2H, m, $=C\mathit{H}CH_2$); ^{13}C-NMR (100 MHz, $CDCl_3$) δ 18.1 (CH_3), 25.9 (CH_3), 30.3 (CH_2), 32.5 (CH), 119.3 (CH), 122.4 (C), 135.9 (C).

Reduction of 2,2-dimethylhexadecanenitrile 30 using "*in situ* donor"

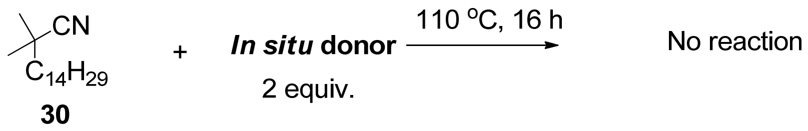

The general reduction reaction procedure was applied to 2,2-dimethylhexadecanenitrile **30** (0.0265 g, 0.1 mmol) using the salt **8** (0.308 g, 0.4 mmol) for 16 h at 110 °C. After following the general work-up procedure, the reaction provided recovery of starting material (0.0246 g, 93%) only.

4. Electrochemical studies of compounds 1, S1a and 4

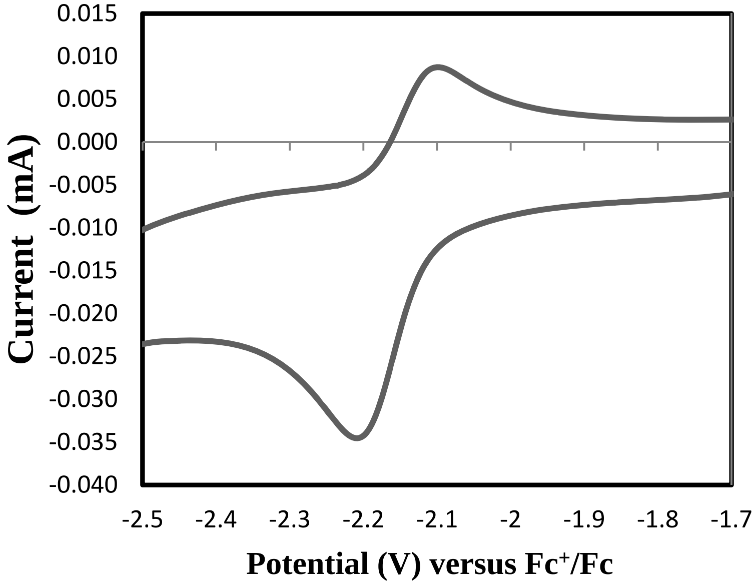

Figure S2. Cyclic voltammogram of **1** vs Fc^+/Fc in DMF with [nBu_4NPF_6] as supporting electrolyte. Scan rate was 50 mV/s.

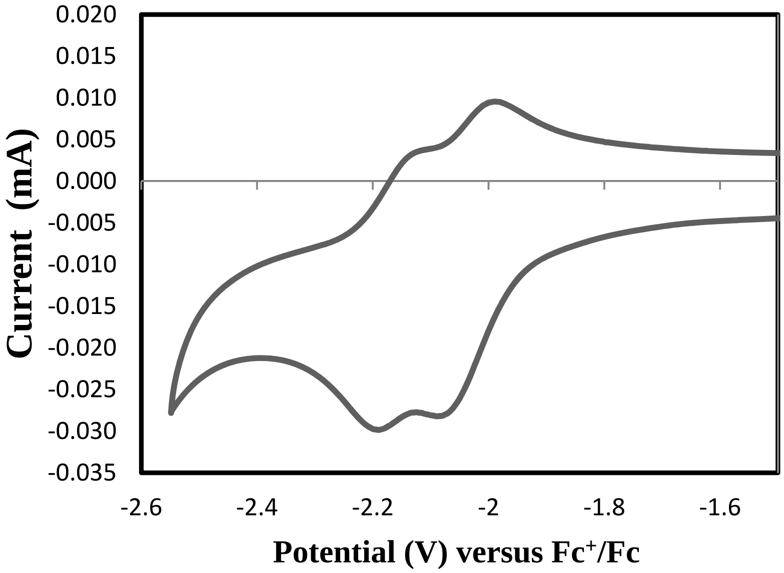

Figure S3. Cyclic voltammogram of **S1a** vs Fc^{+}/Fc in DMF with [nBu_4NPF_6] as supporting electrolyte. Scan rate was 50 mV/s.

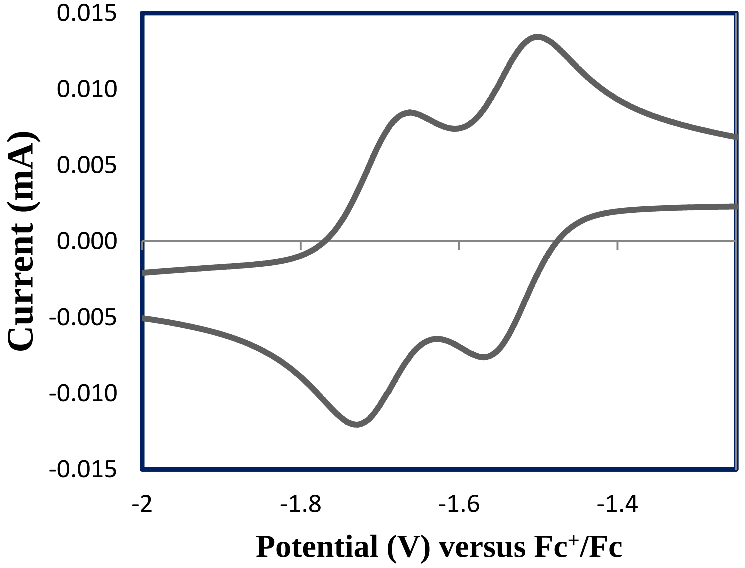

Figure S4. Cyclic voltammogram of **4** vs Fc^{+}/Fc in DMF with [nBu_4NPF_6] as supporting electrolyte. Scan rate was 50 mV/s.

5. References

[1] Q. Zhang, C. Shi, H. R. Zhang, K. K. Wang, *J. Org. Chem.* **2000**, *65*, 7977-7983.

[2] I. Yavari, M. Adib, L. Hojabri, *Tetrahedron* **2002**, *58*, 7213-7219.

[3] J. Y. Balandier, A. Belyasmine, M. Sallé, *Eur. J. Org. Chem.* **2008**, *2008*, 269-276.

[4] M. Taillefer, N. Rahier, A. Hameau, J. Volle, *Chem. Commun.* **2006**, 3238-3239.

[5] H. J. Cristau, M. Taillefer, N. Rahier, *J. Organomet. Chem.* **2002**, *646*, 94-106.

[6] H. J. Cristau, L. Chiche, J. Kadoura, E. Torreilles, *Tetrahedron Lett.* **1988**, *29*, 3931-3934.

[7] F. Schoenebeck, J. A. Murphy, S. Zhou, Y. Uenoyama, Y. Miclo, T. Tuttle, *J. Am. Chem. Soc.* **2007**, *129*, 13368-13369.

[8] S. Kim, T. A. Lee, *Synlett* **1997**, 950.

[9] Z. Liu, R. C. Larock, *J. Org. Chem.* **2006**, *71*, 3198.

[10] H. F. Hodson, D. J. Madge, A. N. Slawin, D. A. Widowson, D. J. Williams, *Tetrahedron* **1994**, *50*, 1899.

[11] S. O'Sullivan, E. Doni, T. Tuttle, J. A. Murphy, *Angew. Chem. Int. Ed.* **2014**, *53*, 474-478.

[12] N. J. Findlay, S. R. Park, F. Schoenebeck, E. Cahard, S. Zhou, L. E. Berlouis, M. D. Spicer, T. Tuttle, J. A. Murphy, *J. Am. Chem. Soc.* **2010**, *132*, 15462-15464.

[13] Z. Zhang, J. Mao, D. Zhu, F. Wu, H. Chen, B. Wan, *Tetrahedron* **2006**, *62*, 4435.

[14] H. R. Snyder, E. P. Merica, C. R. Force, E. G. White, *J. Am. Chem. Soc.* **1958**, *80*, 4622-4625.

[15] J. Garnier, D. W. Thomson, S. Zhou, P. I. Jolly, L. E. A. Berlouis, J. A. Murphy, *Beilstein J. Org. Chem.* **2012**, *8*, 994-1002.

[16] M. S. Goedheijt, B. E. Hanson, J. N. H. Reek, P. C. J. Kamer, Van Leeuwen, P. W. N., *J. Am. Chem. Soc.* **2000**, *122*, 1650-1657.

[17] E. Cahard, F. Schoenebeck, J. Garnier, S. P. Y. Cutulic, S. Zhou, J. A. Murphy, *Angew. Chem. Int. Ed.* **2012**, *51*, 3673-3676.

[18] D. P. Curran, M. J. Totleben, *J. Am. Chem. Soc.* **1992**, *114*, 6050.

[19] R. Trivedi, J. A. Tunge, *Org. Lett.* **2009**, *11*, 5650.

[20] A. Dahlen, A. Peterson, G. Hilmerson, *Org. Biomol. Chem.* **2003**, *1*, 2423-2426.

[21] X. Huo, M. Quan, G. Yang, X. Zhao , D. Liu, W. Zhang, *Org. Lett.* **2014**, *16*, 1570-1573.

[22] M. Tobisu, R. Nakamura, Y. Kita, N. Chatani, *J. Am. Chem. Soc.* **2009**, *131*, 3174-3175.

[23] E. Doni, J. A. Murphy, *Org. Chem. Front.* **2014**, *1*, 1072-1076.

[24] D. P. Curran, C. M. Seong, *Synlett* **1991**, 107.

6. Spectral Data

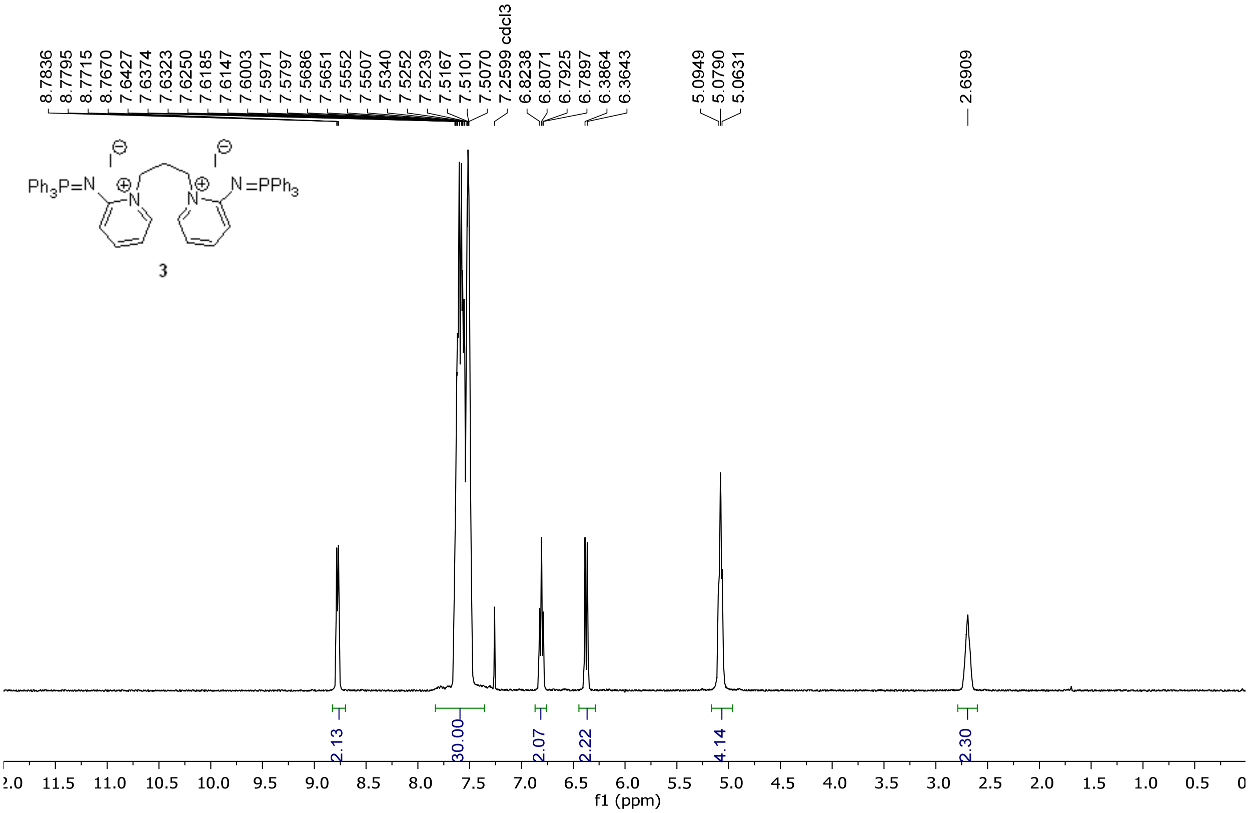

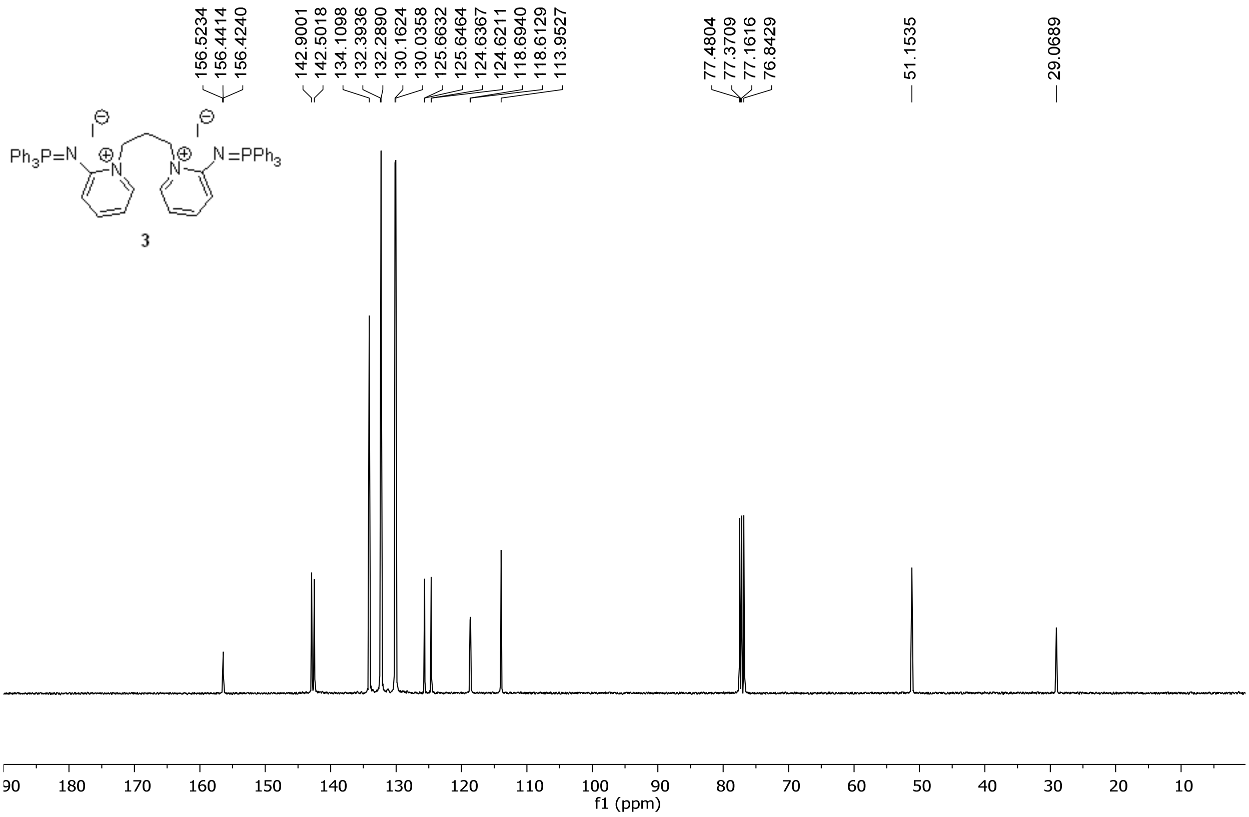
156.5234
156.4414
156.4240
142.9001
142.5018
134.1098
132.3936
132.2890
130.1624
130.0358
125.6632
125.6464
124.6367
124.6211
118.6940
118.6129
113.9527
77.4804
77.3709
77.1616
76.8429
51.1535
29.0689
Ph3P=N
N=PPh3
3
f1 (ppm)

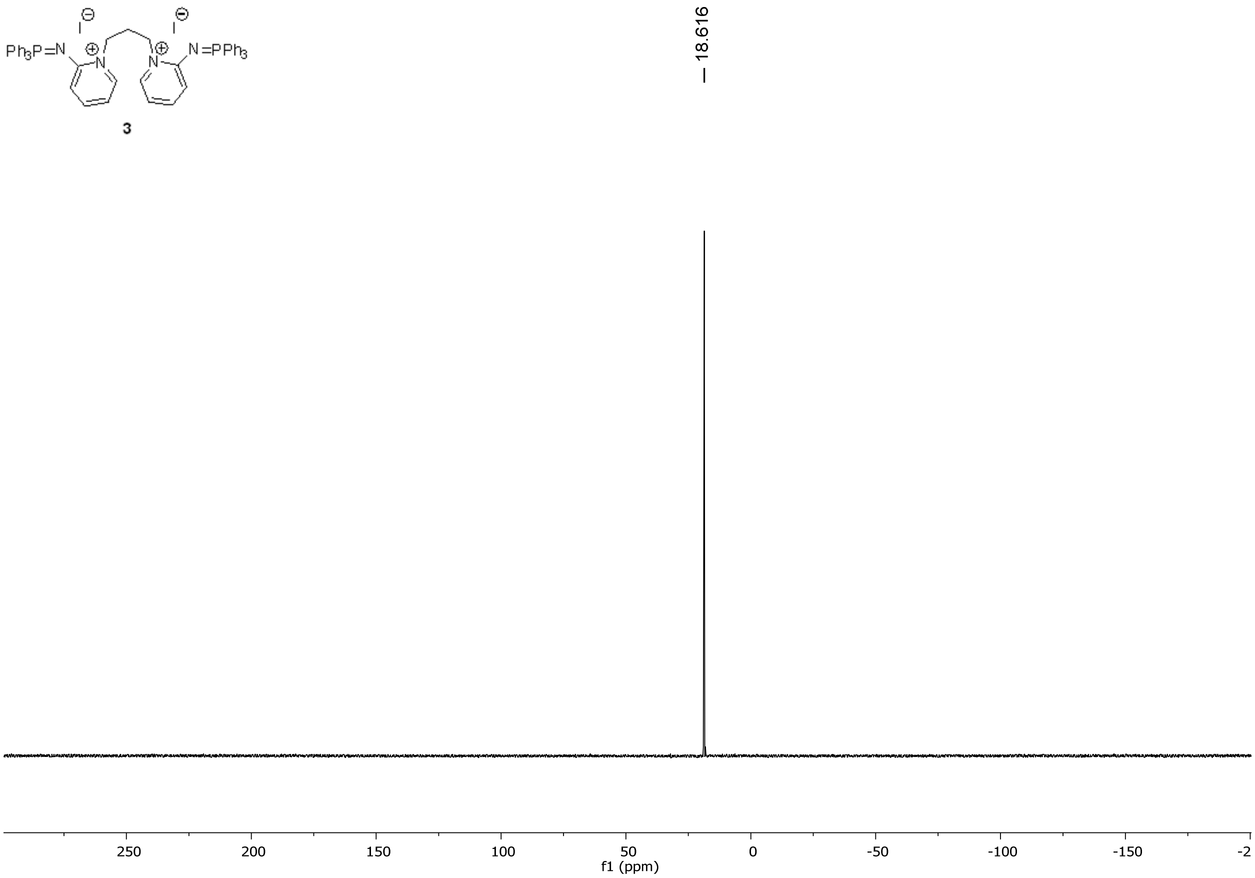
18.616
Ph3P=N
N=PPh3
3
f1 (ppm)

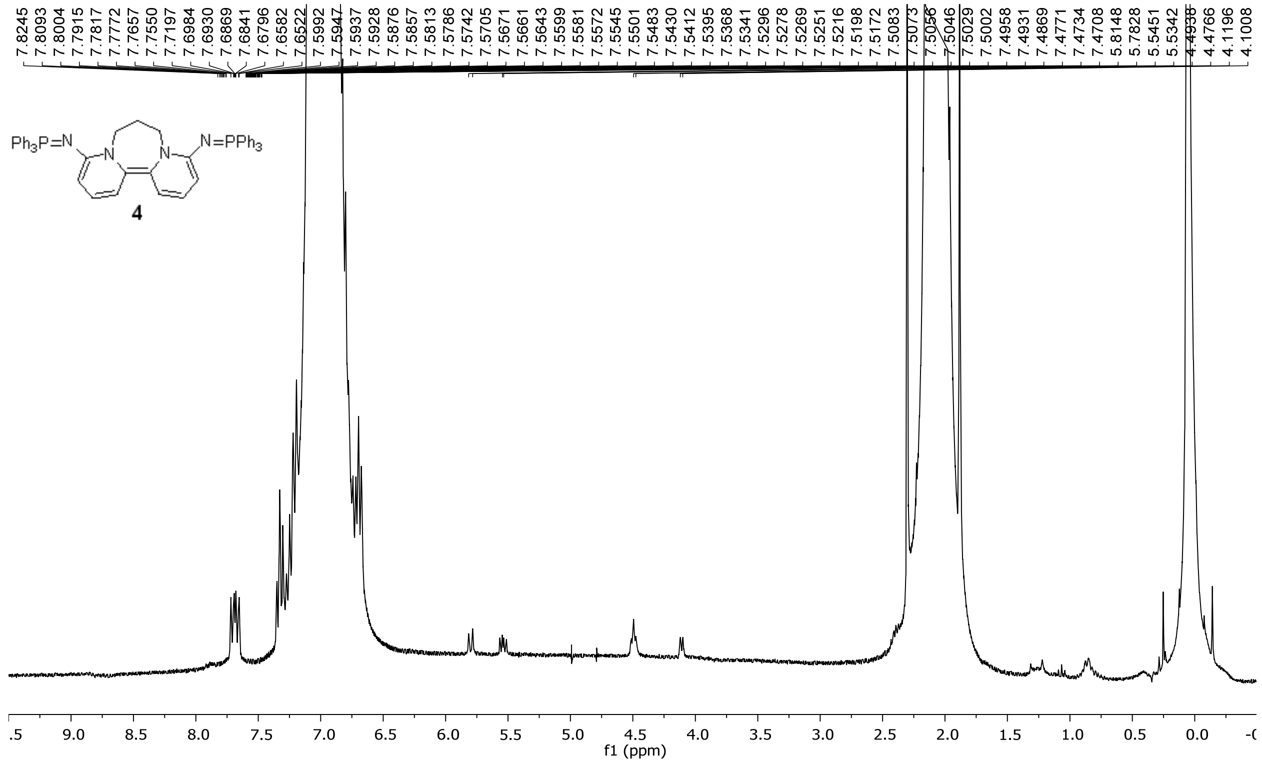

^{1}H NMR of reaction mixture of compound **4** in toluene

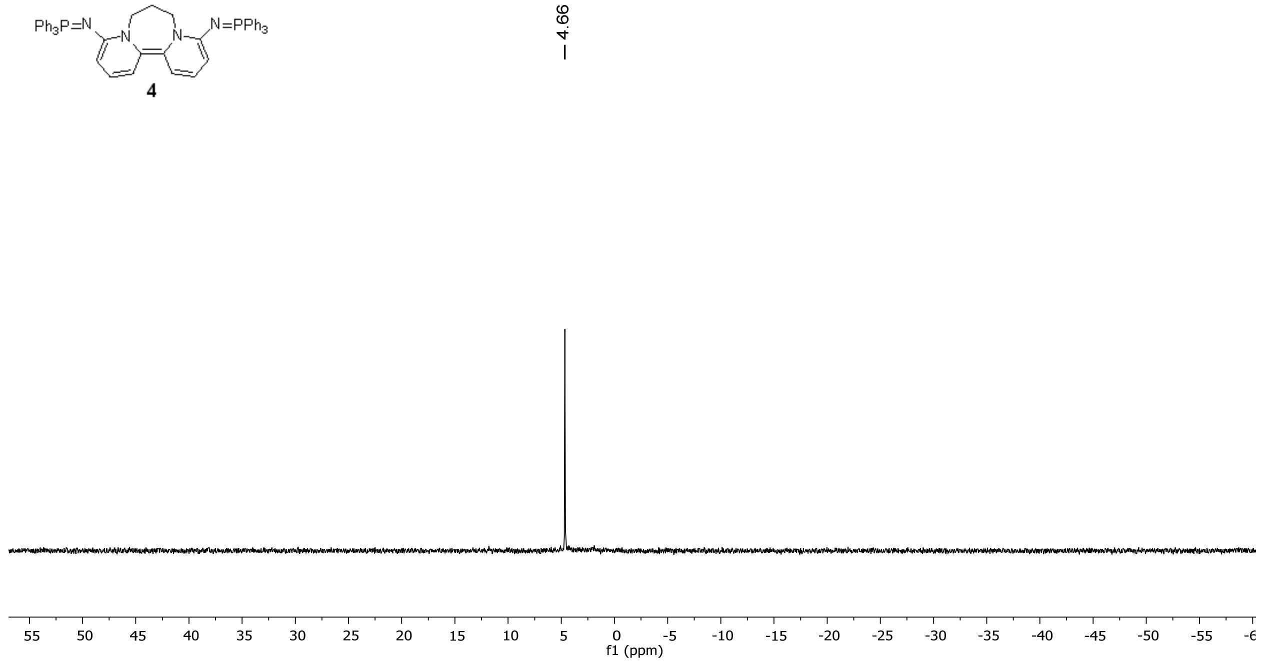

^{31}P NMR of reaction mixture of compound **4** in toluene

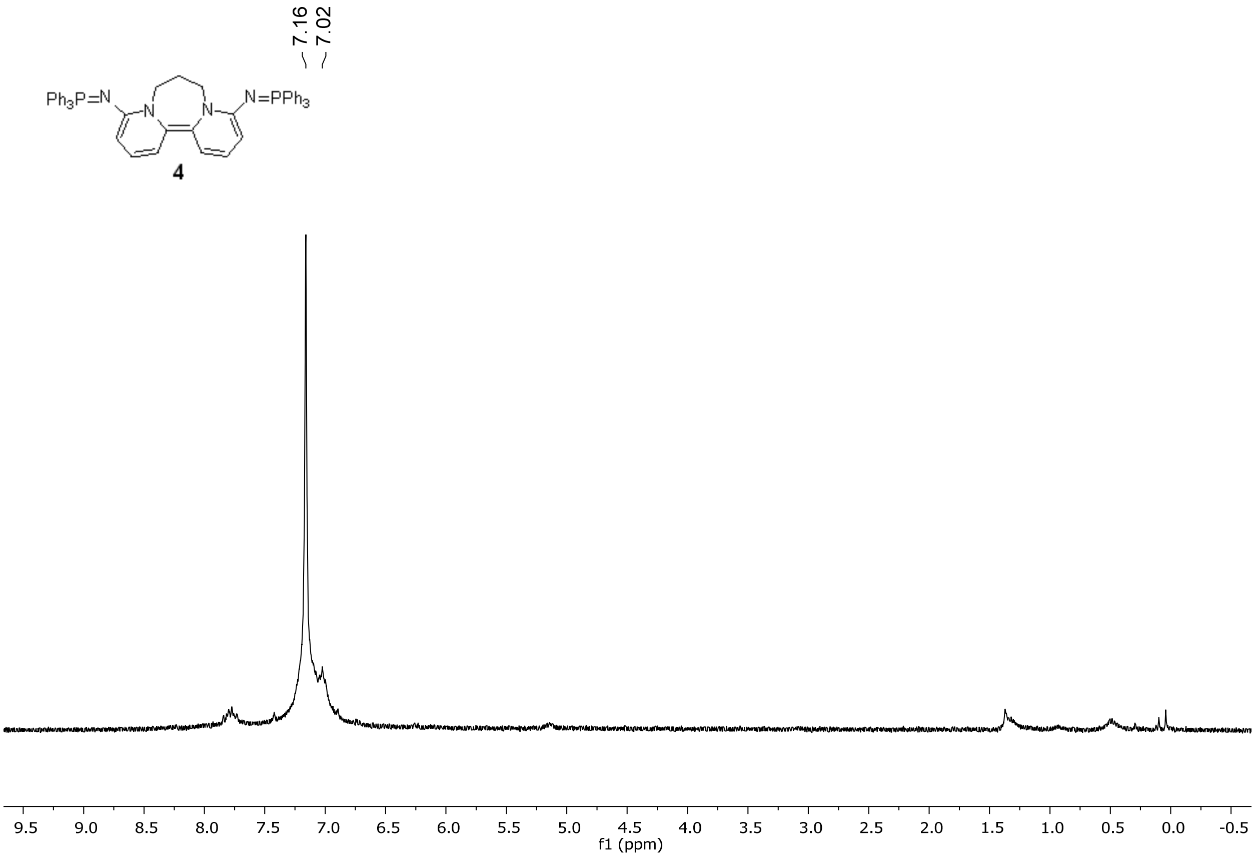

^{1}H NMR of purple solid of **4** in C_6D_6

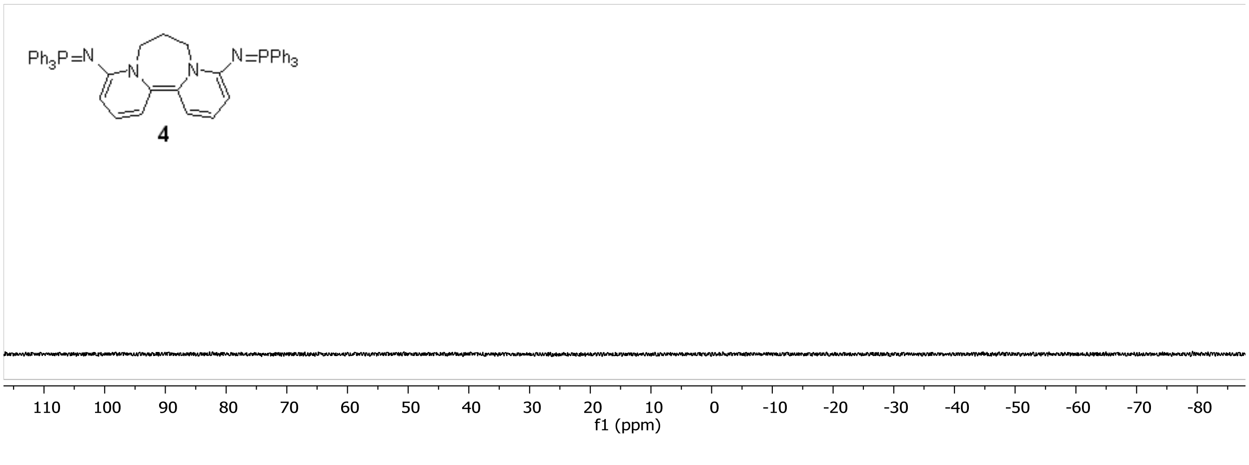

^{31}P NMR of purple solid of **4** in C_6D_6

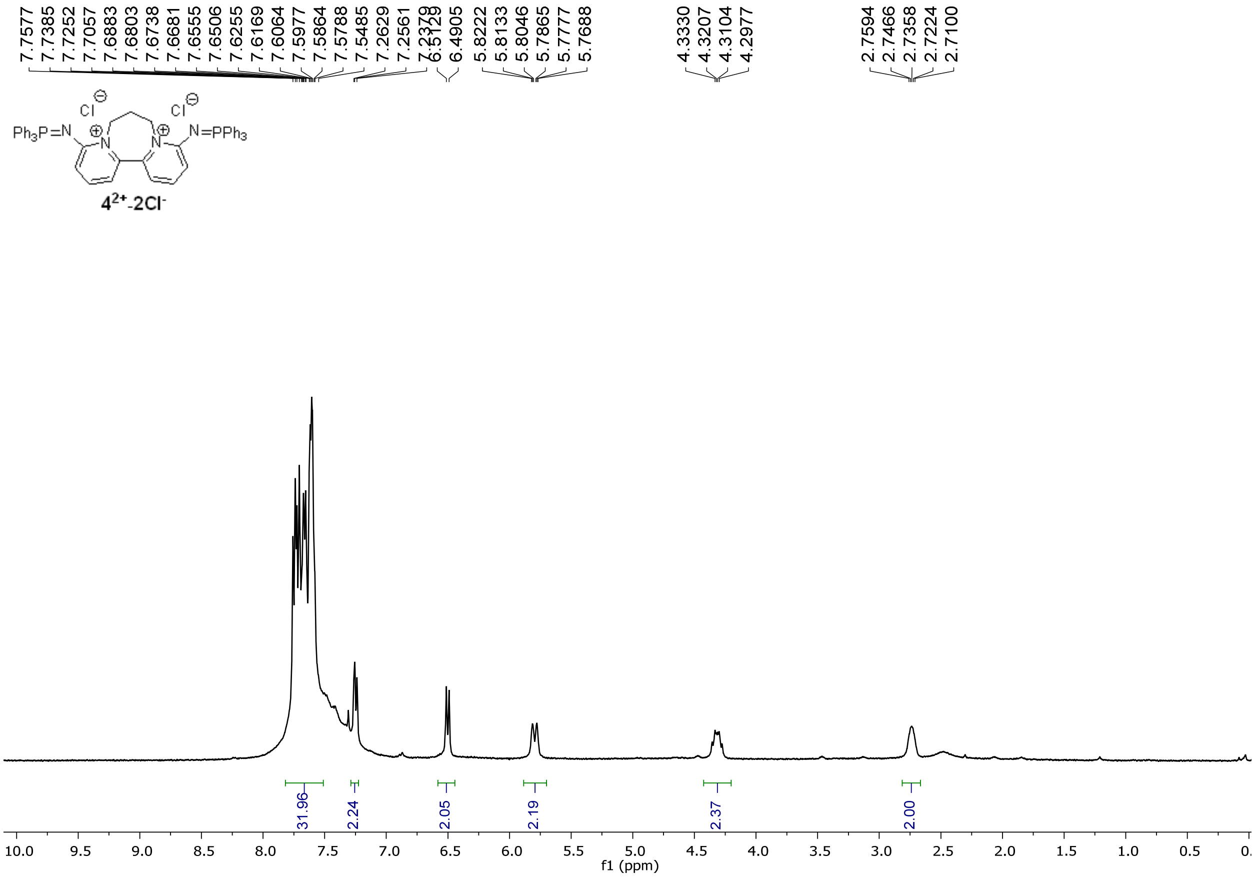

7.7577
7.7385
7.7252
7.7057
7.6883
7.6803
7.6738
7.6681
7.6555
7.6506
7.6255
7.6169
7.6064
7.5977
7.5864
7.5788
7.5485
7.2629
7.2561
7.2379
6.5129
6.4905
5.8222
5.8133
5.8046
5.7865
5.7777
5.7688
4.3330
4.3207
4.3104
4.2977
2.7594
2.7466
2.7358
2.7224
2.7100
Ph3P=N
N=PPh3
Cl
4²⁺-2Cl⁻
31.96
2.24
2.05
2.19
2.37
2.00
10.0
9.5
9.0
8.5
8.0
7.5
7.0
6.5
6.0
5.5
5.0
4.5
4.0
3.5
3.0
2.5
2.0
1.5
1.0
0.5
f1 (ppm)

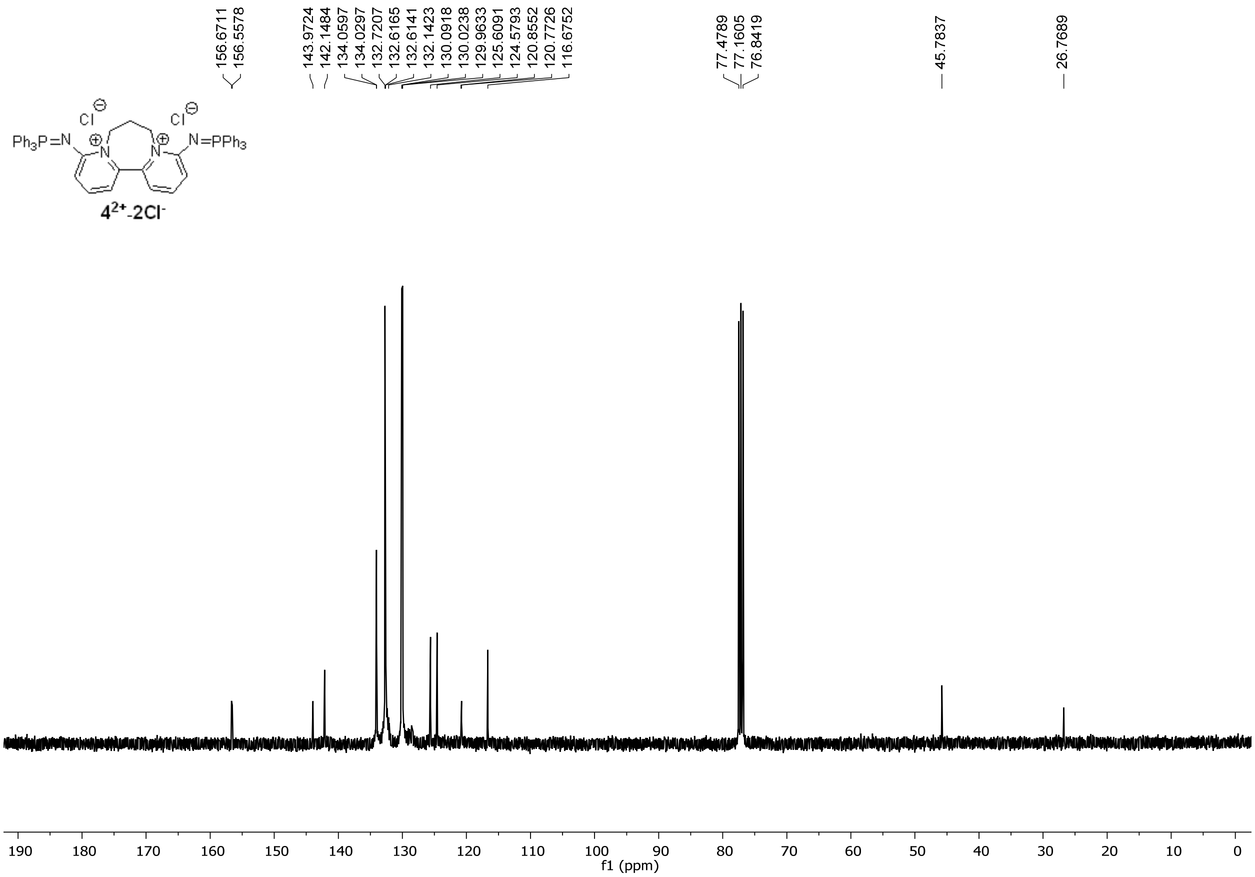
156.6711
156.5578
143.9724
142.1484
134.0597
134.0297
132.7207
132.6165
132.6141
132.1423
130.0918
130.0238
129.9633
125.6091
124.5793
120.8552
120.7726
116.6752
77.4789
77.1605
76.8419
45.7837
26.7689
Ph3P=N
N=PPh3
Cl
4 2+ -2Cl -
190 180 170 160 150 140 130 120 110 100 90 80 70 60 50 40 30 20 10 0
f1 (ppm)

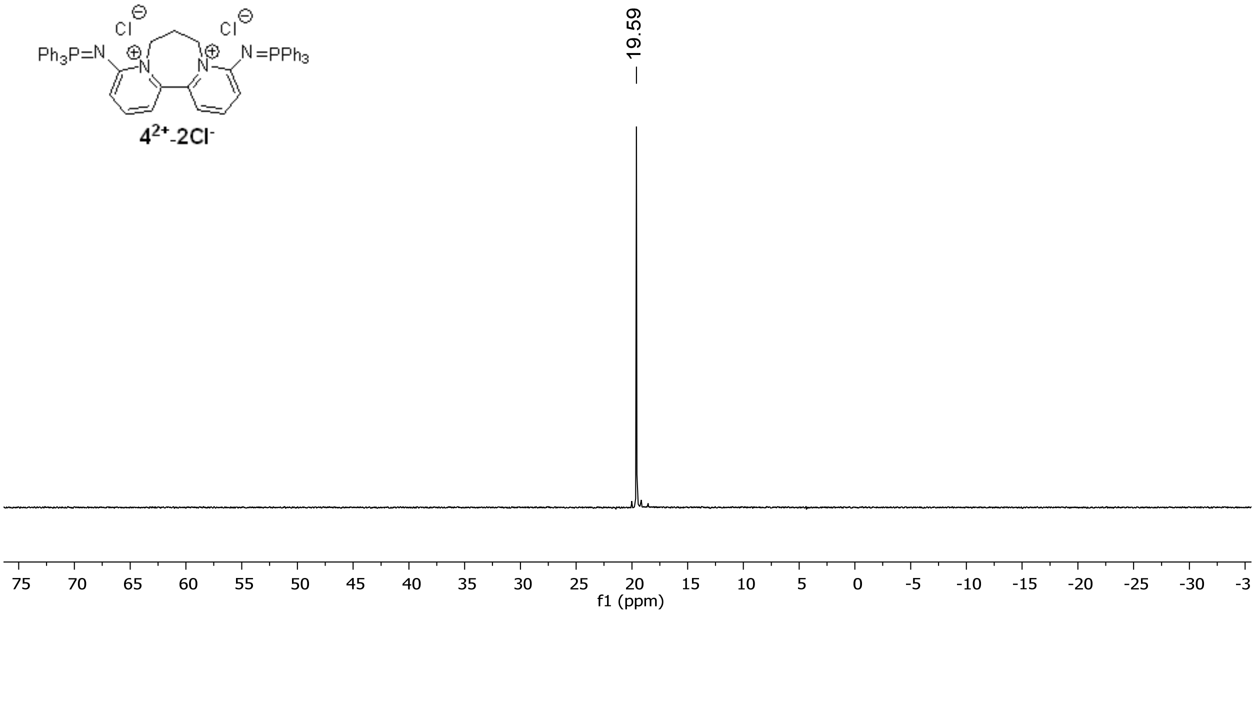
19.59
Ph3P=N
N=PPh3
Cl
4 2+ -2Cl -
75 70 65 60 55 50 45 40 35 30 25 20 15 10 5 0 -5 -10 -15 -20 -25 -30 -3
f1 (ppm)

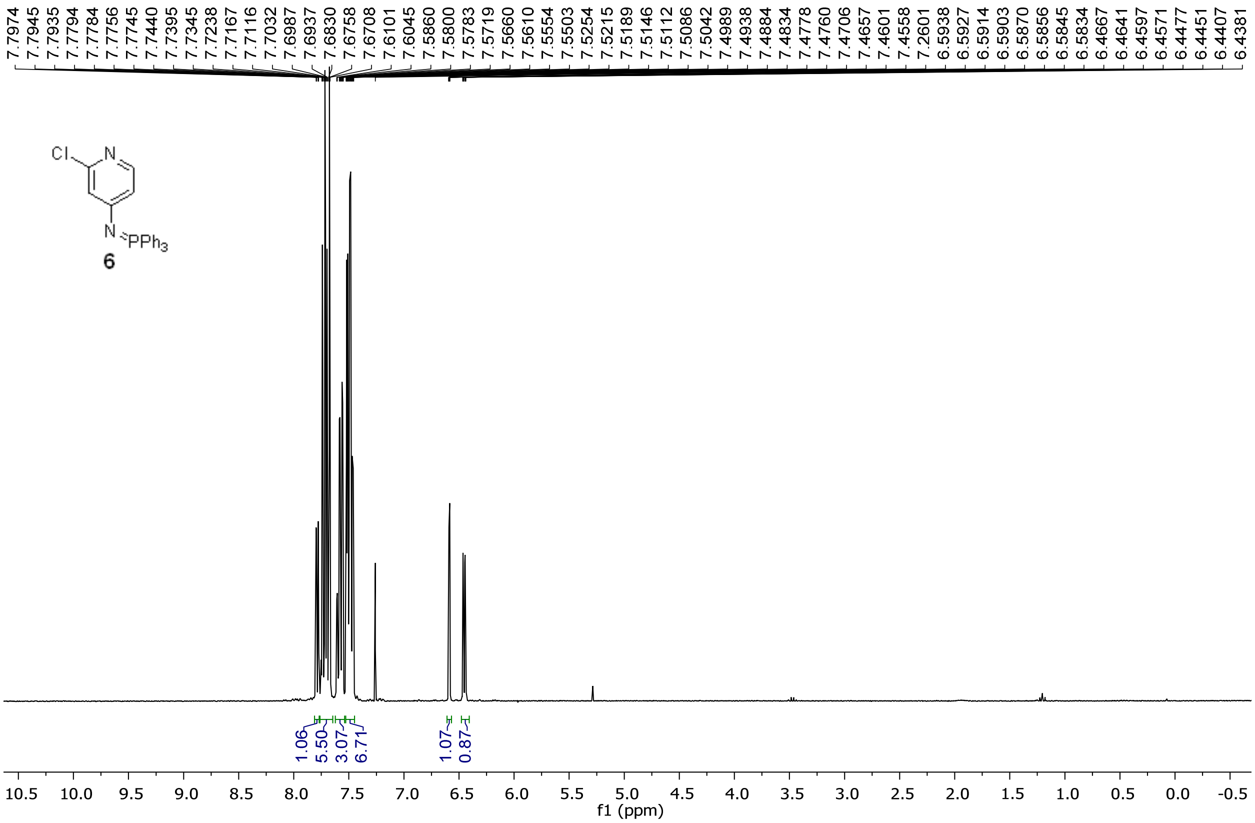
7.7974
7.7945
7.7935
7.7794
7.7784
7.7756
7.7745
7.7440
7.7395
7.7345
7.7238
7.7167
7.7116
7.7032
7.6987
7.6937
7.6830
7.6758
7.6708
7.6101
7.6045
7.5860
7.5800
7.5783
7.5719
7.5660
7.5610
7.5554
7.5503
7.5254
7.5215
7.5189
7.5146
7.5112
7.5086
7.5042
7.4989
7.4938
7.4884
7.4834
7.4778
7.4760
7.4706
7.4657
7.4601
7.4558
7.2601
6.5938
6.5927
6.5914
6.5903
6.5870
6.5856
6.5845
6.5834
6.4667
6.4641
6.4597
6.4571
6.4477
6.4451
6.4407
6.4381
Cl
N
N
PPh3
6
1.06
5.50
3.07
6.71
1.07
0.87
10.5 10.0 9.5 9.0 8.5 8.0 7.5 7.0 6.5 6.0 5.5 5.0 4.5 4.0 3.5 3.0 2.5 2.0 1.5 1.0 0.5 0.0 -0.5
f1 (ppm)

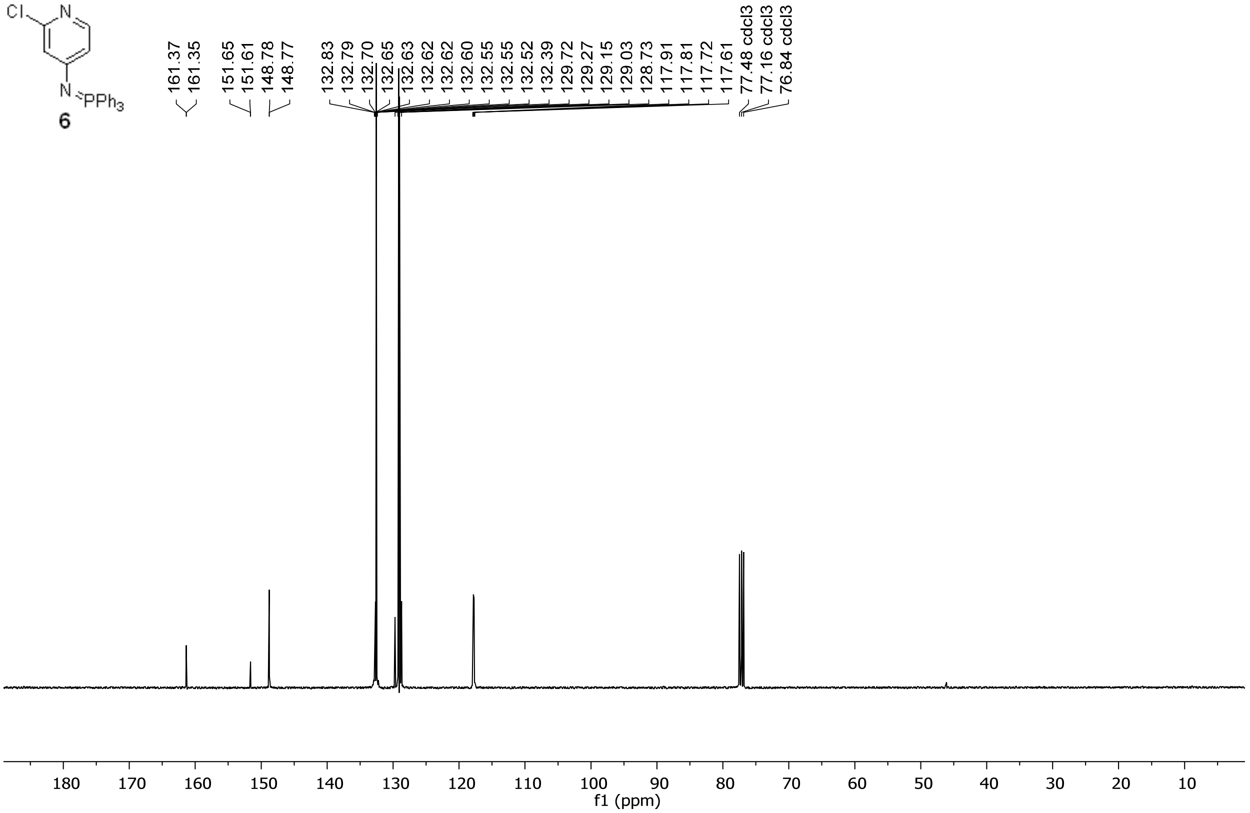
Cl
N
N
PPh3
6
161.37
161.35
151.65
151.61
148.78
148.77
132.83
132.79
132.70
132.65
132.63
132.62
132.62
132.60
132.55
132.55
132.52
132.39
129.72
129.27
129.15
129.03
128.73
117.91
117.81
117.72
117.61
77.48 cdcl3
77.16 cdcl3
76.84 cdcl3
180 170 160 150 140 130 120 110 100 90 80 70 60 50 40 30 20 10
f1 (ppm)

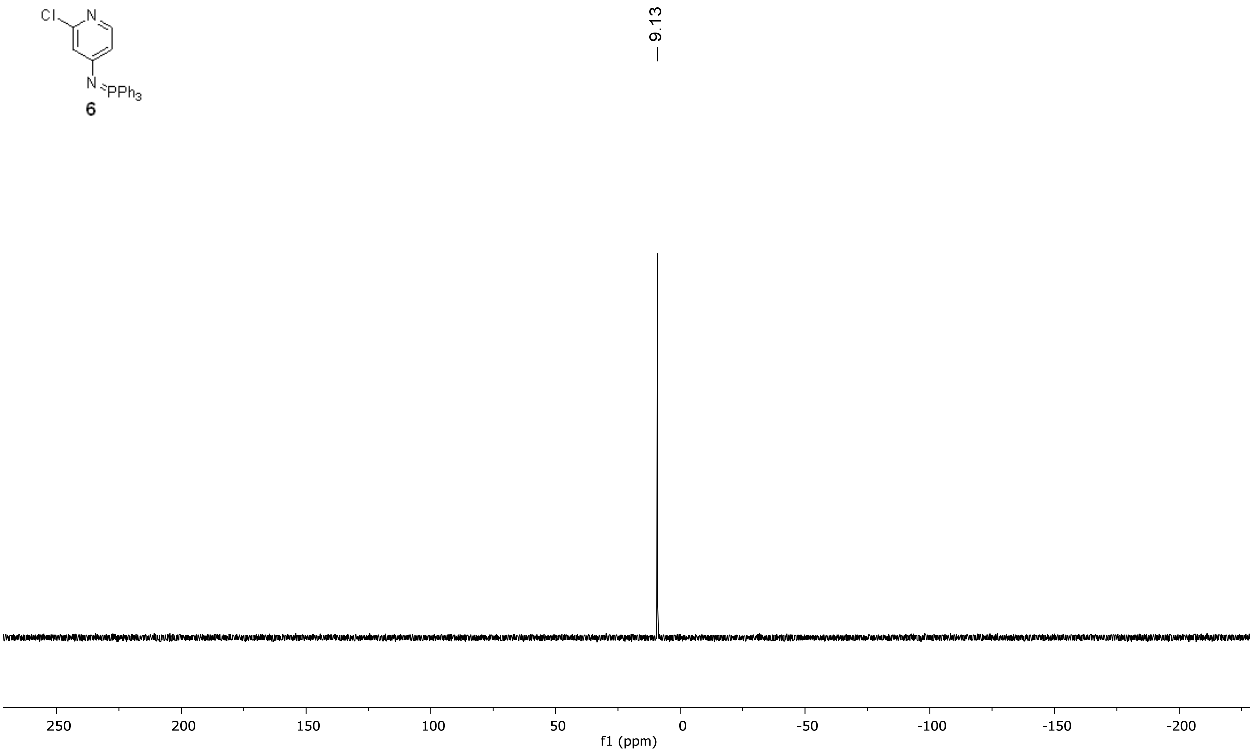
Cl
N
N
PPh3
6
9.13
250
200
150
100
50
0
-50
-100
-150
-200
f1 (ppm)

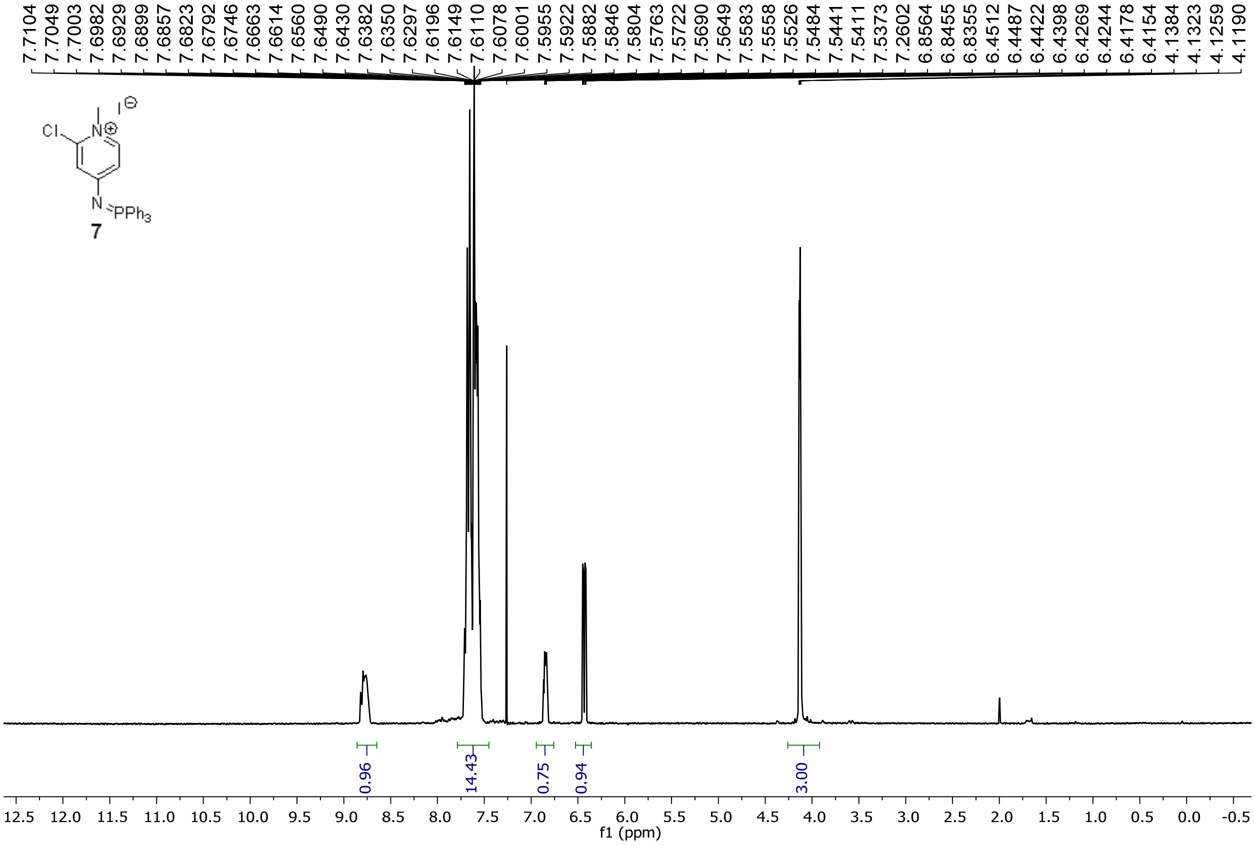
7.7104
7.7049
7.7003
7.6982
7.6929
7.6899
7.6857
7.6823
7.6792
7.6746
7.6663
7.6614
7.6560
7.6490
7.6430
7.6382
7.6350
7.6297
7.6196
7.6149
7.6110
7.6078
7.6001
7.5955
7.5922
7.5882
7.5846
7.5804
7.5763
7.5722
7.5690
7.5649
7.5583
7.5558
7.5526
7.5484
7.5441
7.5411
7.5373
7.2602
6.8564
6.8455
6.8355
6.4512
6.4487
6.4422
6.4398
6.4269
6.4244
6.4178
6.4154
4.1384
4.1323
4.1259
4.1190
Cl
N
I
N
PPh3
7
0.96
14.43
0.75
0.94
3.00
12.5
12.0
11.5
11.0
10.5
10.0
9.5
9.0
8.5
8.0
7.5
7.0
6.5
6.0
5.5
5.0
4.5
4.0
3.5
3.0
2.5
2.0
1.5
1.0
0.5
0.0
-0.5
f1 (ppm)

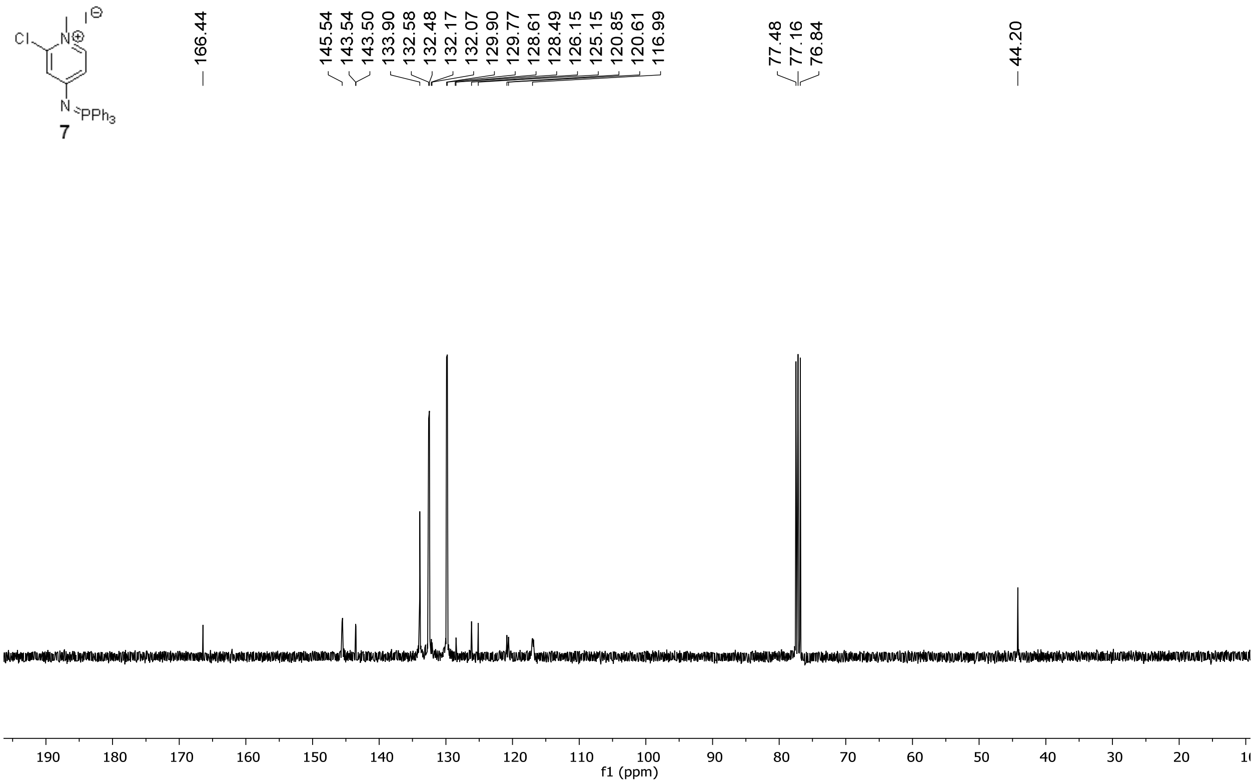
Cl
N⊕
I⊖
N=PPh3
7
166.44
145.54
143.54
143.50
133.90
132.58
132.48
132.17
132.07
129.90
129.77
128.61
128.49
126.15
125.15
120.85
120.61
116.99
77.48
77.16
76.84
44.20
190 180 170 160 150 140 130 120 110 100 90 80 70 60 50 40 30 20
f1 (ppm)

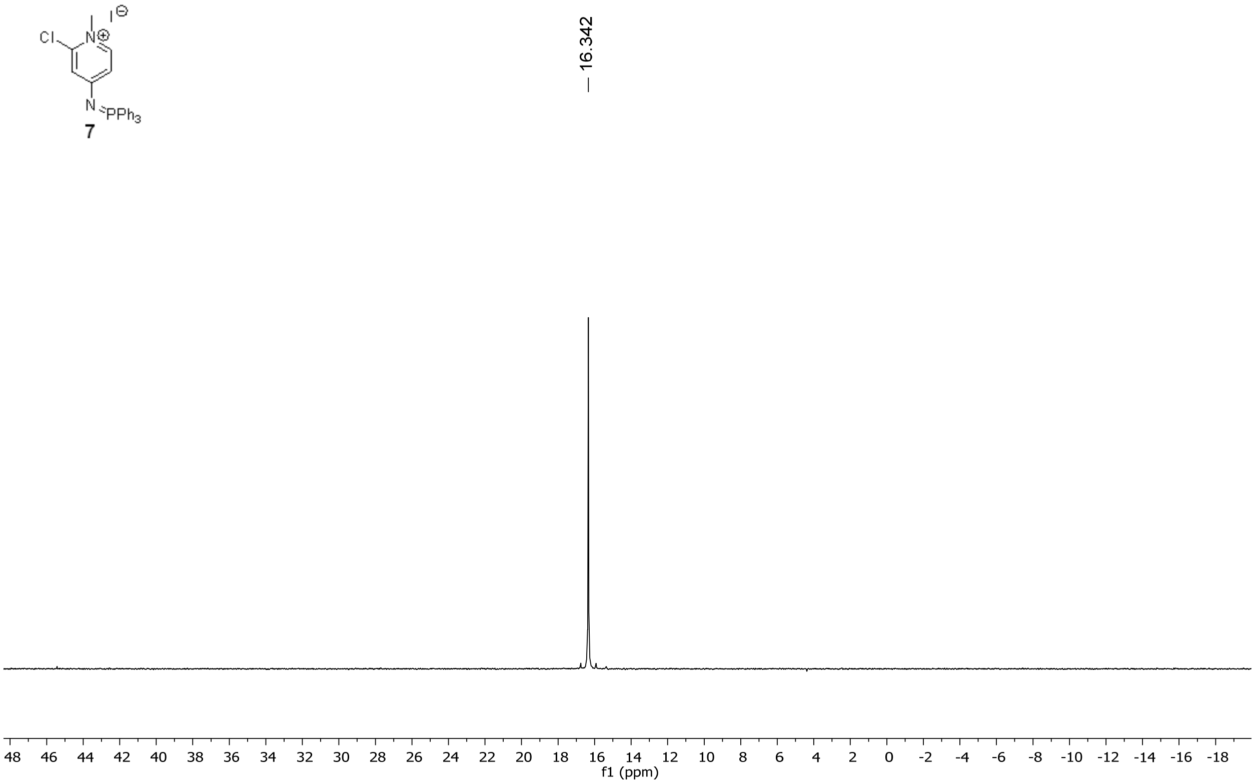
Cl
N⊕
I⊖
N=PPh3
7
16.342
48 46 44 42 40 38 36 34 32 30 28 26 24 22 20 18 16 14 12 10 8 6 4 2 0 -2 -4 -6 -8 -10 -12 -14 -16 -18
f1 (ppm)

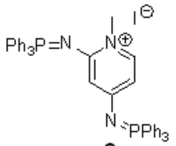
Ph3P=N
I⊖
N⊕
N
PPh3

8

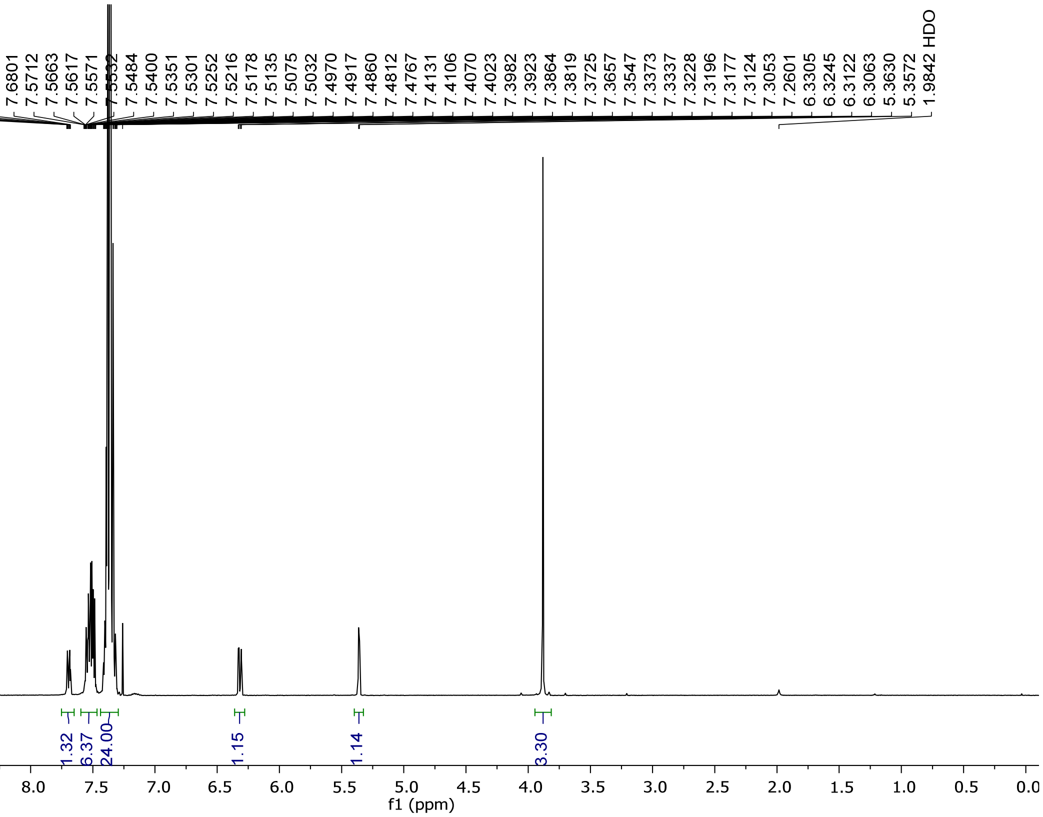
7.7090
7.7038
7.6985
7.6906
7.6854
7.6801
7.5712
7.5663
7.5617
7.5571
7.5532
7.5484
7.5400
7.5351
7.5301
7.5252
7.5216
7.5178
7.5135
7.5075
7.5032
7.4970
7.4917
7.4860
7.4812
7.4767
7.4131
7.4106
7.4070
7.4023
7.3982
7.3923
7.3864
7.3819
7.3725
7.3657
7.3547
7.3373
7.3337
7.3228
7.3196
7.3177
7.3124
7.3053
7.2601
6.3305
6.3245
6.3122
6.3063
5.3630
5.3572
1.9842 HDO
1.32
6.37
24.00
1.15
1.14
3.30
9.5
9.0
8.5
8.0
7.5
7.0
6.5
6.0
5.5
5.0
4.5
4.0
3.5
3.0
2.5
2.0
1.5
1.0
0.5
0.0
f1 (ppm)

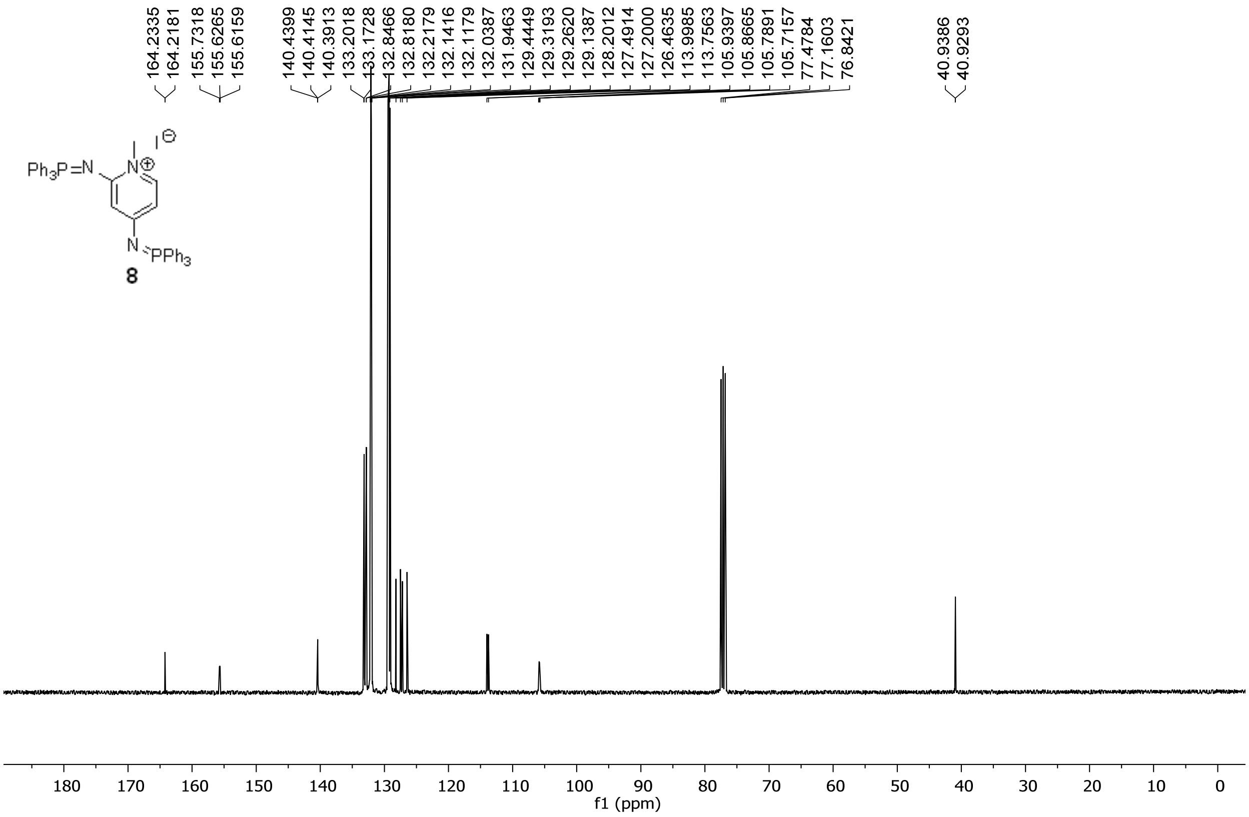

164.2335
164.2181
155.7318
155.6265
155.6159
140.4399
140.4145
140.3913
133.2018
133.1728
132.8466
132.8180
132.2179
132.1416
132.1179
132.0387
131.9463
129.4449
129.3193
129.2620
129.1387
128.2012
127.4914
127.2000
126.4635
113.9985
113.7563
105.9397
105.8665
105.7891
105.7157
77.4784
77.1603
76.8421
40.9386
40.9293
Ph3P=N
I⊖
N⊕
N=PPh3
8
180 170 160 150 140 130 120 110 100 90 80 70 60 50 40 30 20 10 0
f1 (ppm)

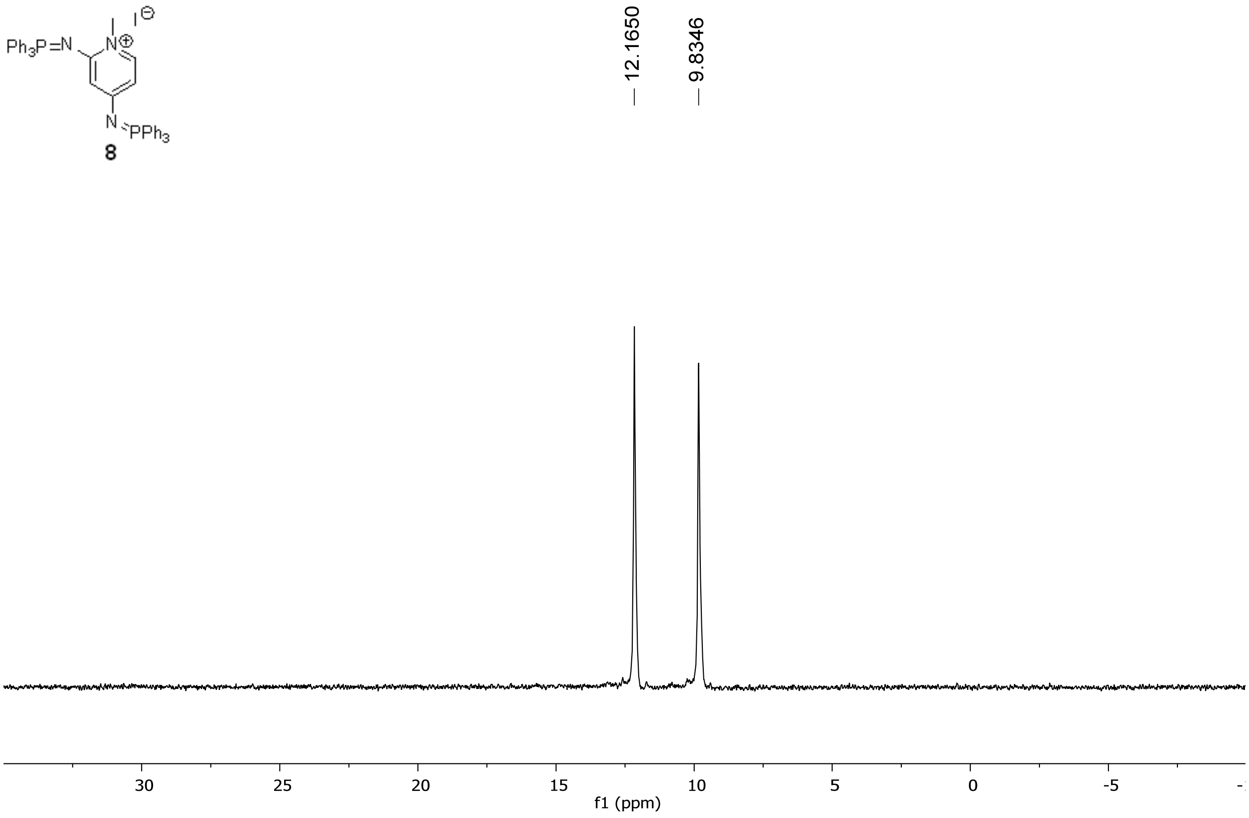

Ph3P=N
I⊖
N⊕
N=PPh3
8
12.1650
9.8346
30 25 20 15 10 5 0 -5
f1 (ppm)

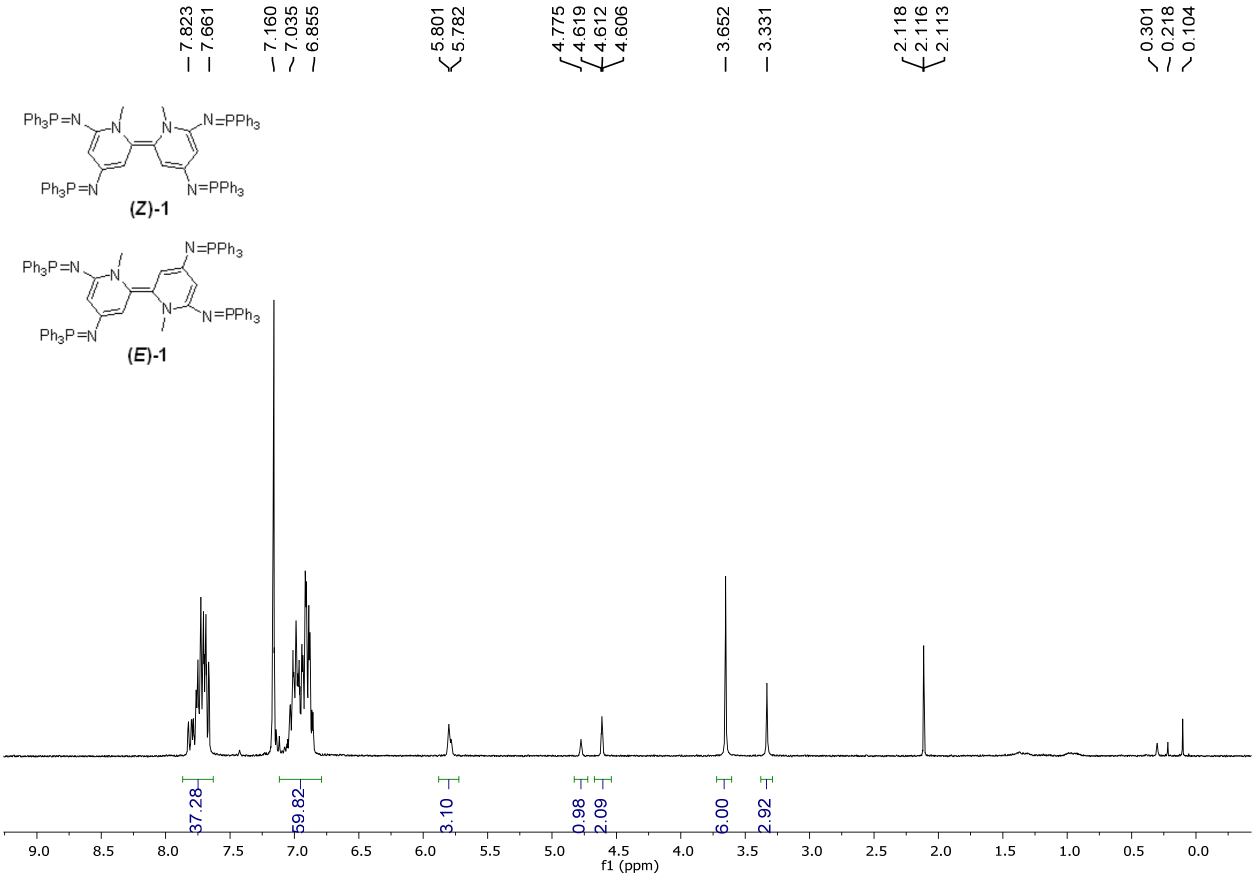
7.823
7.661
7.160
7.035
6.855
5.801
5.782
4.775
4.619
4.612
4.606
3.652
3.331
2.118
2.116
2.113
0.301
0.218
0.104
(Z)-1
(E)-1
37.28
59.82
3.10
0.98
2.09
6.00
2.92
9.0 8.5 8.0 7.5 7.0 6.5 6.0 5.5 5.0 4.5 4.0 3.5 3.0 2.5 2.0 1.5 1.0 0.5 0.0
f1 (ppm)

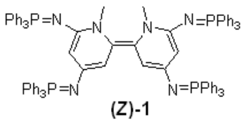
Ph3P=N
N=PPh3
Ph3P=N
N=PPh3

(*Z*)-1

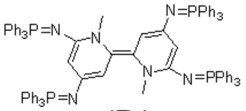
N=PPh3
Ph3P=N
N=PPh3
Ph3P=N

(*E*)-1

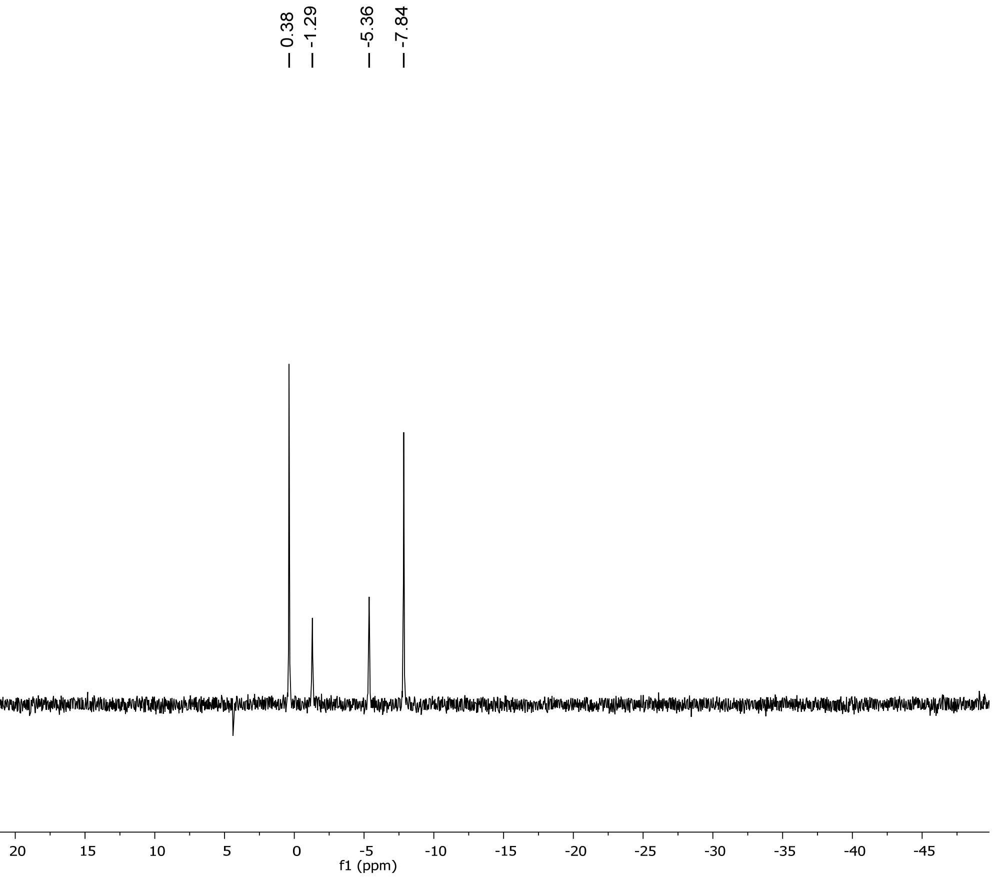
0.38
-1.29
-5.36
-7.84
35
30
25
20
15
10
5
0
-5
-10
-15
-20
-25
-30
-35
-40
-45
f1 (ppm)

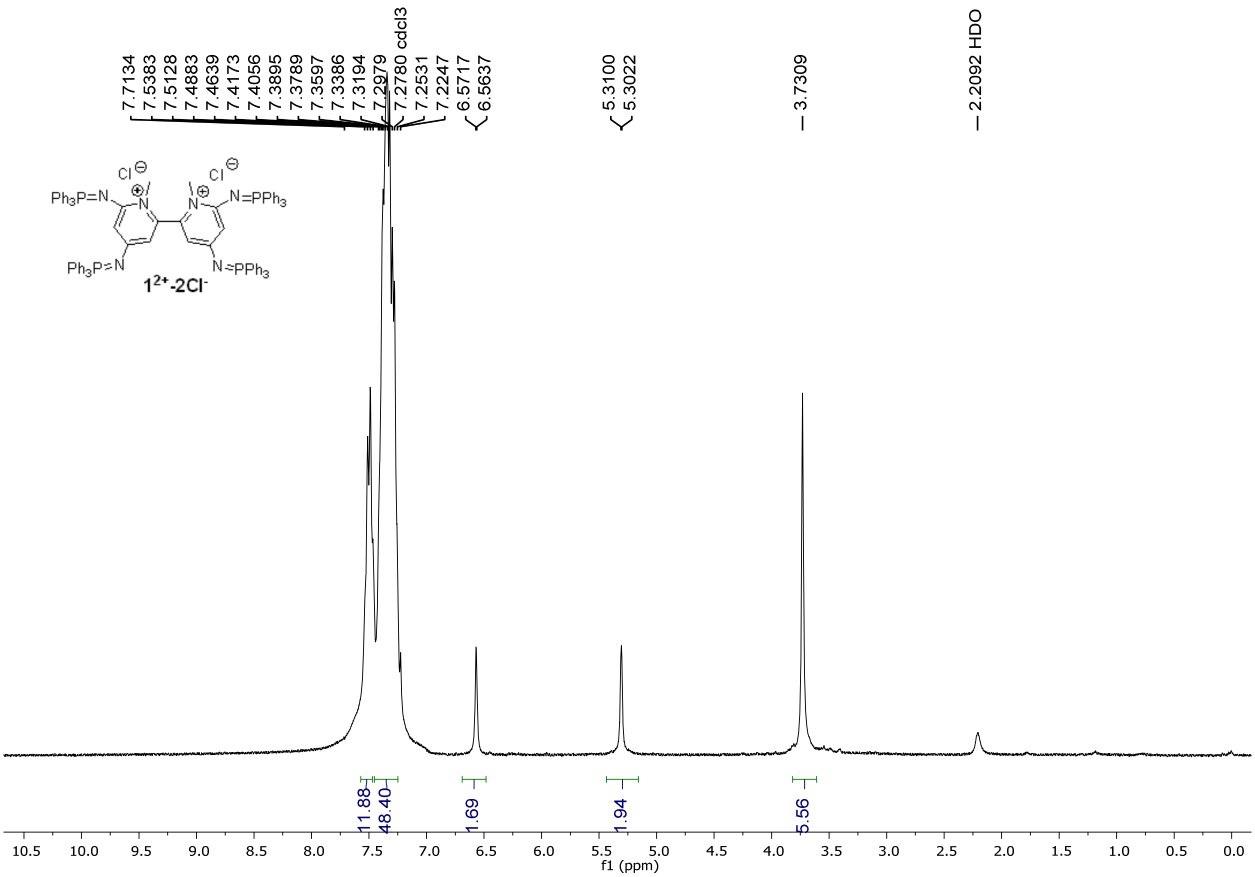
7.7134
7.5383
7.5128
7.4883
7.4639
7.4173
7.4056
7.3895
7.3789
7.3597
7.3386
7.3194
7.2979
7.2780 cdcl3
7.2531
7.2247
6.5717
6.5637
5.3100
5.3022
3.7309
2.2092 HDO
Ph3P=N
Cl
N
Ph3P=N
N=PPh3
N=PPh3
1[2+]-2Cl[-]
11.88
48.40
1.69
1.94
5.56
10.5
10.0
9.5
9.0
8.5
8.0
7.5
7.0
6.5
6.0
5.5
5.0
4.5
4.0
3.5
3.0
2.5
2.0
1.5
1.0
0.5
0.0
f1 (ppm)

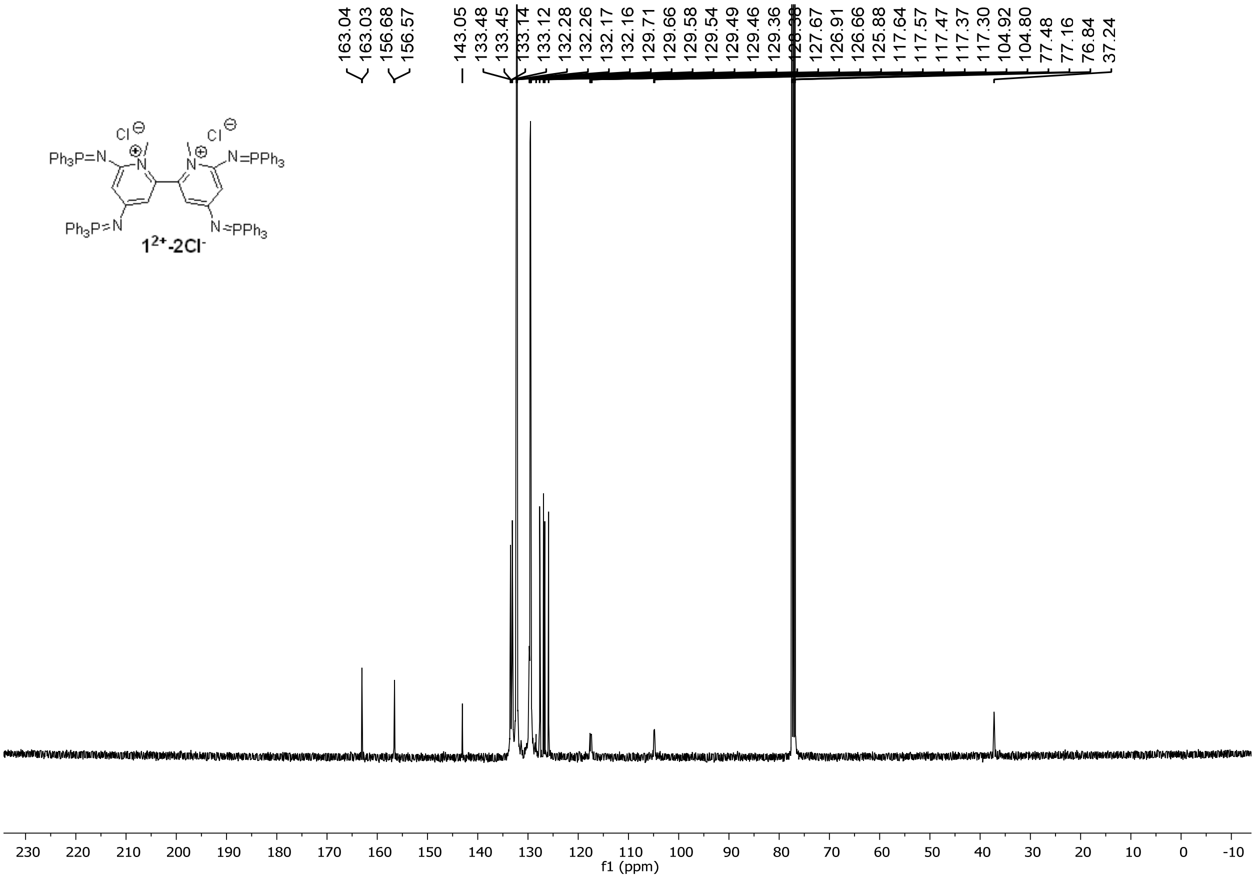
1²⁺-2Cl⁻
163.04
163.03
156.68
156.57
143.05
133.48
133.45
133.14
133.12
132.28
132.26
132.17
132.16
129.71
129.66
129.58
129.54
129.49
129.46
129.36
127.67
126.91
126.66
125.88
117.64
117.57
117.47
117.37
117.30
104.92
104.80
77.48
77.16
76.84
37.24
230 220 210 200 190 180 170 160 150 140 130 120 110 100 90 80 70 60 50 40 30 20 10 0 -10
f1 (ppm)

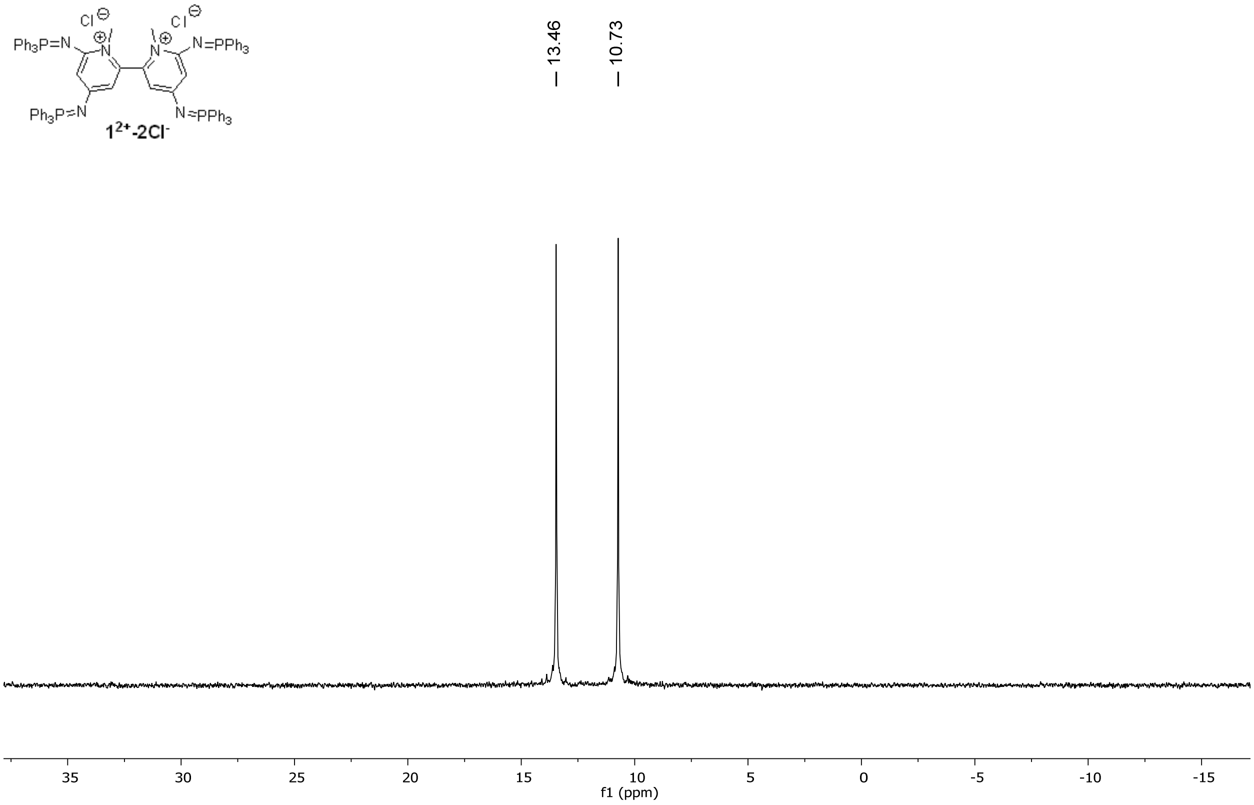
1²⁺-2Cl⁻
13.46
10.73
35 30 25 20 15 10 5 0 -5 -10 -15
f1 (ppm)

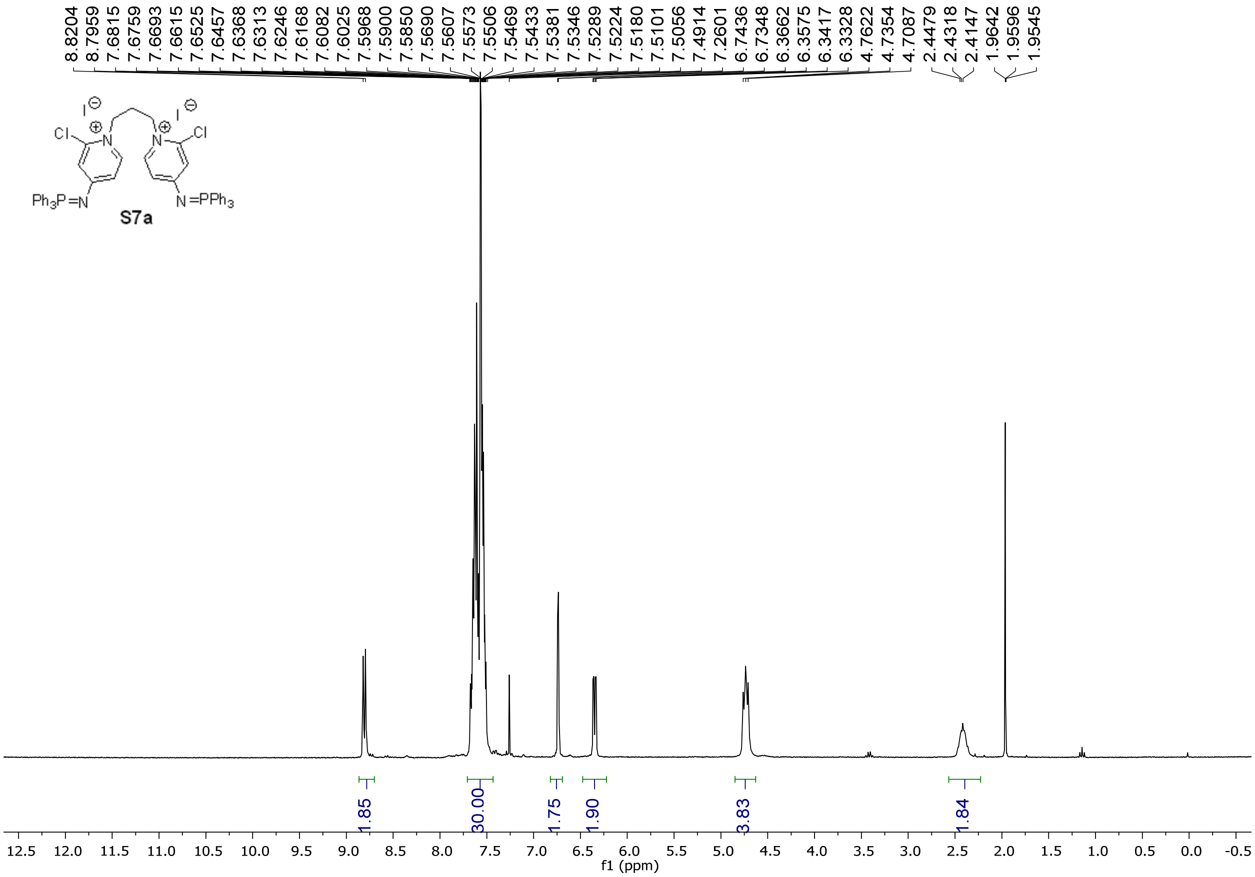
8.8204
8.7959
7.6815
7.6759
7.6693
7.6615
7.6525
7.6457
7.6368
7.6313
7.6246
7.6168
7.6082
7.6025
7.5968
7.5900
7.5850
7.5690
7.5607
7.5573
7.5506
7.5469
7.5433
7.5381
7.5346
7.5289
7.5224
7.5180
7.5101
7.5056
7.4914
7.2601
6.7436
6.7348
6.3662
6.3575
6.3417
6.3328
4.7622
4.7354
4.7087
2.4479
2.4318
2.4147
1.9642
1.9596
1.9545
S7a
1.85
30.00
1.75
1.90
3.83
1.84
f1 (ppm)

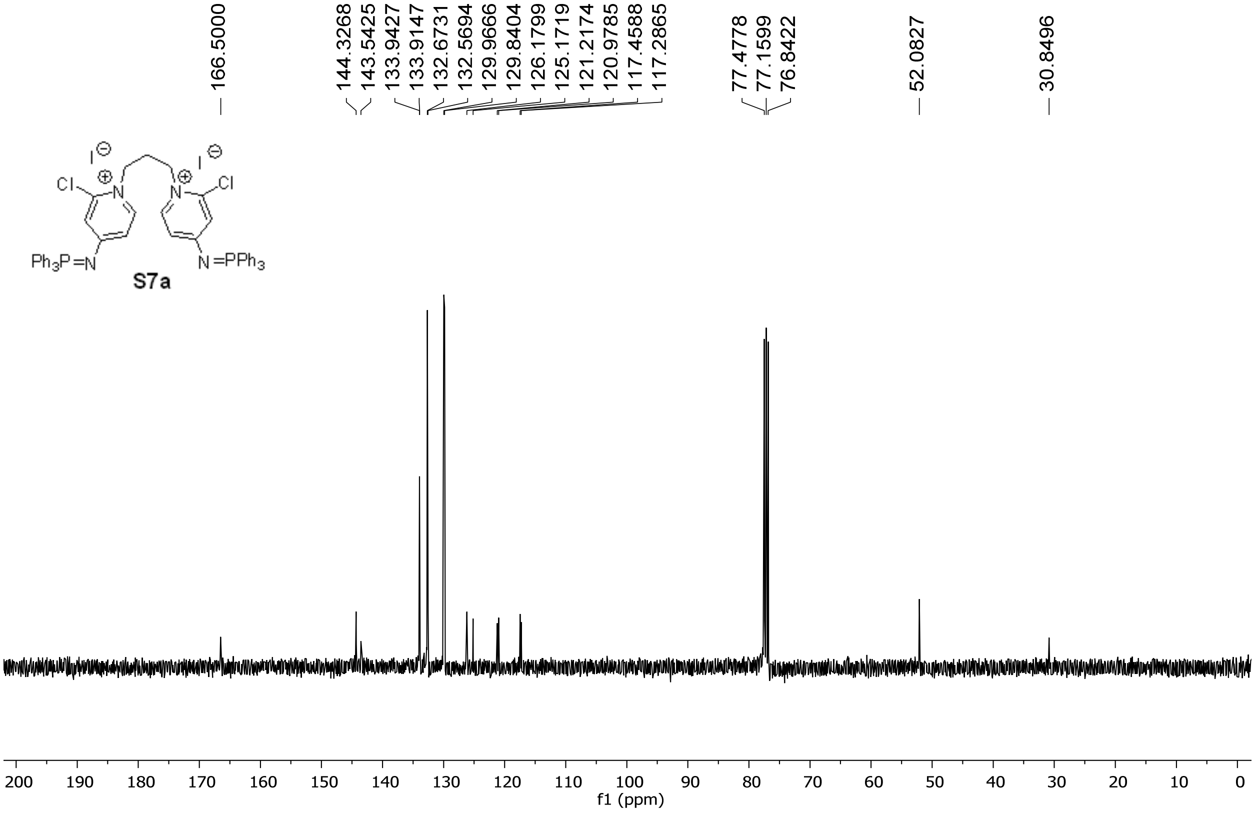
166.5000
144.3268
143.5425
133.9427
133.9147
132.6731
132.5694
129.9666
129.8404
126.1799
125.1719
121.2174
120.9785
117.4588
117.2865
77.4778
77.1599
76.8422
52.0827
30.8496
S7a
f1 (ppm)

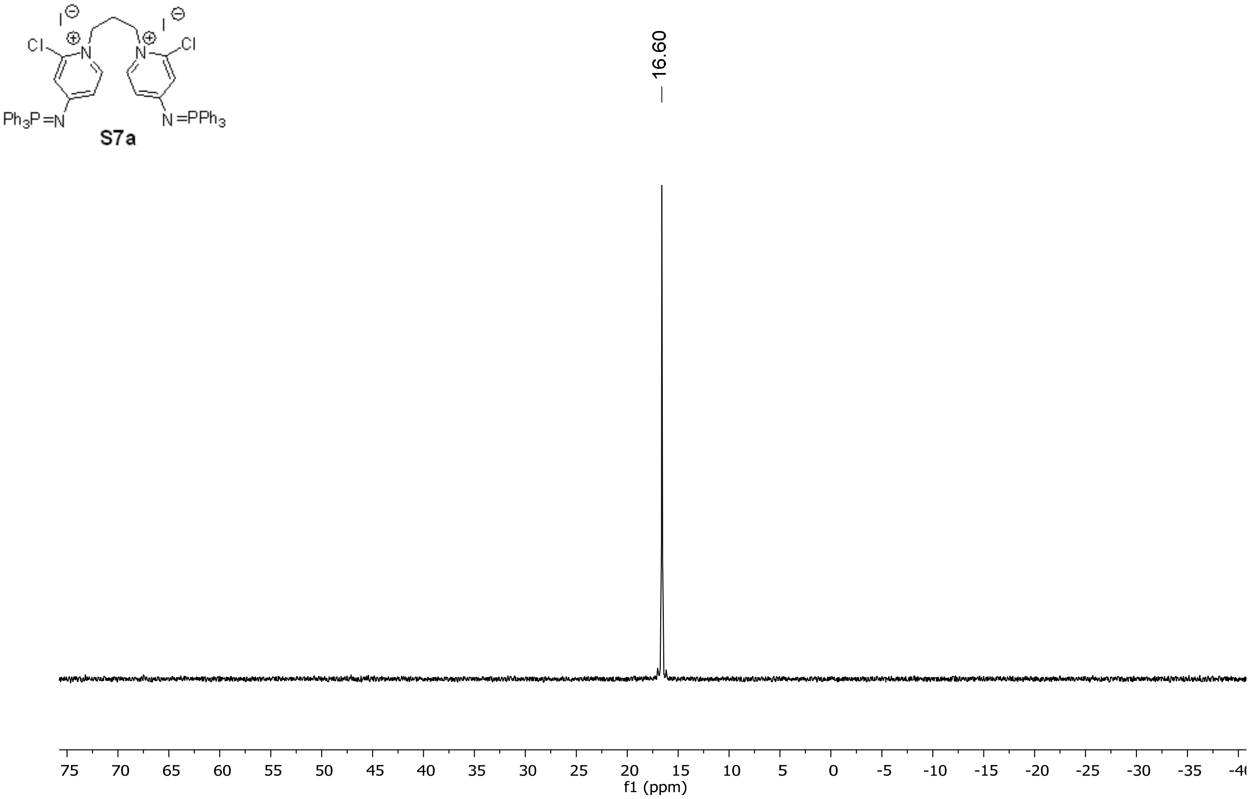
S7a
16.60
f1 (ppm)
75 70 65 60 55 50 45 40 35 30 25 20 15 10 5 0 -5 -10 -15 -20 -25 -30 -35

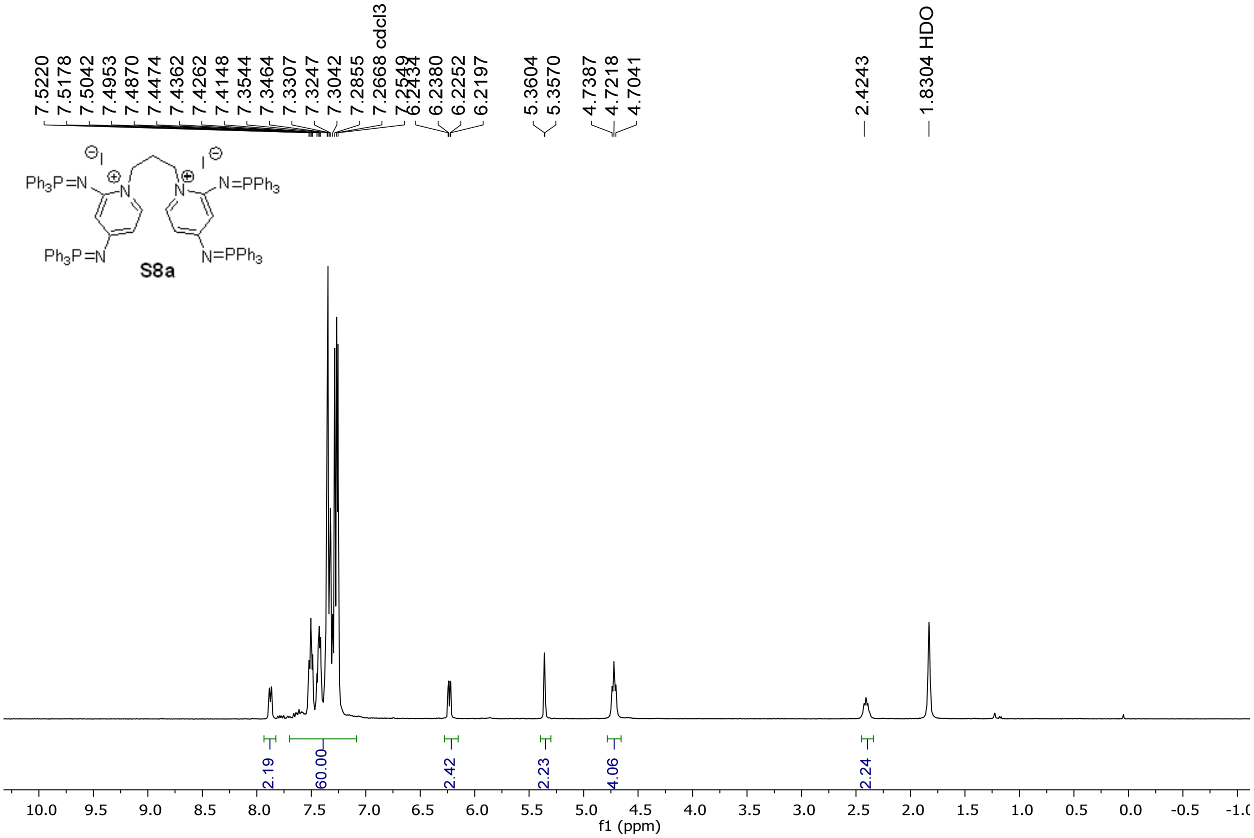
S8a
7.5220 7.5178 7.5042 7.4953 7.4870 7.4474 7.4362 7.4262 7.4148 7.3544 7.3464 7.3307 7.3247 7.3042 7.2855 7.2668 cdcl3 7.2549 6.2434 6.2380 6.2252 6.2197
5.3604 5.3570
4.7387 4.7218 4.7041
2.4243
1.8304 HDO
2.19 60.00 2.42 2.23 4.06 2.24
f1 (ppm)
10.0 9.5 9.0 8.5 8.0 7.5 7.0 6.5 6.0 5.5 5.0 4.5 4.0 3.5 3.0 2.5 2.0 1.5 1.0 0.5 0.0 -0.5

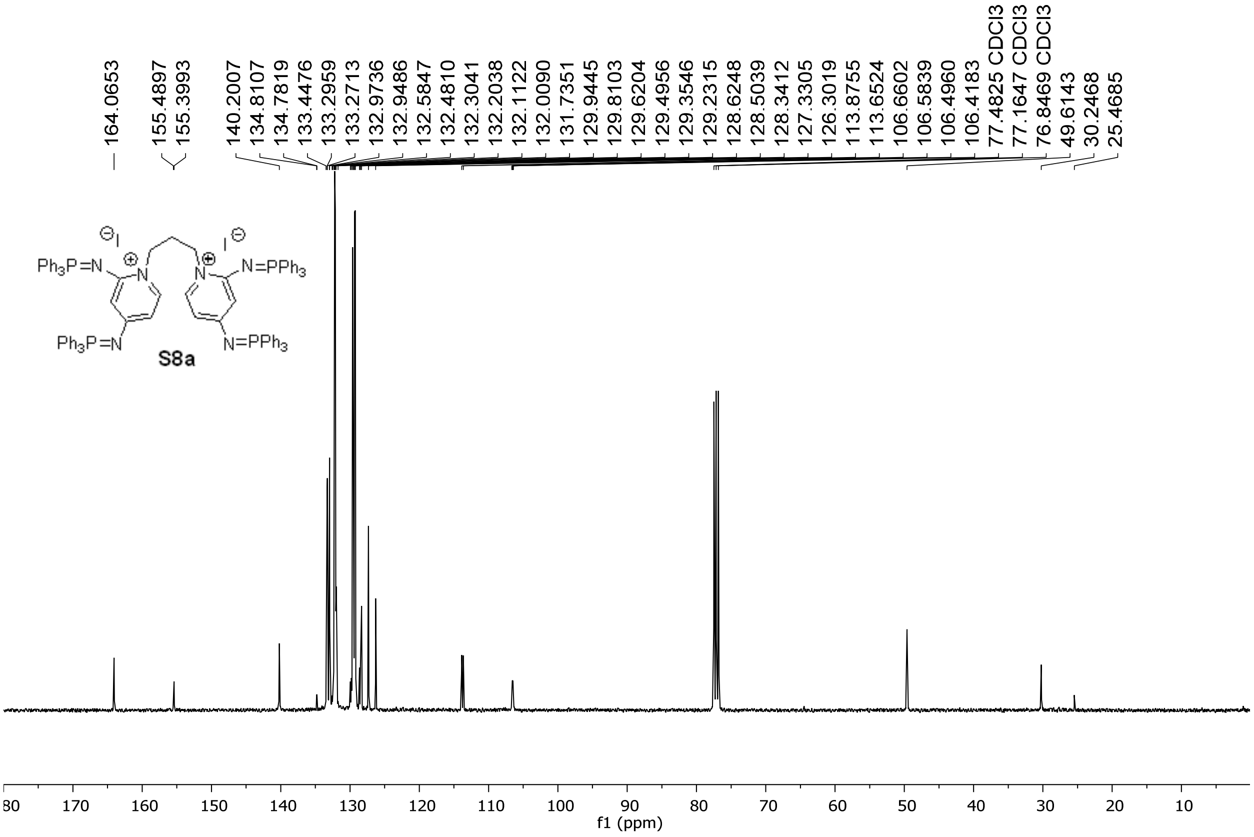

164.0653
155.4897
155.3993
140.2007
134.8107
134.7819
133.4476
133.2959
133.2713
132.9736
132.9486
132.5847
132.4810
132.3041
132.2038
132.1122
132.0090
131.7351
129.9445
129.8103
129.6204
129.4956
129.3546
129.2315
128.6248
128.5039
128.3412
127.3305
126.3019
113.8755
113.6524
106.6602
106.5839
106.4960
106.4183
77.4825 CDCl3
77.1647 CDCl3
76.8469 CDCl3
49.6143
30.2468
25.4685
Ph3P=N
N=PPh3
Ph3P=N
N=PPh3
S8a
80 170 160 150 140 130 120 110 100 90 80 70 60 50 40 30 20 10
f1 (ppm)

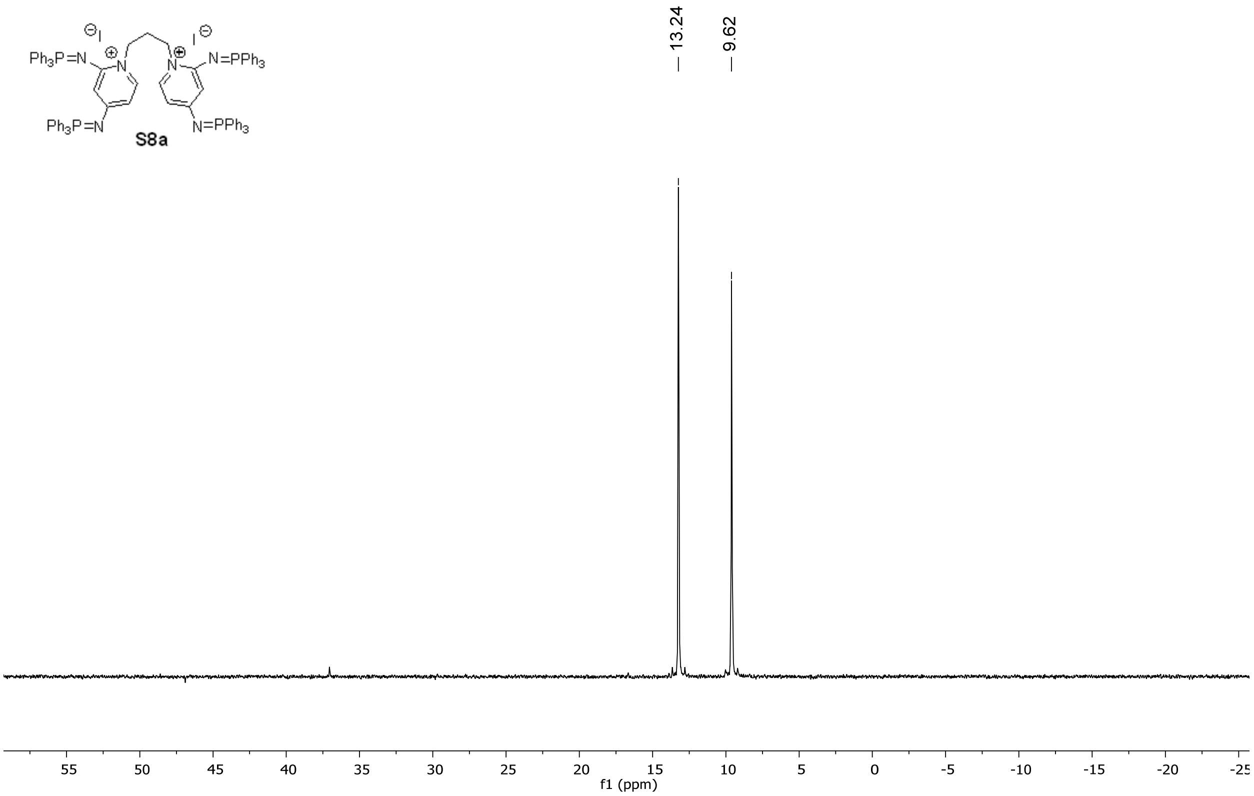

Ph3P=N
N=PPh3
I
S8a
13.24
9.62
f1 (ppm)
55 50 45 40 35 30 25 20 15 10 5 0 -5 -10 -15 -20 -25

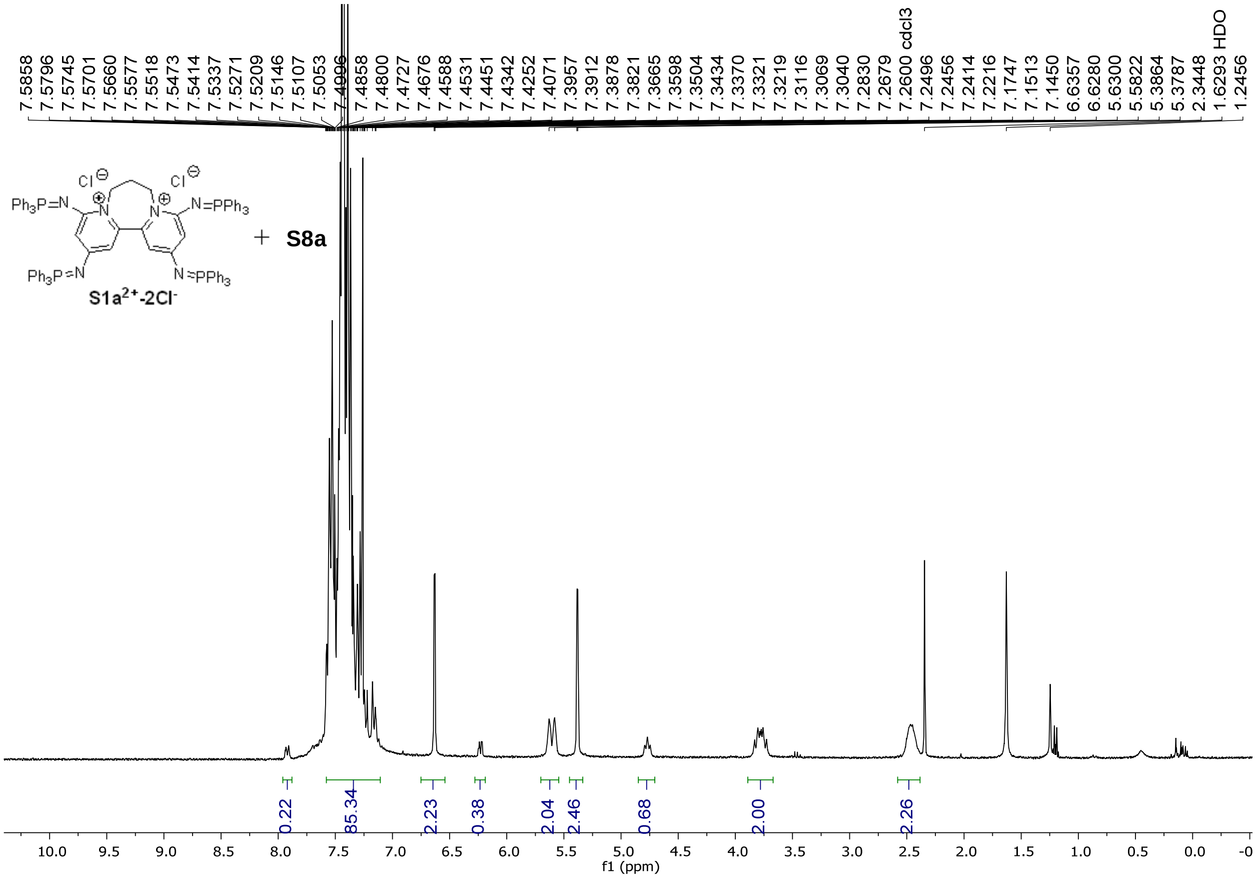

7.5858
7.5796
7.5745
7.5701
7.5660
7.5577
7.5518
7.5473
7.5414
7.5337
7.5271
7.5209
7.5146
7.5107
7.5053
7.4996
7.4858
7.4800
7.4727
7.4676
7.4588
7.4531
7.4451
7.4342
7.4252
7.4071
7.3957
7.3912
7.3878
7.3821
7.3665
7.3598
7.3504
7.3434
7.3370
7.3321
7.3219
7.3116
7.3069
7.3040
7.2830
7.2679
7.2600 cdcl3
7.2496
7.2456
7.2414
7.2216
7.1747
7.1513
7.1450
6.6357
6.6280
5.6300
5.5822
5.3864
5.3787
2.3448
1.6293 HDO
1.2456
Ph3P=N
N=PPh3
Cl
S1a2+-2Cl-
+ S8a
0.22
85.34
2.23
0.38
2.04
2.46
0.68
2.00
2.26
10.0 9.5 9.0 8.5 8.0 7.5 7.0 6.5 6.0 5.5 5.0 4.5 4.0 3.5 3.0 2.5 2.0 1.5 1.0 0.5 0.0 -0
f1 (ppm)

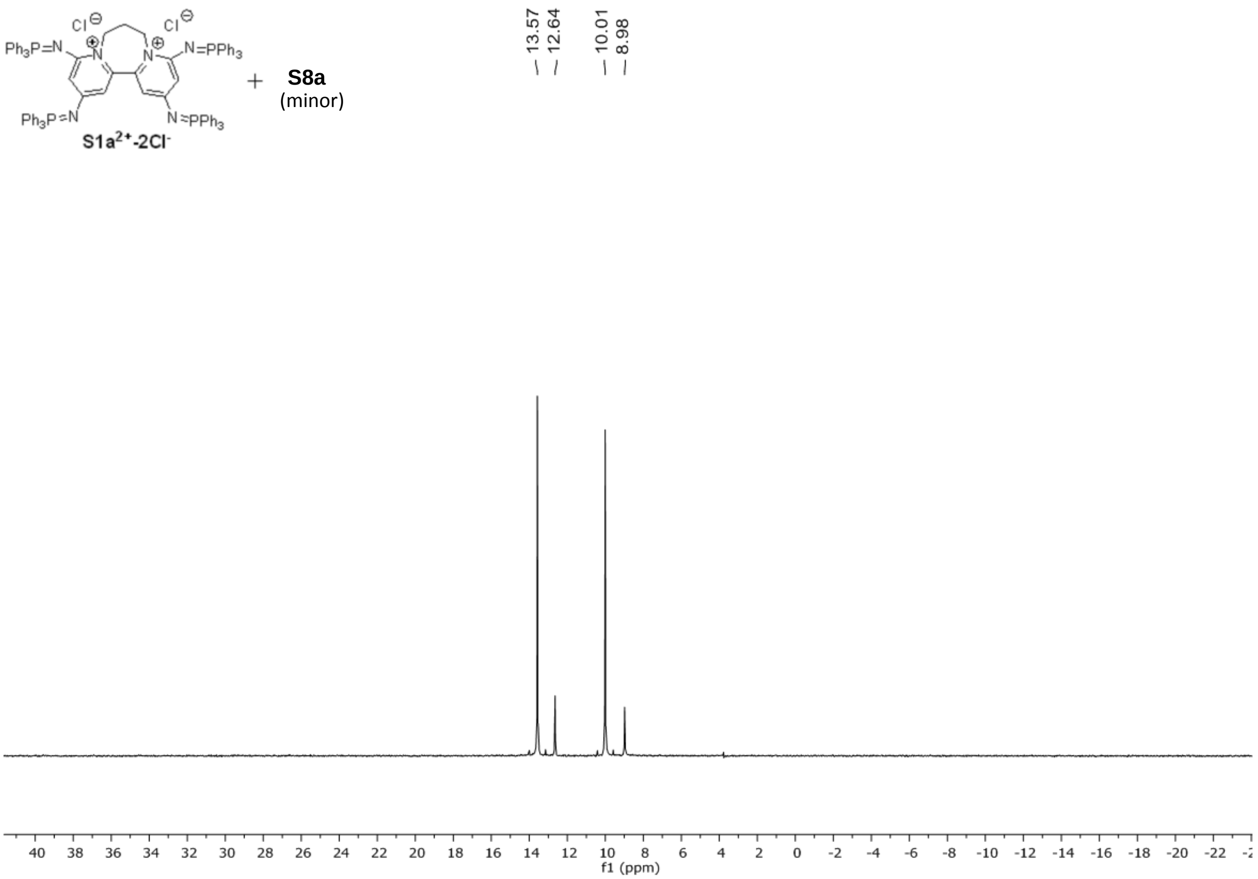
Ph3P=N
Cl⊖
Cl⊖
N=PPh3
Ph3P=N
N=PPh3
S1a2+-2Cl-
+ S8a
(minor)
13.57
12.64
10.01
8.98
40 38 36 34 32 30 28 26 24 22 20 18 16 14 12 10 8 6 4 2 0 -2 -4 -6 -8 -10 -12 -14 -16 -18 -20 -22
f1 (ppm)

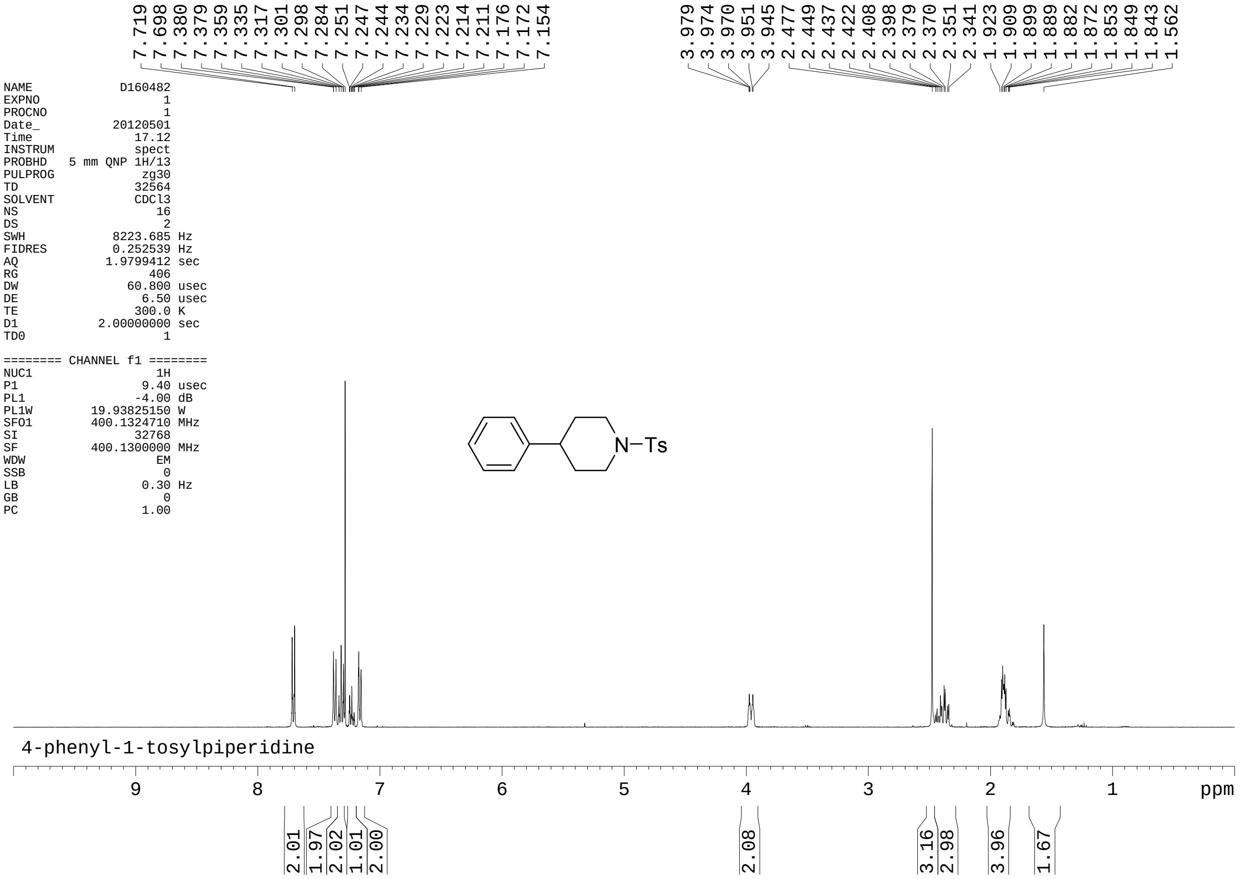
NAME D160482
EXPNO 1
PROCNO 1
Date_ 20120501
Time 17.12
INSTRUM spect
PROBHD 5 mm QNP 1H/13
PULPROG zg30
TD 32564
SOLVENT CDCl3
NS 16
DS 2
SWH 8223.685 Hz
FIDRES 0.252539 Hz
AQ 1.9799412 sec
RG 406
DW 60.800 usec
DE 6.50 usec
TE 300.0 K
D1 2.00000000 sec
TD0 1
======== CHANNEL f1 ========
NUC1 1H
P1 9.40 usec
PL1 -4.00 dB
PL1W 19.93825150 W
SFO1 400.1324710 MHz
SI 32768
SF 400.1300000 MHz
WDW EM
SSB 0
LB 0.30 Hz
GB 0
PC 1.00
7.719 7.698 7.380 7.379 7.359 7.335 7.317 7.301 7.298 7.284 7.251 7.247 7.244 7.234 7.229 7.223 7.214 7.211 7.176 7.172 7.154
3.979 3.974 3.970 3.951 3.945 2.477 2.449 2.437 2.422 2.408 2.398 2.379 2.370 2.351 2.341 1.923 1.909 1.899 1.882 1.872 1.853 1.849 1.843 1.562
N–Ts
4-phenyl-1-tosylpiperidine
9 8 7 6 5 4 3 2 1 ppm
2.01 1.97 2.02 1.01 2.00 2.08 3.16 2.98 3.96 1.67

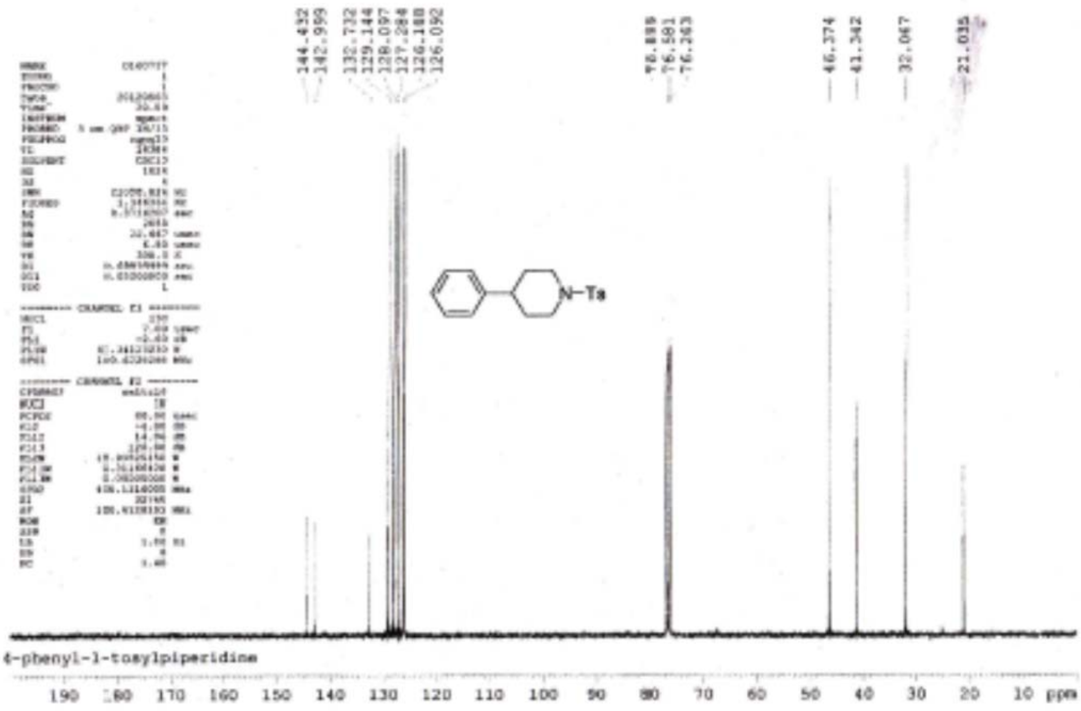
144.432
142.999
132.732
129.144
128.097
127.284
126.188
126.092
76.899
76.581
76.263
46.374
41.342
32.067
21.035
N–Ts
4-phenyl-1-tosylpiperidine
190
180
170
160
150
140
130
120
110
100
90
80
70
60
50
40
30
20
10 ppm

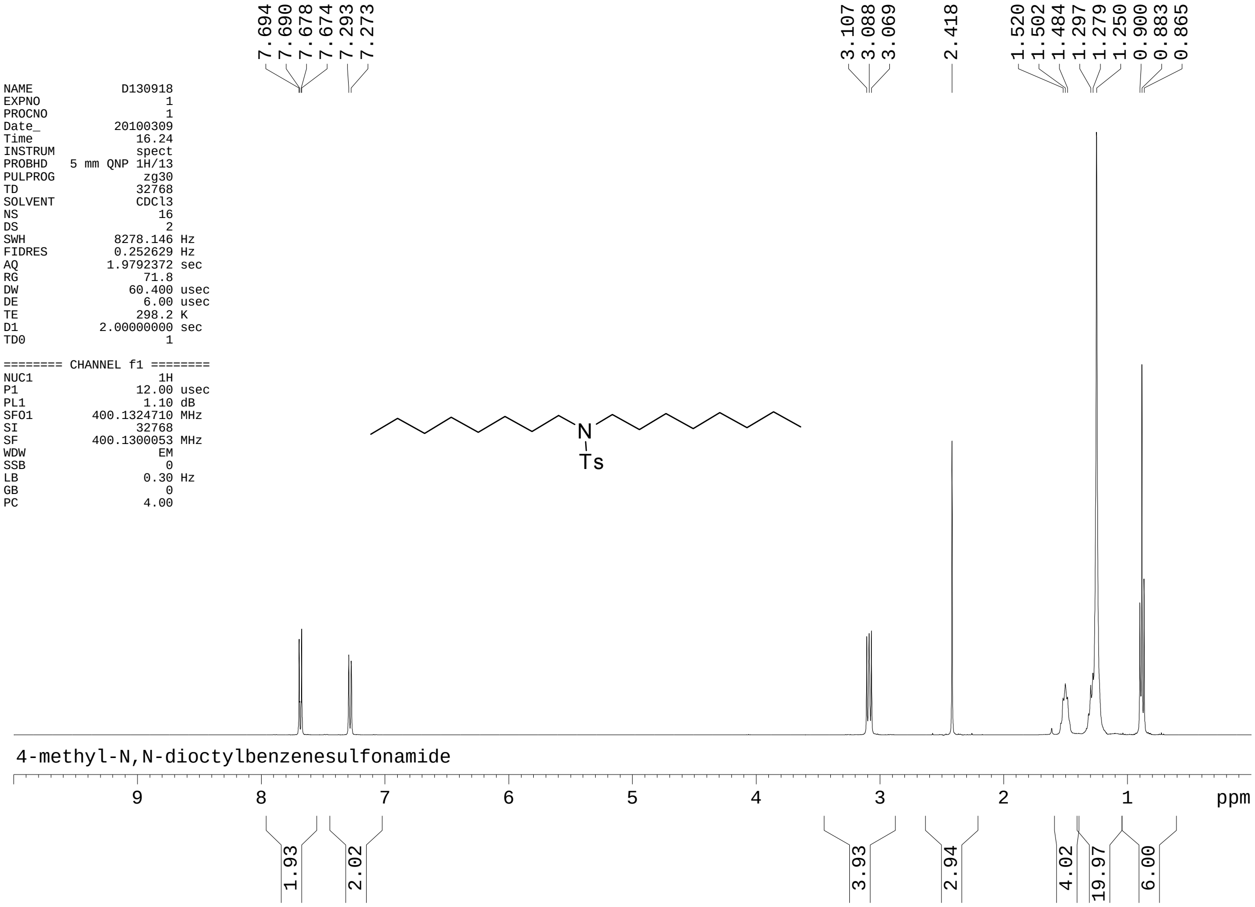

4-methyl-N,N-dioctylbenzenesulfonamide

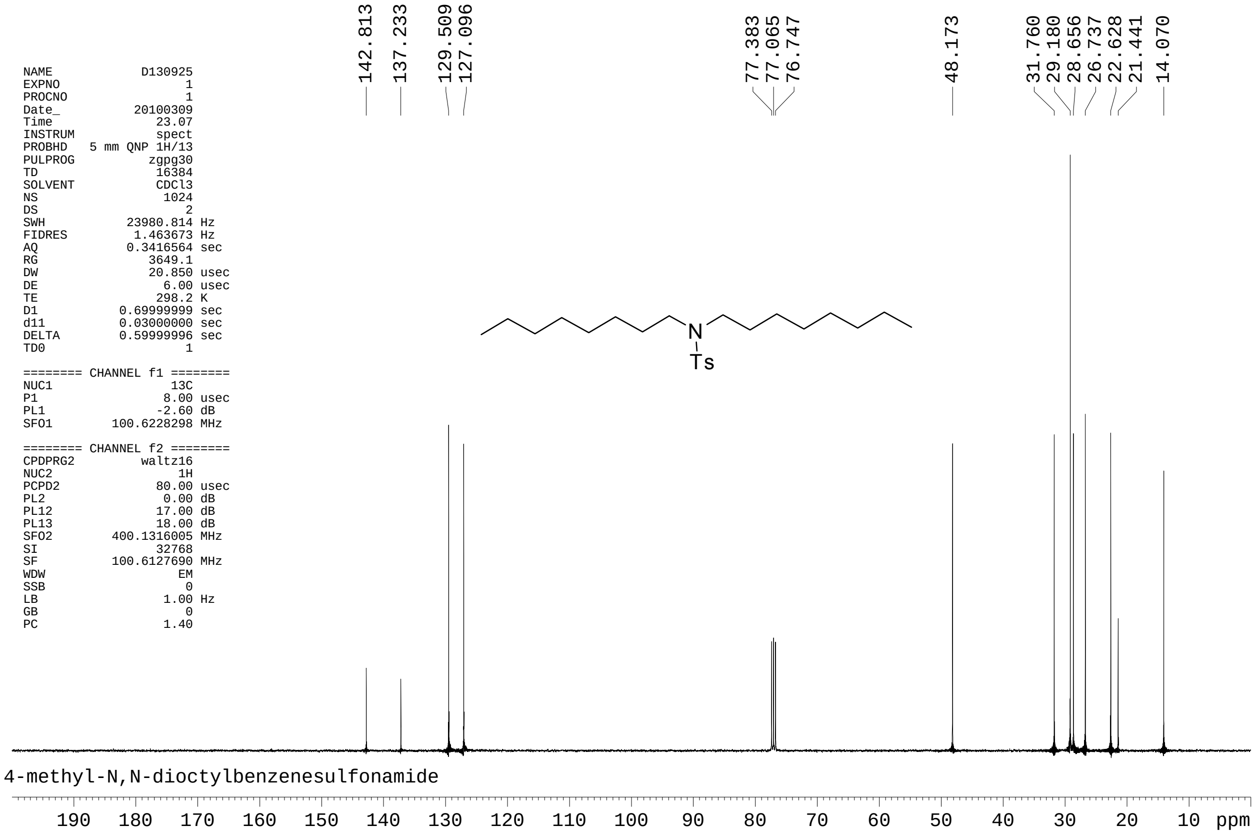
NAME D130925
EXPNO 1
PROCNO 1
Date_ 20100309
Time 23.07
INSTRUM spect
PROBHD 5 mm QNP 1H/13
PULPROG zgpg30
TD 16384
SOLVENT CDCl3
NS 1024
DS 2
SWH 23980.814 Hz
FIDRES 1.463673 Hz
AQ 0.3416564 sec
RG 3649.1
DW 20.850 usec
DE 6.00 usec
TE 298.2 K
D1 0.69999999 sec
d11 0.03000000 sec
DELTA 0.59999996 sec
TD0 1
======== CHANNEL f1 ========
NUC1 13C
P1 8.00 usec
PL1 -2.60 dB
SFO1 100.6228298 MHz
======== CHANNEL f2 ========
CPDPRG2 waltz16
NUC2 1H
PCPD2 80.00 usec
PL2 0.00 dB
PL12 17.00 dB
PL13 18.00 dB
SFO2 400.1316005 MHz
SI 32768
SF 100.6127690 MHz
WDW EM
SSB 0
LB 1.00 Hz
GB 0
PC 1.40
142.813
137.233
129.509
127.096
77.383
77.065
76.747
48.173
31.760
29.180
28.656
26.737
22.628
21.441
14.070
N
Ts
4-methyl-N,N-dioctylbenzenesulfonamide
190 180 170 160 150 140 130 120 110 100 90 80 70 60 50 40 30 20 10 ppm

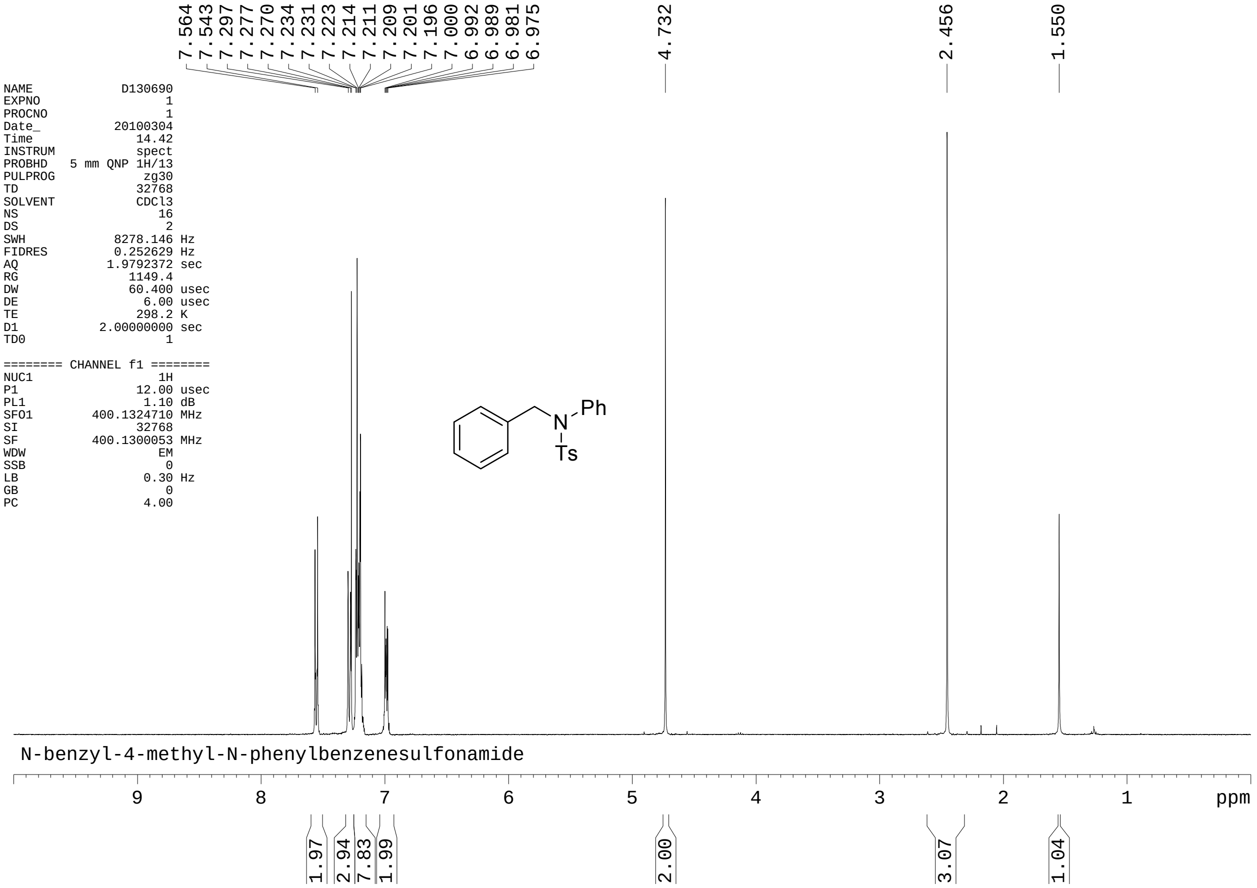
7.564 7.543 7.297 7.277 7.270 7.234 7.231 7.223 7.214 7.211 7.209 7.201 7.196 7.000 6.992 6.989 6.981 6.975
4.732
2.456
1.550
NAME D130690
EXPNO 1
PROCNO 1
Date_ 20100304
Time 14.42
INSTRUM spect
PROBHD 5 mm QNP 1H/13
PULPROG zg30
TD 32768
SOLVENT CDCl3
NS 16
DS 2
SWH 8278.146 Hz
FIDRES 0.252629 Hz
AQ 1.9792372 sec
RG 1149.4
DW 60.400 usec
DE 6.00 usec
TE 298.2 K
D1 2.00000000 sec
TD0 1
======== CHANNEL f1 ========
NUC1 1H
P1 12.00 usec
PL1 1.10 dB
SFO1 400.1324710 MHz
SI 32768
SF 400.1300053 MHz
WDW EM
SSB 0
LB 0.30 Hz
GB 0
PC 4.00
N
Ph
Ts
N-benzyl-4-methyl-N-phenylbenzenesulfonamide
9 8 7 6 5 4 3 2 1 ppm
1.97 2.94 7.83 1.99 2.00 3.07 1.04

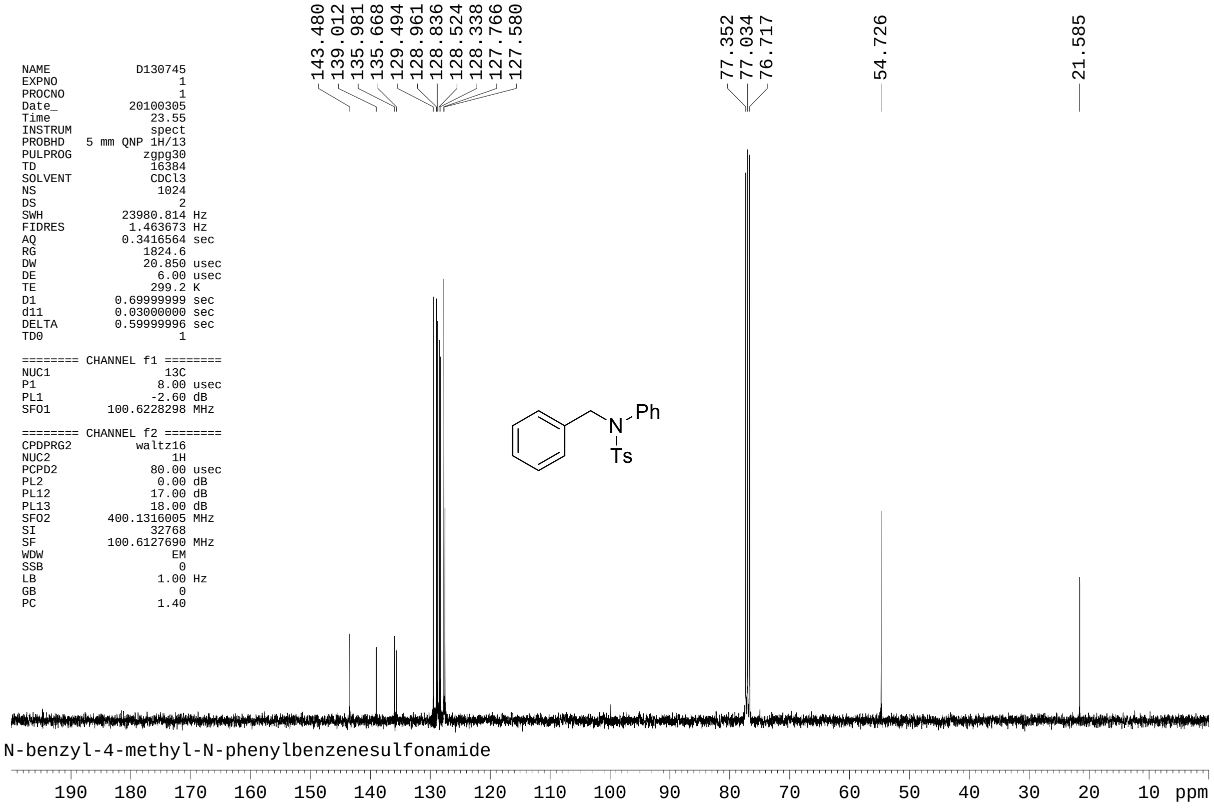
143.480 139.012 135.981 135.668 129.494 128.961 128.836 128.524 128.338 127.766 127.580
77.352 77.034 76.717
54.726
21.585
NAME D130745
EXPNO 1
PROCNO 1
Date_ 20100305
Time 23.55
INSTRUM spect
PROBHD 5 mm QNP 1H/13
PULPROG zgpg30
TD 16384
SOLVENT CDCl3
NS 1024
DS 2
SWH 23980.814 Hz
FIDRES 1.463673 Hz
AQ 0.3416564 sec
RG 1824.6
DW 20.850 usec
DE 6.00 usec
TE 299.2 K
D1 0.69999999 sec
d11 0.03000000 sec
DELTA 0.59999996 sec
TD0 1
======== CHANNEL f1 ========
NUC1 13C
P1 8.00 usec
PL1 -2.60 dB
SFO1 100.6228298 MHz
======== CHANNEL f2 ========
CPDPRG2 waltz16
NUC2 1H
PCPD2 80.00 usec
PL2 0.00 dB
PL12 17.00 dB
PL13 18.00 dB
SFO2 400.1316005 MHz
SI 32768
SF 100.6127690 MHz
WDW EM
SSB 0
LB 1.00 Hz
GB 0
PC 1.40
N
Ph
Ts
N-benzyl-4-methyl-N-phenylbenzenesulfonamide
190 180 170 160 150 140 130 120 110 100 90 80 70 60 50 40 30 20 10 ppm

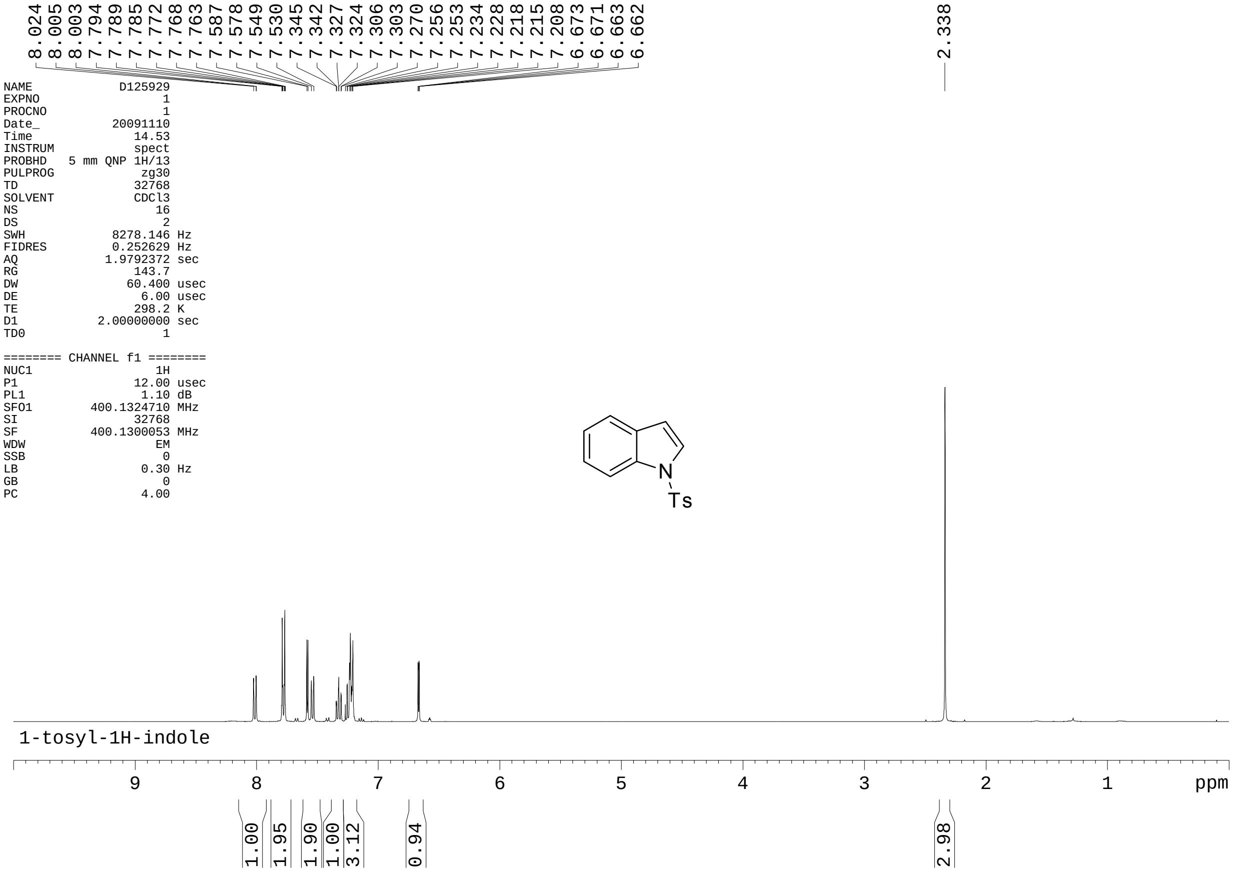

8.024 8.005 8.003 7.794 7.789 7.785 7.772 7.768 7.763 7.587 7.578 7.549 7.530 7.345 7.342 7.327 7.324 7.306 7.303 7.270 7.256 7.253 7.234 7.228 7.218 7.215 7.208 6.673 6.671 6.663 6.662
2.338
NAME D125929
EXPNO 1
PROCNO 1
Date_ 20091110
Time 14.53
INSTRUM spect
PROBHD 5 mm QNP 1H/13
PULPROG zg30
TD 32768
SOLVENT CDCl3
NS 16
DS 2
SWH 8278.146 Hz
FIDRES 0.252629 Hz
AQ 1.9792372 sec
RG 143.7
DW 60.400 usec
DE 6.00 usec
TE 298.2 K
D1 2.00000000 sec
TD0 1
======== CHANNEL f1 ========
NUC1 1H
P1 12.00 usec
PL1 1.10 dB
SFO1 400.1324710 MHz
SI 32768
SF 400.1300053 MHz
WDW EM
SSB 0
LB 0.30 Hz
GB 0
PC 4.00
N
Ts
1-tosyl-1H-indole
9 8 7 6 5 4 3 2 1 ppm
1.00 1.95 1.90 1.00 3.12 0.94 2.98

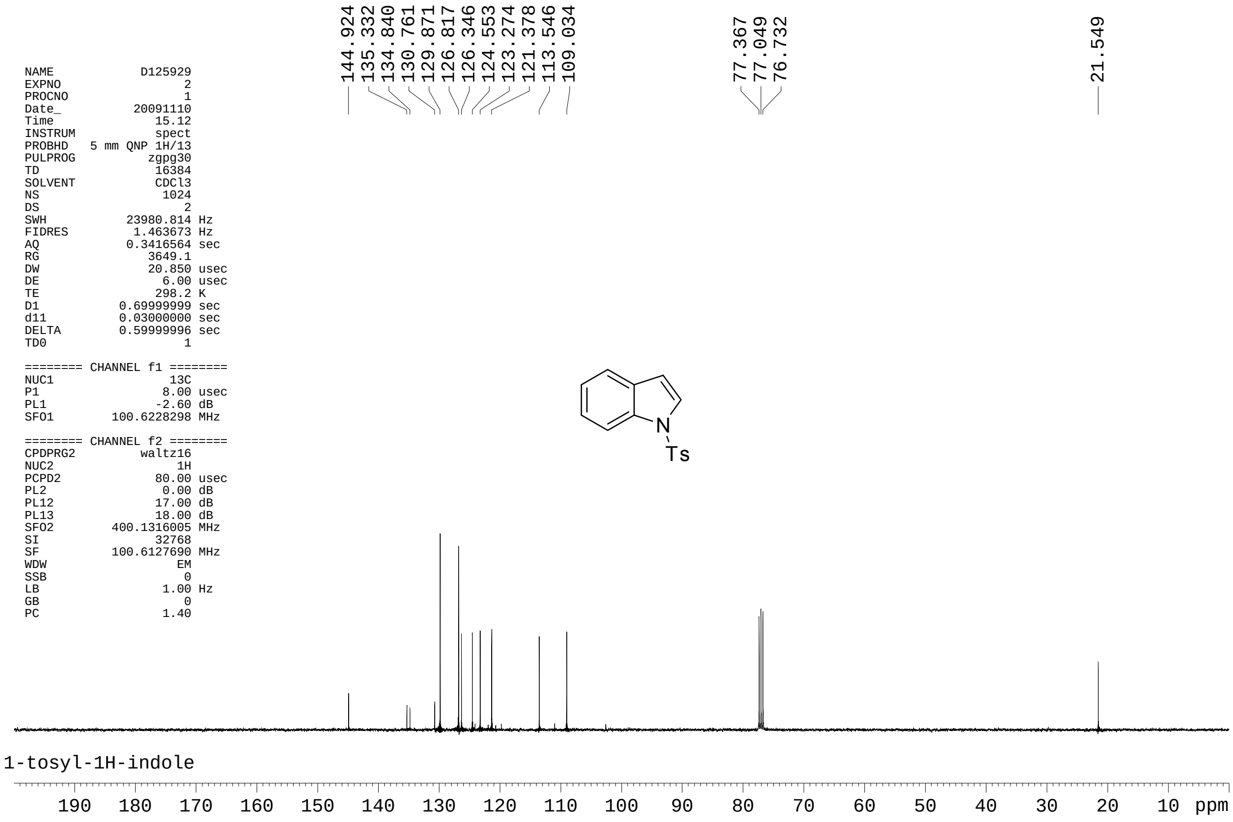

144.924 135.332 134.840 130.761 129.871 126.817 126.346 124.553 123.274 121.378 113.546 109.034
77.367 77.049 76.732
21.549
NAME D125929
EXPNO 2
PROCNO 1
Date_ 20091110
Time 15.12
INSTRUM spect
PROBHD 5 mm QNP 1H/13
PULPROG zgpg30
TD 16384
SOLVENT CDCl3
NS 1024
DS 2
SWH 23980.814 Hz
FIDRES 1.463673 Hz
AQ 0.3416564 sec
RG 3649.1
DW 20.850 usec
DE 6.00 usec
TE 298.2 K
D1 0.69999999 sec
d11 0.03000000 sec
DELTA 0.59999996 sec
TD0 1
======== CHANNEL f1 ========
NUC1 13C
P1 8.00 usec
PL1 -2.60 dB
SFO1 100.6228298 MHz
======== CHANNEL f2 ========
CPDPRG2 waltz16
NUC2 1H
PCPD2 80.00 usec
PL2 0.00 dB
PL12 17.00 dB
PL13 18.00 dB
SFO2 400.1316005 MHz
SI 32768
SF 100.6127690 MHz
WDW EM
SSB 0
LB 1.00 Hz
GB 0
PC 1.40
N
Ts
1-tosyl-1H-indole
190 180 170 160 150 140 130 120 110 100 90 80 70 60 50 40 30 20 10 ppm

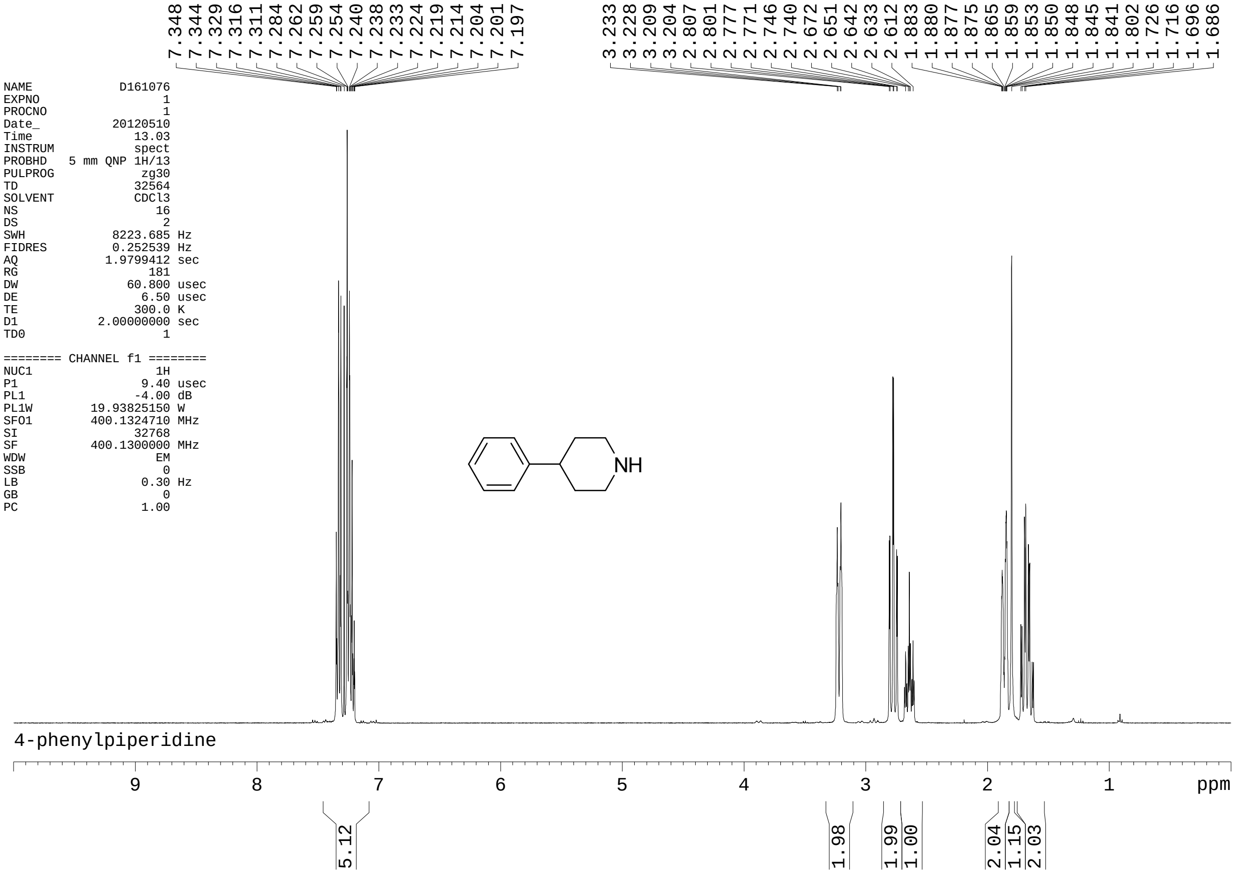
7.348 7.344 7.329 7.316 7.311 7.284 7.262 7.259 7.254 7.240 7.238 7.233 7.224 7.219 7.214 7.204 7.201 7.197
3.233 3.228 3.209 3.204 2.807 2.801 2.777 2.771 2.746 2.740 2.672 2.651 2.642 2.633 2.612 1.883 1.880 1.877 1.875 1.865 1.859 1.853 1.850 1.848 1.845 1.841 1.802 1.726 1.716 1.696 1.686
NAME D161076
EXPNO 1
PROCNO 1
Date_ 20120510
Time 13.03
INSTRUM spect
PROBHD 5 mm QNP 1H/13
PULPROG zg30
TD 32564
SOLVENT CDCl3
NS 16
DS 2
SWH 8223.685 Hz
FIDRES 0.252539 Hz
AQ 1.9799412 sec
RG 181
DW 60.800 usec
DE 6.50 usec
TE 300.0 K
D1 2.00000000 sec
TD0 1
======== CHANNEL f1 ========
NUC1 1H
P1 9.40 usec
PL1 -4.00 dB
PL1W 19.93825150 W
SFO1 400.1324710 MHz
SI 32768
SF 400.1300000 MHz
WDW EM
SSB 0
LB 0.30 Hz
GB 0
PC 1.00
NH
4-phenylpiperidine
9 8 7 6 5 4 3 2 1 ppm
5.12 1.98 1.99 1.00 2.04 1.15 2.03

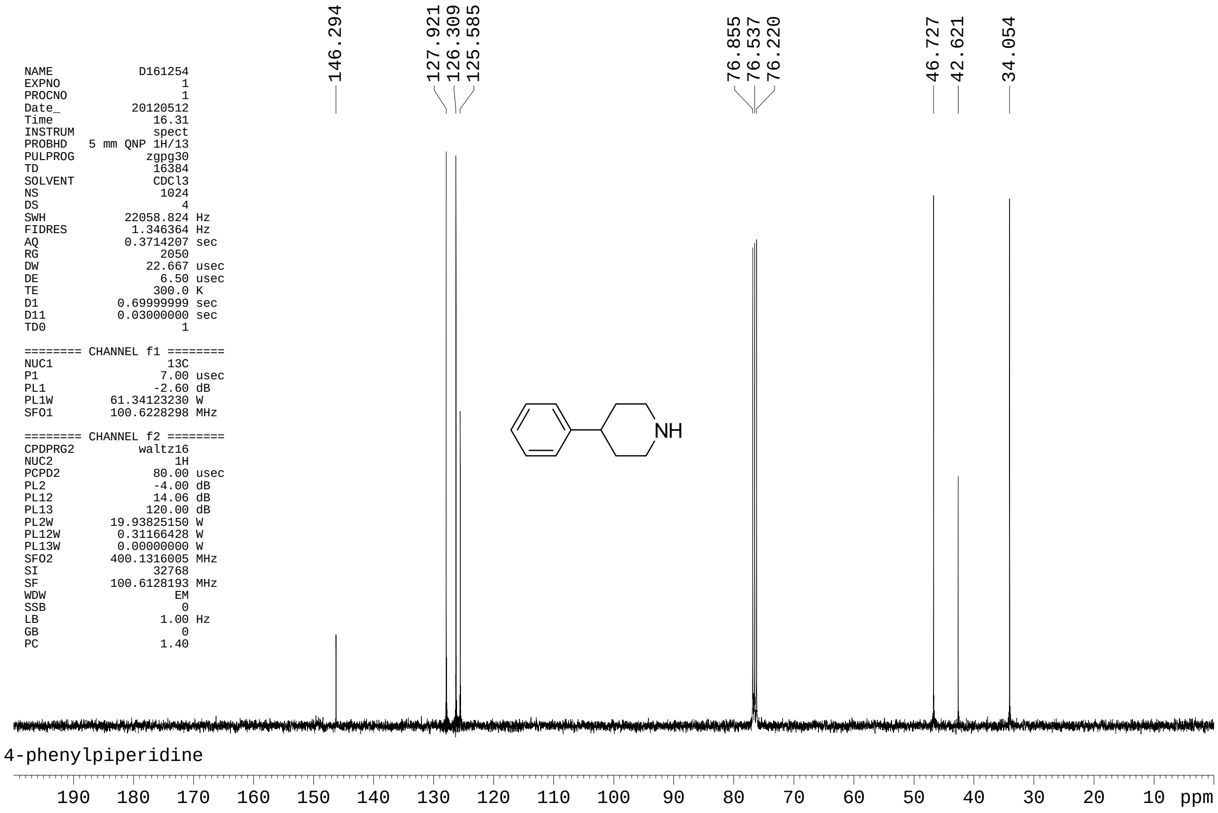
146.294 127.921 126.309 125.585 76.855 76.537 76.220 46.727 42.621 34.054
NAME D161254
EXPNO 1
PROCNO 1
Date_ 20120512
Time 16.31
INSTRUM spect
PROBHD 5 mm QNP 1H/13
PULPROG zgpg30
TD 16384
SOLVENT CDCl3
NS 1024
DS 4
SWH 22058.824 Hz
FIDRES 1.346364 Hz
AQ 0.3714207 sec
RG 2050
DW 22.667 usec
DE 6.50 usec
TE 300.0 K
D1 0.69999999 sec
D11 0.03000000 sec
TD0 1
======== CHANNEL f1 ========
NUC1 13C
P1 7.00 usec
PL1 -2.60 dB
PL1W 61.34123230 W
SFO1 100.6228298 MHz
======== CHANNEL f2 ========
CPDPRG2 waltz16
NUC2 1H
PCPD2 80.00 usec
PL2 -4.00 dB
PL12 14.06 dB
PL13 120.00 dB
PL2W 19.93825150 W
PL12W 0.31166428 W
PL13W 0.00000000 W
SFO2 400.1316005 MHz
SI 32768
SF 100.6128193 MHz
WDW EM
SSB 0
LB 1.00 Hz
GB 0
PC 1.40
NH
4-phenylpiperidine
190 180 170 160 150 140 130 120 110 100 90 80 70 60 50 40 30 20 10 ppm

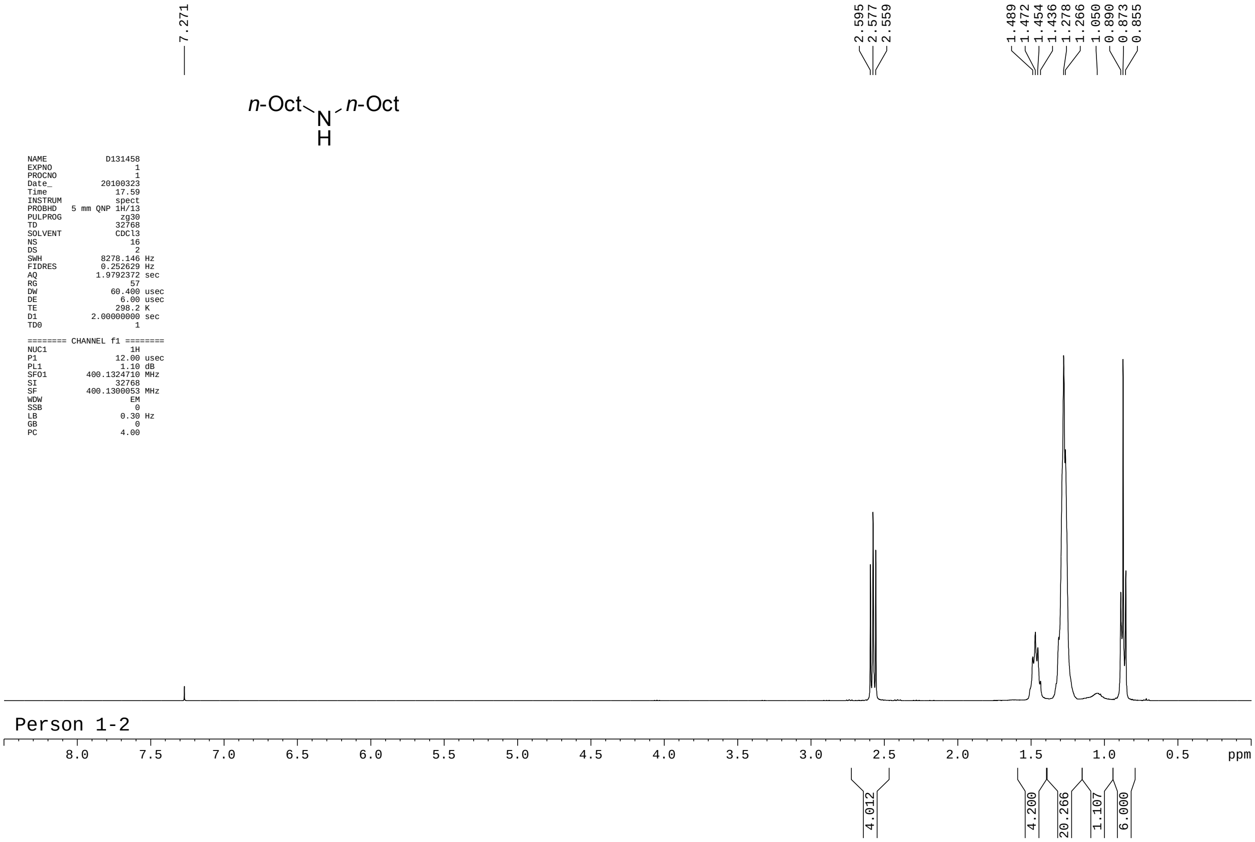
7.271
2.595
2.577
2.559
1.489
1.472
1.454
1.436
1.278
1.266
1.050
0.890
0.873
0.855
n-Oct N n-Oct
H
NAME D131458
EXPNO 1
PROCNO 1
Date_ 20100323
Time 17.59
INSTRUM spect
PROBHD 5 mm QNP 1H/13
PULPROG zg30
TD 32768
SOLVENT CDCl3
NS 16
DS 2
SWH 8278.146 Hz
FIDRES 0.252629 Hz
AQ 1.9792372 sec
RG 57
DW 60.400 usec
DE 6.00 usec
TE 298.2 K
D1 2.00000000 sec
TD0 1
======== CHANNEL f1 ========
NUC1 1H
P1 12.00 usec
PL1 1.10 dB
SFO1 400.1324710 MHz
SI 32768
SF 400.1300053 MHz
WDW EM
SSB 0
LB 0.30 Hz
GB 0
PC 4.00
Person 1-2
8.0 7.5 7.0 6.5 6.0 5.5 5.0 4.5 4.0 3.5 3.0 2.5 2.0 1.5 1.0 0.5 ppm
4.012
4.200
20.266
1.107
6.000

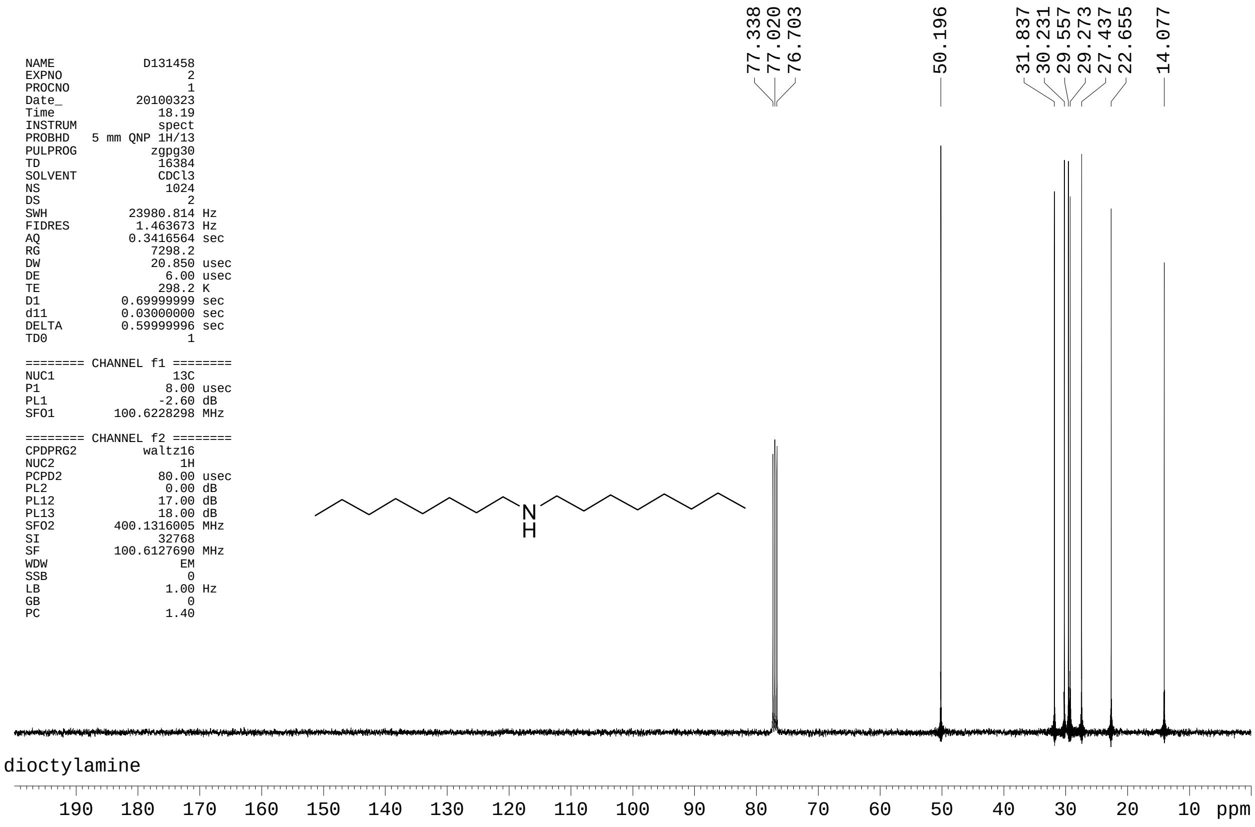
77.338
77.020
76.703
50.196
31.837
30.231
29.557
29.273
27.437
22.655
14.077
NAME D131458
EXPNO 2
PROCNO 1
Date_ 20100323
Time 18.19
INSTRUM spect
PROBHD 5 mm QNP 1H/13
PULPROG zgpg30
TD 16384
SOLVENT CDCl3
NS 1024
DS 2
SWH 23980.814 Hz
FIDRES 1.463673 Hz
AQ 0.3416564 sec
RG 7298.2
DW 20.850 usec
DE 6.00 usec
TE 298.2 K
D1 0.69999999 sec
d11 0.03000000 sec
DELTA 0.59999996 sec
TD0 1
======== CHANNEL f1 ========
NUC1 13C
P1 8.00 usec
PL1 -2.60 dB
SFO1 100.6228298 MHz
======== CHANNEL f2 ========
CPDPRG2 waltz16
NUC2 1H
PCPD2 80.00 usec
PL2 0.00 dB
PL12 17.00 dB
PL13 18.00 dB
SFO2 400.1316005 MHz
SI 32768
SF 100.6127690 MHz
WDW EM
SSB 0
LB 1.00 Hz
GB 0
PC 1.40
N
H
dioctylamine
190 180 170 160 150 140 130 120 110 100 90 80 70 60 50 40 30 20 10 ppm

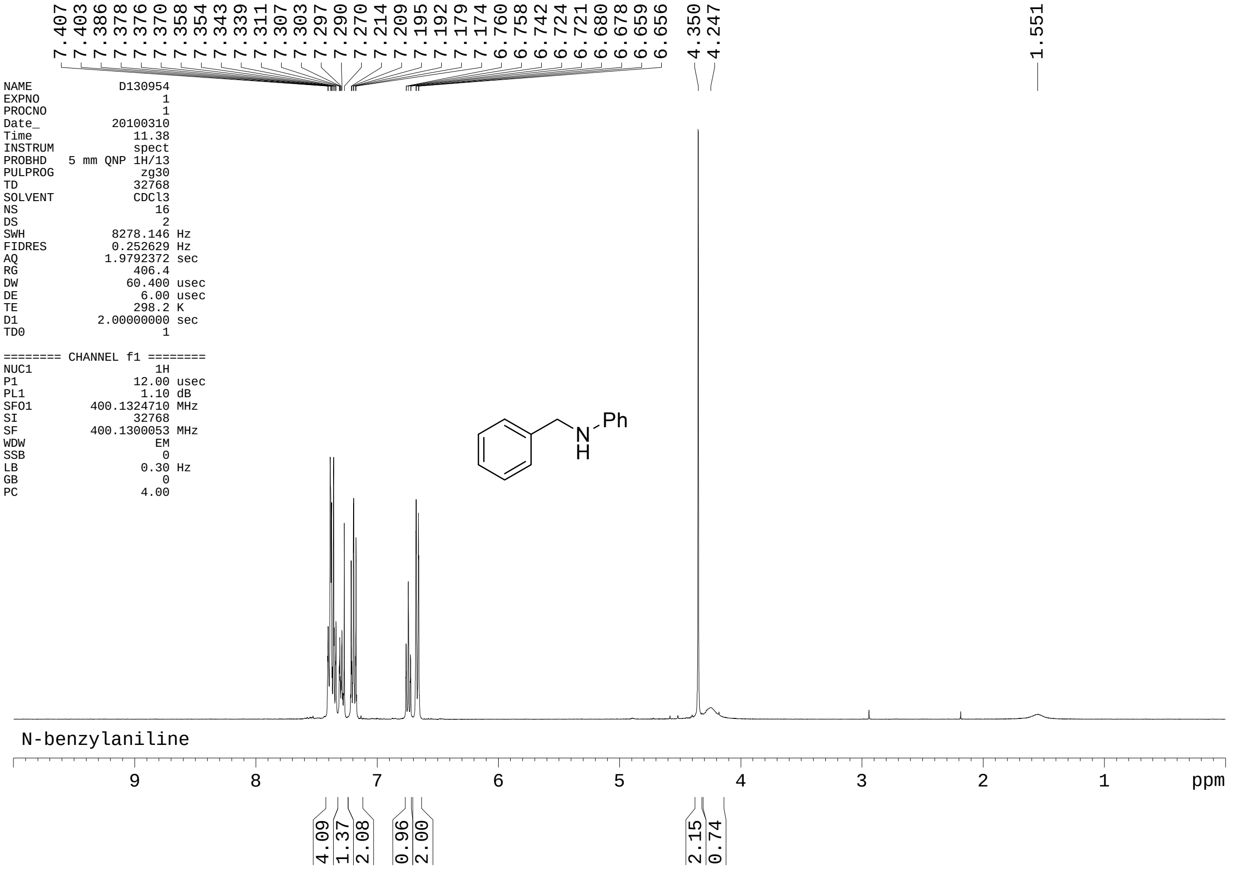
7.407 7.403 7.386 7.378 7.376 7.370 7.358 7.354 7.343 7.339 7.311 7.307 7.303 7.297 7.290 7.270 7.214 7.209 7.195 7.192 7.179 7.174 6.760 6.758 6.742 6.724 6.721 6.680 6.678 6.659 6.656 4.350 4.247 1.551
NAME D130954
EXPNO 1
PROCNO 1
Date_ 20100310
Time 11.38
INSTRUM spect
PROBHD 5 mm QNP 1H/13
PULPROG zg30
TD 32768
SOLVENT CDCl3
NS 16
DS 2
SWH 8278.146 Hz
FIDRES 0.252629 Hz
AQ 1.9792372 sec
RG 406.4
DW 60.400 usec
DE 6.00 usec
TE 298.2 K
D1 2.00000000 sec
TD0 1
======== CHANNEL f1 ========
NUC1 1H
P1 12.00 usec
PL1 1.10 dB
SFO1 400.1324710 MHz
SI 32768
SF 400.1300053 MHz
WDW EM
SSB 0
LB 0.30 Hz
GB 0
PC 4.00
N Ph H
N-benzylaniline
9 8 7 6 5 4 3 2 1 ppm
4.09 1.37 2.08 0.96 2.00 2.15 0.74

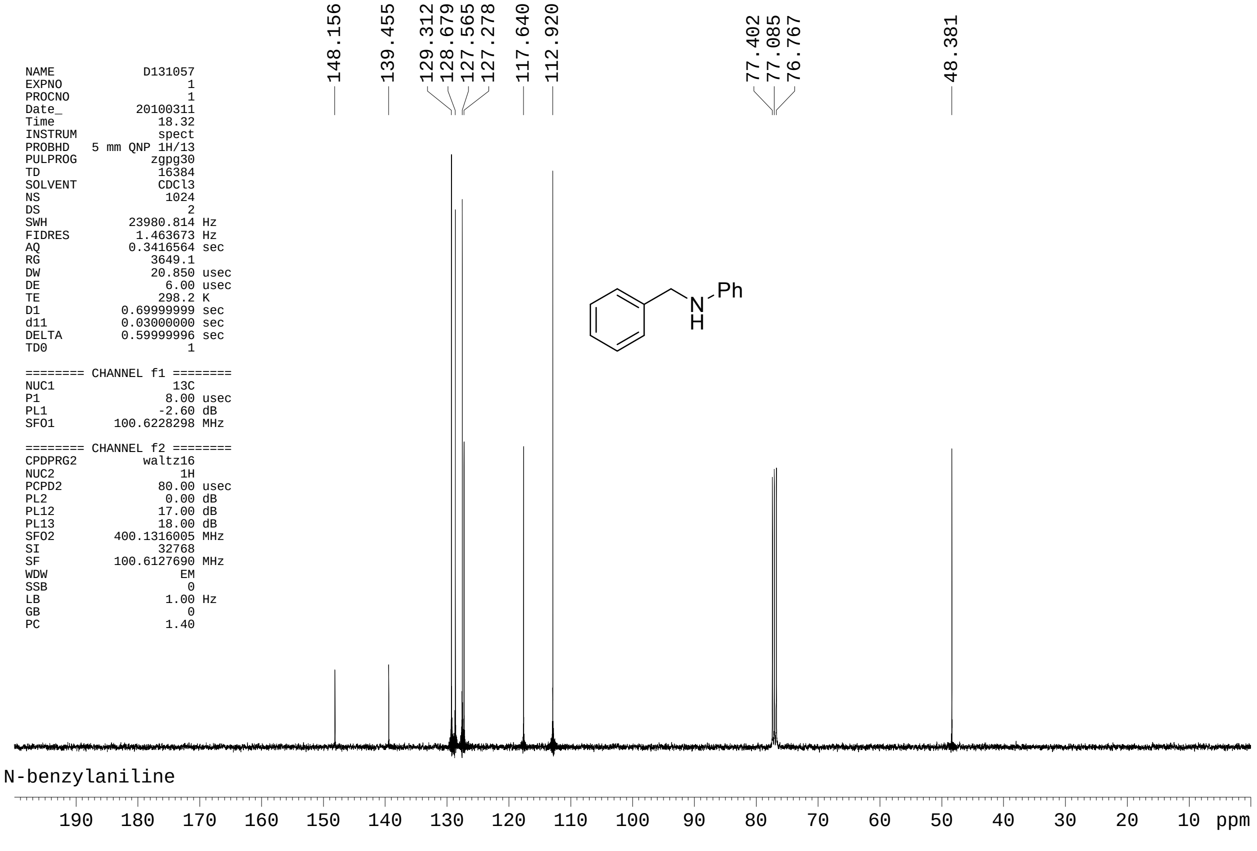
148.156 139.455 129.312 128.679 127.565 127.278 117.640 112.920 77.402 77.085 76.767 48.381
NAME D131057
EXPNO 1
PROCNO 1
Date_ 20100311
Time 18.32
INSTRUM spect
PROBHD 5 mm QNP 1H/13
PULPROG zgpg30
TD 16384
SOLVENT CDCl3
NS 1024
DS 2
SWH 23980.814 Hz
FIDRES 1.463673 Hz
AQ 0.3416564 sec
RG 3649.1
DW 20.850 usec
DE 6.00 usec
TE 298.2 K
D1 0.69999999 sec
d11 0.03000000 sec
DELTA 0.59999996 sec
TD0 1
======== CHANNEL f1 ========
NUC1 13C
P1 8.00 usec
PL1 -2.60 dB
SFO1 100.6228298 MHz
======== CHANNEL f2 ========
CPDPRG2 waltz16
NUC2 1H
PCPD2 80.00 usec
PL2 0.00 dB
PL12 17.00 dB
PL13 18.00 dB
SFO2 400.1316005 MHz
SI 32768
SF 100.6127690 MHz
WDW EM
SSB 0
LB 1.00 Hz
GB 0
PC 1.40
N Ph H
N-benzylaniline
190 180 170 160 150 140 130 120 110 100 90 80 70 60 50 40 30 20 10 ppm

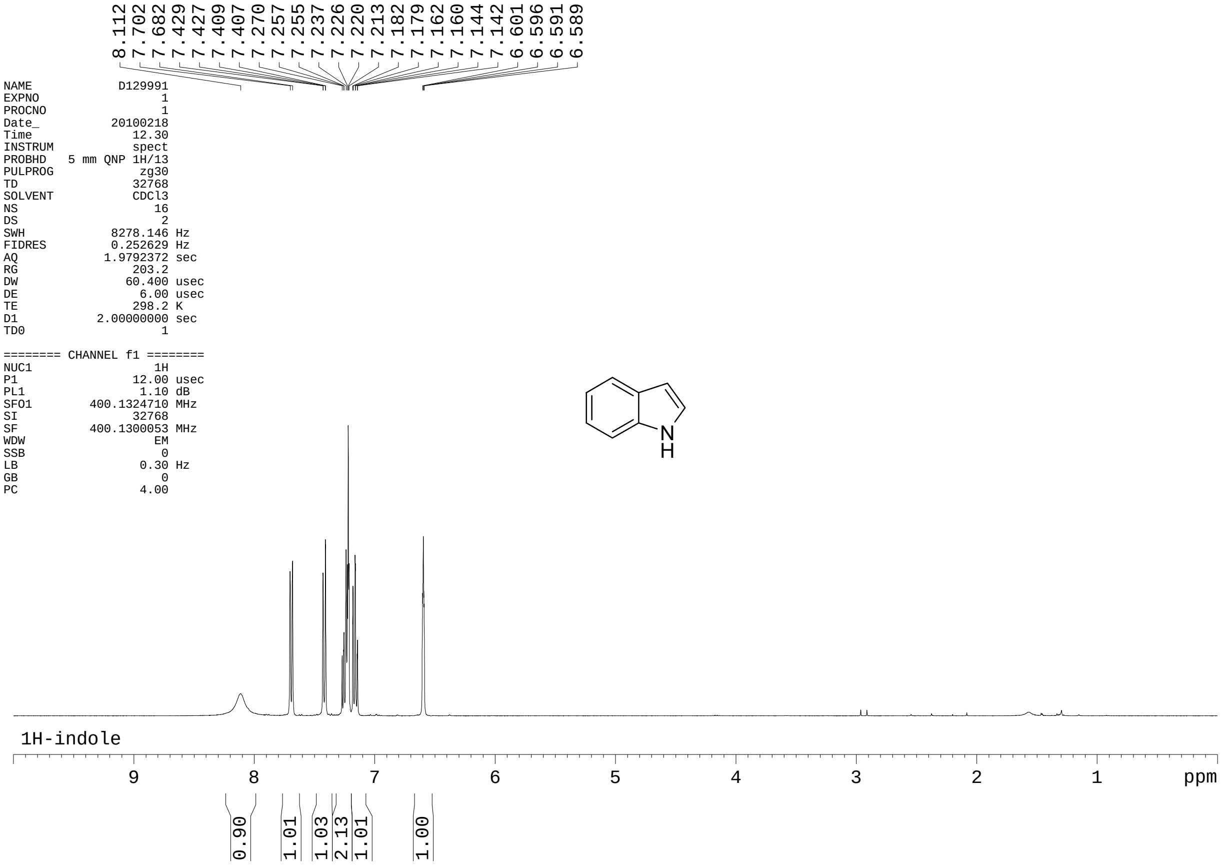
8.112
7.702
7.682
7.429
7.427
7.409
7.407
7.270
7.257
7.255
7.237
7.226
7.220
7.213
7.182
7.179
7.162
7.160
7.144
7.142
6.601
6.596
6.591
6.589
NAME D129991
EXPNO 1
PROCNO 1
Date_ 20100218
Time 12.30
INSTRUM spect
PROBHD 5 mm QNP 1H/13
PULPROG zg30
TD 32768
SOLVENT CDCl3
NS 16
DS 2
SWH 8278.146 Hz
FIDRES 0.252629 Hz
AQ 1.9792372 sec
RG 203.2
DW 60.400 usec
DE 6.00 usec
TE 298.2 K
D1 2.00000000 sec
TD0 1
======== CHANNEL f1 ========
NUC1 1H
P1 12.00 usec
PL1 1.10 dB
SFO1 400.1324710 MHz
SI 32768
SF 400.1300053 MHz
WDW EM
SSB 0
LB 0.30 Hz
GB 0
PC 4.00
N
H
1H-indole
9
8
7
6
5
4
3
2
1
ppm
0.90
1.01
1.03
2.13
1.01
1.00

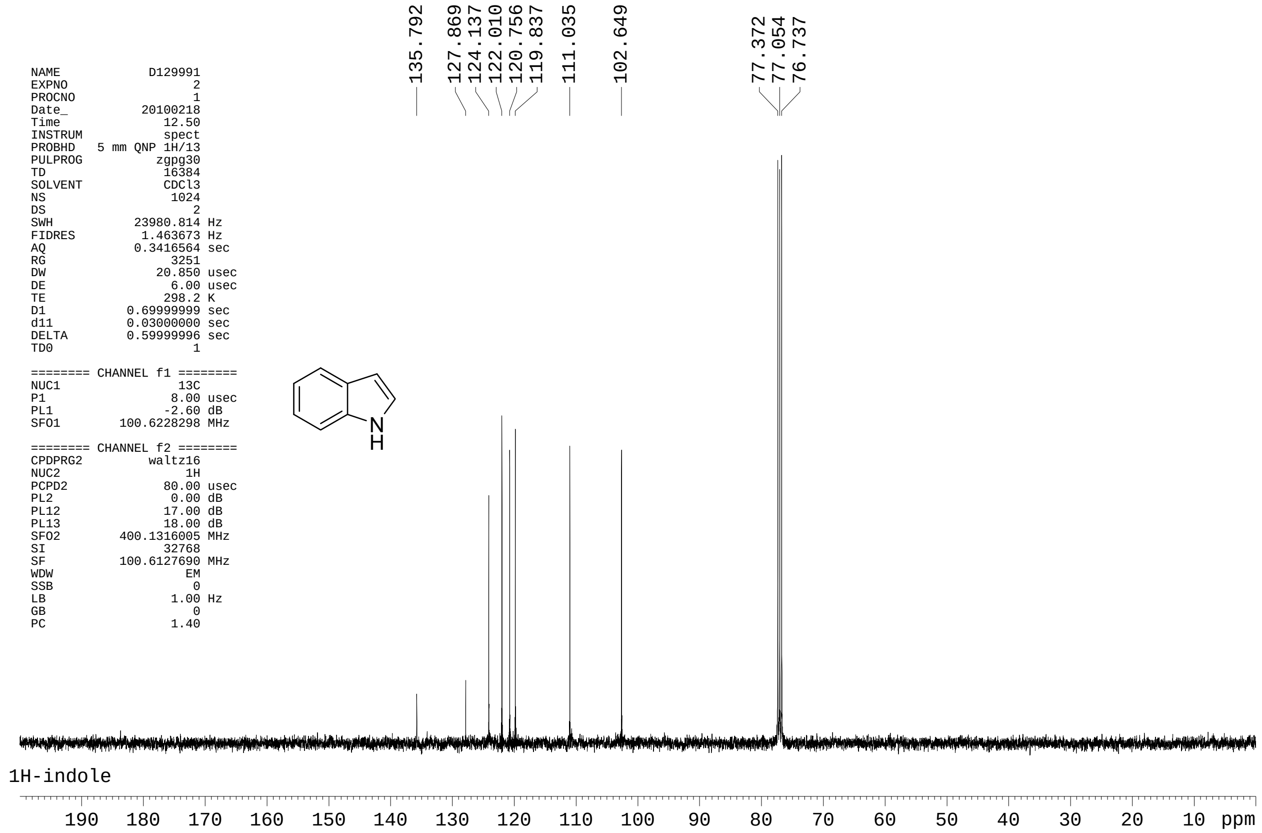
135.792
127.869
124.137
122.010
120.756
119.837
111.035
102.649
77.372
77.054
76.737
NAME D129991
EXPNO 2
PROCNO 1
Date_ 20100218
Time 12.50
INSTRUM spect
PROBHD 5 mm QNP 1H/13
PULPROG zgpg30
TD 16384
SOLVENT CDCl3
NS 1024
DS 2
SWH 23980.814 Hz
FIDRES 1.463673 Hz
AQ 0.3416564 sec
RG 3251
DW 20.850 usec
DE 6.00 usec
TE 298.2 K
D1 0.69999999 sec
d11 0.03000000 sec
DELTA 0.59999996 sec
TD0 1
======== CHANNEL f1 ========
NUC1 13C
P1 8.00 usec
PL1 -2.60 dB
SFO1 100.6228298 MHz
======== CHANNEL f2 ========
CPDPRG2 waltz16
NUC2 1H
PCPD2 80.00 usec
PL2 0.00 dB
PL12 17.00 dB
PL13 18.00 dB
SFO2 400.1316005 MHz
SI 32768
SF 100.6127690 MHz
WDW EM
SSB 0
LB 1.00 Hz
GB 0
PC 1.40
N
H
1H-indole
190
180
170
160
150
140
130
120
110
100
90
80
70
60
50
40
30
20
10
ppm

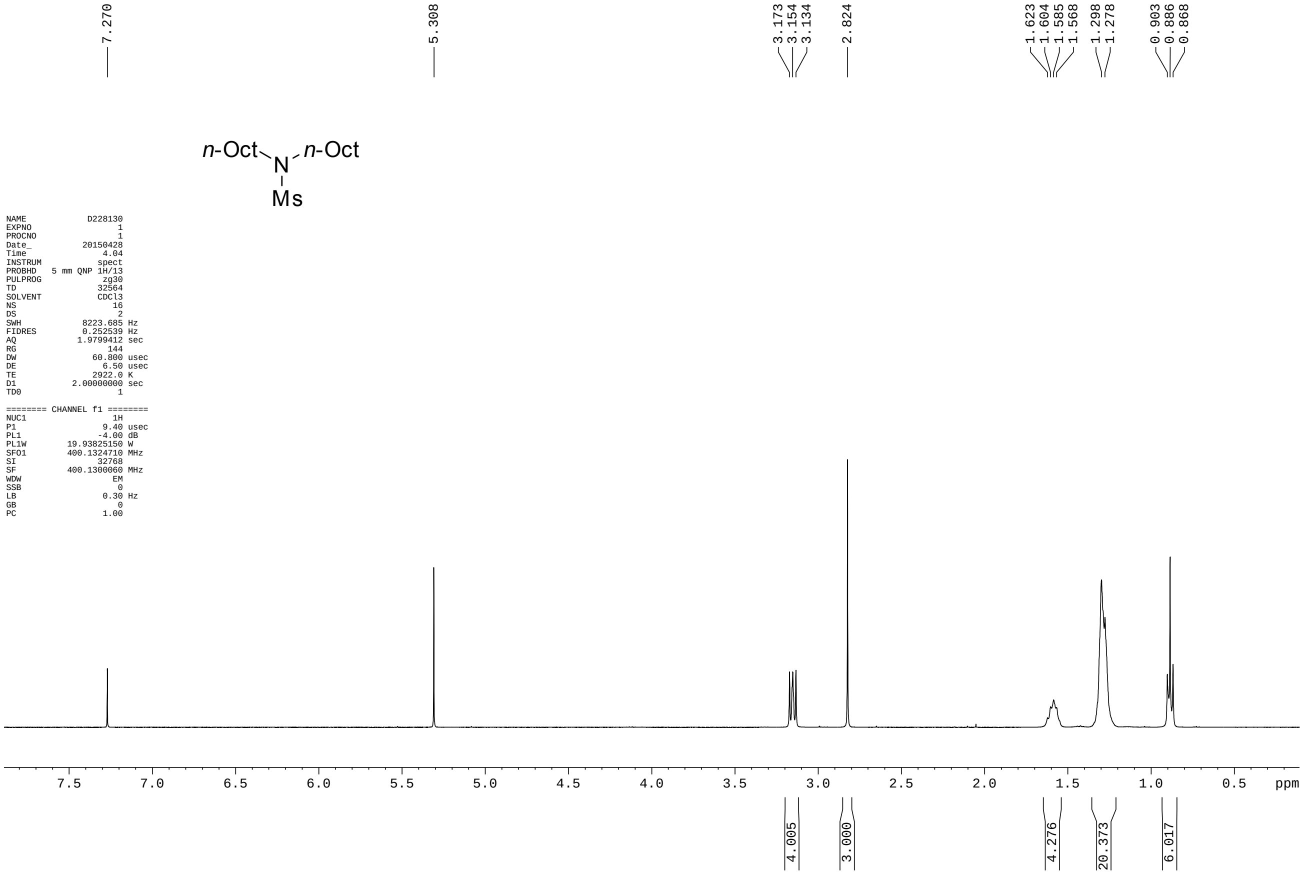
7.270
5.308
3.173
3.154
3.134
2.824
1.623
1.604
1.585
1.568
1.298
1.278
0.903
0.886
0.868
n-Oct N n-Oct
Ms
4.005
3.000
4.276
20.373
6.017
ppm

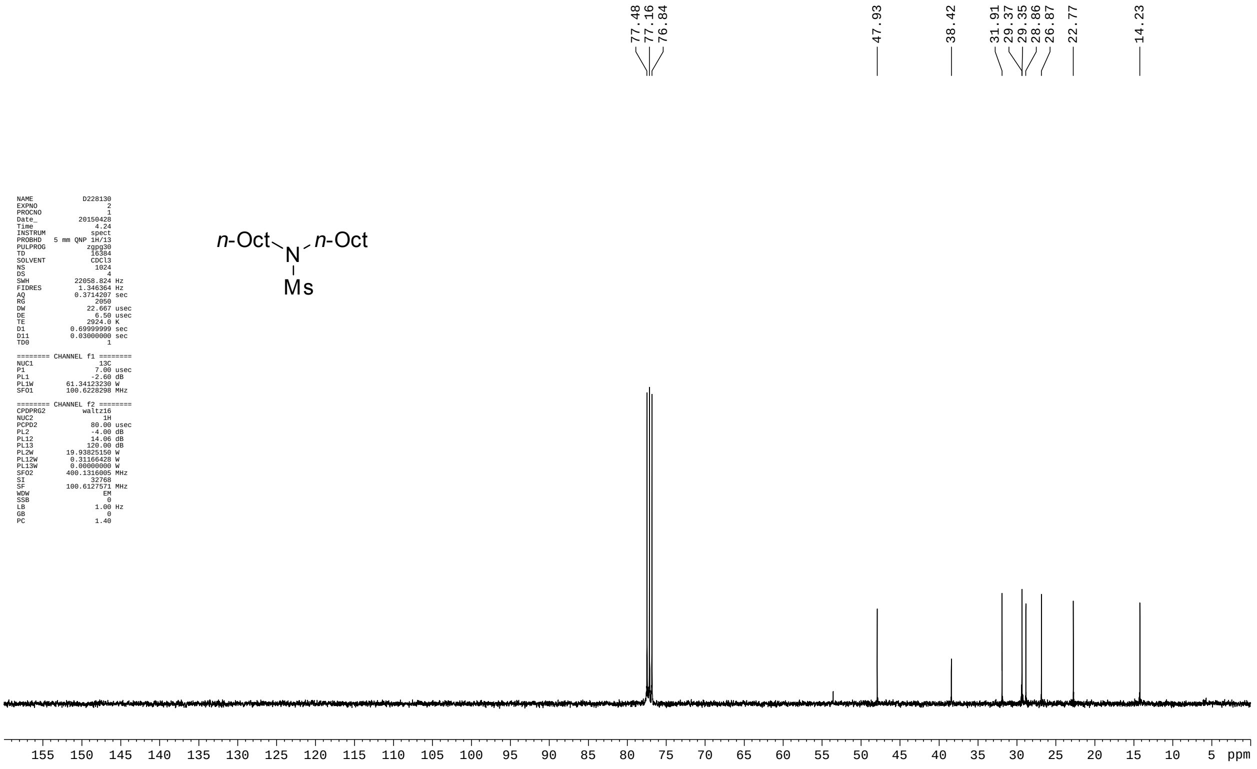
77.48
77.16
76.84
47.93
38.42
31.91
29.37
29.35
28.86
26.87
22.77
14.23
n-Oct N n-Oct
Ms
ppm

```
NAME           D225097
EXPNO                1
PROCNO               1
Date_         20150322
Time             16.58
INSTRUM          spect
PROBHD   5 mm QNP 1H/13
PULPROG           zg30
TD               32564
SOLVENT          CDCl3
NS                  16
DS                   2
SWH           8223.685 Hz
FIDRES        0.252539 Hz
AQ           1.9799412 sec
RG                 406
DW              60.800 usec
DE                6.50 usec
TE               300.0 K
D1          2.00000000 sec
TD0                  1

======== CHANNEL f1 ========
NUC1                1H
P1                9.40 usec
PL1              -4.00 dB
PL1W       19.93825150 W
SFO1       400.1324710 MHz
SI               32768
SF         400.1300059 MHz
WDW                 EM
SSB                  0
LB                0.30 Hz
GB                   0
PC                1.00
```

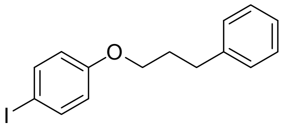

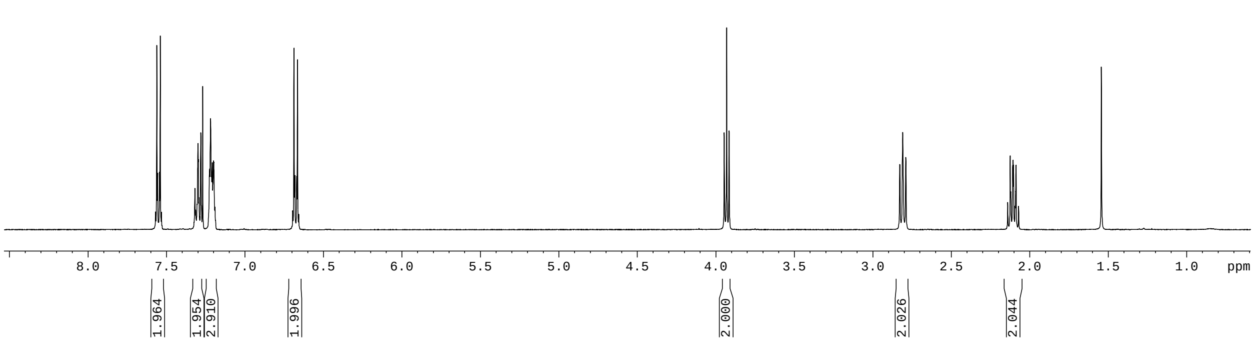


```
NAME            B35934
EXPNO                1
PROCNO               1
Date_         20150403
Time             21.53
INSTRUM          AV400
PROBHD   5 mm PABBO BB-
PULPROG         zgpg30
TD               16384
SOLVENT          CDCl3
NS                1024
DS                   2
SWH          23980.814 Hz
FIDRES        1.463673 Hz
AQ           0.3416564 sec
RG             18390.4
DW              20.850 usec
DE               20.00 usec
TE               300.0 K
D1          0.69999999 sec
D11         0.03000000 sec
TD0                  1

======== CHANNEL f1 ========
NUC1               13C
P1                8.00 usec
PL1              -2.80 dB
PL1W       57.91424179 W
SFO1       100.5976818 MHz

======== CHANNEL f2 ========
CPDPRG2        waltz16
NUC2                1H
PCPD2            90.00 usec
PL2              -3.20 dB
PL12             14.30 dB
PL13             15.30 dB
PL2W       15.00949383 W
PL12W       0.26691073 W
PL13W       0.21201472 W
SFO2       400.0316001 MHz
SI               32768
SF         100.5876108 MHz
WDW                 EM
SSB                  0
LB                1.00 Hz
GB                   0
PC                1.40
```

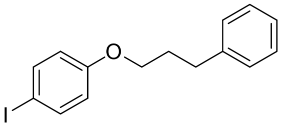

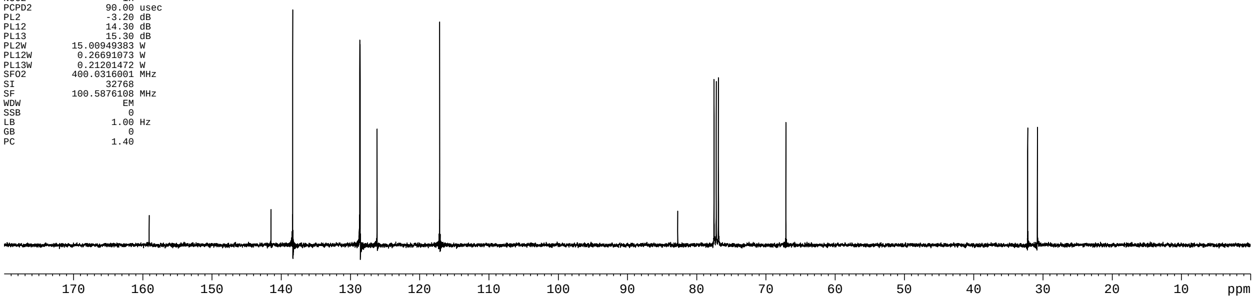

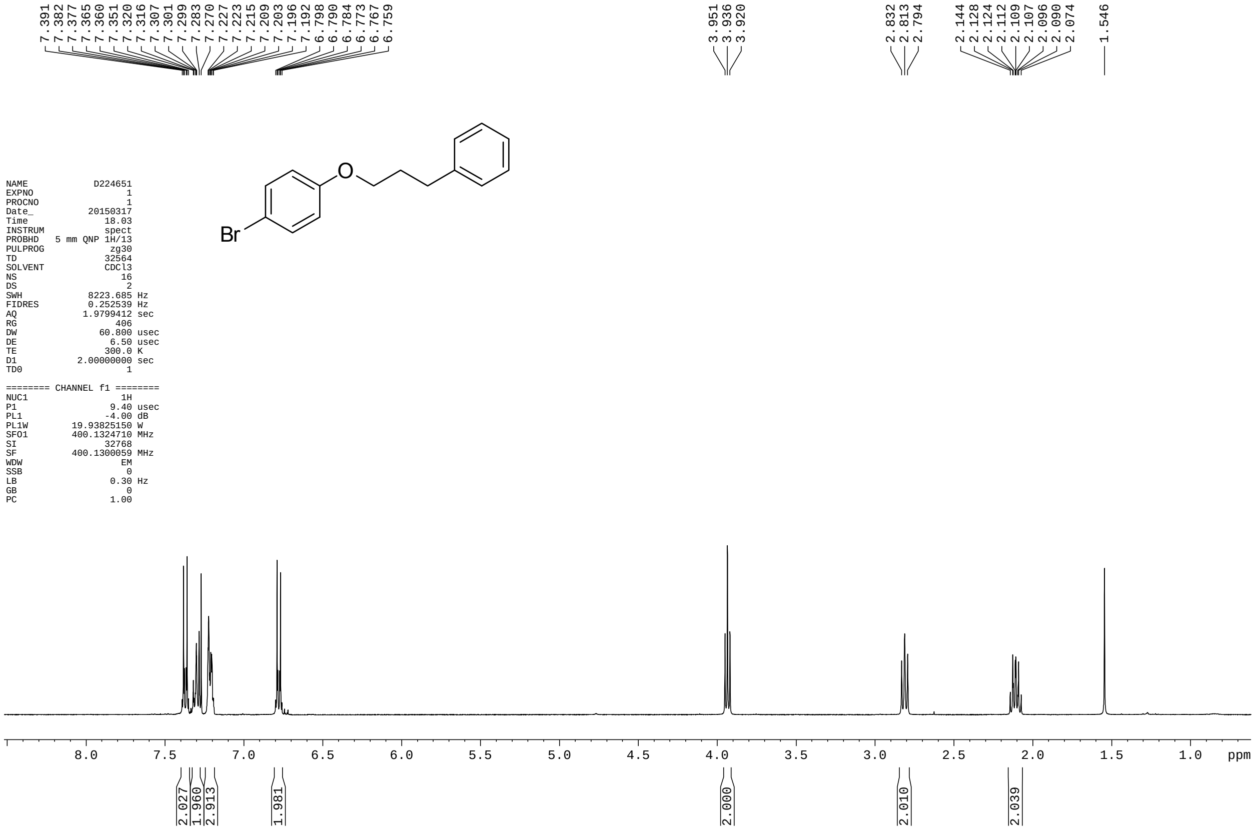
7.391 7.382 7.377 7.365 7.360 7.351 7.320 7.316 7.307 7.301 7.299 7.283 7.270 7.227 7.223 7.215 7.209 7.203 7.196 7.192 6.798 6.790 6.784 6.773 6.767 6.759
3.951 3.936 3.920
2.832 2.813 2.794
2.144 2.128 2.124 2.112 2.109 2.107 2.096 2.090 2.074
1.546
Br
O
NAME D224651
EXPNO 1
PROCNO 1
Date_ 20150317
Time 18.03
INSTRUM spect
PROBHD 5 mm QNP 1H/13
PULPROG zg30
TD 32564
SOLVENT CDCl3
NS 16
DS 2
SWH 8223.685 Hz
FIDRES 0.252539 Hz
AQ 1.9799412 sec
RG 406
DW 60.800 usec
DE 6.50 usec
TE 300.0 K
D1 2.00000000 sec
TD0 1
======== CHANNEL f1 ========
NUC1 1H
P1 9.40 usec
PL1 -4.00 dB
PL1W 19.93825150 W
SFO1 400.1324710 MHz
SI 32768
SF 400.1300059 MHz
WDW EM
SSB 0
LB 0.30 Hz
GB 0
PC 1.00
8.0 7.5 7.0 6.5 6.0 5.5 5.0 4.5 4.0 3.5 3.0 2.5 2.0 1.5 1.0 ppm
2.027 1.960 2.913 1.981 2.000 2.010 2.039

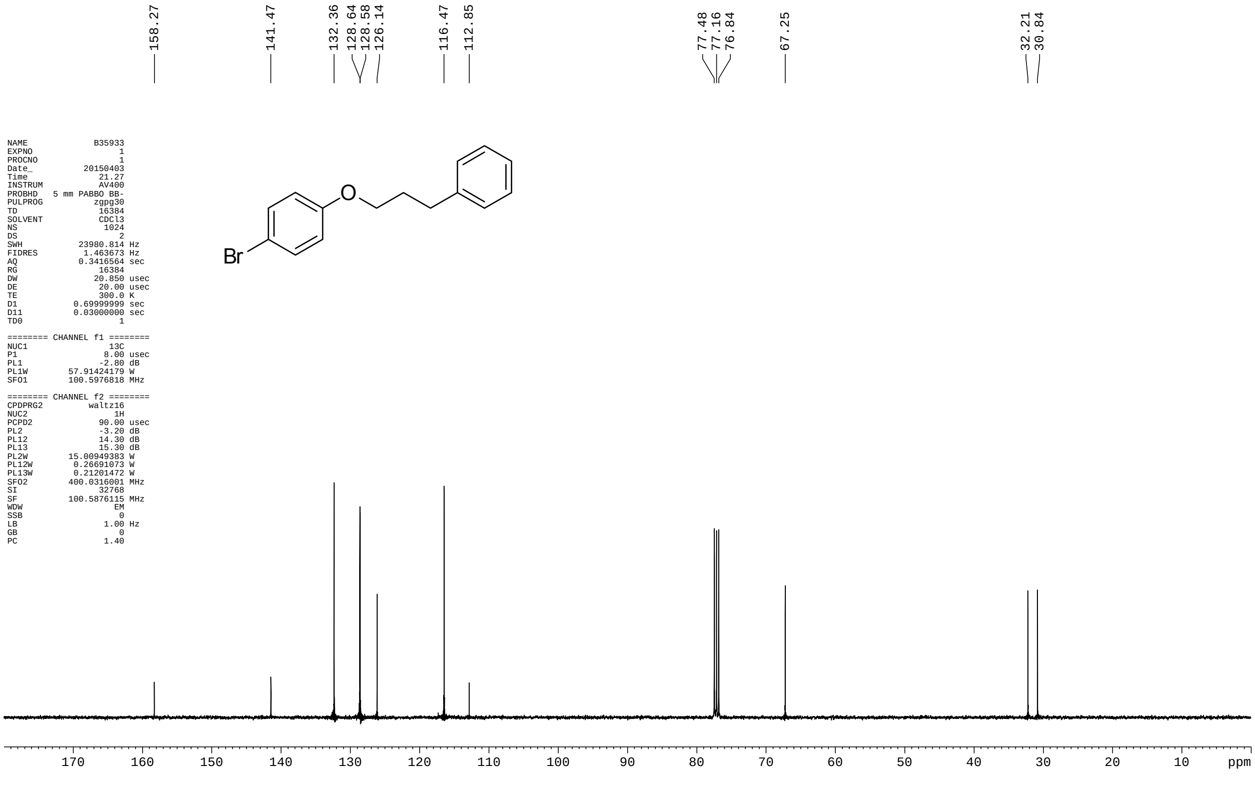
158.27 141.47 132.36 128.64 128.58 126.14 116.47 112.85 77.48 77.16 76.84 67.25 32.21 30.84
Br
O
NAME B35933
EXPNO 1
PROCNO 1
Date_ 20150403
Time 21.27
INSTRUM AV400
PROBHD 5 mm PABBO BB-
PULPROG zgpg30
TD 16384
SOLVENT CDCl3
NS 1024
DS 2
SWH 23980.814 Hz
FIDRES 1.463673 Hz
AQ 0.3416564 sec
RG 16384
DW 20.850 usec
DE 20.00 usec
TE 300.0 K
D1 0.69999999 sec
D11 0.03000000 sec
TD0 1
======== CHANNEL f1 ========
NUC1 13C
P1 8.00 usec
PL1 -2.80 dB
PL1W 57.91424179 W
SFO1 100.5976818 MHz
======== CHANNEL f2 ========
CPDPRG2 waltz16
NUC2 1H
PCPD2 90.00 usec
PL2 -3.20 dB
PL12 14.30 dB
PL13 15.30 dB
PL2W 15.00949383 W
PL12W 0.26691073 W
PL13W 0.21201472 W
SFO2 400.0316001 MHz
SI 32768
SF 100.5876115 MHz
WDW EM
SSB 0
LB 1.00 Hz
GB 0
PC 1.40
170 160 150 140 130 120 110 100 90 80 70 60 50 40 30 20 10 ppm

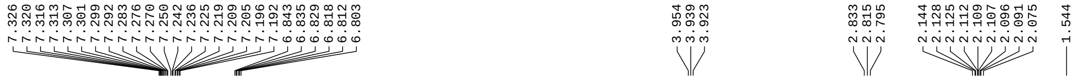


```
NAME            D225098
EXPNO                 1
PROCNO                1
Date_          20150322
Time              17.03
INSTRUM           spect
PROBHD   5 mm QNP 1H/13
PULPROG            zg30
TD                32564
SOLVENT           CDCl3
NS                   16
DS                    2
SWH            8223.685 Hz
FIDRES         0.252539 Hz
AQ            1.9799412 sec
RG                  406
DW               60.800 usec
DE                 6.50 usec
TE                300.0 K
D1           2.00000000 sec
TD0                   1

======== CHANNEL f1 ========
NUC1                 1H
P1                 9.40 usec
PL1               -4.00 dB
PL1W        19.93825150 W
SFO1        400.1324710 MHz
SI                32768
SF          400.1300059 MHz
WDW                  EM
SSB                   0
LB                 0.30 Hz
GB                    0
PC                 1.00
```

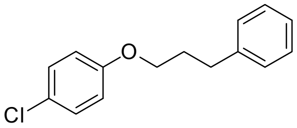

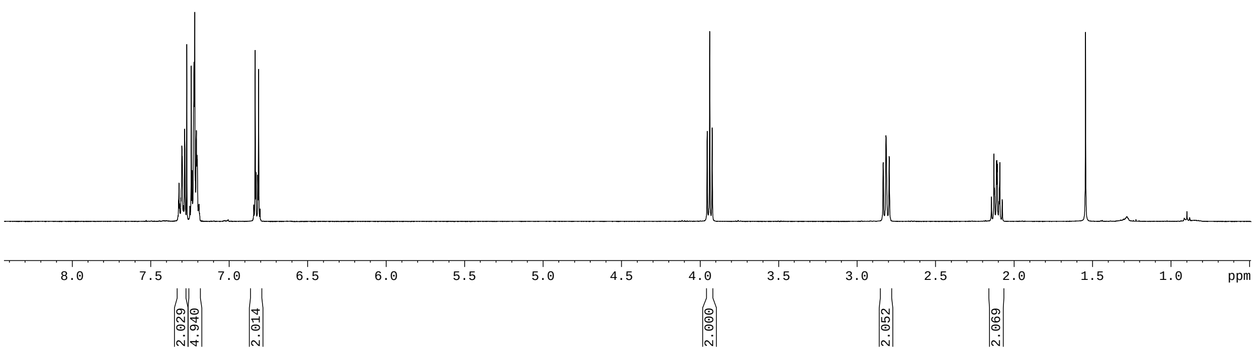

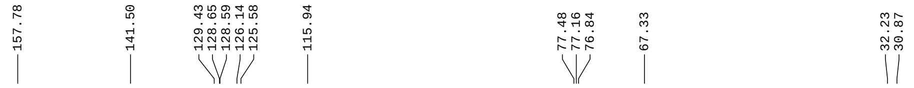


```
NAME             B35935
EXPNO                 1
PROCNO                1
Date_          20150403
Time              22.20
INSTRUM           AV400
PROBHD   5 mm PABBO BB-
PULPROG         zgpg30
TD                16384
SOLVENT           CDCl3
NS                 1024
DS                    2
SWH           23980.814 Hz
FIDRES         1.463673 Hz
AQ            0.3416564 sec
RG              20642.5
DW               20.850 usec
DE                20.00 usec
TE                300.0 K
D1           0.69999999 sec
D11          0.03000000 sec
TD0                   1

======== CHANNEL f1 ========
NUC1                13C
P1                 8.00 usec
PL1               -2.80 dB
PL1W        57.91424179 W
SFO1        100.5976818 MHz

======== CHANNEL f2 ========
CPDPRG2         waltz16
NUC2                 1H
PCPD2             90.00 usec
PL2               -3.20 dB
PL12              14.30 dB
PL13              15.30 dB
PL2W        15.00949383 W
PL12W        0.26691073 W
PL13W        0.21201472 W
SFO2        400.0316001 MHz
SI                32768
SF          100.5876096 MHz
WDW                  EM
SSB                   0
LB                 1.00 Hz
GB                    0
PC                 1.40
```

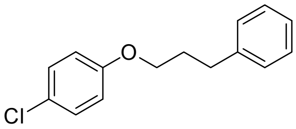

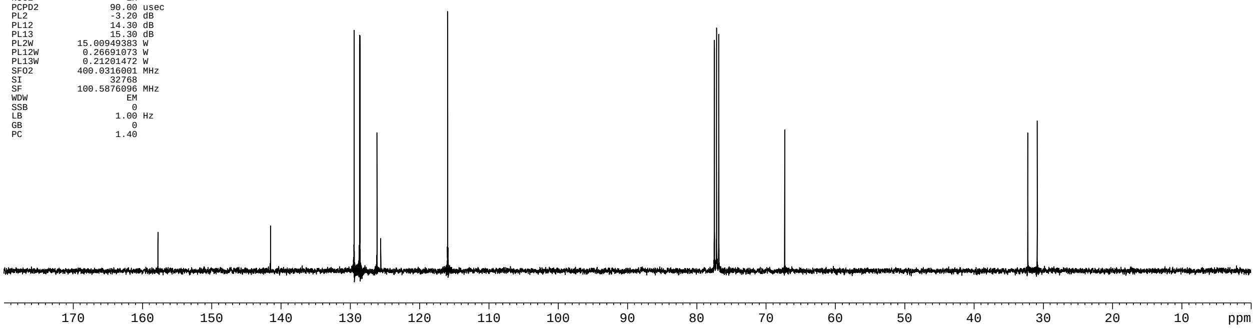

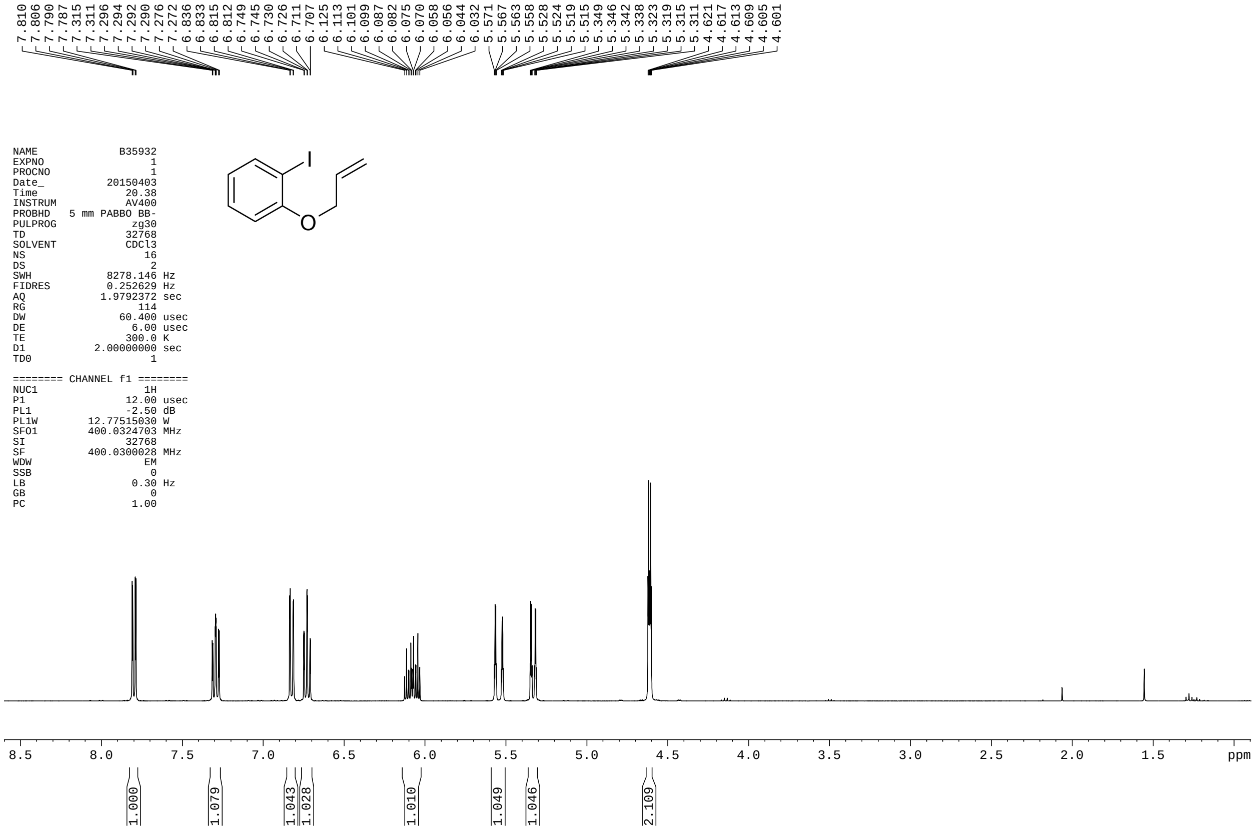
7.810 7.806 7.790 7.787 7.315 7.311 7.296 7.294 7.292 7.290 7.276 7.272 6.836 6.833 6.815 6.812 6.749 6.745 6.730 6.726 6.711 6.707 6.125 6.113 6.101 6.099 6.087 6.082 6.075 6.070 6.058 6.056 6.044 6.032 5.571 5.567 5.563 5.558 5.528 5.524 5.519 5.515 5.349 5.346 5.342 5.338 5.323 5.319 5.315 5.311 4.621 4.617 4.613 4.609 4.605 4.601
NAME B35932
EXPNO 1
PROCNO 1
Date_ 20150403
Time 20.38
INSTRUM AV400
PROBHD 5 mm PABBO BB-
PULPROG zg30
TD 32768
SOLVENT CDCl3
NS 16
DS 2
SWH 8278.146 Hz
FIDRES 0.252629 Hz
AQ 1.9792372 sec
RG 114
DW 60.400 usec
DE 6.00 usec
TE 300.0 K
D1 2.00000000 sec
TD0 1
======== CHANNEL f1 ========
NUC1 1H
P1 12.00 usec
PL1 -2.50 dB
PL1W 12.77515030 W
SFO1 400.0324703 MHz
SI 32768
SF 400.0300028 MHz
WDW EM
SSB 0
LB 0.30 Hz
GB 0
PC 1.00
I
O
8.5 8.0 7.5 7.0 6.5 6.0 5.5 5.0 4.5 4.0 3.5 3.0 2.5 2.0 1.5 ppm
1.000 1.079 1.043 1.028 1.010 1.049 1.046 2.109

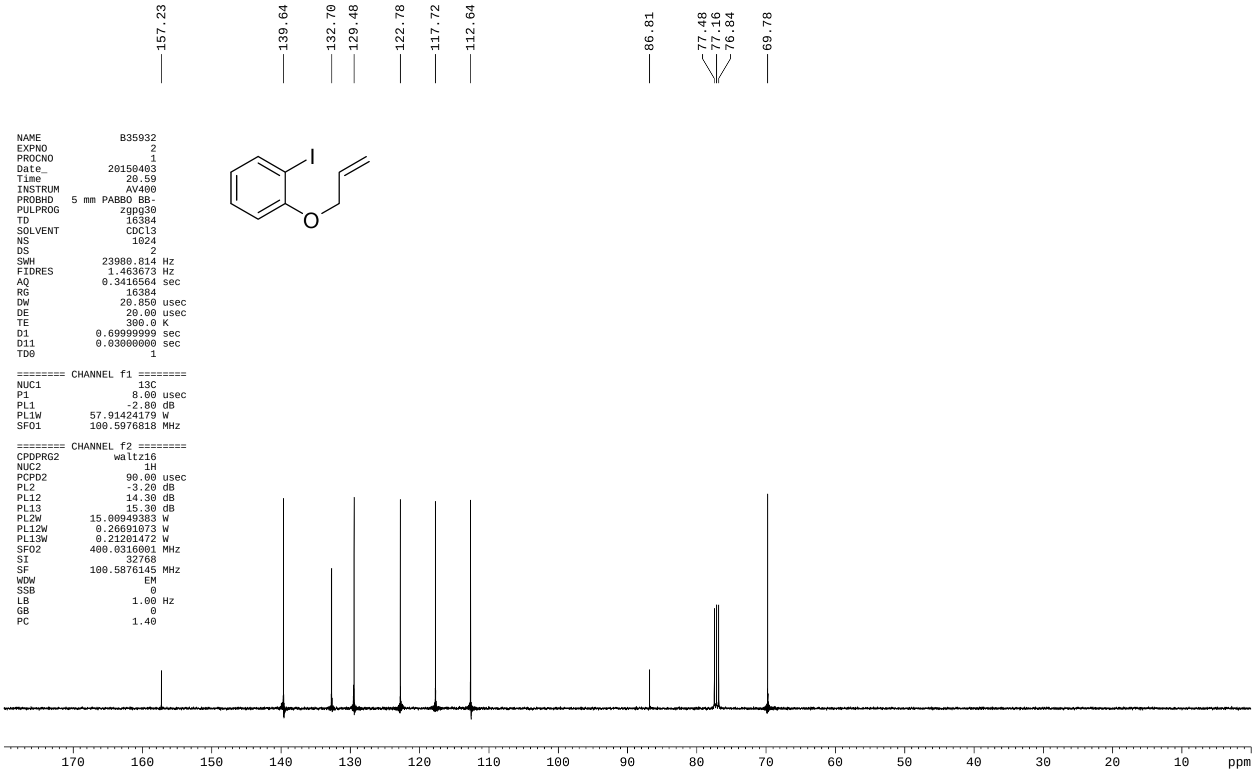
157.23 139.64 132.70 129.48 122.78 117.72 112.64 86.81 77.48 77.16 76.84 69.78
NAME B35932
EXPNO 2
PROCNO 1
Date_ 20150403
Time 20.59
INSTRUM AV400
PROBHD 5 mm PABBO BB-
PULPROG zgpg30
TD 16384
SOLVENT CDCl3
NS 1024
DS 2
SWH 23980.814 Hz
FIDRES 1.463673 Hz
AQ 0.3416564 sec
RG 16384
DW 20.850 usec
DE 20.00 usec
TE 300.0 K
D1 0.69999999 sec
D11 0.03000000 sec
TD0 1
======== CHANNEL f1 ========
NUC1 13C
P1 8.00 usec
PL1 -2.80 dB
PL1W 57.91424179 W
SFO1 100.5976818 MHz
======== CHANNEL f2 ========
CPDPRG2 waltz16
NUC2 1H
PCPD2 90.00 usec
PL2 -3.20 dB
PL12 14.30 dB
PL13 15.30 dB
PL2W 15.00949383 W
PL12W 0.26691073 W
PL13W 0.21201472 W
SFO2 400.0316001 MHz
SI 32768
SF 100.5876145 MHz
WDW EM
SSB 0
LB 1.00 Hz
GB 0
PC 1.40
I
O
170 160 150 140 130 120 110 100 90 80 70 60 50 40 30 20 10 ppm

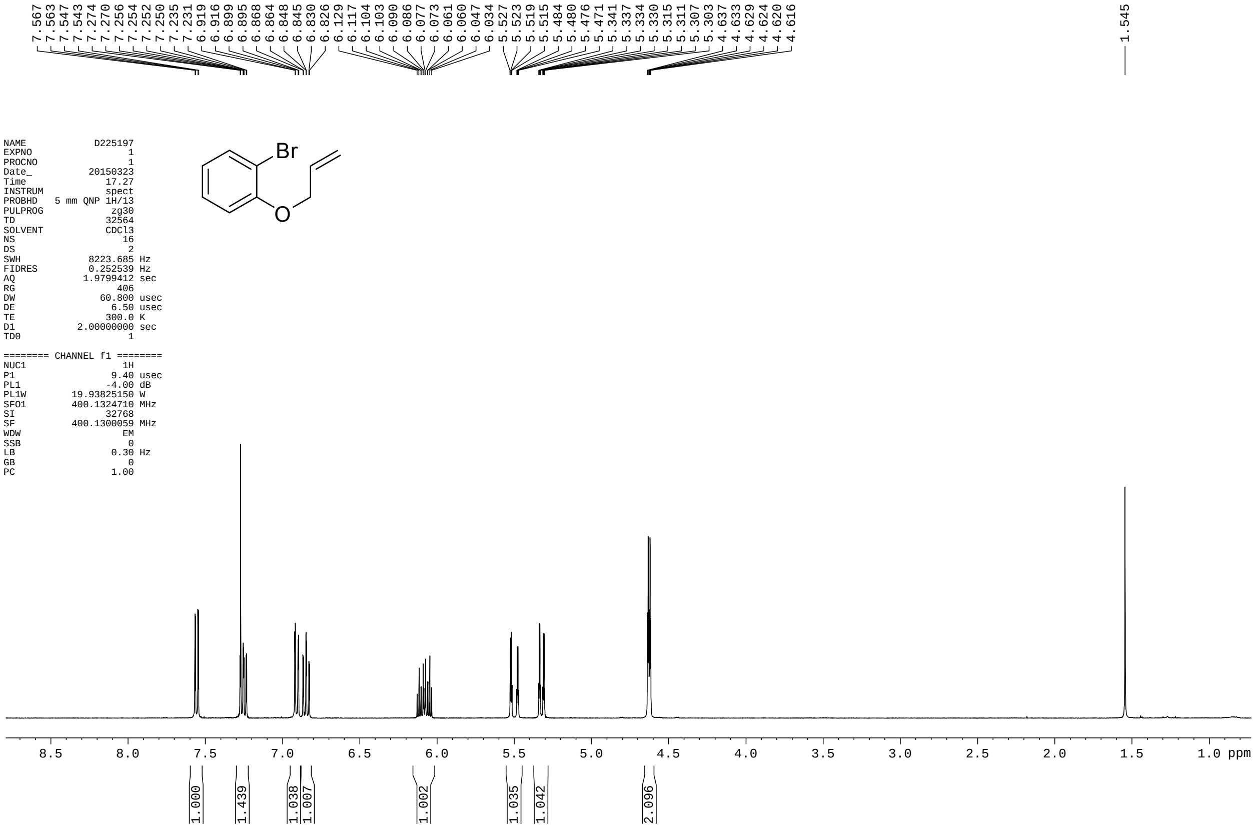
7.567 7.563 7.547 7.543 7.274 7.270 7.256 7.254 7.252 7.250 7.235 7.231 6.919 6.916 6.899 6.895 6.868 6.864 6.848 6.845 6.830 6.826 6.129 6.117 6.104 6.103 6.090 6.086 6.077 6.073 6.061 6.060 6.047 6.034 5.527 5.523 5.519 5.515 5.484 5.480 5.476 5.471 5.341 5.337 5.334 5.330 5.315 5.311 5.307 5.303 4.637 4.633 4.629 4.624 4.620 4.616
1.545
Br
O
NAME D225197
EXPNO 1
PROCNO 1
Date_ 20150323
Time 17.27
INSTRUM spect
PROBHD 5 mm QNP 1H/13
PULPROG zg30
TD 32564
SOLVENT CDCl3
NS 16
DS 2
SWH 8223.685 Hz
FIDRES 0.252539 Hz
AQ 1.9799412 sec
RG 406
DW 60.800 usec
DE 6.50 usec
TE 300.0 K
D1 2.00000000 sec
TD0 1
======== CHANNEL f1 ========
NUC1 1H
P1 9.40 usec
PL1 -4.00 dB
PL1W 19.93825150 W
SFO1 400.1324710 MHz
SI 32768
SF 400.1300059 MHz
WDW EM
SSB 0
LB 0.30 Hz
GB 0
PC 1.00
8.5 8.0 7.5 7.0 6.5 6.0 5.5 5.0 4.5 4.0 3.5 3.0 2.5 2.0 1.5 1.0 ppm
1.000 1.439 1.038 1.007 1.002 1.035 1.042 2.096

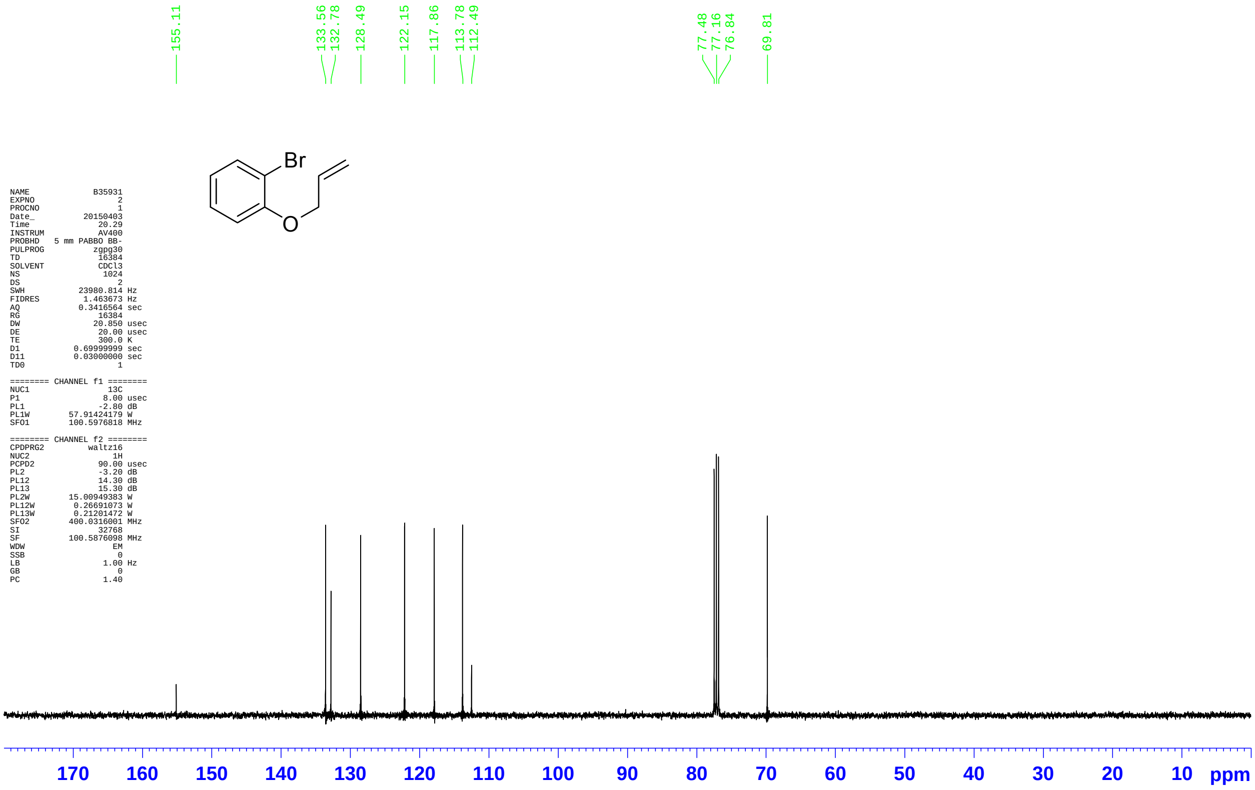
155.11 133.56 132.78 128.49 122.15 117.86 113.78 112.49 77.48 77.16 76.84 69.81
Br
O
NAME B35931
EXPNO 2
PROCNO 1
Date_ 20150403
Time 20.29
INSTRUM AV400
PROBHD 5 mm PABBO BB-
PULPROG zgpg30
TD 16384
SOLVENT CDCl3
NS 1024
DS 2
SWH 23980.814 Hz
FIDRES 1.463673 Hz
AQ 0.3416564 sec
RG 16384
DW 20.850 usec
DE 20.00 usec
TE 300.0 K
D1 0.69999999 sec
D11 0.03000000 sec
TD0 1
======== CHANNEL f1 ========
NUC1 13C
P1 8.00 usec
PL1 -2.80 dB
PL1W 57.91424179 W
SFO1 100.5976818 MHz
======== CHANNEL f2 ========
CPDPRG2 waltz16
NUC2 1H
PCPD2 90.00 usec
PL2 -3.20 dB
PL12 14.30 dB
PL13 15.30 dB
PL2W 15.00949383 W
PL12W 0.26691073 W
PL13W 0.21201472 W
SFO2 400.0316001 MHz
SI 32768
SF 100.5876098 MHz
WDW EM
SSB 0
LB 1.00 Hz
GB 0
PC 1.40
170 160 150 140 130 120 110 100 90 80 70 60 50 40 30 20 10 ppm

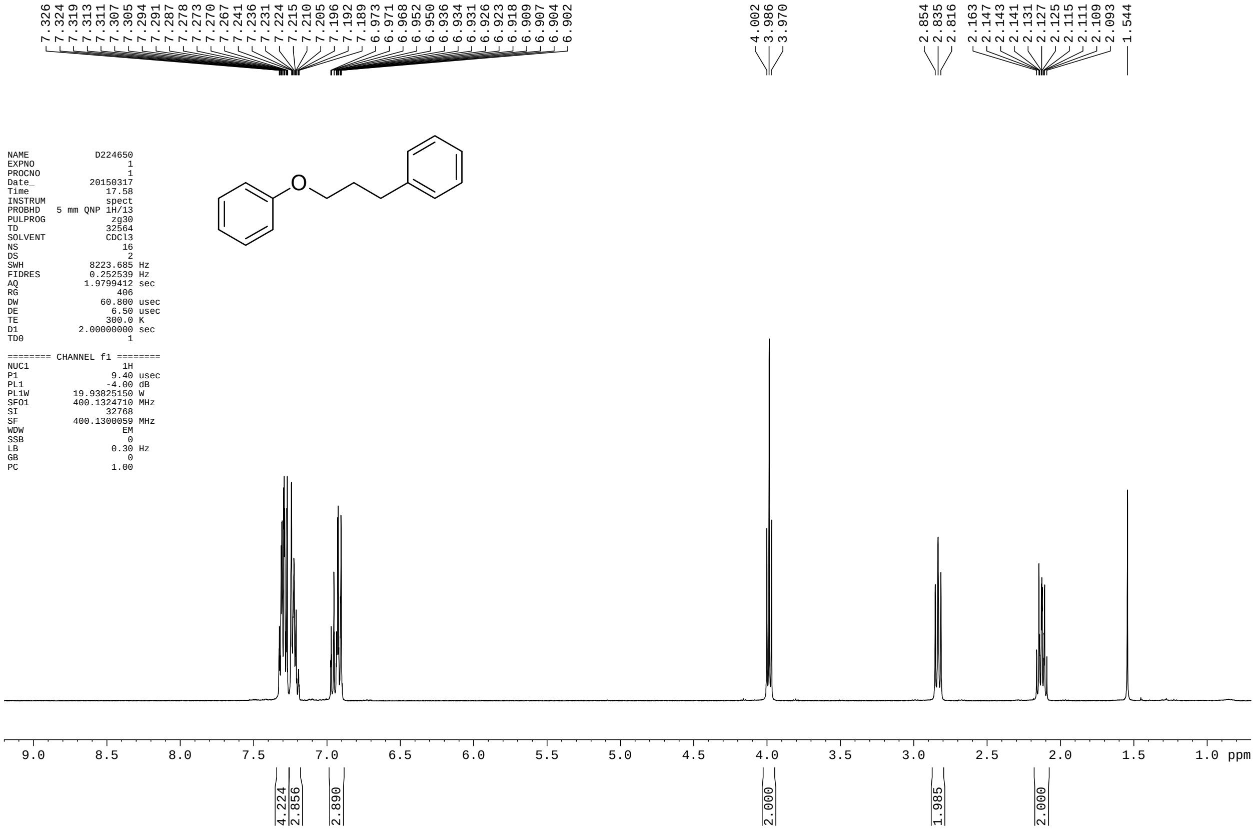
7.326 7.324 7.319 7.313 7.311 7.307 7.305 7.294 7.291 7.287 7.278 7.273 7.270 7.267 7.241 7.236 7.231 7.224 7.215 7.210 7.205 7.196 7.192 7.189 6.973 6.971 6.968 6.952 6.950 6.936 6.934 6.931 6.926 6.923 6.918 6.909 6.907 6.904 6.902
4.002 3.986 3.970
2.854 2.835 2.816 2.163 2.147 2.143 2.141 2.131 2.127 2.125 2.115 2.111 2.109 2.093 1.544
O
NAME D224650
EXPNO 1
PROCNO 1
Date_ 20150317
Time 17.58
INSTRUM spect
PROBHD 5 mm QNP 1H/13
PULPROG zg30
TD 32564
SOLVENT CDCl3
NS 16
DS 2
SWH 8223.685 Hz
FIDRES 0.252539 Hz
AQ 1.9799412 sec
RG 406
DW 60.800 usec
DE 6.50 usec
TE 300.0 K
D1 2.00000000 sec
TD0 1
======== CHANNEL f1 ========
NUC1 1H
P1 9.40 usec
PL1 -4.00 dB
PL1W 19.93825150 W
SFO1 400.1324710 MHz
SI 32768
SF 400.1300059 MHz
WDW EM
SSB 0
LB 0.30 Hz
GB 0
PC 1.00
9.0 8.5 8.0 7.5 7.0 6.5 6.0 5.5 5.0 4.5 4.0 3.5 3.0 2.5 2.0 1.5 1.0 ppm
4.224 2.856 2.890 2.000 1.985 2.000

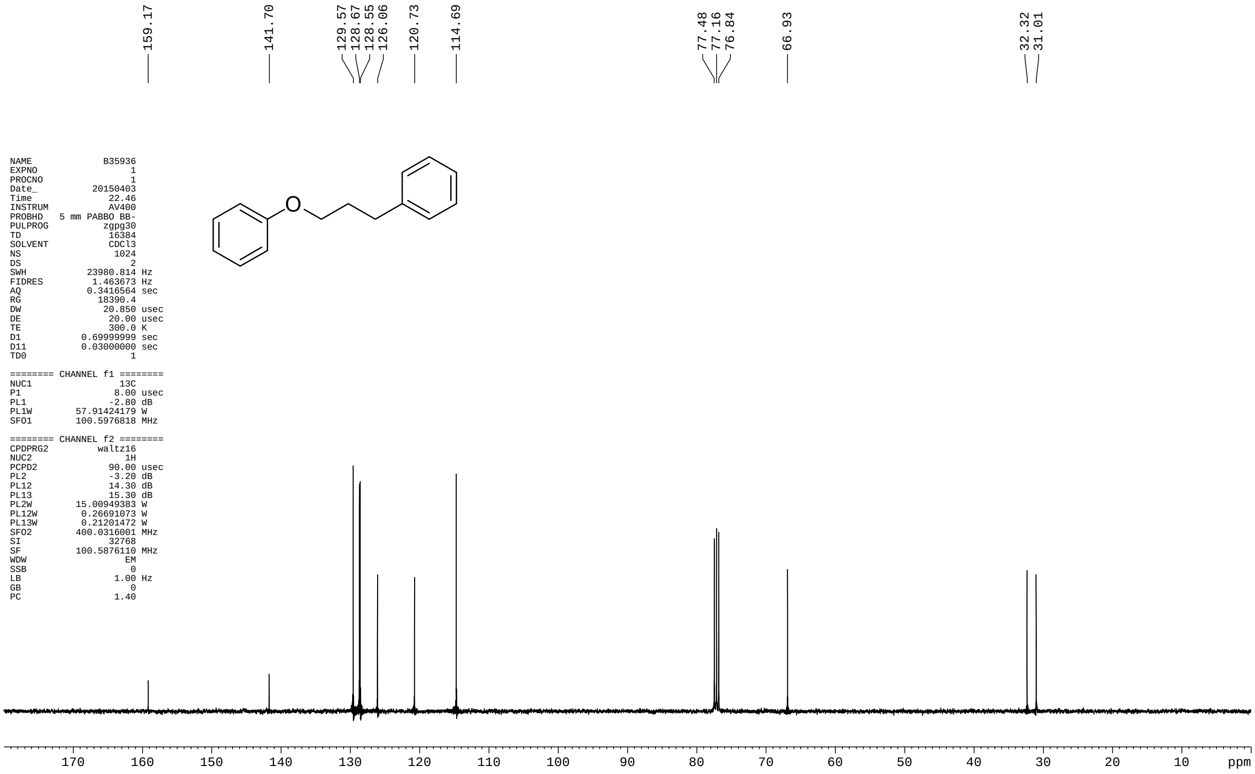
159.17 141.70 129.57 128.67 128.55 126.06 120.73 114.69 77.48 77.16 76.84 66.93 32.32 31.01
O
NAME B35936
EXPNO 1
PROCNO 1
Date_ 20150403
Time 22.46
INSTRUM AV400
PROBHD 5 mm PABBO BB-
PULPROG zgpg30
TD 16384
SOLVENT CDCl3
NS 1024
DS 2
SWH 23980.814 Hz
FIDRES 1.463673 Hz
AQ 0.3416564 sec
RG 18390.4
DW 20.850 usec
DE 20.00 usec
TE 300.0 K
D1 0.69999999 sec
D11 0.03000000 sec
TD0 1
======== CHANNEL f1 ========
NUC1 13C
P1 8.00 usec
PL1 -2.80 dB
PL1W 57.91424179 W
SFO1 100.5976818 MHz
======== CHANNEL f2 ========
CPDPRG2 waltz16
NUC2 1H
PCPD2 90.00 usec
PL2 -3.20 dB
PL12 14.30 dB
PL13 15.30 dB
PL2W 15.00949383 W
PL12W 0.26691073 W
PL13W 0.21201472 W
SFO2 400.0316001 MHz
SI 32768
SF 100.5876110 MHz
WDW EM
SSB 0
LB 1.00 Hz
GB 0
PC 1.40
170 160 150 140 130 120 110 100 90 80 70 60 50 40 30 20 10 ppm

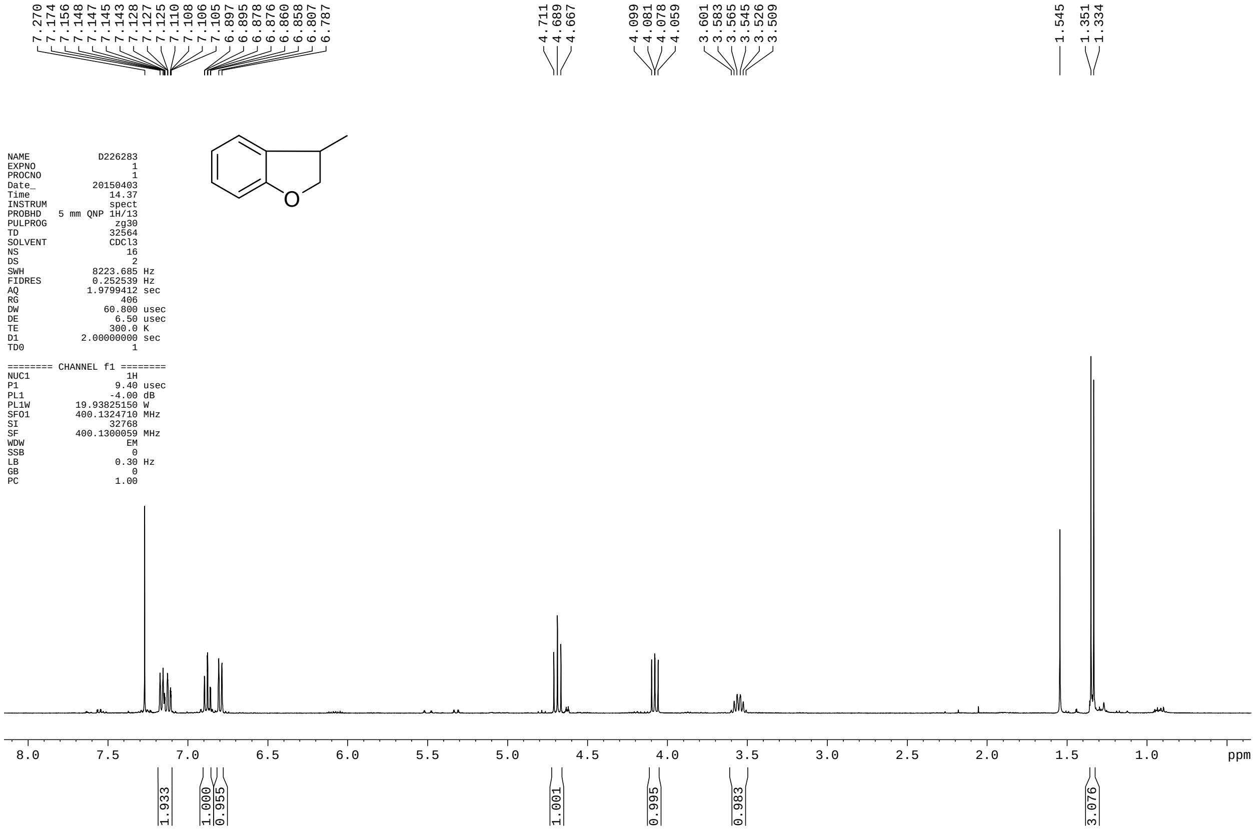
7.270
7.174
7.156
7.148
7.147
7.145
7.143
7.128
7.127
7.125
7.110
7.108
7.106
7.105
6.897
6.895
6.878
6.876
6.860
6.858
6.807
6.787
4.711
4.689
4.667
4.099
4.081
4.078
4.059
3.601
3.583
3.565
3.545
3.526
3.509
1.545
1.351
1.334
O
NAME D226283
EXPNO 1
PROCNO 1
Date_ 20150403
Time 14.37
INSTRUM spect
PROBHD 5 mm QNP 1H/13
PULPROG zg30
TD 32564
SOLVENT CDCl3
NS 16
DS 2
SWH 8223.685 Hz
FIDRES 0.252539 Hz
AQ 1.9799412 sec
RG 406
DW 60.800 usec
DE 6.50 usec
TE 300.0 K
D1 2.00000000 sec
TD0 1
======== CHANNEL f1 ========
NUC1 1H
P1 9.40 usec
PL1 -4.00 dB
PL1W 19.93825150 W
SFO1 400.1324710 MHz
SI 32768
SF 400.1300059 MHz
WDW EM
SSB 0
LB 0.30 Hz
GB 0
PC 1.00
8.0 7.5 7.0 6.5 6.0 5.5 5.0 4.5 4.0 3.5 3.0 2.5 2.0 1.5 1.0 ppm
1.933
1.000
0.955
1.001
0.995
0.983
3.076

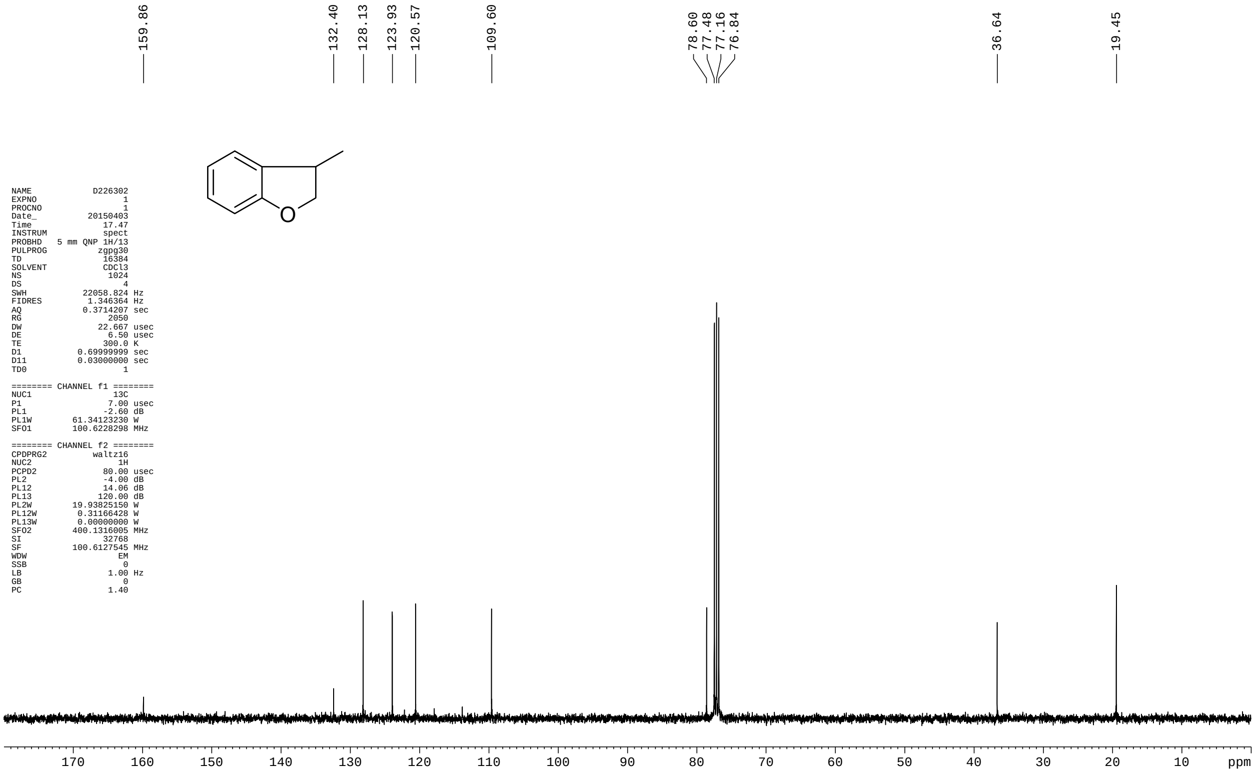
159.86
132.40
128.13
123.93
120.57
109.60
78.60
77.48
77.16
76.84
36.64
19.45
O
NAME D226302
EXPNO 1
PROCNO 1
Date_ 20150403
Time 17.47
INSTRUM spect
PROBHD 5 mm QNP 1H/13
PULPROG zgpg30
TD 16384
SOLVENT CDCl3
NS 1024
DS 4
SWH 22058.824 Hz
FIDRES 1.346364 Hz
AQ 0.3714207 sec
RG 2050
DW 22.667 usec
DE 6.50 usec
TE 300.0 K
D1 0.69999999 sec
D11 0.03000000 sec
TD0 1
======== CHANNEL f1 ========
NUC1 13C
P1 7.00 usec
PL1 -2.60 dB
PL1W 61.34123230 W
SFO1 100.6228298 MHz
======== CHANNEL f2 ========
CPDPRG2 waltz16
NUC2 1H
PCPD2 80.00 usec
PL2 -4.00 dB
PL12 14.06 dB
PL13 120.00 dB
PL2W 19.93825150 W
PL12W 0.31166428 W
PL13W 0.00000000 W
SFO2 400.1316005 MHz
SI 32768
SF 100.6127545 MHz
WDW EM
SSB 0
LB 1.00 Hz
GB 0
PC 1.40
170 160 150 140 130 120 110 100 90 80 70 60 50 40 30 20 10 ppm

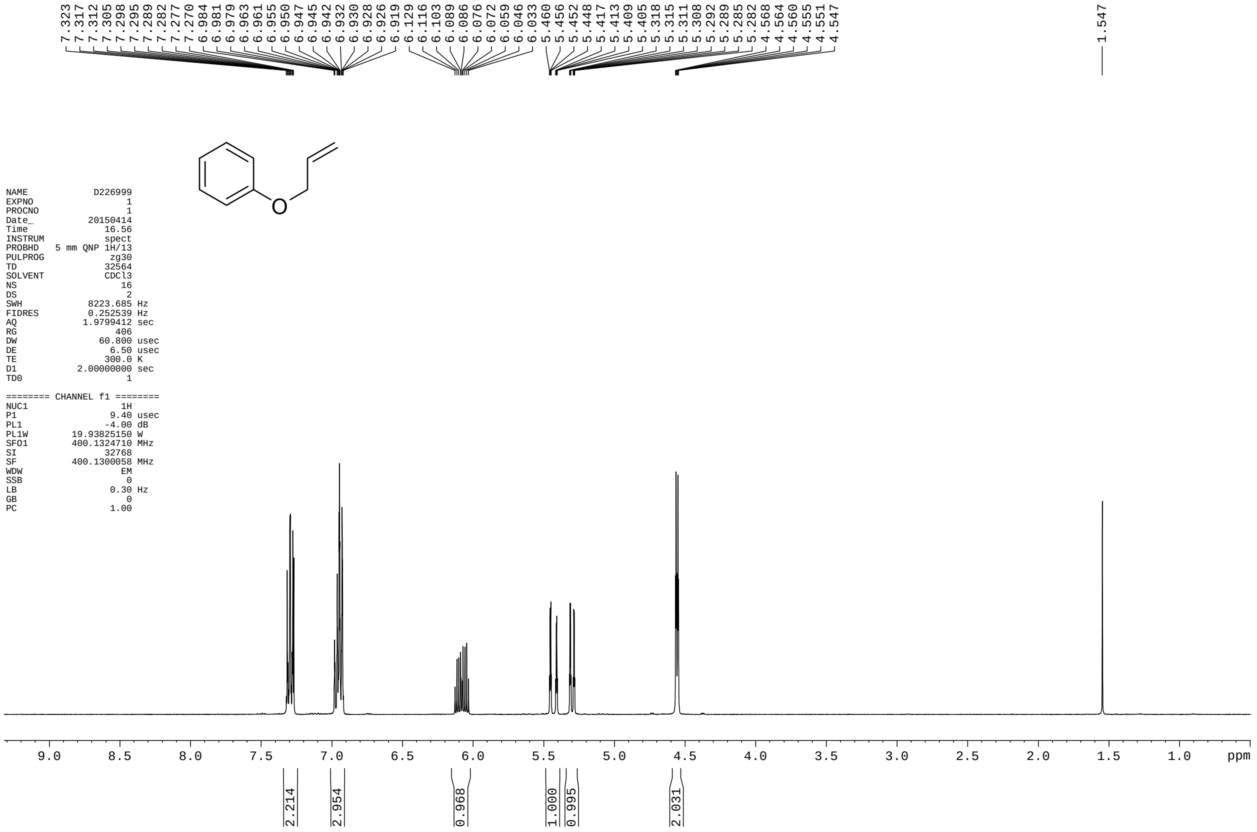
7.323 7.317 7.312 7.305 7.298 7.295 7.289 7.282 7.277 7.270 6.984 6.981 6.979 6.963 6.961 6.955 6.950 6.947 6.945 6.942 6.932 6.930 6.928 6.926 6.919 6.129 6.116 6.103 6.089 6.086 6.076 6.072 6.059 6.046 6.033 5.460 5.456 5.452 5.448 5.417 5.413 5.409 5.405 5.318 5.315 5.311 5.308 5.292 5.289 5.285 5.282 4.568 4.564 4.560 4.555 4.551 4.547
1.547
NAME D226999
EXPNO 1
PROCNO 1
Date_ 20150414
Time 16.56
INSTRUM spect
PROBHD 5 mm QNP 1H/13
PULPROG zg30
TD 32564
SOLVENT CDCl3
NS 16
DS 2
SWH 8223.685 Hz
FIDRES 0.252539 Hz
AQ 1.9799412 sec
RG 406
DW 60.800 usec
DE 6.50 usec
TE 300.0 K
D1 2.00000000 sec
TD0 1
======== CHANNEL f1 ========
NUC1 1H
P1 9.40 usec
PL1 -4.00 dB
PL1W 19.93825150 W
SFO1 400.1324710 MHz
SI 32768
SF 400.1300058 MHz
WDW EM
SSB 0
LB 0.30 Hz
GB 0
PC 1.00
O
9.0 8.5 8.0 7.5 7.0 6.5 6.0 5.5 5.0 4.5 4.0 3.5 3.0 2.5 2.0 1.5 1.0 ppm
2.214 2.954 0.968 1.000 0.995 2.031

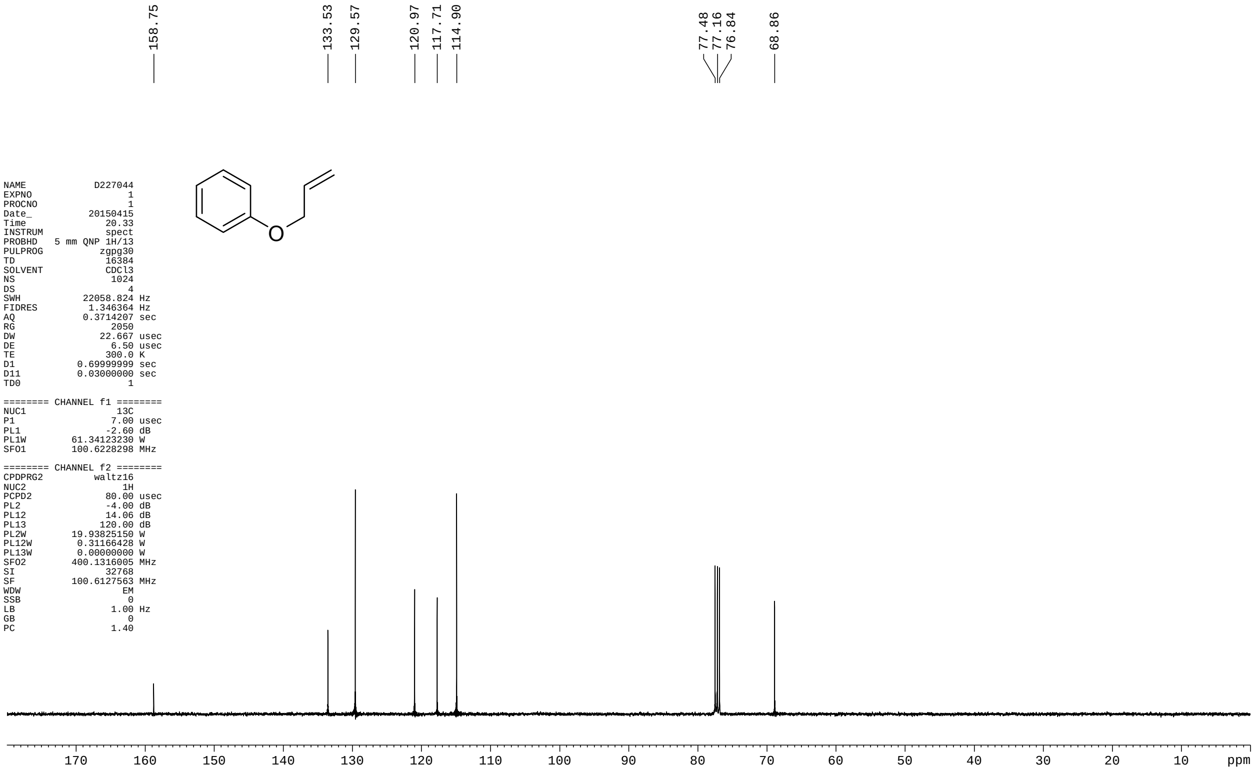
158.75 133.53 129.57 120.97 117.71 114.90 77.48 77.16 76.84 68.86
NAME D227044
EXPNO 1
PROCNO 1
Date_ 20150415
Time 20.33
INSTRUM spect
PROBHD 5 mm QNP 1H/13
PULPROG zgpg30
TD 16384
SOLVENT CDCl3
NS 1024
DS 4
SWH 22058.824 Hz
FIDRES 1.346364 Hz
AQ 0.3714207 sec
RG 2050
DW 22.667 usec
DE 6.50 usec
TE 300.0 K
D1 0.69999999 sec
D11 0.03000000 sec
TD0 1
======== CHANNEL f1 ========
NUC1 13C
P1 7.00 usec
PL1 -2.60 dB
PL1W 61.34123230 W
SFO1 100.6228298 MHz
======== CHANNEL f2 ========
CPDPRG2 waltz16
NUC2 1H
PCPD2 80.00 usec
PL2 -4.00 dB
PL12 14.06 dB
PL13 120.00 dB
PL2W 19.93825150 W
PL12W 0.31166428 W
PL13W 0.00000000 W
SFO2 400.1316005 MHz
SI 32768
SF 100.6127563 MHz
WDW EM
SSB 0
LB 1.00 Hz
GB 0
PC 1.40
O
170 160 150 140 130 120 110 100 90 80 70 60 50 40 30 20 10 ppm

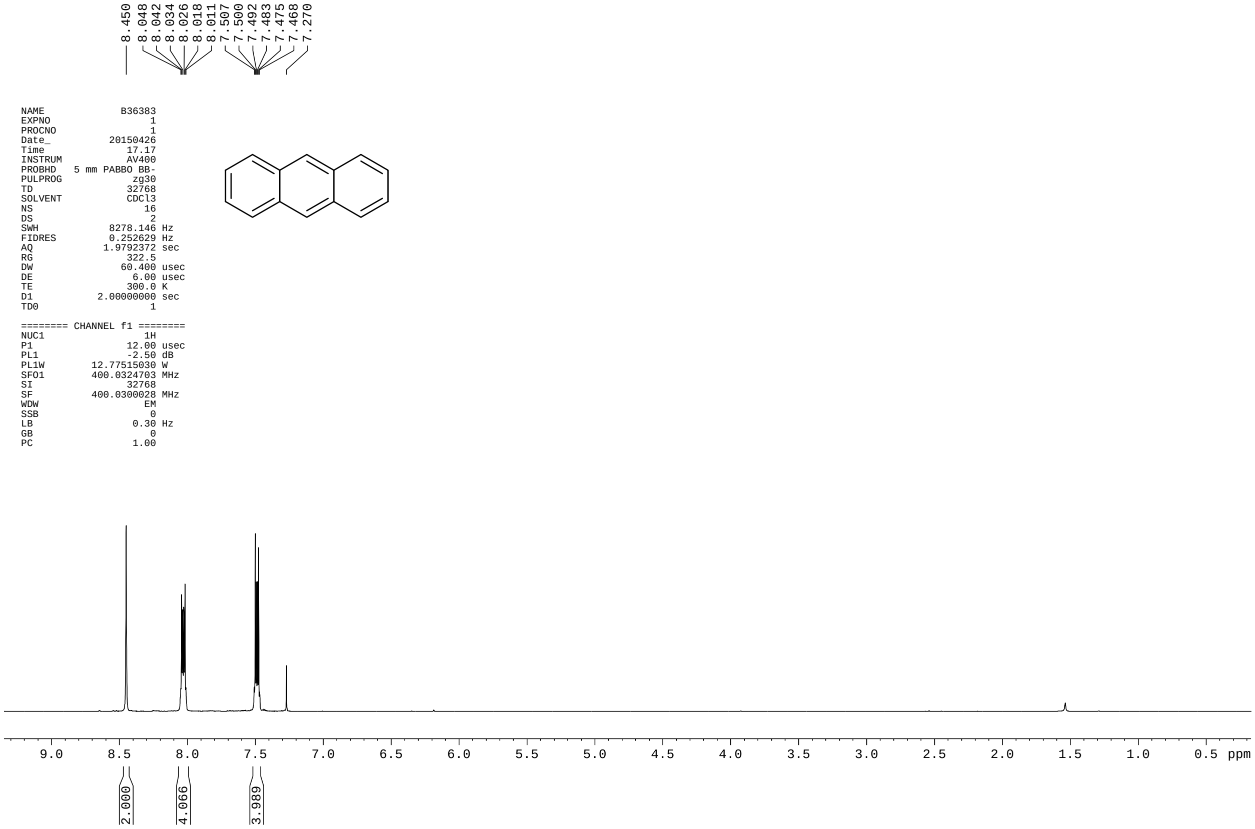
8.450
8.048
8.042
8.034
8.026
8.018
8.011
7.507
7.500
7.492
7.483
7.475
7.468
7.270
NAME B36383
EXPNO 1
PROCNO 1
Date_ 20150426
Time 17.17
INSTRUM AV400
PROBHD 5 mm PABBO BB-
PULPROG zg30
TD 32768
SOLVENT CDCl3
NS 16
DS 2
SWH 8278.146 Hz
FIDRES 0.252629 Hz
AQ 1.9792372 sec
RG 322.5
DW 60.400 usec
DE 6.00 usec
TE 300.0 K
D1 2.00000000 sec
TD0 1
======== CHANNEL f1 ========
NUC1 1H
P1 12.00 usec
PL1 -2.50 dB
PL1W 12.77515030 W
SFO1 400.0324703 MHz
SI 32768
SF 400.0300028 MHz
WDW EM
SSB 0
LB 0.30 Hz
GB 0
PC 1.00
9.0 8.5 8.0 7.5 7.0 6.5 6.0 5.5 5.0 4.5 4.0 3.5 3.0 2.5 2.0 1.5 1.0 0.5 ppm
2.000
4.066
3.989

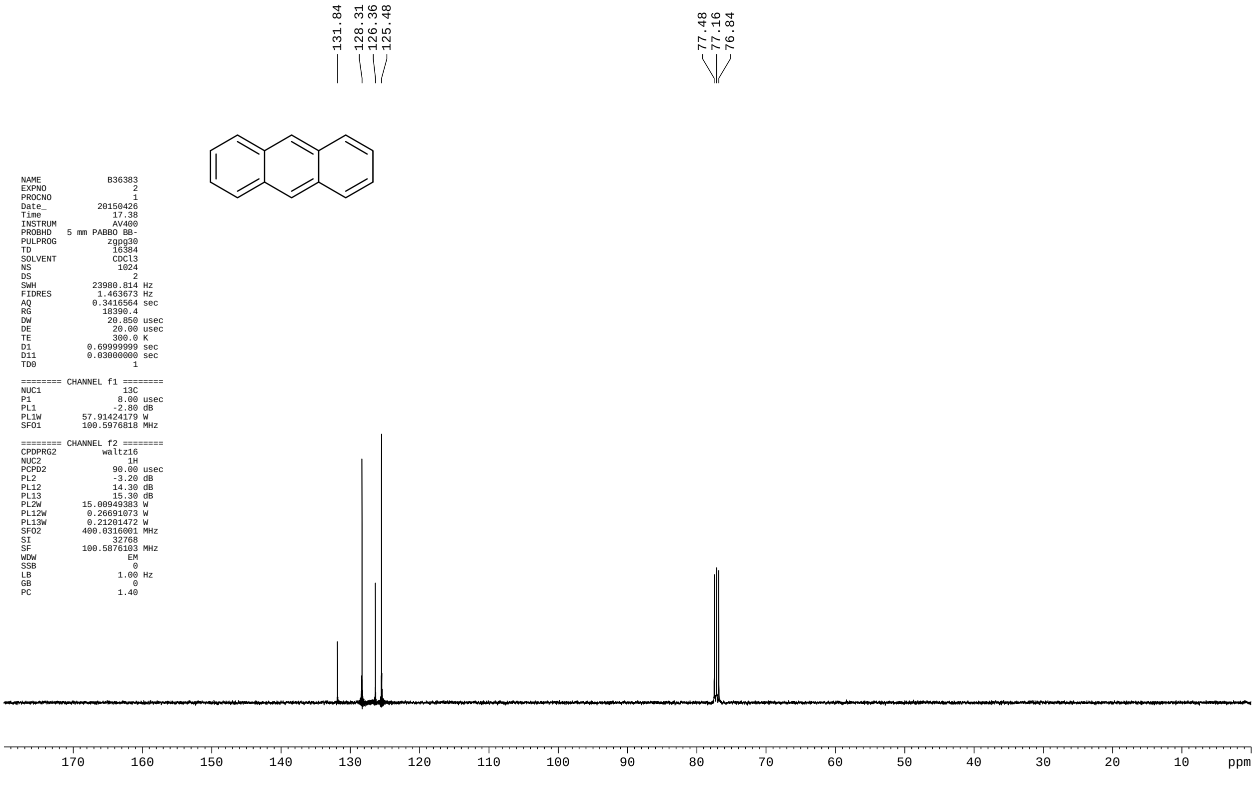
131.84
128.31
126.36
125.48
77.48
77.16
76.84
NAME B36383
EXPNO 2
PROCNO 1
Date_ 20150426
Time 17.38
INSTRUM AV400
PROBHD 5 mm PABBO BB-
PULPROG zgpg30
TD 16384
SOLVENT CDCl3
NS 1024
DS 2
SWH 23980.814 Hz
FIDRES 1.463673 Hz
AQ 0.3416564 sec
RG 18390.4
DW 20.850 usec
DE 20.00 usec
TE 300.0 K
D1 0.69999999 sec
D11 0.03000000 sec
TD0 1
======== CHANNEL f1 ========
NUC1 13C
P1 8.00 usec
PL1 -2.80 dB
PL1W 57.91424179 W
SFO1 100.5976818 MHz
======== CHANNEL f2 ========
CPDPRG2 waltz16
NUC2 1H
PCPD2 90.00 usec
PL2 -3.20 dB
PL12 14.30 dB
PL13 15.30 dB
PL2W 15.00949383 W
PL12W 0.26691073 W
PL13W 0.21201472 W
SFO2 400.0316001 MHz
SI 32768
SF 100.5876103 MHz
WDW EM
SSB 0
LB 1.00 Hz
GB 0
PC 1.40
170 160 150 140 130 120 110 100 90 80 70 60 50 40 30 20 10 ppm

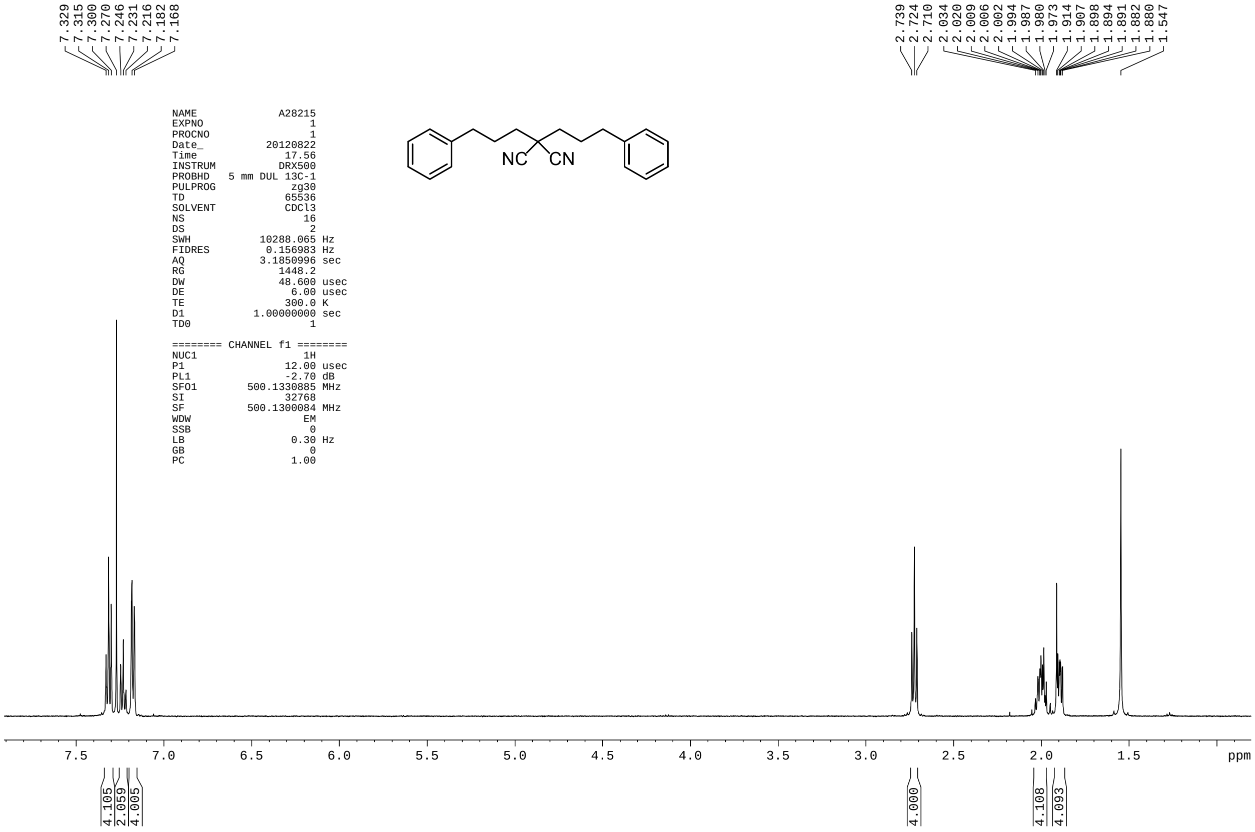
7.329
7.315
7.300
7.270
7.246
7.231
7.216
7.182
7.168
2.739
2.724
2.710
2.034
2.020
2.009
2.006
2.002
1.994
1.987
1.980
1.973
1.914
1.907
1.898
1.894
1.891
1.882
1.880
1.547
NAME A28215
EXPNO 1
PROCNO 1
Date_ 20120822
Time 17.56
INSTRUM DRX500
PROBHD 5 mm DUL 13C-1
PULPROG zg30
TD 65536
SOLVENT CDCl3
NS 16
DS 2
SWH 10288.065 Hz
FIDRES 0.156983 Hz
AQ 3.1850996 sec
RG 1448.2
DW 48.600 usec
DE 6.00 usec
TE 300.0 K
D1 1.00000000 sec
TD0 1
======== CHANNEL f1 ========
NUC1 1H
P1 12.00 usec
PL1 -2.70 dB
SFO1 500.1330885 MHz
SI 32768
SF 500.1300084 MHz
WDW EM
SSB 0
LB 0.30 Hz
GB 0
PC 1.00
NC
CN
7.5
7.0
6.5
6.0
5.5
5.0
4.5
4.0
3.5
3.0
2.5
2.0
1.5
ppm
4.105
2.059
4.005
4.000
4.108
4.093

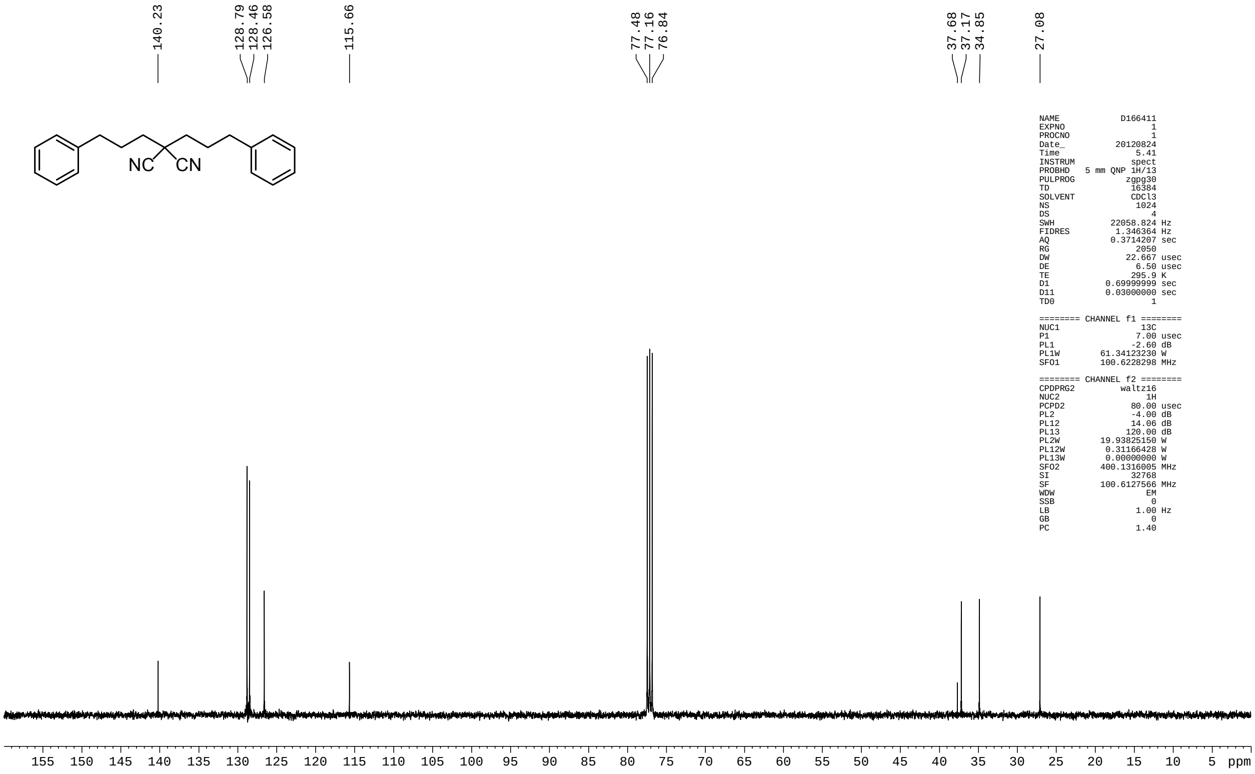
140.23
128.79
128.46
126.58
115.66
77.48
77.16
76.84
37.68
37.17
34.85
27.08
NC
CN
NAME D166411
EXPNO 1
PROCNO 1
Date_ 20120824
Time 5.41
INSTRUM spect
PROBHD 5 mm QNP 1H/13
PULPROG zgpg30
TD 16384
SOLVENT CDCl3
NS 1024
DS 4
SWH 22058.824 Hz
FIDRES 1.346364 Hz
AQ 0.3714207 sec
RG 2050
DW 22.667 usec
DE 6.50 usec
TE 295.9 K
D1 0.69999999 sec
D11 0.03000000 sec
TD0 1
======== CHANNEL f1 ========
NUC1 13C
P1 7.00 usec
PL1 -2.60 dB
PL1W 61.34123230 W
SFO1 100.6228298 MHz
======== CHANNEL f2 ========
CPDPRG2 waltz16
NUC2 1H
PCPD2 80.00 usec
PL2 -4.00 dB
PL12 14.06 dB
PL13 120.00 dB
PL2W 19.93825150 W
PL12W 0.31166428 W
PL13W 0.00000000 W
SFO2 400.1316005 MHz
SI 32768
SF 100.6127566 MHz
WDW EM
SSB 0
LB 1.00 Hz
GB 0
PC 1.40
155 150 145 140 135 130 125 120 115 110 105 100 95 90 85 80 75 70 65 60 55 50 45 40 35 30 25 20 15 10 5 ppm

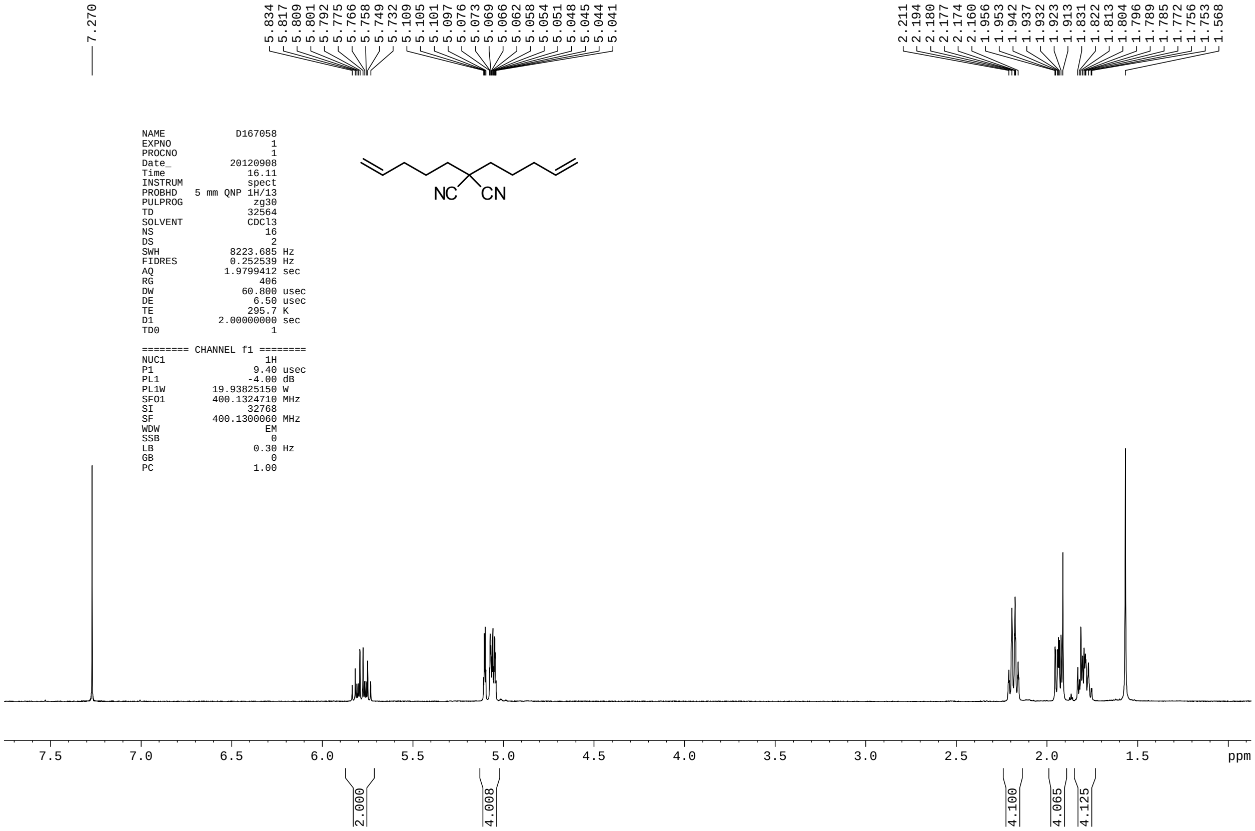
7.270
5.834 5.817 5.809 5.801 5.792 5.775 5.766 5.758 5.749 5.732 5.109 5.105 5.101 5.097 5.076 5.073 5.069 5.066 5.062 5.058 5.054 5.051 5.048 5.045 5.044 5.041
2.211 2.194 2.180 2.177 2.174 2.160 1.956 1.953 1.942 1.937 1.932 1.923 1.913 1.831 1.822 1.813 1.804 1.796 1.789 1.785 1.772 1.756 1.753 1.568
NAME D167058
EXPNO 1
PROCNO 1
Date_ 20120908
Time 16.11
INSTRUM spect
PROBHD 5 mm QNP 1H/13
PULPROG zg30
TD 32564
SOLVENT CDCl3
NS 16
DS 2
SWH 8223.685 Hz
FIDRES 0.252539 Hz
AQ 1.9799412 sec
RG 406
DW 60.800 usec
DE 6.50 usec
TE 295.7 K
D1 2.00000000 sec
TD0 1
======== CHANNEL f1 ========
NUC1 1H
P1 9.40 usec
PL1 -4.00 dB
PL1W 19.93825150 W
SFO1 400.1324710 MHz
SI 32768
SF 400.1300060 MHz
WDW EM
SSB 0
LB 0.30 Hz
GB 0
PC 1.00
NC
CN
7.5 7.0 6.5 6.0 5.5 5.0 4.5 4.0 3.5 3.0 2.5 2.0 1.5 ppm
2.000
4.008
4.100
4.065
4.125

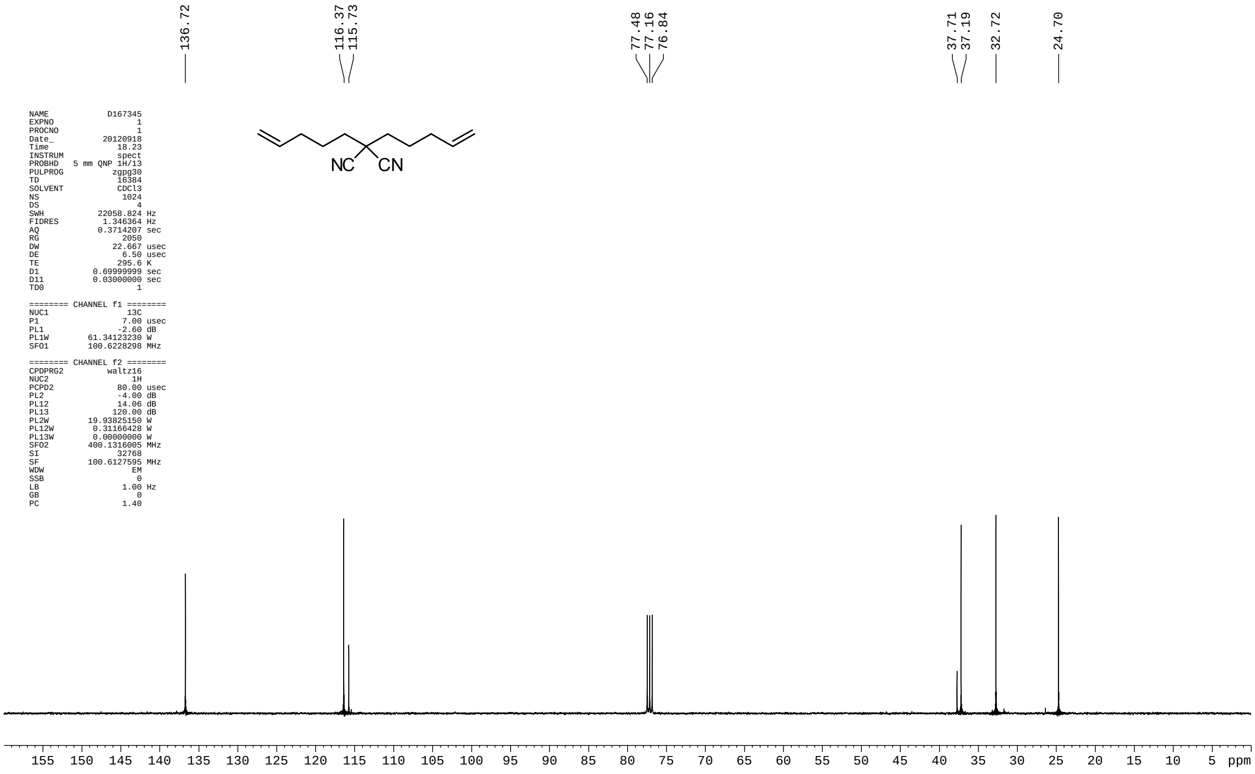
136.72
116.37 115.73
77.48 77.16 76.84
37.71 37.19 32.72
24.70
NAME D167345
EXPNO 1
PROCNO 1
Date_ 20120918
Time 18.23
INSTRUM spect
PROBHD 5 mm QNP 1H/13
PULPROG zgpg30
TD 16384
SOLVENT CDCl3
NS 1024
DS 4
SWH 22058.824 Hz
FIDRES 1.346364 Hz
AQ 0.3714207 sec
RG 2050
DW 22.667 usec
DE 6.50 usec
TE 295.6 K
D1 0.69999999 sec
D11 0.03000000 sec
TD0 1
======== CHANNEL f1 ========
NUC1 13C
P1 7.00 usec
PL1 -2.60 dB
PL1W 61.34123230 W
SFO1 100.6228298 MHz
======== CHANNEL f2 ========
CPDPRG2 waltz16
NUC2 1H
PCPD2 80.00 usec
PL2 -4.00 dB
PL12 14.06 dB
PL13 120.00 dB
PL2W 19.93825150 W
PL12W 0.31166428 W
PL13W 0.00000000 W
SFO2 400.1316005 MHz
SI 32768
SF 100.6127595 MHz
WDW EM
SSB 0
LB 1.00 Hz
GB 0
PC 1.40
NC
CN
155 150 145 140 135 130 125 120 115 110 105 100 95 90 85 80 75 70 65 60 55 50 45 40 35 30 25 20 15 10 5 ppm

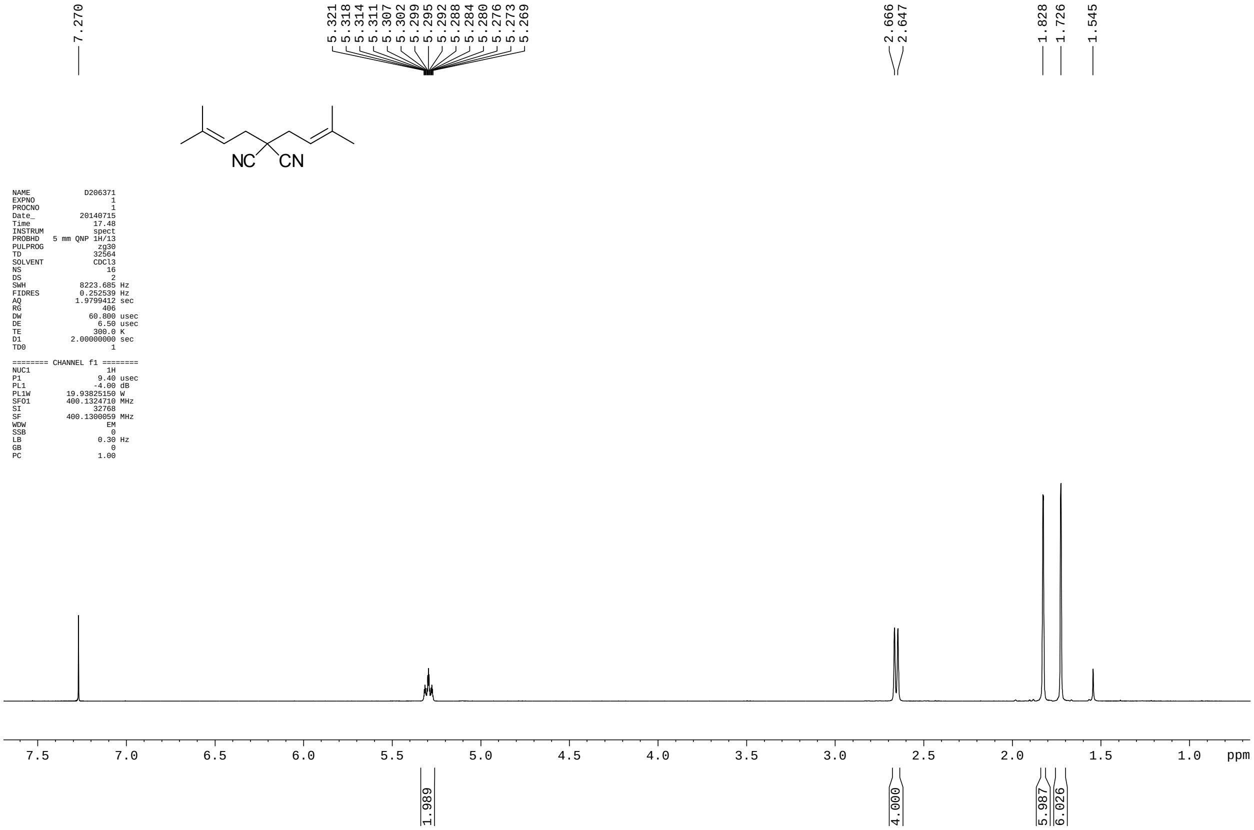
7.270
5.321
5.318
5.314
5.311
5.307
5.302
5.299
5.295
5.292
5.288
5.284
5.280
5.276
5.273
5.269
2.666
2.647
1.828
1.726
1.545
NC CN
NAME D206371
EXPNO 1
PROCNO 1
Date_ 20140715
Time 17.48
INSTRUM spect
PROBHD 5 mm QNP 1H/13
PULPROG zg30
TD 32564
SOLVENT CDCl3
NS 16
DS 2
SWH 8223.685 Hz
FIDRES 0.252539 Hz
AQ 1.9799412 sec
RG 406
DW 60.800 usec
DE 6.50 usec
TE 300.0 K
D1 2.00000000 sec
TD0 1
======== CHANNEL f1 ========
NUC1 1H
P1 9.40 usec
PL1 -4.00 dB
PL1W 19.93825150 W
SFO1 400.1324710 MHz
SI 32768
SF 400.1300059 MHz
WDW EM
SSB 0
LB 0.30 Hz
GB 0
PC 1.00
7.5 7.0 6.5 6.0 5.5 5.0 4.5 4.0 3.5 3.0 2.5 2.0 1.5 1.0 ppm
1.989
4.000
5.987
6.026

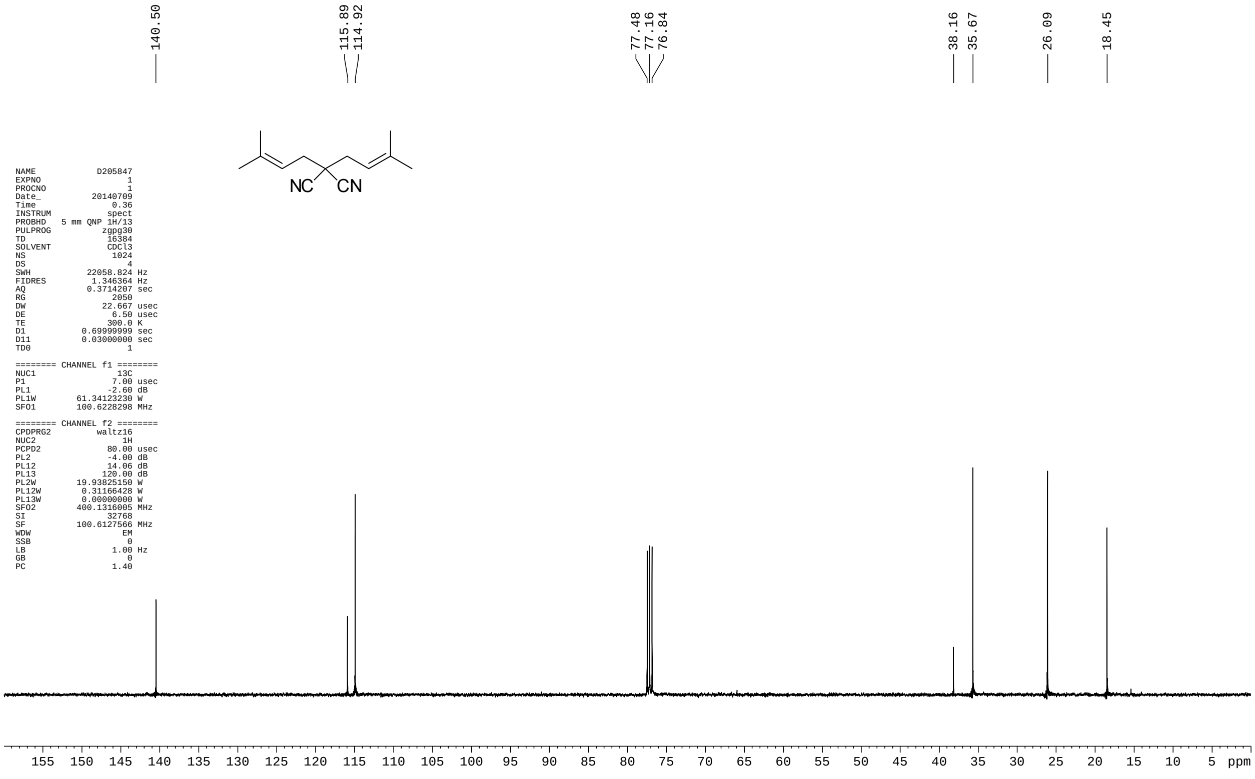
140.50
115.89
114.92
77.48
77.16
76.84
38.16
35.67
26.09
18.45
NC CN
NAME D205847
EXPNO 1
PROCNO 1
Date_ 20140709
Time 0.36
INSTRUM spect
PROBHD 5 mm QNP 1H/13
PULPROG zgpg30
TD 16384
SOLVENT CDCl3
NS 1024
DS 4
SWH 22058.824 Hz
FIDRES 1.346364 Hz
AQ 0.3714207 sec
RG 2050
DW 22.667 usec
DE 6.50 usec
TE 300.0 K
D1 0.69999999 sec
D11 0.03000000 sec
TD0 1
======== CHANNEL f1 ========
NUC1 13C
P1 7.00 usec
PL1 -2.60 dB
PL1W 61.34123230 W
SFO1 100.6228298 MHz
======== CHANNEL f2 ========
CPDPRG2 waltz16
NUC2 1H
PCPD2 80.00 usec
PL2 -4.00 dB
PL12 14.06 dB
PL13 120.00 dB
PL2W 19.93825150 W
PL12W 0.31166428 W
PL13W 0.00000000 W
SFO2 400.1316005 MHz
SI 32768
SF 100.6127566 MHz
WDW EM
SSB 0
LB 1.00 Hz
GB 0
PC 1.40
155 150 145 140 135 130 125 120 115 110 105 100 95 90 85 80 75 70 65 60 55 50 45 40 35 30 25 20 15 10 5 ppm

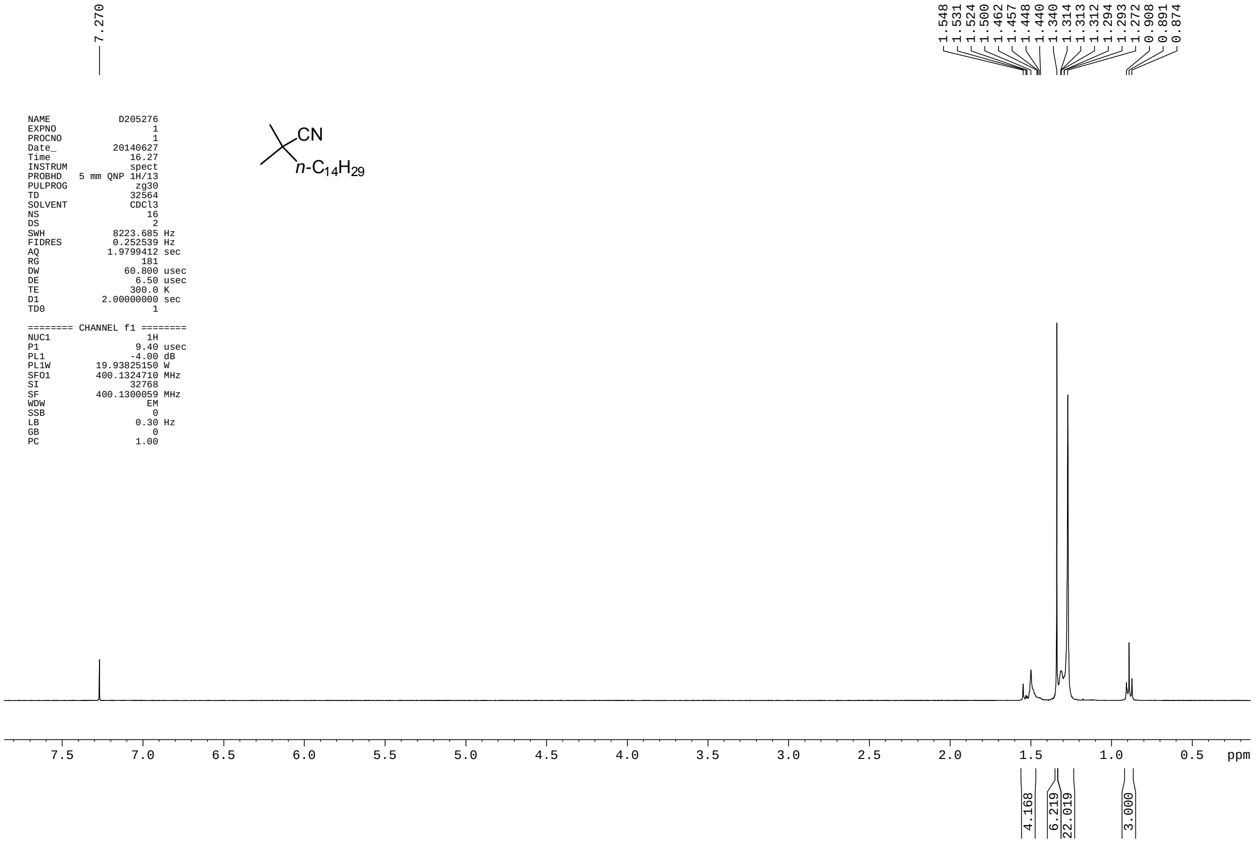
7.270
1.548 1.531 1.524 1.500 1.462 1.457 1.448 1.440 1.340 1.314 1.313 1.312 1.294 1.293 1.272 0.908 0.891 0.874
CN
n-C14H29
NAME D205276
EXPNO 1
PROCNO 1
Date_ 20140627
Time 16.27
INSTRUM spect
PROBHD 5 mm QNP 1H/13
PULPROG zg30
TD 32564
SOLVENT CDCl3
NS 16
DS 2
SWH 8223.685 Hz
FIDRES 0.252539 Hz
AQ 1.9799412 sec
RG 181
DW 60.800 usec
DE 6.50 usec
TE 300.0 K
D1 2.00000000 sec
TD0 1
======== CHANNEL f1 ========
NUC1 1H
P1 9.40 usec
PL1 -4.00 dB
PL1W 19.93825150 W
SFO1 400.1324710 MHz
SI 32768
SF 400.1300059 MHz
WDW EM
SSB 0
LB 0.30 Hz
GB 0
PC 1.00
7.5 7.0 6.5 6.0 5.5 5.0 4.5 4.0 3.5 3.0 2.5 2.0 1.5 1.0 0.5 ppm
4.168 6.219 22.019 3.000

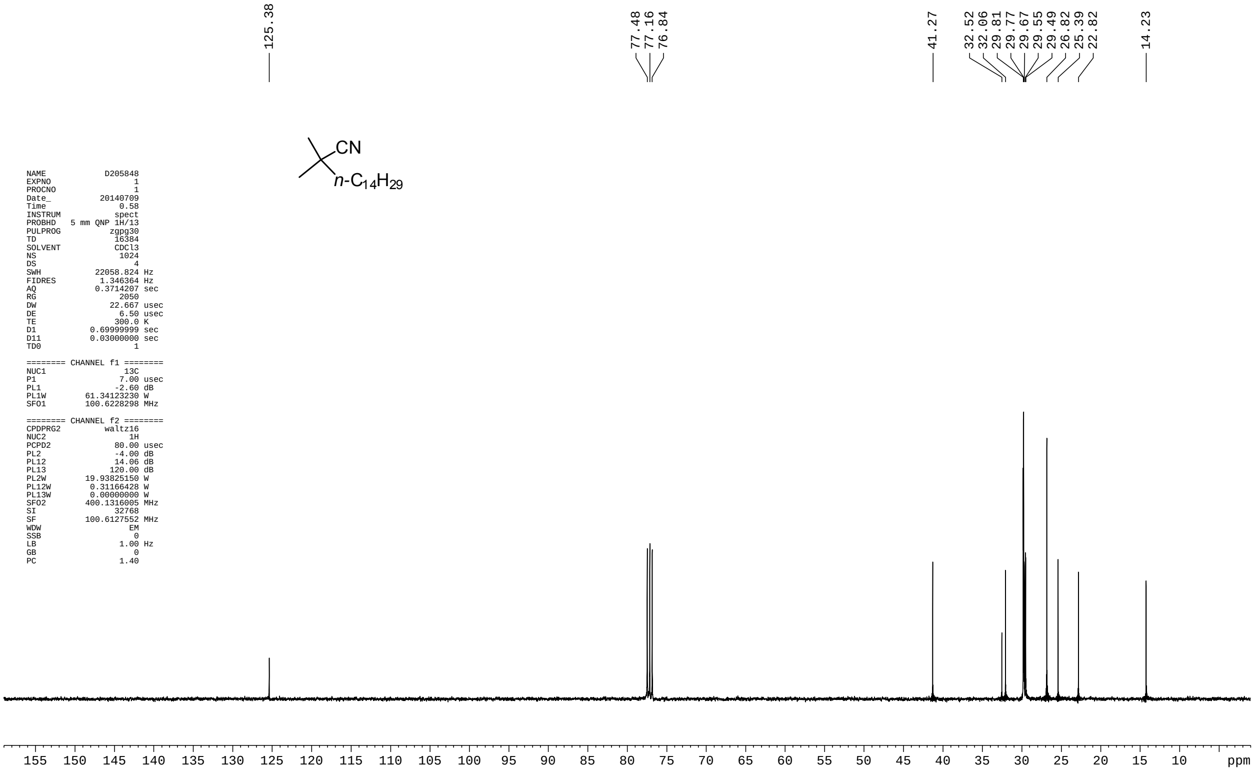
125.38
77.48 77.16 76.84
41.27
32.52 32.06 29.81 29.77 29.67 29.55 29.49 26.82 25.39 22.82
14.23
CN
n-C14H29
155 150 145 140 135 130 125 120 115 110 105 100 95 90 85 80 75 70 65 60 55 50 45 40 35 30 25 20 15 10 ppm

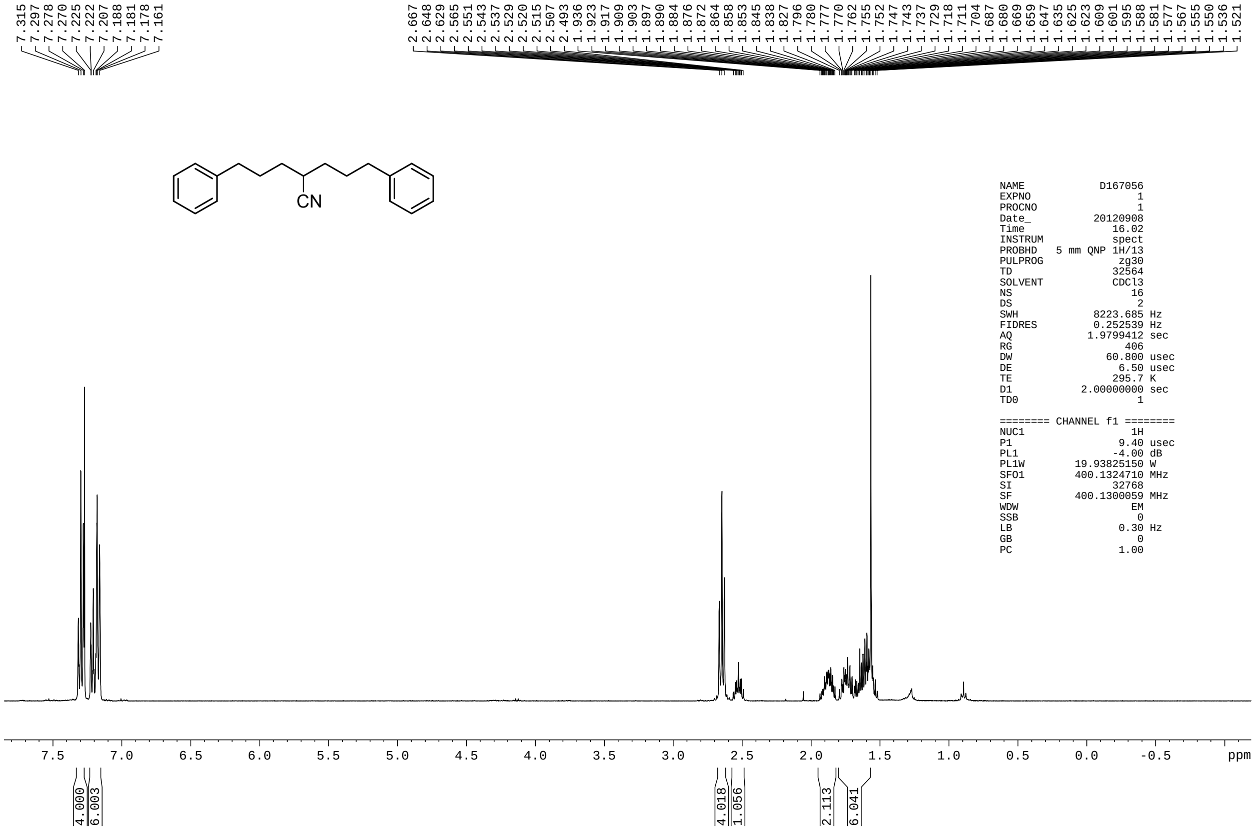
7.315
7.297
7.278
7.270
7.225
7.222
7.207
7.188
7.181
7.178
7.161
2.667
2.648
2.629
2.565
2.551
2.543
2.537
2.529
2.520
2.515
2.507
2.493
1.936
1.923
1.917
1.909
1.903
1.897
1.890
1.884
1.876
1.872
1.864
1.858
1.853
1.845
1.838
1.827
1.796
1.780
1.777
1.770
1.762
1.755
1.752
1.747
1.743
1.737
1.729
1.718
1.711
1.704
1.687
1.680
1.669
1.659
1.647
1.635
1.625
1.623
1.609
1.601
1.595
1.588
1.581
1.577
1.567
1.555
1.550
1.536
1.521
CN
NAME D167056
EXPNO 1
PROCNO 1
Date_ 20120908
Time 16.02
INSTRUM spect
PROBHD 5 mm QNP 1H/13
PULPROG zg30
TD 32564
SOLVENT CDCl3
NS 16
DS 2
SWH 8223.685 Hz
FIDRES 0.252539 Hz
AQ 1.9799412 sec
RG 406
DW 60.800 usec
DE 6.50 usec
TE 295.7 K
D1 2.00000000 sec
TD0 1
======== CHANNEL f1 ========
NUC1 1H
P1 9.40 usec
PL1 -4.00 dB
PL1W 19.93825150 W
SFO1 400.1324710 MHz
SI 32768
SF 400.1300059 MHz
WDW EM
SSB 0
LB 0.30 Hz
GB 0
PC 1.00
7.5 7.0 6.5 6.0 5.5 5.0 4.5 4.0 3.5 3.0 2.5 2.0 1.5 1.0 0.5 0.0 -0.5 ppm
4.000
6.003
4.018
1.056
2.113
6.041

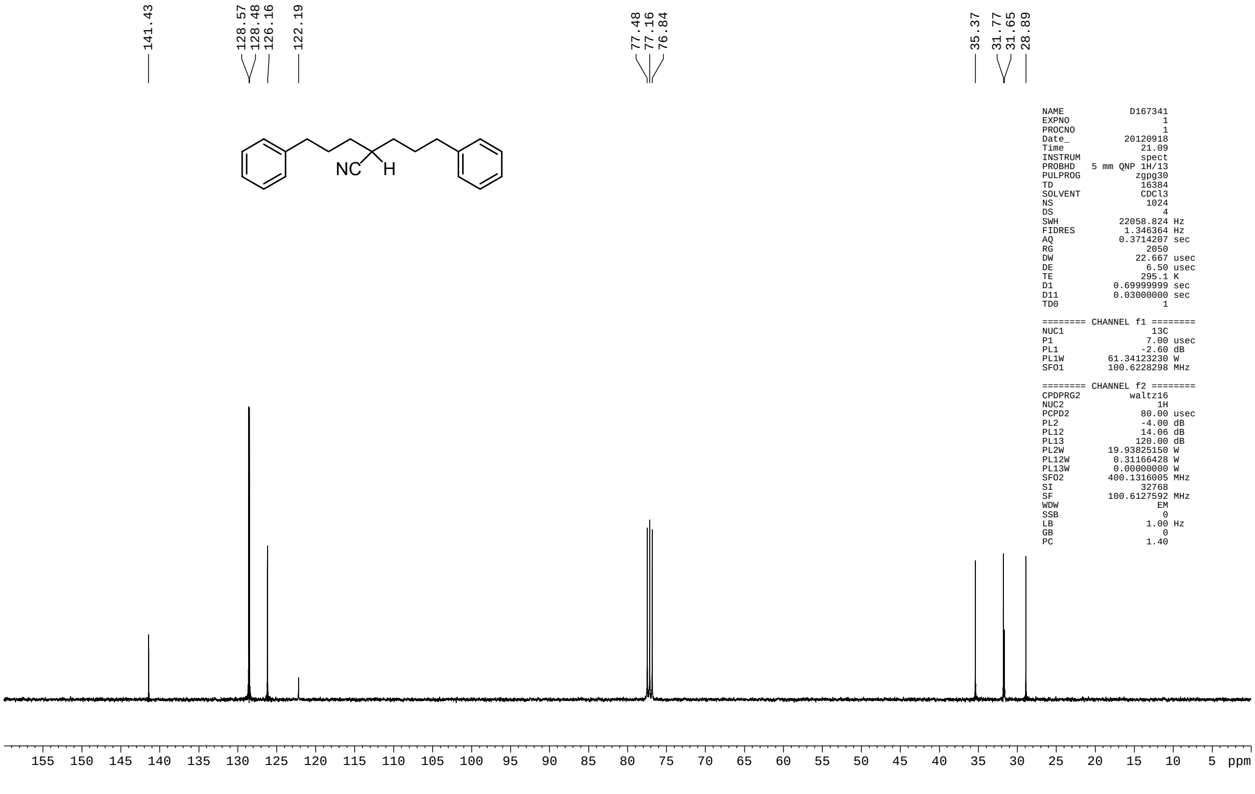
141.43
128.57
128.48
126.16
122.19
77.48
77.16
76.84
35.37
31.77
31.65
28.89
NC
H
NAME D167341
EXPNO 1
PROCNO 1
Date_ 20120918
Time 21.09
INSTRUM spect
PROBHD 5 mm QNP 1H/13
PULPROG zgpg30
TD 16384
SOLVENT CDCl3
NS 1024
DS 4
SWH 22058.824 Hz
FIDRES 1.346364 Hz
AQ 0.3714207 sec
RG 2050
DW 22.667 usec
DE 6.50 usec
TE 295.1 K
D1 0.69999999 sec
D11 0.03000000 sec
TD0 1
======== CHANNEL f1 ========
NUC1 13C
P1 7.00 usec
PL1 -2.60 dB
PL1W 61.34123230 W
SFO1 100.6228298 MHz
======== CHANNEL f2 ========
CPDPRG2 waltz16
NUC2 1H
PCPD2 80.00 usec
PL2 -4.00 dB
PL12 14.06 dB
PL13 120.00 dB
PL2W 19.93825150 W
PL12W 0.31166428 W
PL13W 0.00000000 W
SFO2 400.1316005 MHz
SI 32768
SF 100.6127592 MHz
WDW EM
SSB 0
LB 1.00 Hz
GB 0
PC 1.40
155 150 145 140 135 130 125 120 115 110 105 100 95 90 85 80 75 70 65 60 55 50 45 40 35 30 25 20 15 10 5 ppm

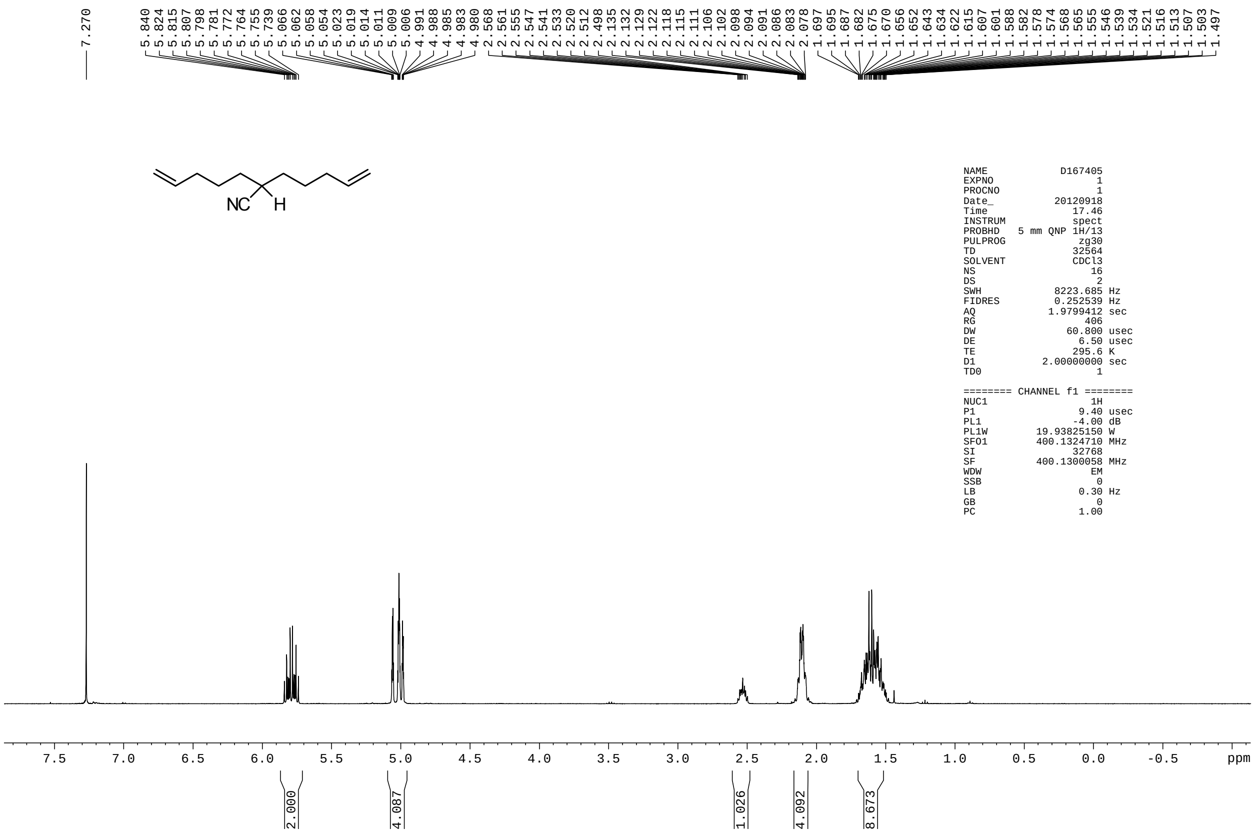
7.270
NC H
NAME D167405
EXPNO 1
PROCNO 1
Date_ 20120918
Time 17.46
INSTRUM spect
PROBHD 5 mm QNP 1H/13
PULPROG zg30
TD 32564
SOLVENT CDCl3
NS 16
DS 2
SWH 8223.685 Hz
FIDRES 0.252539 Hz
AQ 1.9799412 sec
RG 406
DW 60.800 usec
DE 6.50 usec
TE 295.6 K
D1 2.00000000 sec
TD0 1
======== CHANNEL f1 ========
NUC1 1H
P1 9.40 usec
PL1 -4.00 dB
PL1W 19.93825150 W
SFO1 400.1324710 MHz
SI 32768
SF 400.1300058 MHz
WDW EM
SSB 0
LB 0.30 Hz
GB 0
PC 1.00
2.000
4.087
1.026
4.092
8.673
ppm

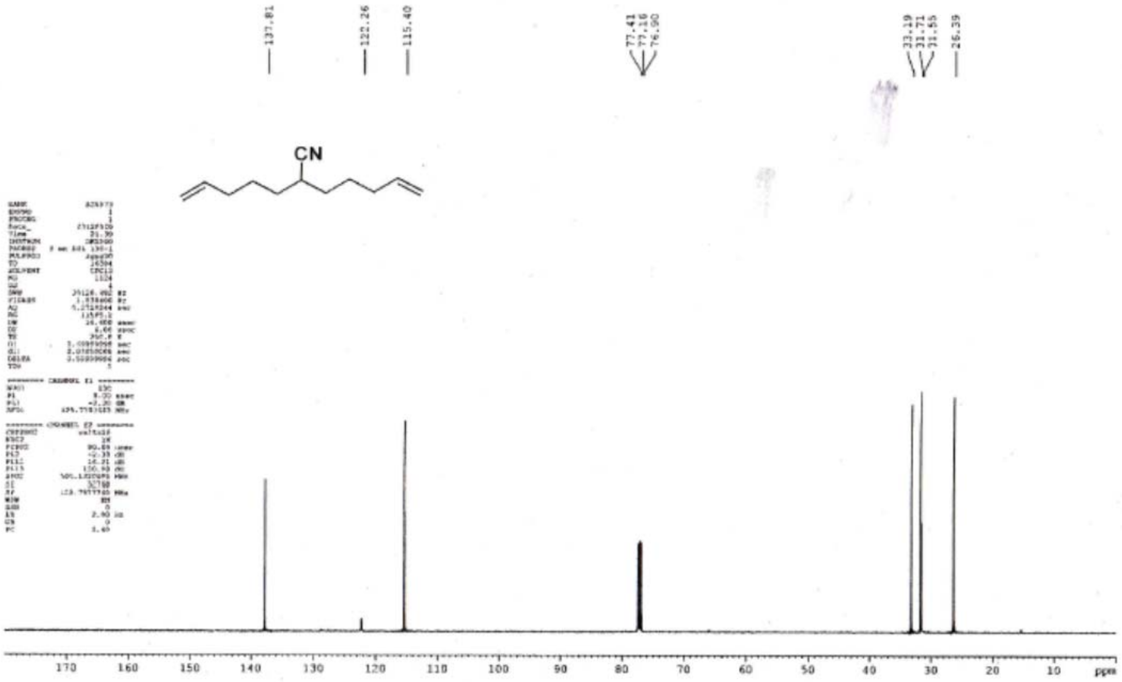
137.81
122.26
115.40
77.41
77.16
76.90
33.19
31.71
31.55
26.39
CN
ppm

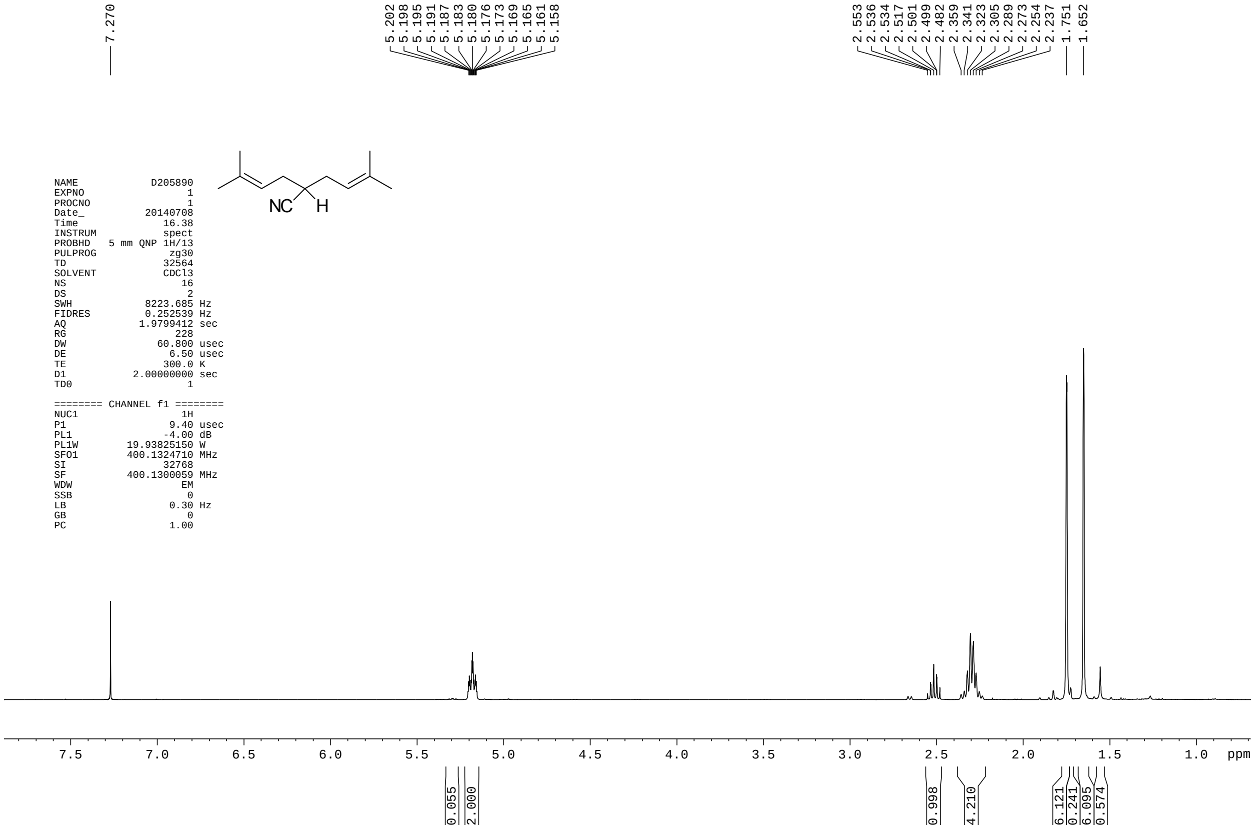
7.270
5.202 5.198 5.195 5.191 5.187 5.183 5.180 5.176 5.173 5.169 5.165 5.161 5.158
2.553 2.536 2.534 2.517 2.501 2.499 2.482 2.359 2.341 2.323 2.305 2.289 2.273 2.254 2.237 1.751 1.652
NC H
NAME D205890
EXPNO 1
PROCNO 1
Date_ 20140708
Time 16.38
INSTRUM spect
PROBHD 5 mm QNP 1H/13
PULPROG zg30
TD 32564
SOLVENT CDCl3
NS 16
DS 2
SWH 8223.685 Hz
FIDRES 0.252539 Hz
AQ 1.9799412 sec
RG 228
DW 60.800 usec
DE 6.50 usec
TE 300.0 K
D1 2.00000000 sec
TD0 1
======== CHANNEL f1 ========
NUC1 1H
P1 9.40 usec
PL1 -4.00 dB
PL1W 19.93825150 W
SFO1 400.1324710 MHz
SI 32768
SF 400.1300059 MHz
WDW EM
SSB 0
LB 0.30 Hz
GB 0
PC 1.00
7.5 7.0 6.5 6.0 5.5 5.0 4.5 4.0 3.5 3.0 2.5 2.0 1.5 1.0 ppm
0.055 2.000 0.998 4.210 6.121 0.241 6.095 0.574

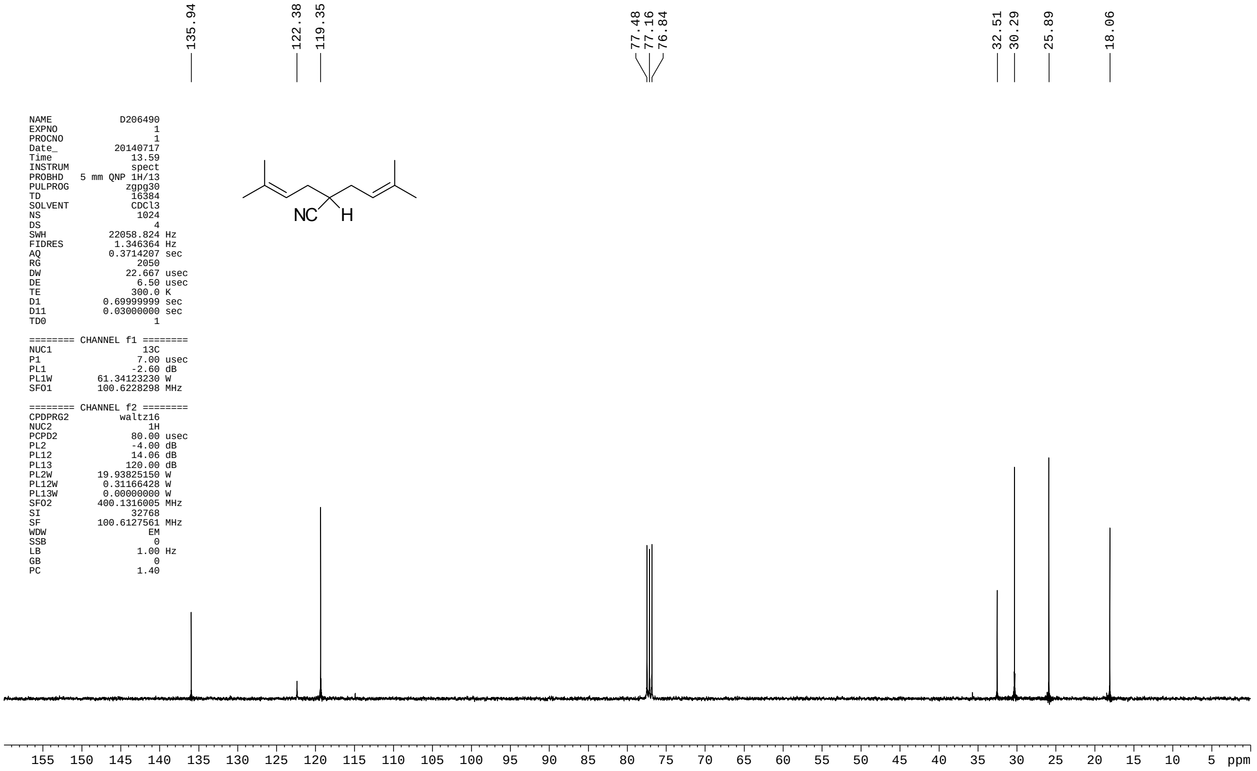
135.94
122.38 119.35
77.48 77.16 76.84
32.51 30.29 25.89
18.06
NC H
NAME D206490
EXPNO 1
PROCNO 1
Date_ 20140717
Time 13.59
INSTRUM spect
PROBHD 5 mm QNP 1H/13
PULPROG zgpg30
TD 16384
SOLVENT CDCl3
NS 1024
DS 4
SWH 22058.824 Hz
FIDRES 1.346364 Hz
AQ 0.3714207 sec
RG 2050
DW 22.667 usec
DE 6.50 usec
TE 300.0 K
D1 0.69999999 sec
D11 0.03000000 sec
TD0 1
======== CHANNEL f1 ========
NUC1 13C
P1 7.00 usec
PL1 -2.60 dB
PL1W 61.34123230 W
SFO1 100.6228298 MHz
======== CHANNEL f2 ========
CPDPRG2 waltz16
NUC2 1H
PCPD2 80.00 usec
PL2 -4.00 dB
PL12 14.06 dB
PL13 120.00 dB
PL2W 19.93825150 W
PL12W 0.31166428 W
PL13W 0.00000000 W
SFO2 400.1316005 MHz
SI 32768
SF 100.6127561 MHz
WDW EM
SSB 0
LB 1.00 Hz
GB 0
PC 1.40
155 150 145 140 135 130 125 120 115 110 105 100 95 90 85 80 75 70 65 60 55 50 45 40 35 30 25 20 15 10 5 ppm
